# Nutrient restriction-activated Fra-2 promotes tumor progression via IGF1R in miR-15a downmodulated pancreatic ductal adenocarcinoma

**DOI:** 10.1038/s41392-024-01740-4

**Published:** 2024-02-12

**Authors:** Gian Luca Rampioni Vinciguerra, Marina Capece, Luca Reggiani Bonetti, Giovanni Nigita, Federica Calore, Sydney Rentsch, Paolo Magistri, Roberto Ballarin, Fabrizio Di Benedetto, Rosario Distefano, Roberto Cirombella, Andrea Vecchione, Barbara Belletti, Gustavo Baldassarre, Francesca Lovat, Carlo M. Croce

**Affiliations:** 1 Department of Cancer Biology and Genetics and Comprehensive Cancer Center, The Ohio State University, Columbus, 43210 OH USA; 2 Department of Clinical and Molecular Medicine, Faculty of Medicine and Psychology, Sant’Andrea Hospital, University of Rome “Sapienza”, Rome, 00189 Italy; 3 Department of Diagnostic, Clinic and Public Health Medicine, University of Modena and Reggio Emilia, Modena, 41100 Italy; 4 Hepato-pancreato-biliary Surgery and Liver Transplantation Unit, University of Modena and Reggio Emilia, Modena, 41100 Italy; 5 Division of Molecular Oncology, Centro di Riferimento Oncologico di Aviano (CRO), Istituto di Ricovero e Cura a Carattere Scientifico (IRCCS), National Cancer Institute, Aviano, 33081 Italy

**Keywords:** Gastrointestinal cancer

## Abstract

Pancreatic ductal adenocarcinoma (PDAC) is a lethal disease, characterized by an intense desmoplastic reaction that compresses blood vessels and limits nutrient supplies. PDAC aggressiveness largely relies on its extraordinary capability to thrive and progress in a challenging tumor microenvironment. Dysregulation of the onco-suppressor miR-15a has been extensively documented in PDAC. Here, we identified the transcription factor Fos-related antigen-2 (Fra-2) as a miR-15a target mediating the adaptive mechanism of PDAC to nutrient deprivation. We report that the IGF1 signaling pathway was enhanced in nutrient deprived PDAC cells and that Fra-2 and IGF1R were significantly overexpressed in miR-15a downmodulated PDAC patients. Mechanistically, we discovered that miR-15a repressed IGF1R expression *via* Fra-2 targeting. In miR-15a-low context, IGF1R hyperactivated mTOR, modulated the autophagic flux and sustained PDAC growth in nutrient deprivation. In a genetic mouse model, Mir15a^KO^ PDAC showed Fra-2 and Igf1r upregulation and mTOR activation in response to diet restriction. Consistently, nutrient restriction improved the efficacy of IGF1R inhibition in a Fra-2 dependent manner. Overall, our results point to a crucial role of Fra-2 in the cellular stress response due to nutrient restriction typical of pancreatic cancer and support IGF1R as a promising and vulnerable target in miR-15a downmodulated PDAC.

## Introduction

Pancreatic ductal adenocarcinoma (PDAC) accounts for the majority of pancreatic malignancies.^1^ The incidence of PDAC is yearly increasing at a rate of 0.5% to 1.0% and is expected to become the second-leading cause of cancer-related mortality in the next few years.^1^ Early detection of PDAC represents an infrequent event and, at late stage of disease, PDAC remains elusive to treatment.^2^ Indeed, neoplastic cells show a marked resistance to current therapies.^1^ To date, the 5-year survival rate does not exceed 10% of cases.^1,2^ It is well established that the unique PDAC microenvironment plays a crucial role in the aggressiveness of the disease.^3–5^ Compared to other solid neoplasms, PDAC stands out by the abnormal prominence of its desmoplastic reaction, that frequently represents the major component of the tumor bulk.^4^ The dense extracellular matrix compresses tumor cells and blood vessels, limiting oxygen and nutrient availability in the tumor microenvironment.^3,4^ To thrive and progress under mechanical stress, hypoxia, and nutrient scarcity, PDAC cells are challenged to rapidly adopt different strategies, resulting in a plastic rewiring of transcriptomic, metabolic and signaling pathways.^3,4,6,7^ The study of PDAC adaptive mechanisms could lead to the identification of critical vulnerabilities and, as a result, novel therapeutic approaches.

MicroRNAs represent a class of small non-coding RNAs (approximately 22 nucleotides in length) that, by negatively regulating gene expression at the post-transcriptional level, mediate several biological functions.^8–12^ MicroRNAs have been reported to play key roles in the adaptive response to microenvironmental changing, hypoxia and metabolic stress in cancer and, importantly, in PDAC.^13–15^ In particular, the expression of miR-15a has been found to be downmodulated in neoplastic samples compared to the adjacent benign tissue.^16^ In this context, miR-15a mediates the suppression of several oncogenes such as BMI-1.^17^ Therefore, miR-15a dysregulation promotes cell cycle progression, cell viability, epithelial-mesenchymal transition and drug resistance in PDAC.^16–18^ However, the possible role of miR-15a in the stress response of PDAC to nutrient restriction is still unexplored.

Among the putative targets of miR-15a, we identified Fos-related antigen-2 (hereafter, Fra-2) as a promising player in the adaptive mechanism of PDAC to microenvironmental stress and, specifically, to nutrient deprivation. Fra-2 is a member of the Activator Protein-1 (AP-1) transcription factor family and its activity is frequently enhanced in cancer, representing a critical mediator of tumorigenesis in RAS-mutated malignancies.^19^ Likewise the other AP-1 proteins, Fra-2 acts as an immediate early gene, rapidly activated as a result of a wide range of stimuli, including inflammation, cellular stress, and different kinds of environmental cues.^19^ For instance, exposure to ultraviolet irradiation and oxidative stress induce the AP-1 activation in fibroblasts that, in turn, prevents cell death.^20^ In both physiological and neoplastic models, Fra-2 activity has been involved in the regulation of autophagic flux,^21^ hypoxia,^22^ and in response to oxidative stress.^23^ and DNA damage.^24^ Over the las 4 years, several studies have underscored a role of Fra-2 in PDAC.^25–28^ First, Fra-2 expression levels are increased and correlate with poor prognosis in PDAC patients.^25^ The Fra-2 consensus sequence represents one of the most accessible transcription factor binding motif in transformed pancreatic cells compared to the normal epithelium,^25^ supporting the possibility that Fra-2 can be primarily involved in reprogramming the transcriptomic landscape of PDAC. In vitro, Fra-2 expression is strongly triggered by hypoxia and serum deprivation in PDAC cells^26^ and, once activated, Fra-2 can sustain either pro- or anti-inflammatory programs, promoting DNA damage or immune evasion, depending on the context.^27,28^ Collectively, this converging evidence supports a critical role of Fra-2 in PDAC and, potentially, in the stress response to nutrient shortage.

Herein, we report the identification of miR-15a and its target Fra-2 as main regulators of the IGF1 signaling pathway and describe the mechanism whereby they orchestrate the cell-stress adaptation to nutrient restriction in PDAC.

## Results

### IGF1 signaling pathway is activated in response to nutrient deprivation in PDAC, *via* miR-15a/Fra-2 axis

To investigate the possible role of the onco-suppressor miR-15a in the PDAC adaptation to nutrient shortage, we selected Fra-2 as a promising miR-15a target. Belonging to AP-1 family, Fra-2 is a transcription factor acting as an immediate early gene in the cellular stress response to hypoxia and serum deprivation^26^ and its consensus sequence is one of the most accessible motifs in the chromatin of PDAC.^25^ To assess the relevance of this potential miR-15a/Fra-2 axis, we explored the expression of miR-15a and Fra-2 in PDAC patients from the TCGA consortium. As already reported,^18^ low levels of miR-15a correlated with poor prognosis in PDAC patients (Supplementary Fig. S1a). In the same cohort, we also observed a shorter overall survival in PDAC patients with high expression of Fra-2 (Supplementary Fig. S1b) and a significant anti-correlation between miR-15a and Fra-2 levels (Supplementary Fig. S1c). These findings suggested a functional role of miR-15a in the regulation of Fra-2 expression in PDAC. To validate that miR-15a was able to directly target Fra-2, we cloned the 3′UTR of Fra-2 containing the miR-15a seed sequence into a luciferase (Luc) vector and observed that Luc activity was significantly decreased by miR-15a expression and rescued by miR-15a-binding site deletion (Supplementary Fig. S1d, e). Consistently, the ectopic overexpression of miR-15a significantly decreased Fra-2 levels in MIA PaCa-2 PDAC cells (Supplementary Fig. S1f-h).

**Fig. 1:**
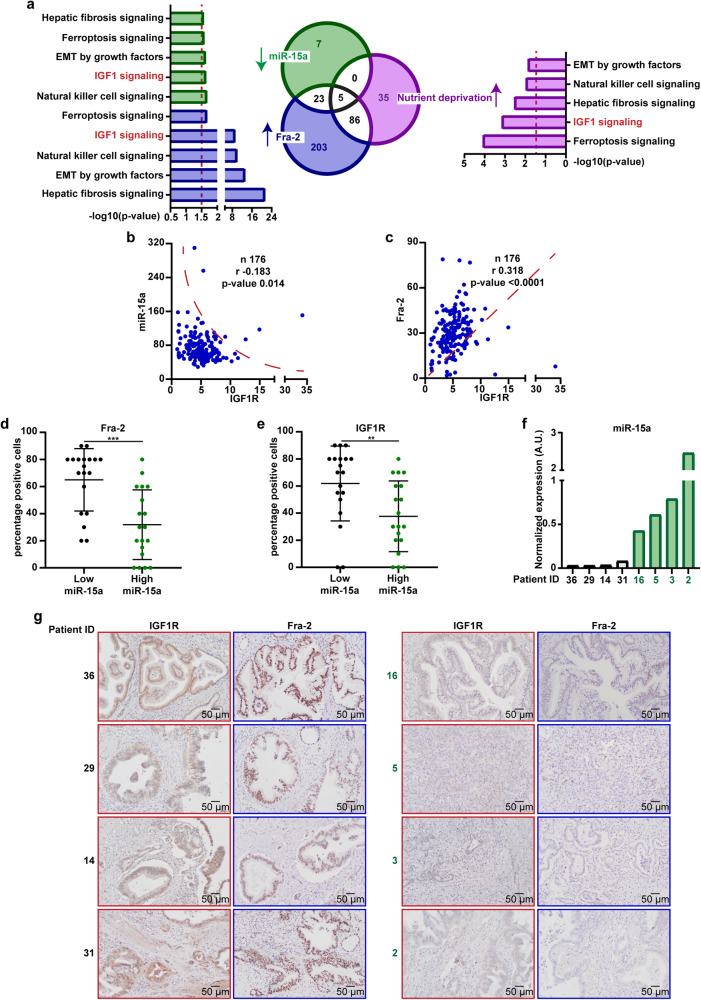
IGF1 signaling pathway is activated in response to nutrient deprivation and potentially regulated by miR-15a and Fra-2 in PDAC. **a** Venn diagram showing the number of molecular pathways enriched in nutrient deprived PDAC cells (purple) and potentially upregulated by miR-15a downregulation (green) and Fra-2 expression (blue) in PDAC patients from the TCGA dataset (*n* = 176). For nutrient deprivation (purple), data were obtained by performing ingenuity pathway analysis (IPA®) on the overexpressed genes in AsPC-1 and MIA PaCa-2 cell lines, cultured in nutrient deprivation (0% FBS) for 72 h. For miR-15a (green) and Fra-2 (blue), data were obtained by performing IPA® analyses on the genes significantly correlated with Fra-2 expression and the putative target genes inversely correlated with miR-15a levels in PDAC patients from the TCGA dataset. Histograms showing the significance of the five commonly activated pathways, as obtained by the Venn diagram. Dotted red lines indicate the established threshold of 1.5 of −log10(*p* value). Scatter plots representing the correlation of IGF1R expression with levels of miR-15a (**b**) and Fra-2 (**c**) in PDAC samples from the TCGA dataset. The number of analyzed samples (n), the Spearman correlation value (r), and its significance (*p* value) are reported in the graphs. Box plots showing the expression of Fra-2 (**d**) and IGF1R (**e**) in an independent cohort of 38 PDAC samples stratified according to miR-15a levels. miR-15a expression was assessed by qRT-PCR, Fra-2 and IGF1R were evaluated by immunohistochemistry and data represent the percentage of positive cells in each tumor. Unpaired *t* test was used for statistical analyses and asterisks indicate significant differences. **p < 0.01; ***p < 0.001. **f** Histograms showing miR-15a expression in patients from the lower quartile (in black) and from the upper quartile (in green). **g** Representative images of immunohistochemical staining of IGF1R and Fra-2 in PDAC samples stratified according to miR-15a levels, as shown in (**f**) (10x, magnification)

Next, we explored the molecular networks potentially enriched by Fra-2 activity in PDAC with low expression of miR-15a. We performed a pathway enrichment analysis (*via* IPA® software) of the genes targeted by and inversely correlated with miR-15a expression in the PDAC cohort, obtaining 35 pathways possibly repressed by miR-15a (−log10(*p* value) > 1.5) (Supplementary Table 1). Similarly, by performing an IPA® analysis of genes significantly correlated with Fra-2 levels, we obtained 317 molecular pathways potentially activated by Fra-2 (−log10(*p* value) > 1.5) (Supplementary Table 1). To refine our research and identify molecular mediators of PDAC response to nutrient restriction, we set up an in vitro experiment and compared the transcriptomic landscape of AsPC-1 and MIA PaCa-2 PDAC cell lines, cultured either in presence (10% FBS, Normal serum, Ns) or in absence (0% FBS, nutrient deprivation, N-dep) of serum for 72 h. Filtering the microarray data of gene expression (*p* value < 0.01 and Fold-Change > 2.0) (Supplementary Table 2) and performing an IPA® analysis, we identified 126 pathways significantly enriched by N-dep in both PDAC cell lines (−log10(*p* value) > 1.5) (Supplementary Table 1). Finally, we intersected the in silico and in vitro data and identified 5 common pathways, significantly enriched by nutrient restriction, potentially activated by Fra-2 and repressed by miR-15a (Fig. 1a).

We decided to focus on the insulin-like growth factor-1 (IGF1) signaling pathway, given its relevant role in the response to cell stress and metabolic challenges^29–31^ and the established regulation of the IGF1 receptor (IGF1R) by the miR-15 family.^8^ Accordingly, querying the TCGA-PDAC dataset, we confirmed that miR-15a inversely correlated with IGF1R levels (Fig. 1b), whereas Fra-2 and IGF1R expression showed a strong positive correlation (Fig. 1c).

Overall, these results showed that the IGF1 signaling pathway is enriched due to nutrient restriction and that both Fra-2 and IGF1R are highly expressed in miR-15a downmodulated PDAC.

### miR-15a inversely correlates with its targets Fra-2 and IGF1R in human PDAC

To validate these findings, we assessed the expression of our genes of interest in an independent cohort of 38 PDAC patients (Supplementary Tables 3 and 4). In this setting, tumors with low levels of miR-15a by qRT-PCR were characterized by high positivity of Fra-2 (Fig. 1d) and IGF1R (Fig. 1e), as assessed by immunohistochemistry. Accordingly, miR-15a stratified IGF1R and Fra-2 expression in PDAC: in fact, tumors from the miR-15a lower quartile (in black, Fig. 1f) showed diffuse and intense positivity to both IGF1R and Fra-2 (Fig. 1g), whereas tumors from the miR-15a upper quartile (in green, Fig. 1f) showed little/no expression of both Fra-2 and IGF1R (Fig. 1g). In all PDAC samples, the percentage of Fra-2 positive cells was strongly associated with the co-expression of IGF1R (Supplementary Fig. S2a).

As robustly assessed in the TCGA dataset, we also observed in our independent cohort that downmodulation of miR-15a (*p* value 0.08, Supplementary Fig. S2b) and overexpression of Fra-2 (*p* value 0.043, Supplementary Fig. S2c) and IGF1R (*p* value 0.021, Supplementary Fig. S2d) correlated with shorter overall survival of PDAC patients.

### Fra-2 directly regulates IGF1R expression in PDAC response to nutrient deprivation

Our findings suggested that IGF1R modulation could be mediated by Fra-2 and miR-15a, functionally contributing to PDAC response in nutrient deprivation. To explore this hypothesis, we first evaluated the expression of our genes of interest in a panel of PDAC cell lines (Supplementary Fig. S3a, b). In this setting, Fra-2 expression correlated with IGF1R levels (Supplementary Fig. S3a). Consistently with the knowledge that miR-15a is downmodulated in PDAC^18^ (Supplementary Fig. S3b), we found that 3 out of 4 PDAC cell lines harbored a copy number alteration of MIR15A gene (Supplementary Fig. S3c).

**Fig. 2:**
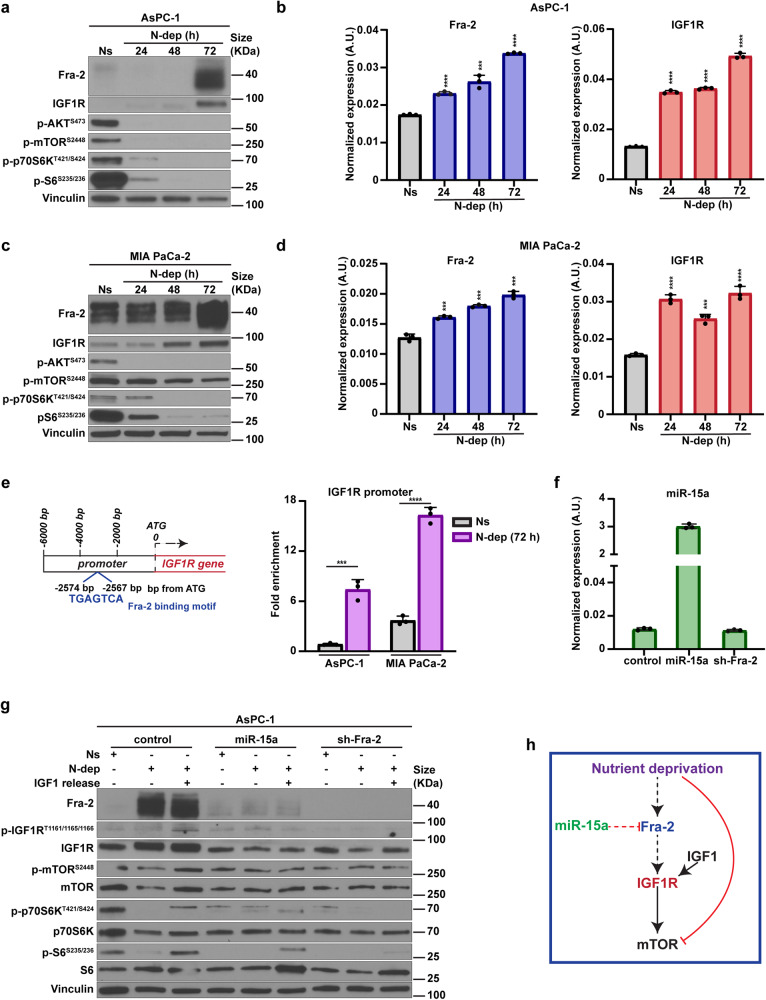
Fra-2 directly regulates IGF1R expression in PDAC response to nutrient deprivation. **a** Western blot analysis of the indicated proteins in AsPC-1 cells, cultured in normal serum (10% FBS, Ns) or in nutrient deprivation (0% FBS, N-dep) and collected at the indicated timepoints (hours, h). Vinculin was used as loading control. **b** Graphs report the normalized expression of Fra-2 (left) and IGF1R (right), evaluated by qRT-PCR analysis in AsPC-1 parental cells cultured as described in (**a**). **c** Western blot analysis of the indicated proteins in MIA PaCa-2 cells, cultured in normal serum (10% FBS, Ns) or in nutrient deprivation (0% FBS, N-dep) and collected at the indicated timepoints (hours, h). Vinculin was used as loading control. **d** Graphs report the normalized expression of Fra-2 (left) and IGF1R (right), evaluated by qRT-PCR analysis in MIA PaCa-2 parental cells cultured as described in (**c**). **e** On the left, schematic representation of Fra-2-binding sequence on IGF1R promoter. On the right, chromatin immunoprecipitation (ChIP) analysis of Fra-2 bound to the IGF1R promoter in AsPC-1 and MIA PaCa-2 cells cultured in normal serum (Ns) or in nutrient deprivation (N-dep) for 72 h (**h**). **f** qRT-PCR analysis of miR-15a normalized expression in control, miR-15a overexpressing and Fra-2 silenced (sh-Fra-2) AsPC-1 cells, as used in the experiment described in (**g**). Data represent the mean (±SD) of three independent experiments. **g** Expression of the indicated proteins and phospho-proteins in cell lysates of control, miR-15a overexpressing and Fra-2 silenced AsPC1 cells, cultured in normal serum (Ns), nutrient deprivation (N-dep) and released with IGF1 (80 ng/ml) for 1 h (IGF1 release), as indicated. Vinculin was used as loading control. **h** Working model for miR-15a modulation of mTOR activity *via* Fra-2/IGF1R in nutrient deprived PDAC cells. Nutrient deprivation-induced cell stress triggers Fra-2 transcriptional activity that, in turn, increases IGF1R expression and eventually restores the phosphorylation of mTOR pathway members. This mechanism is counteracted by miR-15a targeting of IGF1R *via* Fra-2. Dashed lines indicate the novel interaction between miR-15a/Fra-2 and IGF1R, whereas solid lines represent the well-established effects of nutrient deprivation and IGF1R activity on mTOR pathway. In (**b**, **d**, **e**), data represent the mean (±SD) of three independent experiments performed in triplicate. Unpaired *t* test was used for statistical analyses and asterisks indicate significant differences compared to the Ns condition. ***p < 0.001; ****p < 0.0001

Then, we cultured PDAC cells in presence (10% FBS, Ns) or in absence of serum for 72 h (0% FBS, N-dep) and assessed the expression of miR-15a, Fra-2 and IGF1R. Testing AsPC-1, MIA PaCa-2 and Panc 2.3 PDAC cell lines, we observed that miR-15a levels remained substantially stable (Supplementary Fig. 3d–f), whereas Fra-2 and IGF1R expression significantly increased under N-dep in a time-dependent manner. Likely, Fra-2 and IGF1R upregulation were due to changes in the transcription rate since mRNA levels significantly increased under N-dep and the translational block with cycloheximide efficiently impaired protein accumulation in western blot analyses (Fig. 2a–d and Supplementary Fig. S3g–i). Similarly, Insulin Receptor Substrate 2 (IRS2), a direct interactor of IGF1R and member of the IGF1 signaling network,^32^ was also overexpressed in N-dep PDAC cell lines (Supplementary Fig. S3j–l). By contrast, exposure to N-dep decreased the levels of activating phosphorylation of mTOR and its downstream effectors (p70S6K and S6) (Fig. 2a, c and Supplementary Fig. S3g), representing critical drivers of cell proliferation and metabolic homeostasis, usually activated by IGF1R and inhibited by ATP reduction and amino acids unavailability.^33,34^

Aside from stress mediators, different stimuli can drive AP-1 activation.^19^ We thus evaluated Fra-2 and IGF1R expression in PDAC cell lines upon serum components and growth factors. However, our genes of interest did not display a univocal trend in AsPC-1 and MIA PaCa-2 cells (Supplementary Fig. S4). For instance, EGF, IGF1, insulin and glucose administration slightly increased Fra-2 and IGF1R in AsPC-1 (Supplementary Fig. S4a–c), but not in MIA PaCa-2 cell line (Supplementary Fig. S4d–f). This observation further corroborated the specific association between the expression of Fra-2 and activation of the IGF1R signaling pathway in the context of nutrient shortage. To assess whether the IGF1R and IRS2 upregulation directly depended on Fra-2 transcriptional activity, we scanned the upstream regions of IGF1R and IRS2 start codons, identifying an AP-1 binding motif^19^ (Fig. 2e, Supplementary Fig. S4g). Thus, performing chromatin immunoprecipitation assay (ChIP) (Fig. 2e, Supplementary Fig. S4g) and luciferase assay (Supplementary Fig. S5a, b), we observed that Fra-2 directly bound and activated IGF1R and IRS2 promoters and, importantly, Fra-2 activity was significantly increased by N-dep.

Next, to test whether miR-15a could counteract IGF1R expression and activation in N-deprived PDAC cells, we tested the levels of IGF1R in miR-15a overexpressing and Fra-2 silenced cells (Fig. 2f, g). Under basal conditions (Ns), AsPC-1 cells showed low levels of Fra-2 and neither miR-15a overexpression nor Fra-2 silencing affected IGF1R expression compared to the control (Fig. 2g). However, when cells were cultured in N-dep, a significant overexpression of both Fra-2 and IGF1R was observed in control, but not in miR-15a overexpressing and Fra-2 silenced, cells (Fig. 2g, Supplementary Fig. S5c), confirming the inhibitory role of miR-15a on IGF1R expression, *via* Fra-2 targeting.

To explore the significance of IGF1R overexpression in PDAC response to nutrient shortage, we stimulated N-dep AsPC-1 cells with IGF1 and assessed the downstream activation of the IGF1R signaling.^34^ In control cells, IGF-1 stimulation induced an increase of IGF1R phosphorylation (p-IGF1R^T1161/1165/1166^) that, in turn, restored levels of p-mTOR^S2448^, p-p70S6K^T421/S424^ and p-S6^S235/236^ (Fig. 2g, Supplementary Fig. S5c). By contrast, miR-15a overexpressing and Fra-2-silenced AsPC-1 cells (Fig. 2g, Supplementary Fig. S5c) did not overexpress IGF1R in N-dep and, consequently, IGF1 administration did not increase the phosphorylated form of mTOR and its downstream interactors.

Overall, the collected evidence supported that IGF1R overexpression relies on Fra-2 transcriptional activity and sustains mTOR pathway activation in nutrient deprived PDAC cells. This mechanism is counteracted by miR-15a *via* Fra-2 targeting (Fig. 2h).

### miR-15a impairs PDAC cell growth during nutrient restriction, *via* Fra-2 targeting and IGF1R signaling downmodulation

**Fig. 3:**
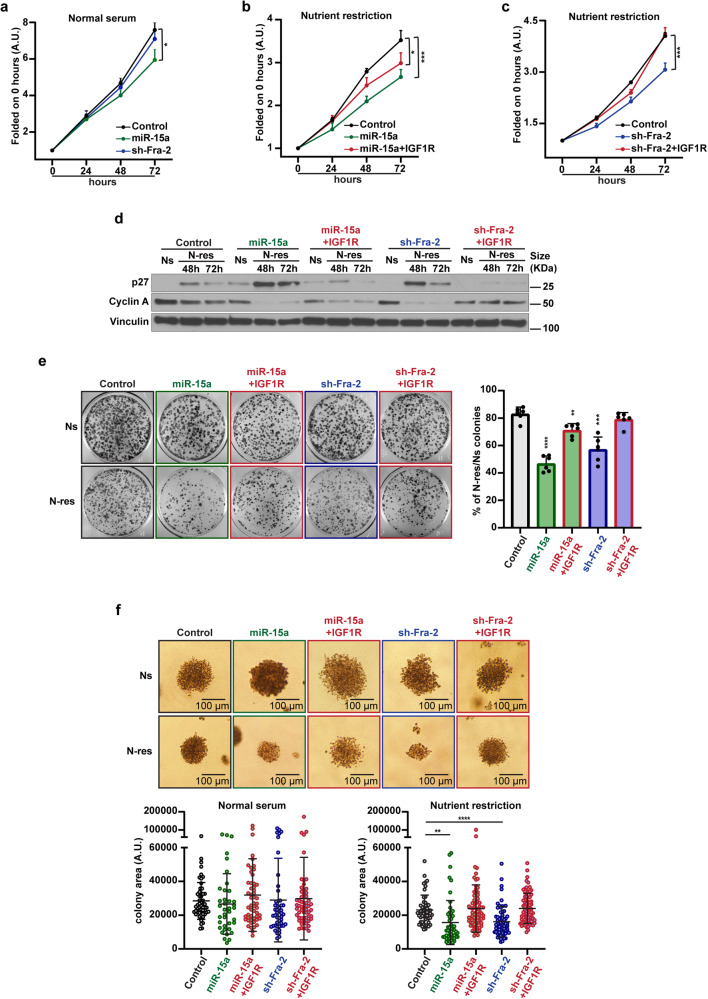
miR-15a impairs PDAC cell growth during nutrient restriction, *via* Fra-2 targeting and IGF1R signaling downmodulation. **a** Graph reports the growth rate of control, miR-15a overexpressing and Fra-2 silenced AsPC-1 cells cultured in normal serum (10% FBS) over a period of 72 h. **b** Graph reports the growth rate of control, miR-15a and miR-15a + IGF1R overexpressing AsPC-1 cells, cultured in nutrient restriction (2.5% FBS) over a period of 72 h. **c** Graph reports the growth rate of control, Fra-2 silenced and Fra-2 silenced+IGF1R overexpressing AsPC-1 cells, cultured in nutrient restriction (2.5% FBS) over a period of 72 h. In (**a**–**c**), data are folded on the 0 h timepoint and represent the mean (±SD) of three independent experiments performed in triplicate. Two-way ANOVA was used to verify the statistical significance and asterisks indicate significant differences compared to controls. *p < 0.05; ***p < 0.001. **d** Western blot analysis evaluating p27 and Cyclin A protein levels in AsPC-1 cells transfected as indicated and cultured in normal serum (10% FBS, Ns) and nutrient restriction (2.5% FBS, N-res). **e** Representative images (left) and graph (right) of colony formation assay of control, miR-15a, miR15a + IGF1R overexpressing, Fra-2 silenced, and Fra-2 silenced+IGF1R overexpressing AsPC-1 cells, cultured in normal serum (10% FBS, Ns) and in nutrient restriction (2.5% FBS, N-res). Data represent the percentage of colonies in N-res folded on the colonies number counted in Ns condition. Data collect the mean (±SD) of three independent experiments performed in duplicate. **f** Representative images (top) and graphs (bottom) of soft agar assay of the indicated cells, as described in (**e**), cultured in normal serum (10% FBS, Ns) and in nutrient restriction (2.5% FBS, N-res). Bottom graphs report the measured areas, and each dot represents a different colony as evaluated in three independent experiments. In (**e**, **f**), unpaired *t* test was used for statistical analyses and asterisks indicate significant differences compared to controls. **p < 0.01; ***p < 0.001; ****p < 0.0001

It is extensively reported in the literature that IGF1R and mTOR signaling play a critical function in cancer, promoting progression, growth and anoikis of neoplastic cells.^32,34^ Due to this, we hypothesized that IGF1R overexpression induced by Fra-2 could drive the progression of PDAC cells grown in nutrient shortage. To explore this possibility, we compared the growing capability of control, miR-15a overexpressing and Fra-2 silenced AsPC-1 cells, cultured in normal serum (10% FBS, Ns) or under nutrient restricted conditions (2.5% FBS, N-res). In Ns, miR-15a overexpression reduced cell proliferation compared to control, whereas Fra-2 silencing resulted ineffective (Fig. 3a). This observation was in line with the established knowledge that miR-15a inhibited several mediators of cell growth in PDAC^17^ and suggested that Fra-2 targeting did not contribute to proliferation arrest, in the presence of nutrients. Conversely, when AsPC-1 cells were challenged with N-res, both miR-15a overexpression and Fra-2 silencing impaired cell proliferation compared to controls (Fig. 3b, c). In this setting, the ectopic expression of IGF1R (Supplementary Fig. S5d) partially and fully rescued the proliferation rate of miR-15a overexpressing and Fra-2 silenced cells, respectively (Fig. 3b, c, red lines).

Accordingly, N-res cultured miR-15a overexpressing and Fra-2 silenced cells showed higher expression of the cell cycle inhibitor p27^kip1^ and lower levels of Cyclin A compared to control cells, both rescued by concomitant ectopic expression of IGF1R (Fig. 3d).

Comparable results were observed when miR-15a overexpressing and Fra-2 silenced cells were evaluated in the ability to form colonies in N-res compared to controls in both anchorage-dependent (Fig. 3e) and -independent (Fig. 3f) assay. These phenotypes were all rescued by re-introducing IGF1R (Supplementary Fig. S5d), confirming that, in a nutrient restricted context, miR-15a overexpression impairs cell growth by targeting IGF1R *via* Fra-2 (Fig. 3e, f).

### In vivo, protein-restricted diet induces Fra-2 and IGF1R overexpression and IGF1 signaling activation in Mir15a^KO^ PDAC

To thrive and progress in a highly hypoxic and nutrient deprived microenvironment, PDAC cells can use amino acids to synthetize glutamine, thus reducing glucose dependency and fueling the cell metabolism.^6,35,36^ Consistently, amino-acids addition strongly reduced Fra-2 overexpression in N-dep cultured AsPC-1 and MIA PaCa-2 cells, while glucose did not exert this effect (Supplementary Fig. S6a).

**Fig. 4:**
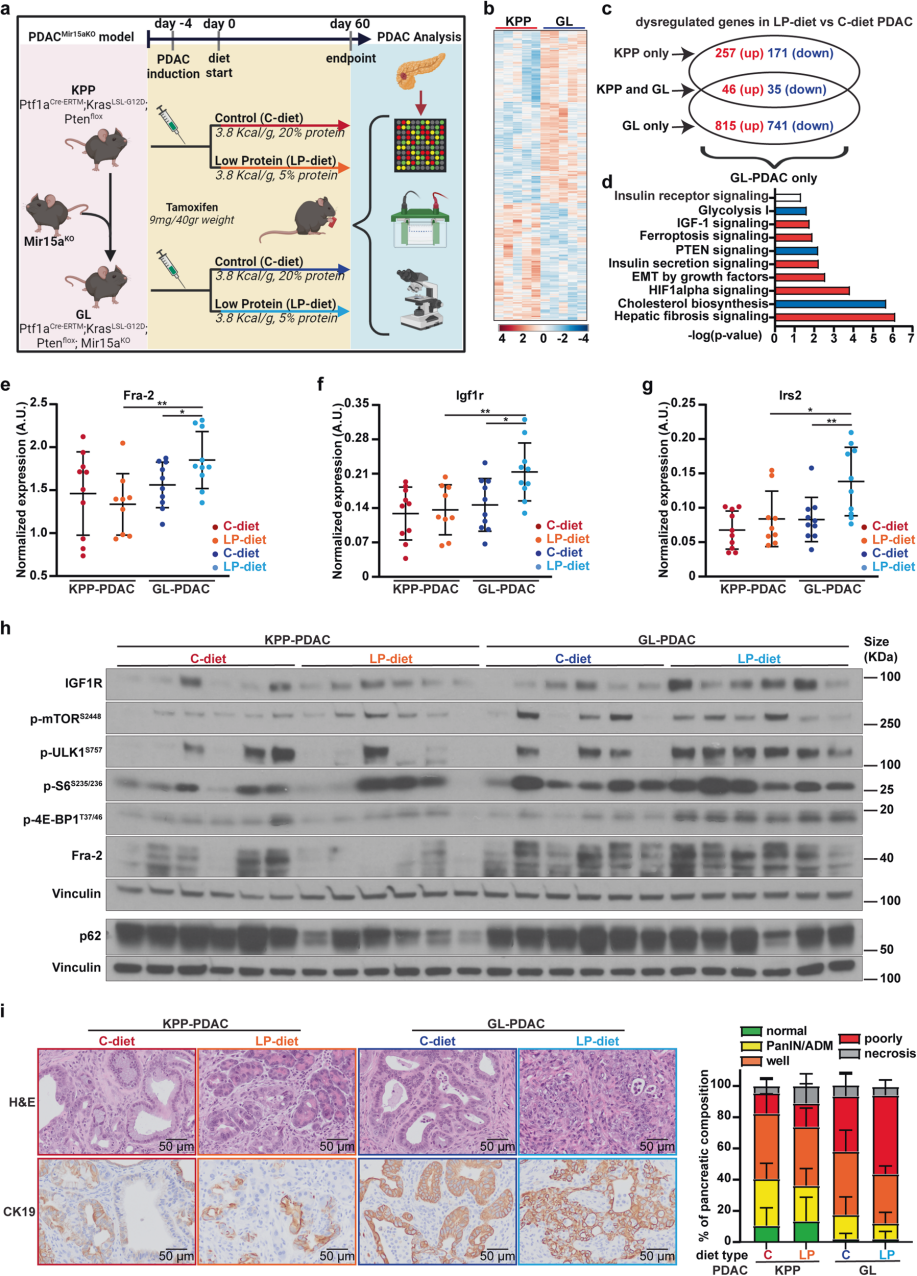
In vivo, protein-restricted diet induces Fra-2 and IGF1R overexpression and IGF1 signaling activation in Mir15a^KO^ PDAC. **a** Schematic representation of the experimental workflow used for the generation and study of an inducible transgenic mouse model of PDAC. The crossbreeding of the Kras^LSL-G12D^, Ptf1a^Cre-ERTM^, Pten^flox^ (named KPP) inducible PDAC model with the Mir15a^KO^ mouse resulted in the KPP/Mir15a^KO^ mouse (named GL). At 6–8 weeks of age, KPP and GL mice were induced by three intraperitoneal injections of Tamoxifen (9 mg/40 gr mouse weight), and at the third injection mice were randomly distributed in two different cohorts, fed with control (3.8 Kcal/g, 20% of proteins, C-diet) or isocaloric, low protein diet (3.8 Kcal/g, 5% of proteins, LP-diet). After 60 days, mice were sacrificed and pancreata were collected and analyzed. Created with BioRender.com. **b** Heat map of deregulated genes in PDAC from KPP and GL mice after 60 days from tumor induction and C-diet feeding (*n* = 5 mice each group). **c** Venn diagram showing the number of dysregulated genes in PDAC from KPP and GL mice fed with LP-diet versus C-diet (*n* = 5 mice each group). Numbers in red represent upregulated genes in response to LP-diet; numbers in blue represent downregulated genes in response to LP-diet compared to C-diet. **d** Histograms showing molecular networks significantly modulated in GL-PDAC fed with LP-diet compared to C-diet. Data were obtained by performing IPA® analyses on the genes specifically altered in GL-PDAC only (*n* = 815 upregulated and *n* = 741 downregulated), obtained from the comparison in (**c**). Blue and red bars represent a profile of negatively and positively enriched pathways, respectively, based on the z-score. White bar represents a neutral profile of enriched pathway, in which the z-score was undetermined. **e-f** Graphs report the normalized expression of Fra-2 (**e**), Igf1r (**f**) and Irs2 (**g**), evaluated by qRT-PCR analysis in PDAC from KPP and GL mice fed with C- and LP-diet, as indicated. Each dot represents a different tumor and unpaired *t* test was used to assess the statistical significance. *p < 0.05; **p < 0.01. **h** Western blot analysis of the indicated proteins in PDAC from KPP and GL mice fed with C- and LP-diet, as indicated. Vinculin was used as loading control. **i** Histology evaluation of KPP- and GL-PDAC fed with C- and LP-diet. On the left, representative images of H&E and CK19 stained sections from PDAC tumors (20x, magnification); on the right, graph represents the percentage of phenotypic components of pancreatic tissues: normal epithelium (non-neoplastic, green), Pancreatic intraepithelial neoplasia and acinar-to-ductal metaplasia (PanIN/ADM, yellow), well-differentiated PDAC (Well, orange), poorly differentiated PDAC (Poorly, red), or necrosis (gray). (*n* = 10 KPP C-diet; 9 KPP LP-diet; 10 GL C-diet; 10 GL LP-diet)

To validate these results in vivo, we established a reduced dietary protein intake in mice and evaluated whether this condition elicited a miR15a/Fra-2 dependent IGF1R signaling activation in PDAC, as observed in vitro. We crossbred the Kras^LSL-G12D^, Ptf1a^Cre-ERTM^, Pten^flox^ (hereafter named KPP) inducible PDAC mouse model^37^ with the Mir15a^KO^ mouse,^9,10^ obtaining the KPP/Mir15a^KO^ mice (hereafter named GL) (Fig. 4a). Once tumors were induced by tamoxifen, KPP and GL mice were randomly distributed in two different cohorts, fed either with control diet (20% of proteins, C-diet), or with isocaloric, low protein diet (5% of proteins, LP-diet) (Fig. 4a). Since KPP mice develop an aggressive disease with an average survival of 80 days from the induction,^37^ mice were sacrificed and PDAC analyzed after 60 days.

LP-diet induced a remarkable wasting compared to C-diet, in both genotypes (Supplementary Fig. S6b). At the time of necroscopy, the tumor marker Krt19 was used to verify comparable levels of tumor burden among the collected pancreata samples (Supplementary Fig. 6c). Analysis of the PDAC transcriptional landscape (*n* = 5 mice/group) showed significant differences in gene expression between KPP and GL in the C-diet group (Fig. 4b, Supplementary Table 5) (*p* value < 0.05 and |Fold-Change | > 1.5). Interestingly, neither Fra-2 nor Igf1r were present among the dysregulated genes in GL- compared to KPP-PDAC in C-diet, suggesting that, in absence of stress stimuli, Fra-2 was inactive and did not induce Igf1r transcription regardless Mir15a status. Then, we analyzed the impact of LP-diet on PDAC transcriptional profiles. Intriguingly, GL-PDAC (*n* = 1637 genes) showed a higher number of dysregulated genes in response to LP-diet compared to KPP-PDAC (*n* = 509) (Fig. 4c), whereas miR-15a was stably expressed in KPP-PDAC regardless of diet (Supplementary Fig. S6d). To investigate the specific contribution of Mir15a^KO^ in the PDAC response to LP-diet, we performed the IPA® analysis on those genes specifically altered in GL-PDAC only (815 upregulated and 741 downregulated genes). LP-diet induced a profile of pathway enrichment highly consistent with our findings in PDAC cells cultured in N-dep (e.g., IGF1 signaling, Ferroptosis signaling and EMT) (compare Fig. 1a with Fig. 4d), supporting that LP-diet may represent a valid approach to translate in vivo the phenotypes elicited in vitro by nutrient deprivation and confirming that loss of miR15a promoted an adaptive mechanism in PDAC, relying on growth factor signaling and metabolic switch (Fig. 4d).

In line with our previous findings, GL-PDAC significantly overexpressed Fra-2 (Fig. 4e) and, with regards to the IGF1 signaling pathway, Igf1r (Fig. 4f), Irs2 (Fig. 4g) and Mapk8 (Supplementary Fig. S6e) in response to LP-diet.

By western blot analysis, we confirmed IGF1R overexpression in response to LP-diet and, although mTOR is generally inhibited by amino acid scarcity,^33^ we also found high levels of phosphorylation of mTOR (S2448) and its downstream effectors ULK1 (S757), S6 (S235/236) and 4E-BP1 (T37/46), more prominently in GL-PDAC than in KPP-PDAC (Fig. 4h, Supplementary Fig. S6f).

Aiming to assess the impact of protein restriction on tumor progression, we explored the morphology of pancreata among the different cohorts of mice. KPP-PDAC histology showed that a large component of normal epithelium (CK19-) and pancreatic intraepithelial neoplasia (PanIN) (CK19+) was still present (≈40% of pancreatic tissue) (Fig. 4i), whereas well-differentiated was more represented than poorly differentiated PDAC (CK19+) (≈40% and ≈13%, respectively). Notably, pancreatic phenotype was not affected by diet (Supplementary Fig. S6g, h).

On the other side, GL-PDAC analysis displayed an increased transformation of pancreatic tissue with a minimal residual component of normal tissue and PanIN (≈14%), already in C-diet context (Fig. 4i, Supplementary Fig. S6g). LP-diet promoted even further the tumor aggressiveness of this Mir15a^KO^ background, showing an increased neoplastic transformation and a higher percentage of poorly differentiated PDAC (Fig. 4i, Supplementary Fig. S6g, h). Consistent with the proliferative phenotypes observed in vitro (Fig. 3), LP-diet significantly inhibited the local aggressiveness of KPP-PDAC but not of GL-PDAC (Supplementary Fig. S6i).

Overall, data collected in vivo demonstrated that Mir15a^KO^ favors tumor progression in nutrient restriction, pointing to a critical role of IGF1 signaling in the adaptive mechanisms of PDAC. These findings may be in contrast with the established knowledge that protein-restricted diet reduces circulating levels of IGF1.^38^ In line with the current literature,^38^ we observed that LP-diet effectively reduced levels of circulating free IGF1 in both KPP and GL mice (Supplementary Fig. S7a). However, when PDAC were analyzed, no significant difference was found in IGF1 levels both at mRNA and protein levels (Supplementary Fig. S7b, c). Collectively, these results suggested that, at least in the tumor microenvironment, IGF1 bioavailability was not compromised by protein restricted diet and could still activate IGF1R overexpressing Mir15a^KO^ PDAC.

### miR-15a modulates autophagic flux *via* Fra-2 and IGF1R targeting in nutrient deprived PDAC cells

Our results showed that, in response to nutrient deprivation, Fra-2 and IGF1R were overexpressed in miR-15a downmodulated PDAC, eventually activating mTOR and its downstream effectors. Consequently, we explored whether this axis could impact on the autophagy, a stress response mechanism frequently altered in PDAC^7^ and negatively regulated by mTOR phosphorylation of ULK1 on serine residue 757.^39^ Intriguingly, LP-diet induced a strong increase of phosphorylated ULK1^S757^ and decrease of p62 levels in GL-PDAC but not in KPP-PDAC (Fig. 4h, Supplementary Fig. S6f), suggesting an impairment of the autophagic flux.

**Fig. 5:**
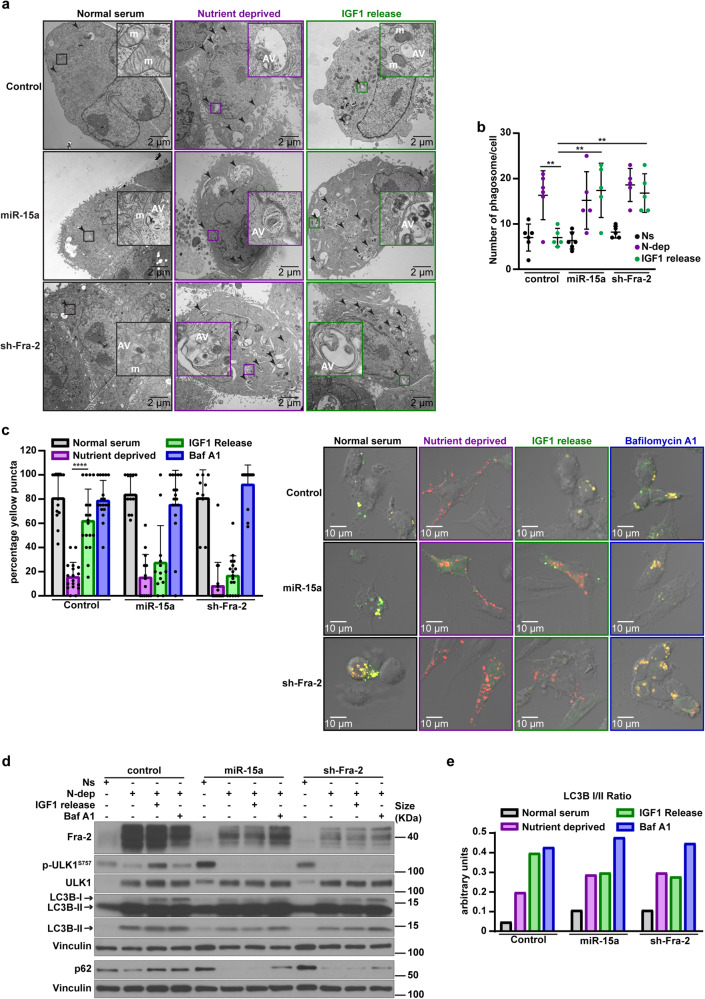
miR-15a modulates autophagic flux *via* Fra-2 and IGF1R targeting in nutrient deprived PDAC cells. **a** Transmission electron microscopy (TEM) ultrastructure analyses of control, miR-15a overexpressing and Fra-2 silenced AsPC-1, cultured in normal serum (10% FBS), nutrient deprivation (0% FBS) and released with IGF1 (80 ng/ml) for 2 h. Arrowheads indicate autophagic vacuoles and insets show an enlargement to highlight the ultrastructure of autophagic vacuoles. Indicated intracellular organelles are mitochondria (m) and autophagic vacuoles (AV). **b** Graph reports the number of autophagic vacuoles per cell, as assessed by TEM. Each dot represents a different evaluated cell and unpaired *t* test was used to verify the statistical significance. **p < 0.01. **c** Confocal microscopy analyses of control, miR-15a overexpressing and Fra-2 silenced AsPC-1 cells transfected with ptfLC3 construct. Cells were cultured in normal serum, nutrient deprivation and released or not with IGF1 (80 ng/ml, 2 h) or Bafilomycin A1 (0.2 μM for 1 h), as indicated. On the left, the graph reports the percentage of yellow LC3 puncta counted per cell (number of cells/experimental condition = 7–12). Unpaired *t* test was used to verify the statistical significance. ****p < 0.0001. On the right, typical confocal images of the described experiment. **d** Western blot analysis evaluating the expression of the indicated autophagy markers in control, miR-15a overexpressing and Fra-2 silenced AsPC-1 cells, cultured in normal serum (10% FBS, Ns), in nutrient deprivation (0% FBS, N-dep), and released with IGF1 (80 ng/ml, 2 h) or Bafilomycin A1 (0.2 μM for 1 h), as indicated. Vinculin was used as loading control. **e** Graph reporting the normalized LC3B I/II ratio in cell lysates, as evaluated in (**d**)

To measure the relevance of this hypothesis at the morphological level, we used transmission electron microscopy (TEM) and observed the intracellular vesicle compartment of the cell under different nutrient contexts. When cultured under N-dep condition, control, miR-15a overexpressing and Fra-2 silenced cells presented an increasing number of vesicles with an ultrastructure compatible with autophagic vacuoles compared to their counterparts grown in Ns (Fig. 5a, b). IGF1 administration significantly reduced autophagosomes in control, but not in miR-15a overexpressing and Fra-2 silenced AsPC-1 cells (Fig. 5a, b and Supplementary Fig. S8a).

An increase in autophagosomes may reflect either an active biosynthesis or an accumulation due to an autophagy inhibition.^40^ To elucidate these possibilities, we explored the autophagic flux in our model, using the inhibitor Bafilomycin A1. To this aim, we exploited a fluorescence assay using an LC3 construct tagged to mRFP-GFP tandem fluorescent proteins (ptfLC3),^41^ allowing the monitoring of phagosomes maturation: in an acidic environment, EGFP protein is quenched, whereas mRFP remains stable, allowing to distinguish if autophagic vesicles are (red fluorescence) or are not (red plus green = yellow fluorescence) fused with lysosomes.^41^

We observed that nutrient deprivation greatly decreased the number of autophagosomes fused with lysosomes, in all cell types, as expected. IGF1 release failed in increasing the percentage of yellow LC3 puncta in miR-15a overexpressing and Fra-2 silenced cells compared to the controls (Fig. 5c). Conversely, Bafilomycin A1 treatment restored the number of yellow puncta (Fig. 5c), suggesting that autophagic flux was intact in all conditions. Western blot analyses showed that IGF1 release increased p62 levels, the inhibitory phosphorylation of ULK^S757^ and attenuated the conversion of LC3B-I to the autophagosome-associated LC3B-II form in N-dep cultured control cells but not in miR-15a overexpressing and Fra-2 silenced cells (Fig. 5d, e and Supplementary Fig. S8b). By contrast, the re-introduction of IGF1R in miR-15a overexpressing and Fra-2 silenced cells rescued levels of phospho-ULK^S757^ and LC3B-I when IGF1 was added (Supplementary Fig. S8c, d).

Collectively, these experiments indicated that IGF1R upregulation contributes to the autophagy modulation in response to N-dep, and miR-15a impairs this mechanism *via* Fra-2 targeting.

### Fra-2 dictates sensitivity to IGF1R inhibition in nutrient deprived PDAC

Next, we explored whether the pharmacological inhibition of IGF1R could represent a valuable strategy to counteract PDAC progression. To exclude any effect caused by miR-15a targeting of unrelated genes, we generated a more controlled model of CRISPR-Cas9 Fra-2 knockout (Fra-2^KO^) AsPC-1 cells and tested their response to the small-molecule Linsitinib, a selective IGF1R inhibitor.

In dose-response curves performed in presence of serum (Ns), wild-type and Fra-2^KO^ AsPC-1 cells displayed the same sensitivity to Linsitinib (Supplementary Fig. S9a). On the contrary, when cells were challenged with N-dep and then treated, only wild-type cells overexpressed IGF1R, becoming more sensitive to Linsitinib compared to Fra-2^KO^ cells (Supplementary Fig. S9b, c). Then, IGF1R re-expression in Fra-2^KO^ cells restored Linsitinib sensitivity (Supplementary Fig. S9b, c). These results demonstrated that, at least in vitro, N-dep induces a status more prone to IGF1R inhibition in Fra-2 proficient PDAC.

To translate these observations in vivo, we exploited a xenograft mouse model of PDAC, injecting wild-type and Fra-2^KO^ AsPC-1 cells into the flank of nude mice (Supplementary Fig. S10a). Once tumors became palpable, we divided them in two groups fed with C-diet and LP-diet; then, after 3 days, mice were treated with Linsitinib (50 mg/kg, daily) or untreated for 3 weeks. At the endpoint, mice were sacrificed, and tumors were collected for further analyses.

**Fig. 6:**
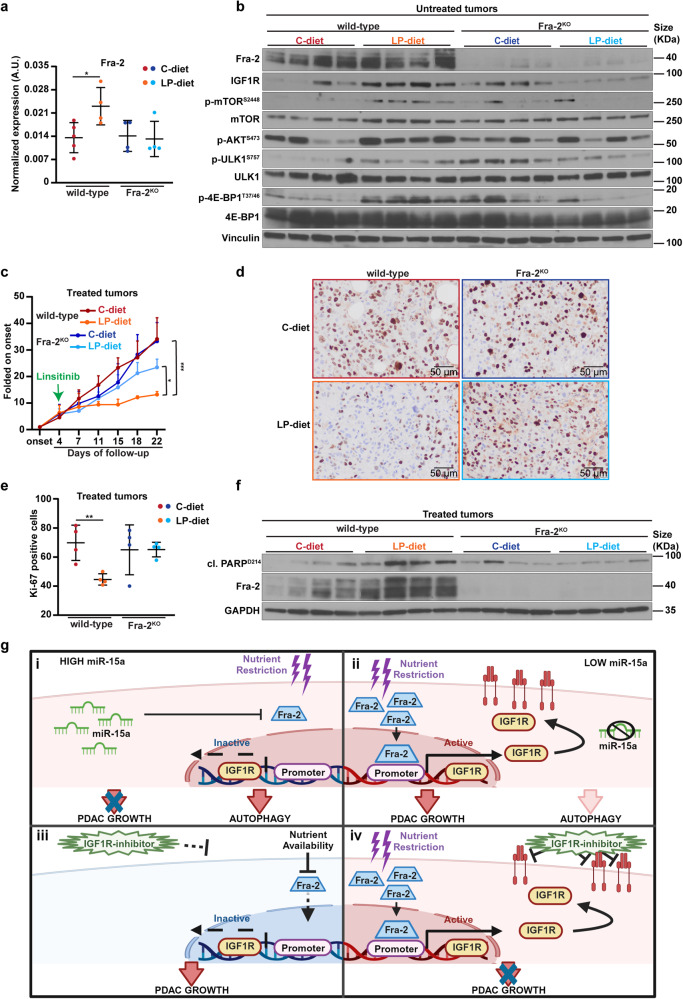
Fra-2 dictates sensitivity to IGF1R inhibition in nutrient deprived PDAC. **a** Graph reports the normalized expression of Fra-2, evaluated by qRT-PCR analysis in tumors explanted from untreated mice cohort (vehicle) as summarized in Supplementary Fig. S10a. Nude mice were injected in the flank with either wild-type or Fra-2^KO^ AsPC-1 cells. Once tumor onset was established, mice were randomly subdivided in two different cohorts, fed with control diet (C-diet) or isocaloric, low protein diet (LP-diet) and treated daily with vehicle. Each dot represents a different tumor and unpaired *t* test was used to assess the statistical significance. *p < 0.05. **b** Western blot analysis evaluating the expression of the indicated proteins in tumors explanted from the untreated (vehicle) cohort of mice described in (**a**). Vinculin was used as loading control. **c** Graph reporting the growth rate of tumors (*n* = 4 tumor/group) from the cohort of mice treated with Linsitinib. Nude mice were injected in the flank with either wild-type or Fra-2^KO^ AsPC-1 cells. Once tumor onset was established, mice were randomly subdivided in two different cohorts, fed with control diet (C-diet) or isocaloric, low protein diet (LP-diet) and treated daily with Linsitinib for 3 weeks. Green arrow indicates the starting point of Linsitinib administration. Data are represented as the mean (±SD) of 4 tumor/group folded on their respective volume at the onset and two-way ANOVA was used to verify the statistical significance. *p < 0.05; ***p < 0.001. **d** Typical images of Ki-67 expression evaluated by immunohistochemistry (IHC) in tumors explanted from mice treated with Linsitinib as described in (**c**) (20x, magnification). **e** Graph reports the percentage of Ki67-positive cells in tumors represented in (**d**). Data are expressed as mean (±SD) of Ki-67 percentage counted in five randomly selected fields per tumor. Each dot represents a different tumor. Unpaired *t* test was used to verify the statistical significance. **p < 0.01. **f** Western blot analysis evaluating the expression of the indicated proteins in tumors explanted from the cohort of mice treated with Linsitinib as described in (**c**). GAPDH was used as loading control. **g** Working model for miR-15a/Fra-2 modulation of IGF1R signaling and sensitivity to IGF1R-inhibitor in PDAC cells exposed to different conditions of nutrient availability. i High levels of miR-15a inhibit IGF1R expression *via* Fra-2 targeting, impinging on PDAC growth during nutrient restriction. ii In miR-15a downmodulated PDAC, nutrient restriction triggers expression and transcriptional activity of Fra-2 that, in turn, leads to PDAC growth by upregulating IGF1R. iii In presence of nutrient availability, does not activate IGF1R transcription and administration of IGF1R-inhibitor results ineffective. iv Fra-2 induces IGF1R overexpression in response to nutrient restriction, sensitizing PDAC cells to IGF1R inhibition. Created with Biorender.com

In the untreated mice, LP-diet reduced the tumor growth compared to the C-diet. However, this impairment was more evident in Fra-2^KO^ than in wild-type tumors (Supplementary Fig. S10b). Moreover, LP-diet induced the overexpression of both Fra-2 and IGF1R (Fig. 6a, Supplementary Fig. S10c) in wild-type but not in Fra-2^KO^ tumors, whereas miR-15a levels remained stable in both genotypes (Supplementary Fig. S10d). Western blot analyses showed that LP-diet significantly increased phosphorylation of mTOR, 4E-BP1, ULK1 and LC3B-I/II ratio in wild-type but not in Fra-2^KO^ tumors, suggesting a strong activation of the mTOR pathway and a modulation of the autophagic flux (Fig. 6b, Supplementary Figs. S10e and S11a).

When Linsitinib-treated tumors were analyzed, we observed that wild-type and Fra-2^KO^ tumors displayed a similar response to the inhibitor in mice fed with C-diet, consistent with their IGF1R expression levels (Fig. 6c and Supplementary Fig. S11b). On the contrary, LP-diet was found to significantly increase the drug sensitivity of wild-type compared to Fra-2^KO^ tumors (Fig. 6c). Moreover, combination of LP-diet and Linsitinib administration significantly reduced cell proliferation of wild-type tumors (Fig. 6d, e) and induced cleavage of PARP, resulting in or due to an increased apoptotic activity (Fig. 6f, Supplementary Fig. S11c).

Overall, our in vivo results confirmed that Fra-2 controls IGF1R expression in response to nutrient deprivation in PDAC. Importantly, this mechanism could be effectively counteracted by administration of IGF1R-inhibitors.

## Discussion

The ability to thrive and progress in unfavorable conditions represents a remarkable feature of PDAC.^4^ Many observations in literature have reported that miR-15a is frequently dysregulated, leading to uncontrolled cell growth, apoptosis inhibition and drug resistance.^18^ However, miR-15a role in PDAC adaptation to nutrient shortage is still largely unexplored. To fill this gap, we investigated the miR-15a regulation of Fra-2, a transcription factor critically activated by stress stimuli and whose consensus sequence is one of the most accessible motif in the chromatin of PDAC compared to normal epithelium.^25^ We found that the IGF1 signaling was enriched in nutrient deprived PDAC cells (Fig. 1a), whereas miR-15a expression inversely correlated with Fra-2 and IGF1R levels in PDAC patients (Fig. 1b, d–g and Supplementary Fig. S1c). In this context, miR-15a downmodulation unleashed Fra-2 expression and transcriptional activity, eventually leading to IGF1R upregulation.

It is interesting to note that our in silico analyses in nutrient deprived cells showed an enrichment in other pathways possibly controlled by miR-15a and Fra-2 in PDAC (Fig. 1a). In particular, ferroptosis signaling, a form of cell death resulting from elevated oxidative stress and finely tuned by PDAC cells,^42^ was also enriched in our genetic mouse model of Mir15a^KO^ PDAC under diet restriction (Fig. 4c, d), further supporting the fact that miR15a loss shapes the adaptive phenotype of PDAC by acting at different levels.

Focusing on the IGF1 signaling, we found that Fra-2 transcriptional activity also involved IRS2, a direct IGF1R interactor (Supplementary Figs. S3j–l, S4g and S5b). Due to this involvement, protein-restricted diet significantly increased Irs2 expression in Mir15a^KO^ murine tumors (Fig. 4g). Collectively, these results support a complex portrait in which Fra-2 orchestrates the stress response in miR-15a downmodulated PDAC, by regulating different key pathways and several effectors.

To explore our findings in vivo, we exploited a genetic model of PDAC using Mir15a^KO^ mice,^9,10^ which develop a spontaneous but indolent form of lymphocytic malignancy only in the elderly (approximately 1.5 years of age). To circumvent this limit and avoid any phenotypic overlap, we crossbred Mir15a^KO^ with KPP mice,^37^ that generate rapidly growing, aggressive PDAC, with an average survival of 80 days. At this young age, Mir15a^KO^ mice do not show any lymphocytic lineage alteration.^9,10^ Whether and how the Mir15a^KO^ tumor microenvironment may contribute to the observed phenotype will certainly be the object of future investigations.

In response to nutrient shortage, we found that IGF1R overexpression activated the mTOR pathway, attenuating the autophagic flux (Figs. 2g, 5d and Supplementary Fig. S8c, d). This finding is largely supported by literature since, in in vitro models, miR-15a expression promotes autophagy by targeting Rictor, a component of the mTOR complex,^43^ whereas AP-1 family members inhibit autophagy of starved cells^44^ and IGF1R activity strongly correlates with reduced autophagy in breast cancer patients.^45^ On the other hand, the fact that PDAC cells reduced the autophagic flux when exposed to nutrient shortage may appear controversial, considering that autophagy represents an alternative source of nutrients for starved PDAC cells, fueling tumor growth.^7^ However, it is established that autophagy could also play an onco-suppressive role in PDAC, since autophagy restriction accelerates the early stages of transformation and tumor progression.^46^

Intriguingly, in our genetic mouse model, protein-restricted diet impaired the neoplastic transformation of pancreatic epithelium in Mir15a^WT^ but not in Mir15a^KO^ pancreata (Fig. 4i and Supplementary Fig. S6g, h), in which Igf1r was overexpressed and the pro-autophagic ULK1 protein inhibited (Fig. 4f, h). Moreover, it is reported that intensely activated autophagy could promote cell death in PDAC^47^; thus, we could speculate that IGF1R overexpression in nutrient deprivation could also exert a protective function in neoplastic cells, preventing an excess of autophagy. Further investigations are needed, but it is conceivable that the described mechanism could play different roles in a context-dependent manner, according to the stage of PDAC progression.

Despite that combination of IGF1R inhibitor and chemotherapy has substantially failed as first line treatment in PDAC patients with metastatic disease,^48^ recent evidence suggests that IGF1R inhibition leads to autophagy dependence, increasing the sensitivity to autophagy inhibitors in preclinical model of PDAC.^49^ In fact, it is known that IGF1R contributes to the insurgence of resistance to multiple drugs in cancer.^32^

Here, we reported that IGF1R overexpression also represents a distinctive trait of the stress-tolerant phenotype mediated by Fra-2 in miR-15a downmodulated PDAC. This mechanism could be also elicited in vivo, since diet restriction significantly induced IGF1 signaling activation, only in Fra-2 proficient tumors (Fig. 6b and Supplementary Fig. S10c).

Thus, we proposed diet restriction as a valid method to sensitize PDAC to the administration of IGF1R inhibitors (Fig. 6g), paving the way for future studies to assess whether this regimen would be tolerated in patients and whether miR-15a downmodulation could be used as a prognostic biomarker to identify tumors more prone to this therapeutic approach.

## Materials and methods

### Patient samples and study approval

Specimens from 38 primary PDAC (Supplementary Tables 3 and 4) were collected from patients who underwent surgery without receiving neoadjuvant therapy at Hepato-pancreato-biliary Surgery and Liver Transplantation Unit, University of Modena and Reggio Emilia, Modena, Italy. The study was approved by the Ethical Committee “Comitato Etico dell’Area Vasta Emilia Nord” of the University of Modena and Reggio Emilia, Italy (Prot. AOU #0019500/21), and by the Internal Review Board of The Ohio State University, USA (IRB #2018C0131). The experiments conformed to the principles set out in the WMA Declaration of Helsinki and the Department of Health and Human Services Belmont Report.

### Animal study approval

All animal experiments were performed in compliance with institutional guidelines and approved by the Institutional Animal Care and Use Committee (IACUC) and the University Laboratory Animal Research at The Ohio State University.

### Cell biology experiments

#### Cell culture, transfection, and reagents

Pancreatic cancer cell lines AsPC-1 (CRL-1682), MIA PaCa-2 (CRL-1420), Panc 2.3 (Panc 02.03 CRL-2553), PSN-1 (CRL-3211), and kidney embryonic cells HEK293 (293 [HEK-293] CRL-1573) were purchased from the American Type Culture Collection (ATCC) and cultured in RPMI1640 or DMEM (only for MIA PaCa-2 cell line) medium (Sigma, USA) supplemented with 10% fetal bovine serum (FBS, Sigma, USA) and 1% Streptomycin/ampicillin solution (Sigma, USA).

For transient transfection, FOSL2 siRNA (Product no. EHU072911), and control siRNA (scramble oligonucleotides; Product no. SIC001) were purchased from Sigma-Aldrich, USA (Mission esiRNA). Pre-miR-15a precursor (Assay ID PM10235), pre-miR Precursor Negative Control #2 (Cat# AM17111), were obtained from ThermoFisher Scientific, USA. IGF1R_pLX307 was a gift from William Hahn and Sefi Rosenbluh (plasmid # 98344, Addgene). ptfLC3 was a gift from Tamotsu Yoshimori (plasmid # 21074, Addgene).^41^ Lipofectamine 2000 (ThermoFisher Scientific, USA) transfection system was used following the manufacturer’s instructions for miR-15a and IGF1R overexpression, ptfLC3 transfection and Fra-2 silencing in AsPC-1 cell line.

Detailed list of reagents can be found in Supplementary Data.

#### Generation of stable Fra-2^KO^ clones

To generate Fra-2 KO clones, CRISPR/Cas-9 technology was used. Stable AsPC-1 Fra-2^KO^ pool was obtained by transduction with lentiviral particles (LV01 U6-gRNA:ef1a-puro-2A-Cas9-2A-tGFP, by Sigma-Aldrich).

Experimental details can be found in Supplementary Data.

#### Dual-Luciferase reporter assay

The predicted miR-15a-binding site of FOSL2/Fra-2 3’UTR were amplified by PCR using specific primers. PCR products were digested with XhoI and NotI (New England Biolabs, USA) and cloned downstream of Renilla luciferase gene of psiCHECK2 vector (Promega, USA). The mutant of FOSL2/Fra-2 3’UTR were generated using QuikChange II XL Site-Directed Mutagenesis Kit (Agilent, USA), according to the manufacturer’s protocol. HEK293 cells were co-transfected with 1 mg of psiCHECK2 constructs and 100 nM of pre-miR-15a precursor (Assay ID PM10235, ThermoFisher Scientific, USA) in 12-well plate using Lipofectamine 2000 (ThermoFisher Scientific, USA) according to manufacturer’s recommendations. After 24 h, Dual-Luciferase Assay (Promega, USA) was performed to measure the reporter activity. Primers are listed in Supplementary Table 6.

#### MTS cell proliferation, colony, soft agar assay, autophagy assay and dose-response curves

In vitro experiments were performed essentially as previously reported.^11,24^ Experimental details can be found in Supplementary Data.

#### Transmission electron microscopy and confocal microscopy

For transmission electron microscopy (TEM) analysis, control, miR-15a overexpressing and Fra-2 silenced AsPC-1 cells were seeded in Lab-Tek^TM^ chamber slide system (Cat. #177429, ThermoFisher Scientific, USA) (15,0000 cells/well). The day after, medium was replaced, and cells were cultured in Normal serum (10% FBS) or in Nutrient deprivation (0% FBS). After 72 h, cells in nutrient deprivation were released or not with IGF1 (80 ng/ml, 2 h). Then, samples were immediately fixed with 2.5% glutaraldehyde in 0.1 M phosphate buffer for 30 min at room temperature. Samples were postfixed with 1% osmium tetroxide and then *en bloc* stained with 1% aqueous uranyl acetate, dehydrated in a graded series of ethanol, and embedded in Eponate 12 epoxy resin (Ted Pella Inc., CA). Ultrathin sections were cut with a Leica EM UC6 ultramicrotome (Leica microsystems Inc., USA). Images were acquired with an FEI Technai G2 Spirit BioTwin transmission electron microscope (Thermo Fisher Scientific, USA) operating at 80 kV, and a Macrofire digital camera (Optronics, Inc., USA) and AMT image capture software (Advanced Microscopy Techniques, USA). At least 5 cells per condition were evaluated.

For confocal microscopy, control, miR-15a overexpressing and Fra-2 silenced AsPC-1 cells were transfected with ptfLC3 construct^41^ and plated on coverslips, cultured in Normal serum (10% FBS) or in nutrient deprivation (0%FBS). After 72 h, nutrient deprived cells were released or not with IGF1 (80 ng/ml, 2 h) or Bafilomycin A1 (0.2 μM, 1 h) and then immediately fixed in PBS 4% PFA for 10’. ptfLC3 expression was analyzed using Olympus FV1000 filter Confocal System microscope essentially as described.^41^ 7–12 cells per condition were evaluated.

### Molecular biology experiments

#### Western blot analysis

Protein lysates and Western blot were performed essentially as previously described.^24^ Experimental details and the list of antibodies can be found in Supplementary Data.

#### Chromatin immunoprecipitation assay

Chromatin preparation, immunoprecipitation and analyses were performed essentially as previously described.^50^ Experimental details and the list of antibodies can be found in Supplementary Data.

#### RNA isolation and quantitative Real-time PCR

Total RNA was isolated, retro-transcribed and analyzed essentially as previously described.^24^ Experimental details and the list of probes can be found in Supplementary Data.

#### Copy number variation

Genomic DNA was isolated from PDAC cell lines using DNeasy Blood & Tissue Kit (Cat. #69504, Qiagen, USA) according to manufacturer’s recommendations. Copy number variation assay was performed on 10 ng of genomic DNA. Quantitative real-time polymerase chain reaction (PCR) TaqMan Copy Number Assay was performed using FAM-dye-labeled custom probe targeting miR-15a (Applied Biosystems, USA). TaqMan CNV reactions were performed in triplicate using as reference VIC-dye labeled TERT assay.

#### In vivo experiments (GL generation and nude mice)

KPP mice^37^ (Kras^LSL-G12D^, Ptf1a^Cre-ERTM^, Pten^flox^) were purchased from The Jackson Laboratory, USA. GL model was generated crossbreeding KPP and Mir15a^KO^ mice.^10^ To evaluate the tumor growth and onset of PDAC cells, primary tumors were established by subcutaneous injection of 1.5 × 10^6^ wild-type (17 mice) and Fra-2^KO^ (16 mice) AsPC-1 cells into the flanks of female athymic nude mice (The Jackson Laboratory, USA).

Experimental details and the list of antibodies can be found in Supplementary Data.

#### Histological analysis and immunohistochemistry

At the endpoint, mouse xenograft samples and abdominal organs from inducible PDAC mouse models were fixed in neutral buffered formalin 10% for 72 h and processed for standard paraffin embedding. Histological sections (5 μm thick) were made from the paraffin blocks, deparaffinated with xylene, and stained with hematoxylin and eosin (H&E), according to standard procedures. Routine deparaffinization of PDAC human tissues and murine samples mounted on positive charge slides was carried out according to standard procedures, followed by rehydration through serial ethanol treatments. Slides were immersed in citrate buffer [0.01 M sodium citrate (pH 6.0)] and heated in a microwave oven at 600 W (three times for 5 min each) to enhance antigen retrieval. Endogenous peroxidase was blocked with 0.3% hydrogen peroxide in methanol for 30 min. Sections were immunostained with Fra-2 (Cat. #19967, Cell Signaling Technology, USA), IGF1R (Cat. #3027, Cell Signaling Technology, USA), Ki67 (Cat. #NBP2-54791, Novus Bio, USA) and CK-19 (Cat. #ab52625, Abcam, USA) antibodies according to manufacturer’s protocol and standardized procedures.

Images were collected with Olympus microscope BX41. The histological evaluation of Fra-2 and IGF1R in PDAC human samples and Ki-67 expression in xenograft samples were assessed as the percentage of moderately/highly positive neoplastic cells counted in 5 random fields. Percentage of pancreatic phenotypes in KPP/GL mice was assessed substantially as described.^5^ All histological and immunohistochemical analyses were performed by two pathologists independently and a consensus was reached in the doubtful cases.

#### Affymetrix microarray

AsPC-1 cell lines were cultured in normal serum or in nutrient deprivation for 72 h, RNA was collected and isolated with TRIzol and RNA clean-up and concentration kit (Cat. #23600, Norgen Biotek Corp, Canada). Pancreatic cancer tissues were collected from GL and KPP mice fed with control or low protein diet and immediately homogenized with grinders in TRIzol^TM^ Reagent (Cat. #15596026, Invitrogen, USA). RNA was collected and isolated with TRIzol and RNA clean-up and concentration kit (Cat. #23600, Norgen Biotek Corp, Canada). Total RNA was treated with RNase-free DNase I Kit (Cat. #25710, Norgen Biotek Corp, Canada), in order to avoid DNA contamination. Human transcriptome analysis was conducted on AsPC-1 samples obtained from three different transfection experiments and was performed with Clariom™ S Assay, Human (ThermoFisher Scientific, USA). Mouse transcriptome was carried out on five pancreatic cancer tissues from each mouse cohort and was performed with Clariom™ S Assay, Mouse (ThermoFisher Scientific, USA).

#### Bioinformatics analyses

Bioinformatics, survival and statistical analyses were performed essentially as previously described.^24^ Experimental details are reported in the Supplementary Data.

### Supplementary information

Supplementary Data

Supplementary Table 1

Supplementary Table 2

Supplementary Table 4

Supplementary Table 5

original and uncropped films of Western blots

## Data Availability

All raw data that support the findings of this study have been submitted to the NCBI Gene Expression Omnibus (GEO; https://www.ncbi.nlm.nih.gov/geo/) under accession number GSE237163 and GSE237164. All data reported in this manuscript are available upon request.

## Code availability

All raw data that support the findings of this study have been submitted to the NCBI Gene Expression Omnibus (GEO; https://www.ncbi.nlm.nih.gov/geo/) under accession number GSE237163 and GSE237164. All data reported in this manuscript are available upon request.
