# Supplementary material for: Nutrient restriction-activated Fra-2 promotes tumor progression via IGF1R in miR-15a downmodulated pancreatic ductal adenocarcinoma
Source: Signal Transduct Target Ther. 2024 Feb 12;9:31. doi: 10.1038/s41392-024-01740-4 (PMC10859382; doi:10.1038/s41392-024-01740-4)
Supplement: Supplementary file 1 — Supplementary Data [file 41392_2024_1740_MOESM1_ESM.docx]

**Supplementary Materials for**

**Nutrient restriction-activated Fra-2 promotes tumor progression *via* IGF1R in miR-15a downmodulated pancreatic ductal adenocarcinoma**

Gian Luca Rampioni Vinciguerra^1,2^, Marina Capece^1^, Luca Reggiani Bonetti^3^, Giovanni Nigita^1^, Federica Calore^1^, Sydney Rentsch^1^, Paolo Magistri^4^, Roberto Ballarin^4^, Fabrizio di Benedetto^4^, Rosario Distefano^1^, Roberto Cirombella^2^, Andrea Vecchione^2^, Barbara Belletti^5^, Gustavo Baldassarre^5^, Francesca Lovat^1^ and Carlo M. Croce^1^.

Correspondence to: Francesca Lovat: [francesca.lovat@osumc.edu](mailto:francesca.lovat@osumc.edu) and Carlo M. Croce [carlo.croce@osumc.edu](mailto:carlo.croce@osumc.edu).

**This PDF file includes:**

Supplementary Figures S1 to S11

Supplementary Table Legends and Supplementary Tables 3 and 6

Supplementary Materials and Methods

Supplementary References


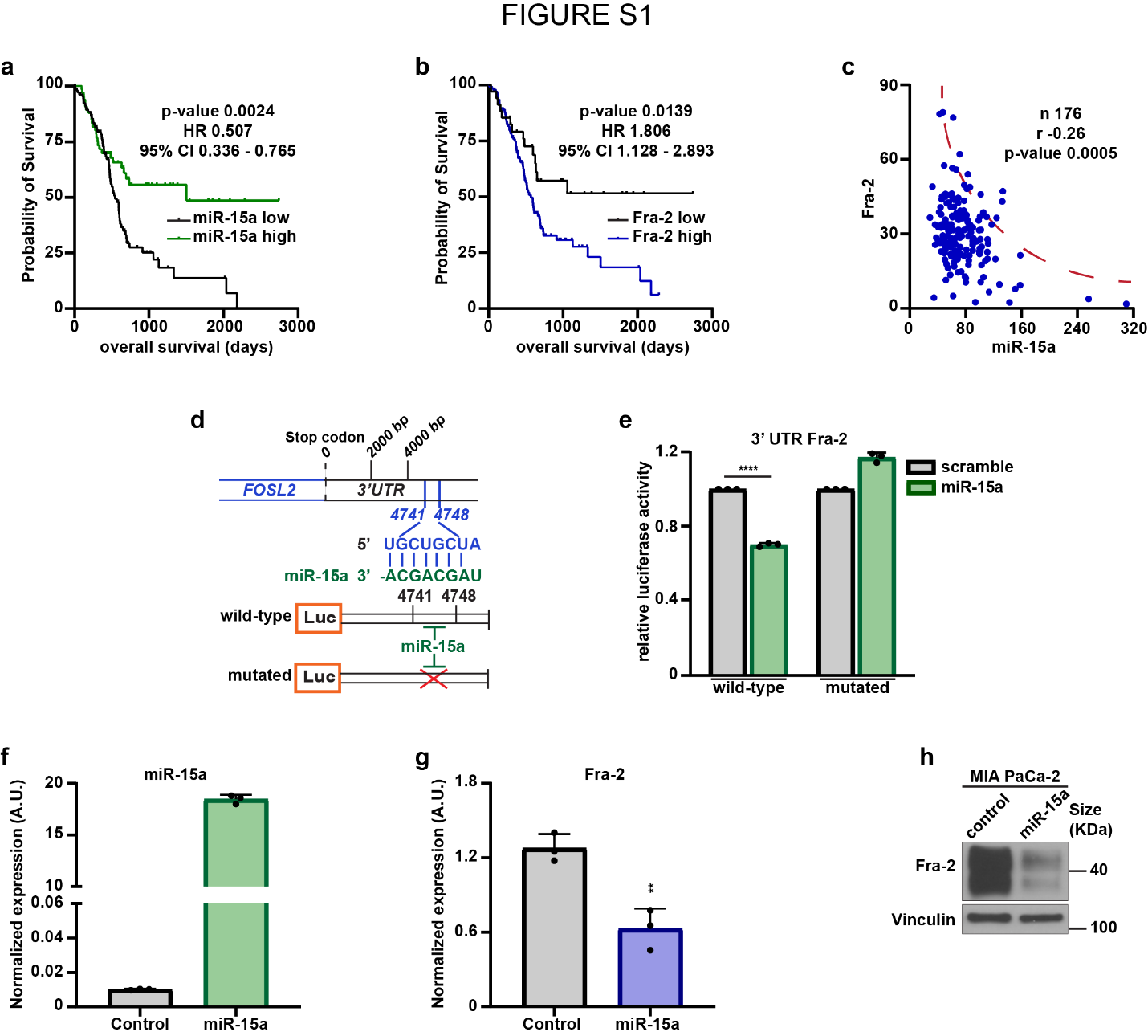


**Figure S1. IGF1 signaling pathway is activated in response to nutrient deprivation and potentially regulated by miR-15a and Fra-2 in PDAC. a, b.** Kaplan-Meier curve evaluating the overall survival of PDAC patients from the TCGA dataset (n=176), clustered according to miR-15a (low n=107; high n=69) (**a**) and Fra-2 (low n=37; high n=132) (**b**) expression. Statistical significance, hazard ratio (HR) and confidence interval (95% CI) were calculated with log-rank (Mantel-Cox) test and reported in the graph. **c.** Scatter plot showing the anti-correlation between Fra-2 and miR-15a in PDAC patients from the TCGA dataset. The number of analyzed samples (n), the Spearman correlation value (r), and its significance (p-value) are reported in the graph. **d.** Schematic representation of miR-15a binding site on FOSL2, encoding Fra-2, 3’UTR and its deletion. **e.** Histogram representing the normalized luciferase activity of psiCHECK2 vector with Fra-2 wild-type 3’UTR insert and with Fra-2 mutated 3’UTR, containing a deletion of the miR-15a target site. Luciferase activity was measured after 24h post-transfection in HEK293 cells. Data represent the mean (±SD) from three independent experiments performed in triplicate and statistical significance was evaluated by unpaired t-test. *****p-value*<0.0001. **f, g.** Histogram reports the normalized expression of miR-15a (**f**) and Fra-2 (**g**), evaluated by qRT-PCR analysis in control and miR-15a overexpressing MIA PaCa-2 cells. In **g**, unpaired t-test was used for statistical analyses and asterisks indicate significant differences compared to controls. ***p*<0.01. **h.** Western blot analysis evaluating Fra-2 protein levels in control and miR-15a overexpressing MIA PaCa-2 cells. Vinculin was used as loading control.


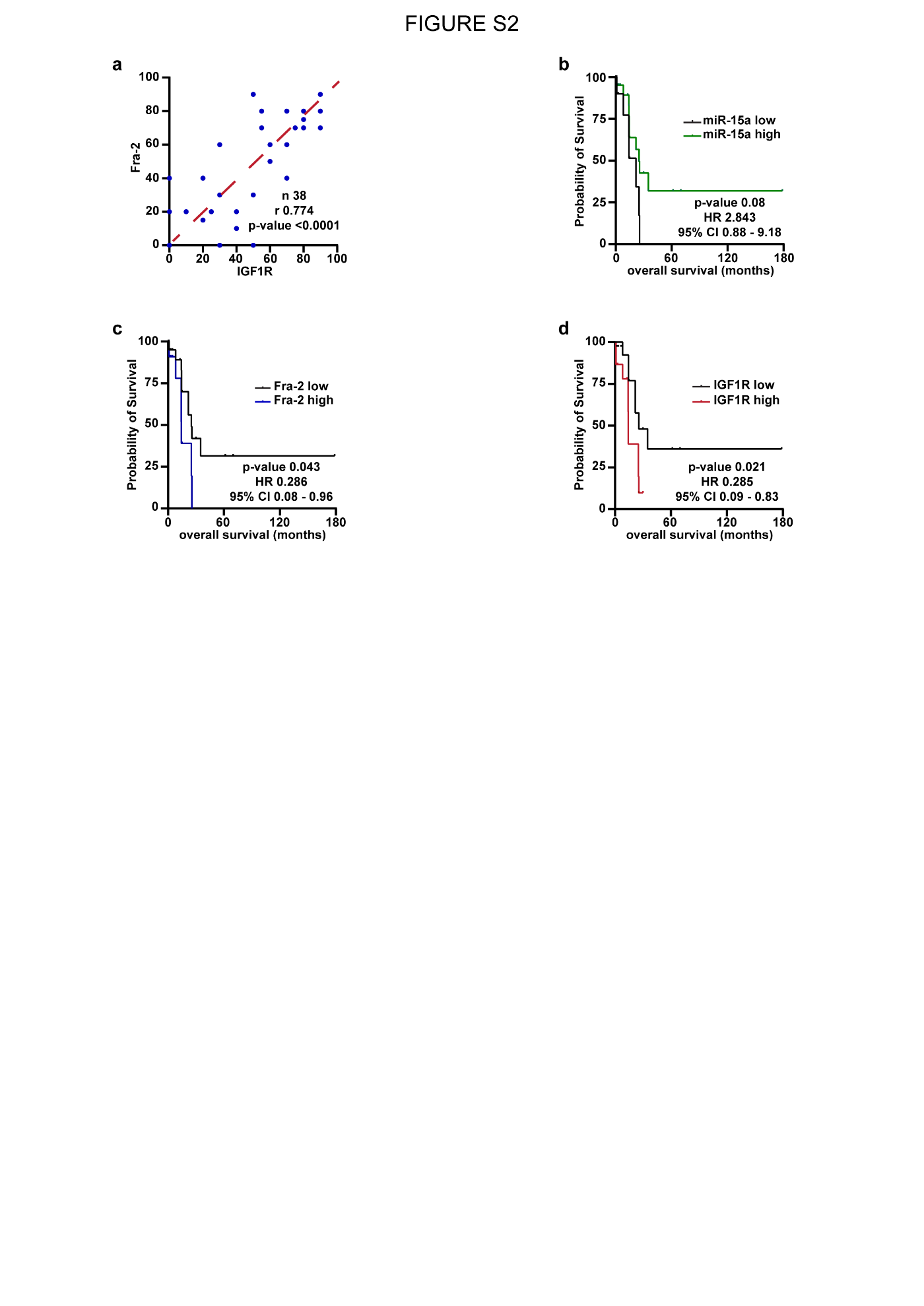


**Figure S2. Fra-2 expression correlates with IGF1R levels and poor prognosis in an independent cohort of PDAC. a.** Scatter plot showing the correlation between Fra-2 and IGF1R expression, evaluated by immunohistochemistry (IHC) in an independent cohort of PDAC patients (n=38). Data are expressed as the percentage of positive cells in the tumor samples and Spearman correlation test was used for statistical analysis. **b-d.** Kaplan Meier curve evaluating the overall survival of PDAC patients from an independent cohort (n=37), stratified according to miR-15a (low n=12; high n=25) (**b**), Fra-2 (low n=21; high n=16) (**c**) and IGF1R (low n=19; high n=18) (**d**) expression. Statistical significance, hazard ratio (HR) and confidence interval (95% CI) were calculated with log-rank (Mantel-Cox) test and reported in the graph.

**
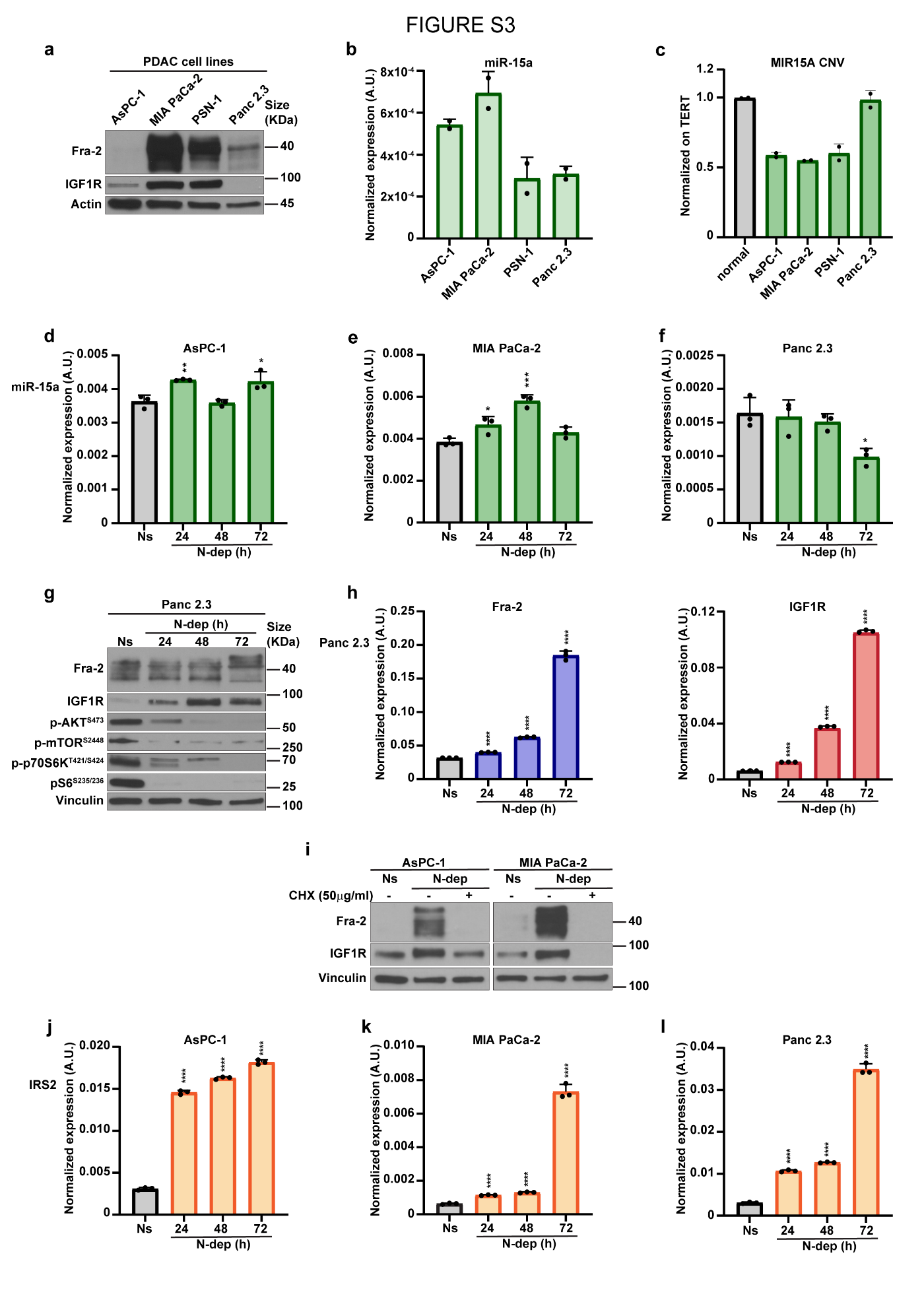
**

**Figure S3. Fra-2 transcriptional activity is triggered by nutrient deprivation and leads to IGF1R overexpression. a.** Western blot analysis evaluating the expression of Fra-2 and IGF1R proteins in a panel of PDAC cell lines. Actin was used as loading control. **b.** Graph reports the normalized expression of miR-15a evaluated by qRT-PCR analysis in the indicated PDAC parental cells. Data represent the mean (±SD) of two independent experiments. **c.** Copy-number variation (CNV) analysis of MIR15A gene in DNA from normal cells and PDAC cell lines. Telomerase reverse transcriptase (TERT) control was used as normalizer. Data represent the mean (±SD) of two independent experiments. **d-f.** Graphs report the normalized expression of miR-15a evaluated by qRT-PCR in AsPC-1 (**d**), MIA PaCa-2 (**e**) and Panc 2.3 (**f**) parental cells, cultured in normal serum (10% FBS, Ns) and nutrient deprivation (0% FBS, N-dep) for the indicated timepoints (hours, h). **g.** Western blot analysis of the indicated proteins in Panc 2.3 parental cells, cultured in normal serum (10% FBS, Ns) and nutrient deprivation (0% FBS, N-dep) and collected at the indicated timepoints (hours, h). Vinculin was used as loading control. **h.** Graphs report the normalized expression of Fra-2 (left) and IGF1R (right), evaluated by qRT-PCR analysis in Panc 2.3 parental cells cultured as described in **d**. **i.** Western blot analysis of the indicated proteins in AsPC-1 and MIA PaCa-2 parental cells cultured in Ns and N-dep for 72 hours, and treated or not with Cycloheximide (CHX) for 48 hours. Vinculin was used as loading control. **j-l.** Graphs report the normalized expression of IRS2 by qRT-PCR analysis in AspPC-1 (**j**), MIA PaCa-2 (**k**) and Panc 2.3 (**l**) parental cells grown as reported for the indicated timepoints. In **d, e, f**, **h, j, k and l** data represent the mean (±SD) of three independent experiments. Unpaired t-test was used for statistical analyses and asterisks indicate significant differences compared to the Ns condition. **p* < 0.05, ***p* < 0.01, ****p* < 0.001, *****p* < 0.0001.

**
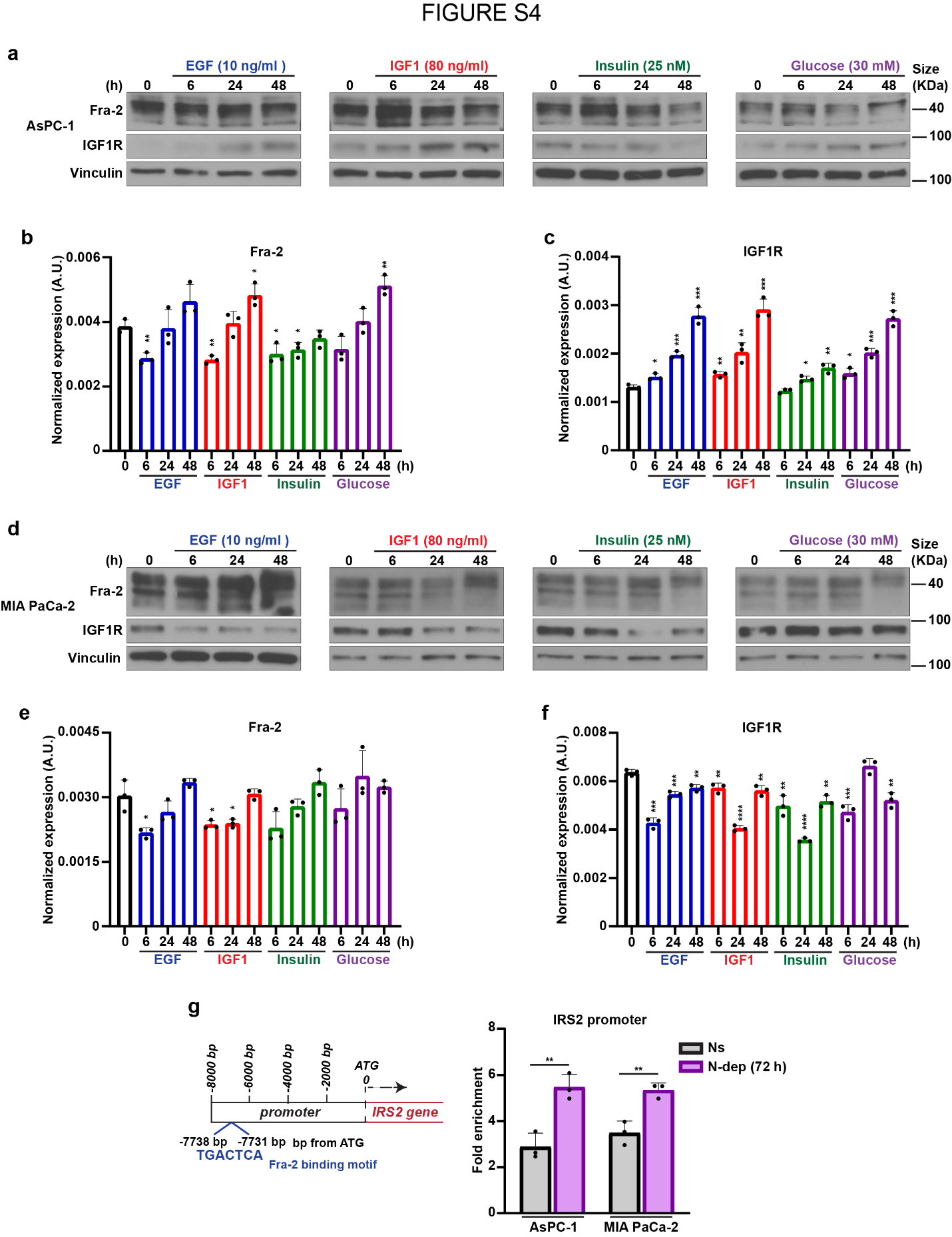
**

**Figure S4. Fra-2 transcriptional activity is triggered by nutrient deprivation and leads to IGF1R overexpression. a.** Western blot analyses of Fra-2 and IGF1R in AsPC-1 parental cells, stimulated with EGF (10 ng/ml), IGF1 (80 ng/ml), Insulin (25 nM) and Glucose (30 mM) and collected at the indicated timepoints (h, hours). Vinculin was used as loading control. **b, c.** Graphs report the normalized expression of Fra-2 (**b**) and IGF1R (**c**), evaluated by qRT-PCR analysis in AsPC-1 parental cells stimulated and collected at different timepoints, as indicated. **d.** Western blot analyses of Fra-2 and IGF1R in MIA PaCa-2 parental cells, stimulated with EGF (10 ng/ml), IGF1 (80 ng/ml), Insulin (25 nM) and Glucose (30 mM) and collected at the indicated timepoints (h, hours). Vinculin was used as loading control. **e, f.** Graphs report the normalized expression of Fra-2 (**e**) and IGF1R (**f**), evaluated by qRT-PCR analysis in MIA PaCa-2 parental cells stimulated and collected at different timepoints, as indicated. In **b**, **c**, **e** and **f**, data represent the mean (±SD) of three independent experiments. Unpaired t-test was used for statistical analyses and asterisks indicate significant differences compared to the 0 timepoint condition. **p*<0.05; ***p*<0.01; ****p*<0.001; *****p* < 0.0001.­ **g.** On the left, schematic representation of Fra-2 binding sequence on IRS2 promoter**.** On the right, graph reports the chromatin immunoprecipitation (ChIP) analysis of Fra-2 bound to the IRS2 promoter in AsPC-1 and MIA PaCa-2 cells cultured in normal serum (10% FBS, Ns) or in nutrient deprivation (0% FBS, N-dep) for 72 hours (h). Data represent the mean (±SD) of three independent experiments. Unpaired t-test was used for statistical analyses and asterisks indicate significant differences compared to the Ns condition. ***p* < 0.01.

**
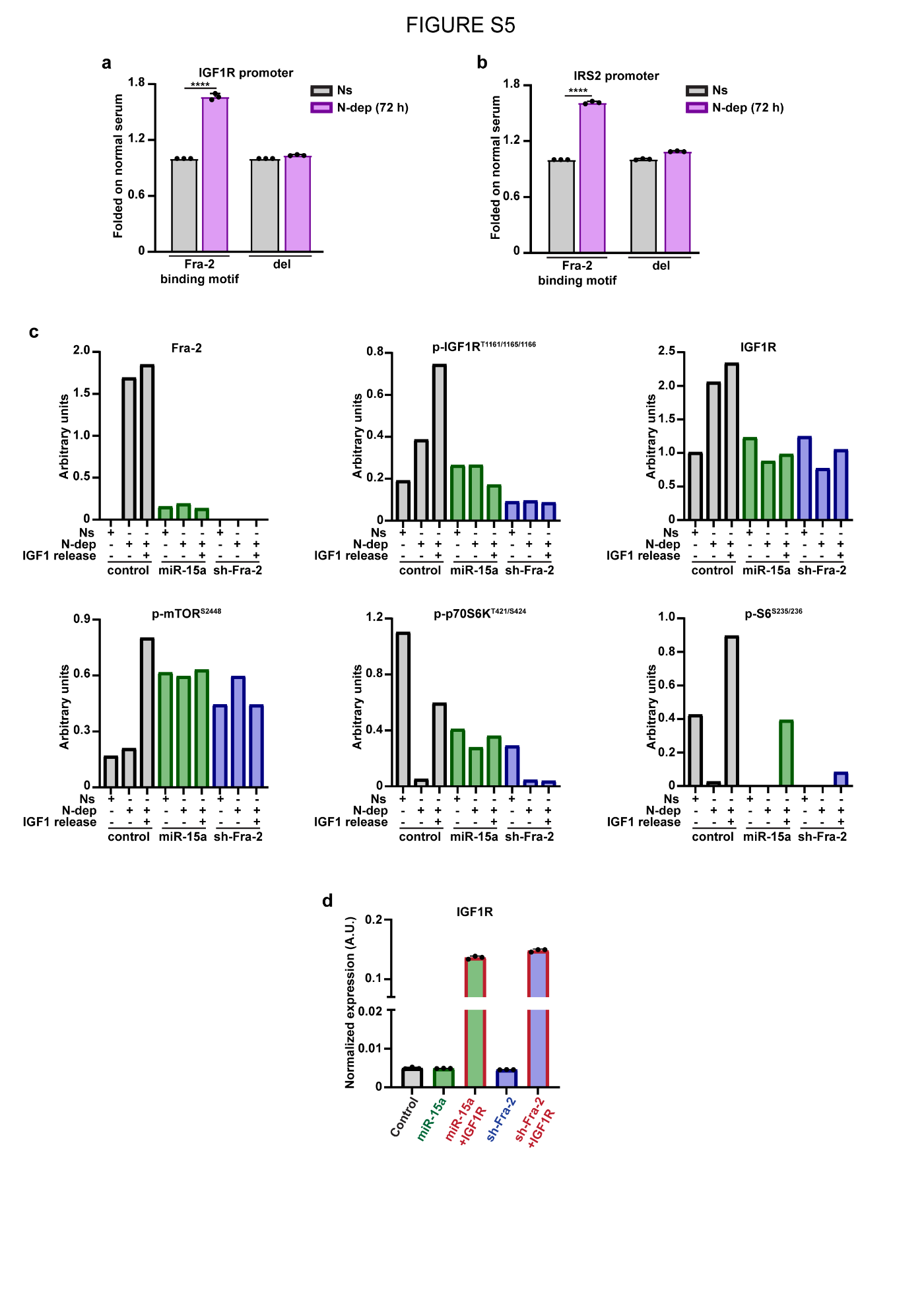
**

**Figure S5. Fra-2 directly regulates IGF1 signaling pathway expression in PDAC response to nutrient deprivation. a, b.** Histograms report the normalized luciferase activity of IGF1R (**a**) and IRS2 (**b**) promoters, containing or not (deleted, del) the Fra-2 binding sequence, in AsPC-1 cells cultured in normal serum (10% FBS, Ns) and in nutrient deprivation (0% FBS, N-dep). Unpaired t-test was used for statistical analyses and asterisks indicate significant differences compared to the Ns condition. **** *p-value* <0.0001. **c.** Histograms reporting the normalized expression of the indicated proteins and phosphoproteins in cell lysates of control, miR-15a overexpressing and Fra-2 silenced AsPC1 cells, cultured in normal serum (Ns), nutrient deprivation (N-dep) and released with IGF1 (80 ng/ml) for 1 hour (IGF1 release), as indicated in Fig. **2g**. **d.** Graph reports the normalized expression of IGF1R, evaluated by qRT-PCR analysis in AsPC-1 cells transfected as indicated and used in the experiments reported in Fig. **3a-f**.

**
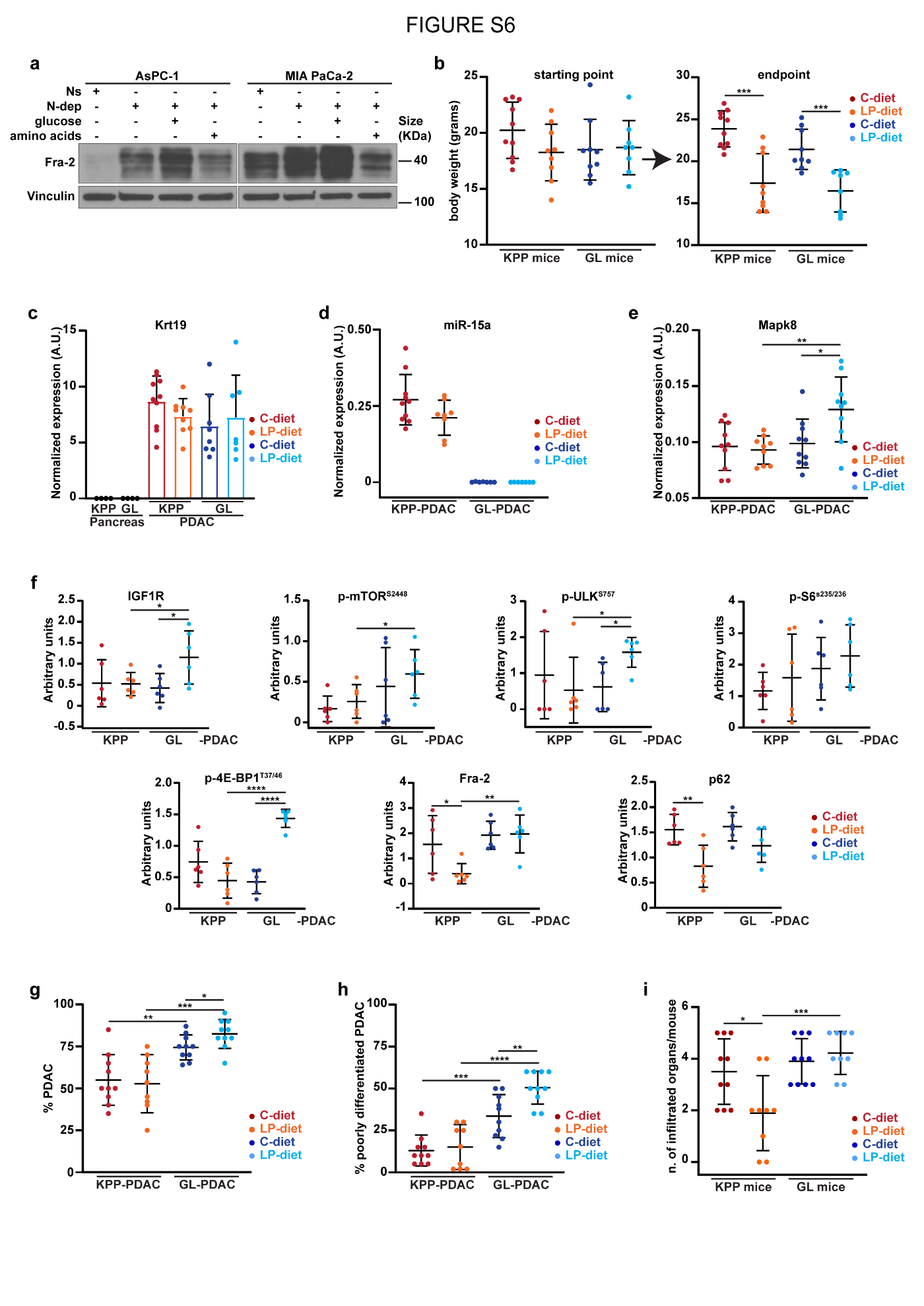
**

**Figure S6. *In vivo*, protein restriction upregulates IGF1 signaling pathway in Mir15a^KO^ PDAC. a.** Western blot analyses of Fra-2 in AsPC-1 and MIA PaCa-2 cells grown in normal serum (10% FBS, Ns) and in nutrient deprivation (0% FBS, N-dep) alone or supplemented with glucose (30 mM) or amino acids (1X Non-Essential Amino Acids Solution and L-glutamine 2 mM), as indicated. **b.** Graphs represent the body weight in grams of KPP and GL mice at the time of tumor induction (on the left, starting point) and at the endpoint (on the right) in the indicated diet cohorts. **c.** Graph reports the normalized expression of Krt19 in normal pancreata from KPP and GL mice and in KPP- and GL-PDAC from mice fed with C- and LP-diet. **d, e.** Graphs report the normalized expression of miR15a (**d**) and MapK8 (**e**) by qRT-PCR in KPP- and GL-PDAC from mice fed with C- and LP-diet. **f.** Graphs reporting the normalized expression of the indicated proteins and phosphoproteins in PDAC ­­lysates collected from KPP and GL mice fed with the indicated diets, as evaluated in Fig. **4h**. **g, h.** Charts represent the percentage of well- and poorly differentiated PDAC components (% PDAC, **g**) and only poorly differentiated PDAC (**h**) evaluated by histology analysis of KPP- and GL-PDAC fed with C- and LP-diet, as indicated in Fig. **4i**. i**.** Chart represents the number of local infiltrated organs evaluated on histological sections of KPP- and GL-PDAC from mice fed with C- and LP-diet, as indicated. In **b-i**, each dot represents a different mouse or tumor, and unpaired t-test was used to verify the statistical significance. **p*<0.05; ***p*<0.01; ****p*<0.001 and *****p*<0.0001.

**
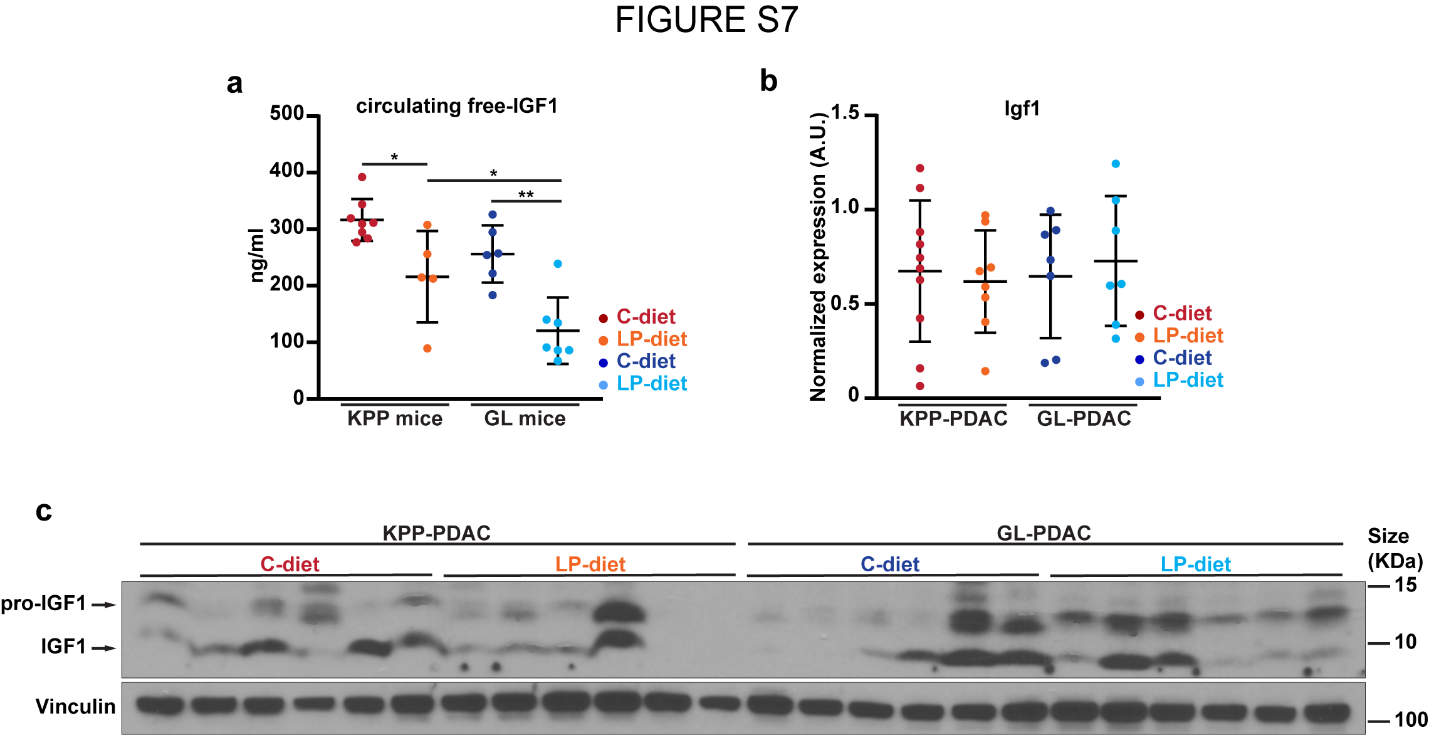
**

**Figure S7. *In vivo*, protein restriction did not alter IGF1 bioavailability in the tumor microenvironment. a.** Graph reproduces the concentration (ng/mL) of circulating free-IGF1 in the plasma of KPP- and GL-mice fed with C- and LP-diet, after 4 hours of fasting, as obtained by ELISA assay. Each dot represents a different mouse and unpaired t-test was used to verify the statistical significance. **p*<0.05; ***p*<0.01. **b.** Graph represents the normalized expression of Igf1 by qRT-PCR in KPP- and GL-PDAC collected from mice fed with C- and LP-diet. Each dot represents a different tumor. **c.** Western blot analysis of IGF1 in KPP- and GL-PDAC collected from mice fed with C- and LP-diet. Vinculin was used as loading control.


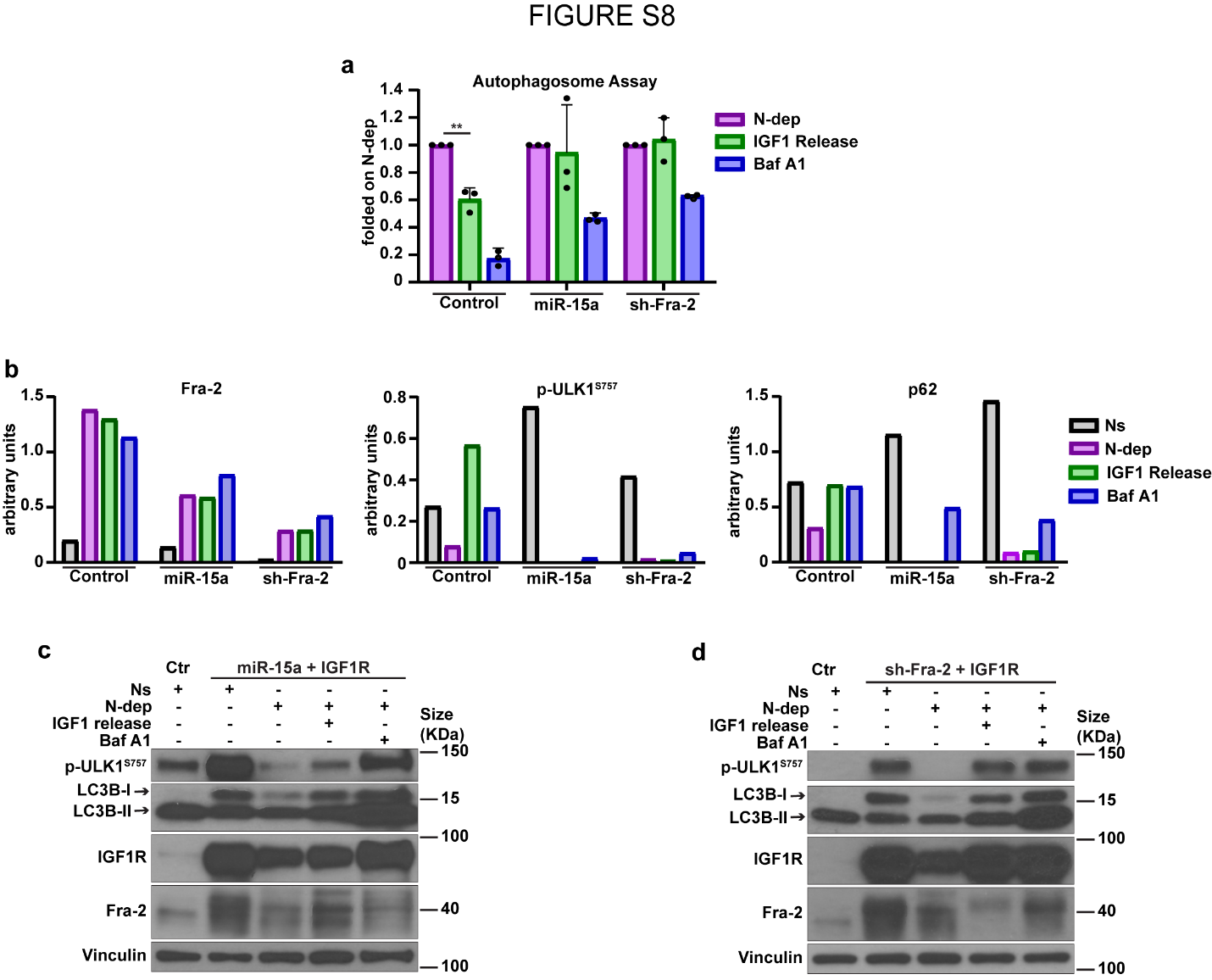


**Figure S8. miR-15a/Fra-2 regulate autophagic flux *via* IGF1R in nutrient deprived PDAC cells. a.** Histograms reporting the fluorescence intensity of autophagosomes in control, miR-15a overexpressing and Fra-2 silenced AsPC-1 cells, cultured in nutrient deprivation (0% FBS, N-dep), and released with IGF1 (80 ng/ml, 2 hours) or Bafilomycin A1 (0.2 μM for 1 hour), as indicated. Data represent the mean (±SD) of three independent experiments performed in esaplicate and are folded over the N-dep condition. Unpaired t-test was used to verify the statistical significance. ***p-value*<0.01. **b.** Histograms represent the normalized expression of the indicated proteins and phosphoproteins in cell lysates of control, miR-15a overexpressing and Fra-2 silenced AsPC-1 cells, cultured in normal serum (10% FBS, Ns), in nutrient deprivation (0% FBS, N-dep), and released with IGF1 (80 ng/ml, 2 hours) or Bafilomycin A1 (0.2 μM for 1 hour), as reported in Fig. **5d**. **c, d.** Western blot analyses of the indicated autophagy markers in control and miR-15a+IGF1R-overexpressing (**c**) and Fra-2-silenced+IGF1R-overexpressing (**d**) AsPC-1 cells, cultured in normal serum (10% FBS, Ns) or in nutrient deprivation (0% FBS, N-dep) and released with IGF1 (80 ng/ml, 2 hours) or Bafilomycin A1 (Baf A1, 0.2 μM for 1 hour), as indicated. Vinculin was used as loading control.

**
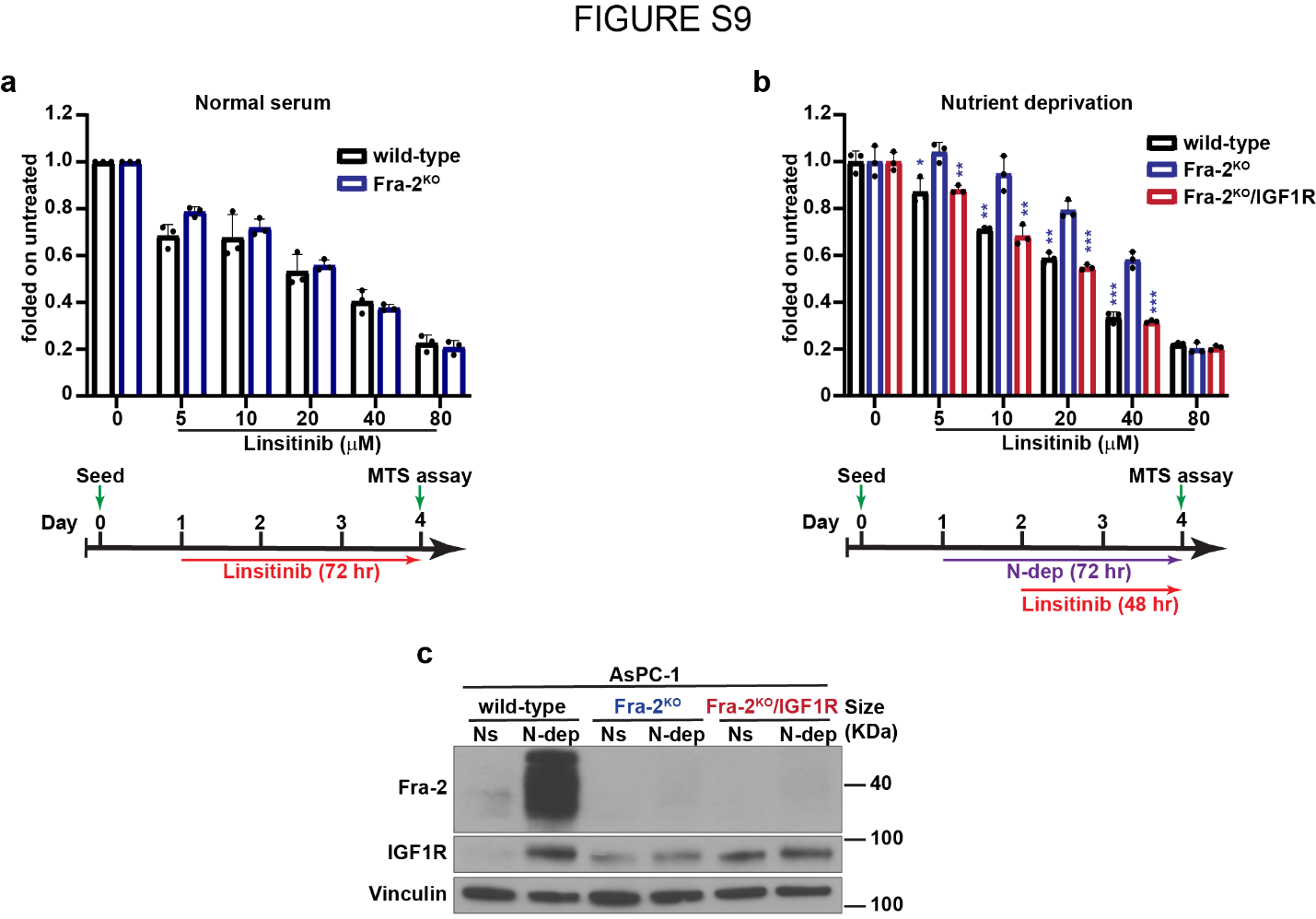
**

**Figure S9. *In vitro*, nutrient deprivation increases sensitivity to IGF1R-inhibition in PDAC cells *via* Fra-2. a.** Dose-response curve of wild-type and Fra-2^KO^ AsPC-1 cells, cultured in normal serum (10% FBS) and treated for 72 h with increasing doses of Linsitinib, as depicted in the experimental timeline shown below the graph. **b.** Dose-response curve of wild-type and Fra-2^KO^ AsPC-1 cells, overexpressing or not IGF1R, cultured in nutrient deprivation (0% FBS) and then treated for 48 hours with increasing doses of Linsitinib, as depicted in the experimental timeline shown below the graph. In **a** and **b**, cell viability was measured by MTS assay and data show the percentage of viable treated cells folded on the untreated condition in three independent experiments. Unpaired t-test was used for statistical analysis and blue asterisks indicate significant differences respect to the Fra-2^KO^ condition. **p* < 0.05; ***p* < 0.01; ****p* < 0.001. **c.** Western blot analysis evaluating the expression of Fra-2 and IGF1R in the indicated cells cultured in normal serum (10% FBS, Ns) and nutrient deprivation (0% FBS, N-dep) for 72 hours as used in **a** and **b**.

**
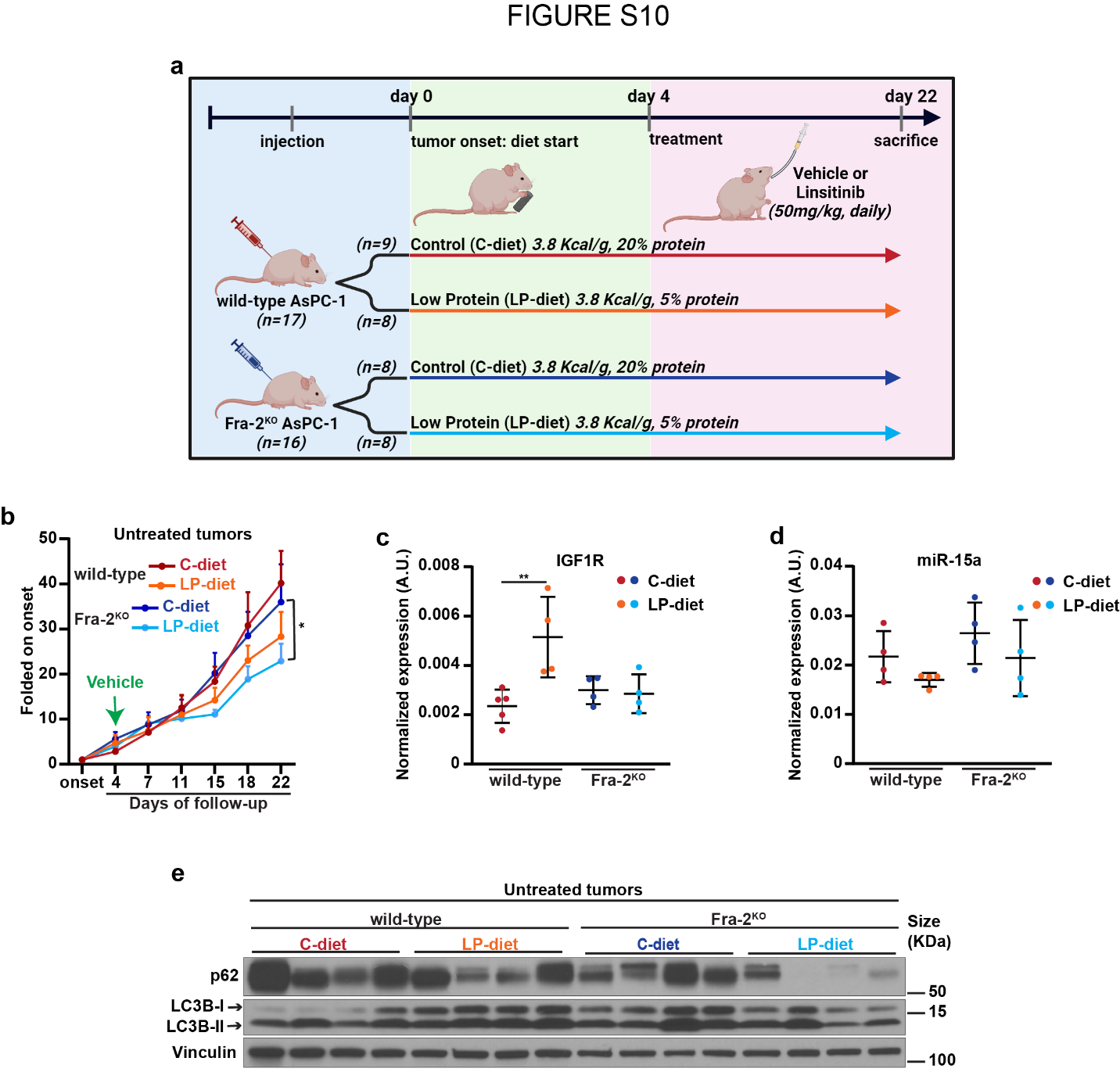
**

**Figure S10. *In vivo*, Fra-2 regulates IGF1R overexpression and autophagic flux in response to protein restriction. a.** Schematic representation of the experimental workflow used for the evaluation of the tumor growth and the response to the IGF1R-inhibitor Linsitinib in mice xenografted with PDAC cells. Nude mice were injected in the flank with either wild-type or Fra-2^KO^ AsPC-1 cells. Once tumor onset was established, mice were randomly subdivided in two different cohorts, fed with control diet (C-diet) or isocaloric, low protein diet (LP-diet) and treated daily with vehicle or Linsitinib for 3 weeks. Created with BioRender.com. **b.** Graph reports the tumor growth rate of wild-type and Fra-2^KO^ PDAC tumors in the untreated (vehicle) cohort of mice fed with C-diet and LP-diet as described in **a**. Green arrow indicates the starting point of vehicle administration. Data represent the mean (±SD) of 4-5 tumors/group folded on their respective volume at the onset and two-way ANOVA was used to verify the statistical significance. **p*<0.05. **c, d.** Graphs report the normalized expression of IGF1R (**c**) and miR-15a (**d**), evaluated by qRT-PCR analysis in tumors explanted from untreated mice cohort (vehicle), as described in **a**. Each dot represents a different tumor and unpaired t-test was used to assess the statistical significance. ***p*<0.01. **e.** Western blot analysis evaluating the expression of the indicated proteins in tumors explanted from the cohort of mice treated with vehicle as described in Fig. **6a**. Vinculin was used as loading control.

**
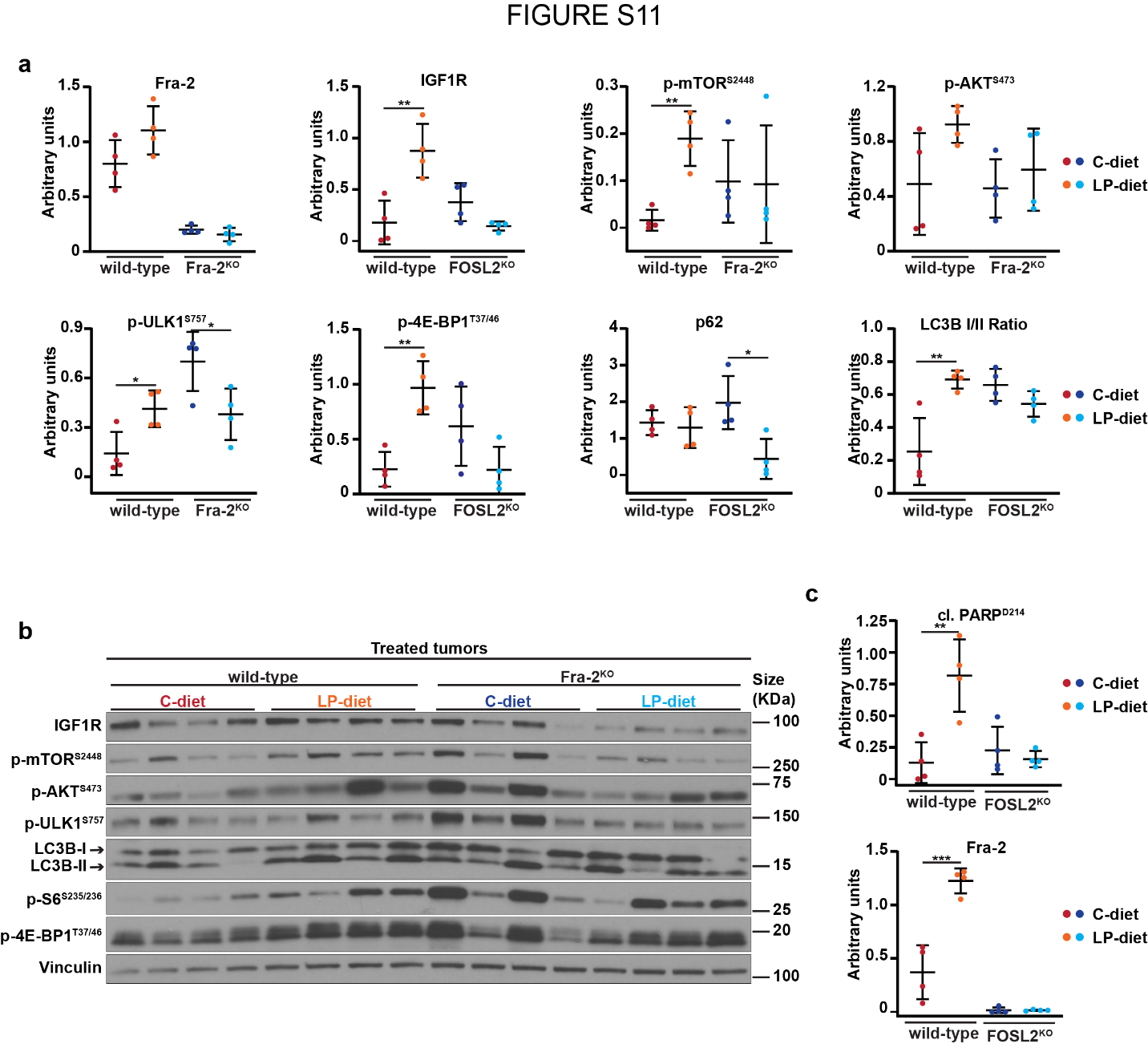
**

**Figure S11. *In vivo*, low protein diet triggers IGF1R overexpression *via* Fra-2 and increases sensitivity to IGF1R-inhibitor in PDAC. a.** Graphs reporting the normalized expression of the indicated proteins and phosphoproteins in tumor lysates collected from untreated mice fed with the indicated diets, as evaluated in Fig. **6b** and **S10e**. **b.** Western blot analysis evaluating the expression of the indicated proteins in tumors explanted from mice treated with Linsitinib and fed with the indicated diets, as described in Figure **S10a**. **c**. Graphs reporting the normalized expression of cleaved PARP^D214^ (top) and Fra-2 (bottom) in tumor lysates collected from mice treated with Linsitinib and fed with the indicated diets, as evaluated in Fig. **6f**. In **a, c**, each dot represents a different tumor lysate, and unpaired t-test was used to verify the statistical significance. **p*<0.05; ***p*<0.01; ****p*<0.001.

**Supplementary Table 1.** Table reports the molecular pathways (column 1) potentially upregulated by low miR-15a (column 2) and high Fra-2 expression (column 3) in PDAC patients from the TCGA dataset (n=176), and enriched in AsPC-1 and MIA PaCa-2 cell lines cultured in nutrient deprivation (column 4). Data were obtained *via* IPA® software and pathways with -log10(p-value)>1.5 are reported in green.

**Supplementary Table 2.** List of significantly overexpressed genes in AsPC-1 and MIA PaCa-2 cell lines cultured in nutrient deprivation compared to normal serum (p-value < 0.01 and Fold-Change > 2.0).

**Supplementary Table 3.** Table summarizes the available clinical features of the independent cohort of 38 PDAC patients. (PD, pancreaticoduodenectomy (Whipple procedure); PPPD, pylorus-preserving pancreaticoduodenectomy (Traverso-Longmire procedure); DSP, distal splenopancreatectomy; SP, splenopancreatectomy)

| **Features** |  | **Value** |
| --- | --- | --- |
| Age at Diagnosis |  |  |
|  | average | 68.21 |
|  | range | 42-83 |
| Gender |  | n. (%) |
|  | Female | 17 (45%) |
|  | Male | 21 (55%) |
| Staging |  |  |
|  | I B | 2 |
|  | II A | 12 |
|  | II B | 24 |
| Grading |  |  |
|  | G1 | 2 |
|  | G2 | 16 |
|  | G3 | 17 |
|  | not reported | 3 |
| Surgical resection |  |  |
|  | PD | 3 |
|  | PPPD | 18 |
|  | DSP | 7 |
|  | SP | 10 |
| Time to recurrence |  |  |
| patient with recurrence |  | 21 |
|  | average (months) | 11.69 |
|  | range | 0.33-35.03 |
| Overall Survival (OS) |  |  |
| patient with OS event |  | 20 |
|  | average (months) | 15.15 |
|  | range | 0.95-34.9 |

**Supplementary Table 4.** Table reports the individual clinical features and the expression of miR-15a (by qRT-PCR), Fra-2 and IGF1R (by IHC staining) in tumor samples of each patient included in the independent cohort of our study. Fra-2 and IGF1R levels are expressed as percentage of positive tumor cells.

**Supplementary Table 5.** List of differentially expressed genes in PDAC from KPP and GL-mice fed with Control diet (n=5 mice per group, p-value<0.05 and |Fold-Change|>1.5).

**Supplementary Table 6.** Table reports primers designed for this study, as described in the materials and methods section.

| Name of primer | Sequence 5’-3’ |
| --- | --- |
| psiCHECK2 FOSL2 Fw | GAAATGGTCCCATTGGAGAGT |
| psiCHECK2 FOSL2 Rv | GCTACTCAACTGAAAGTGGAAATG |
| CRISPR FOSL2 Fw | TCAGAAATTCCGGGTAGATATGC |
| CRISPR FOSL2 Rev | TTGTGGCTGAGGAAAGTGAG |
| ChIP IGF1R promoter Fw | CATCCTACCCGATTGTTTGAG |
| ChIP IGF1R promoter Rev | CCTTAATGTGGTCCGGTTTC |
| ChIP IRS2 promoter Fw | GAATCCAACATTTCACTGGGTTAG |
| ChIP IRS2 promoter Rev | AATGGTGTAGAGTAGAGAAAGTGAC |
| pGL3 IGF1R promoter Fw | CCATCCTACCCGATTGTTTGAG |
| pGL3 IGF1R promoter Rev | AACCAGTACCGACCCTAAGT |
| pGL3 IRS2 promoter Fw | GTTGTGGTCGGTTTGCATTT |
| pGL3 IRS2 promoter Rev | TACCCAGAACATAGGTCTTTGC |

**SUPPLEMENTARY MATERIALS AND METHODS**

**Reagents**

Bafilomycin A1 (Cat. #54645) was purchased from Cell Signaling Technology, USA. Linsitinib (OSI-906, Cat. #HY-10191) was obtained from MedChemExpress, USA. Puromycin (Cat. #A1113803), MEM Non-Essential Amino Acids Solution (100x) (Cat. #11140076), MEM Amino Acids Solution (Cat. #11130051) and Glucose Solution (Cat. #A2494001) were obtained from Gibco (ThermoFisher Scientific, USA). Recombinant human IGF1 protein (Cat. #RP00996), Recombinant human EGF protein (Cat. #RP01030) were purchased from ABclonal, USA. Insulin solution from bovine pancreas (Cat. #I0516) was purchased from Sigma-Aldrich, USA. Cycloheximide (Cat. #01810) was purchased from Sigma Aldrich, USA.

**Generation of stable Fra-2^KO^ clones**

To generate Fra-2 KO clones, CRISPR/Cas-9 technology was used. Stable AsPC-1 Fra-2^KO^ pool was obtained by transduction with lentiviral particles (LV01 U6-gRNA:ef1a-puro-2A-Cas9-2A-tGFP, by Sigma-Aldrich). The gRNA to target FOSL2 was designed by Sigma-Aldrich AAGACCATTGGCACCACCG. Clones were selected after 72 hours from transduction in complete medium supplemented with 1μg/ml of puromycin. To obtain Fra-2^KO^ AsPC-1 clones, single-cell was seeded into 96-well plates. To obtain genomic DNA, cell pellets were resuspended in lysis solution (100 mM Tris-HCl [pH 8.0], 200 mM NaCl, 5 mM EDTA, 1% SDS, and 0.6 mg/ml proteinase K), and incubated at 55°C overnight. After ethanol and sodium acetate precipitation, DNA pellets were washed in 70% ethanol and resuspended in water. The DNA solution was incubated at 60 °C for 15 min and at least 1 hour at room temperature before proceeding. Genomic PCR was performed using Advantage 2 Polymerase Mix (Cat. #639202, Takara Bio USA Inc.,USA ) according to the manufacturer’s instruction, using 50ng of purified DNA. Sanger sequencing was performed on selected clones (primers are listed in Supplementary table 6) and data were analyzed with Synthego ICE software. The clones resulted KO were next confirmed by Western Blot analysis of Fra-2 expression.

**Cycloheximide assay, MTS cell proliferation, colony, soft agar assay, autophagy assay and dose-response curves**

Cycloheximide (CHX) assay was performed in AsPC-1 and MIA PaCa-2 cells cultured in nutrient deprivation (N-dep) for 72 hours. After 24 hours, 50 μg/ml of CHX were added to the medium.

To evaluate the proliferation rate, AsPC-1 and stably overexpressing IGF1R AsPC-1 cells were transfected with control, miR-15a or sh-Fra-2 and, after 5 hours, were counted and seeded into 96-well plates (1000 cells/well) and maintained in normal serum (10% FBS, Ns) or in nutrient restriction (2.5% FBS, N-res) for 72 hours. Cell growth was monitored at the indicated timepoints using RealTime-Glo^TM^ MT Cell Viability assay (Cat. #G9713, Promega, USA). Colony formation assay was performed in AsPC-1 and stably overexpressing IGF1R AsPC-1 cells transfected with control, miR-15a or sh-Fra-2. Cells were counted and seeded into 6-well plates (1000 cells/well) and maintained in normal serum (10% FBS, Ns) or in nutrient restriction (2.5% FBS, N-res) for 9-12 days. Colonies were then fixed, stained with crystal violet solution (0.5 mg/ml in 20% methanol) and counted manually. To evaluate the anchorage-independent cell growth, AsPC-1 and stably overexpressing IGF1R AsPC-1 cells were transfected with control, miR-15a overexpressing or Fra-2 silenced and, after 5 hours, were counted (1000 cells) and resuspended in 2 ml top agar medium (RPMI, 0.4% low melting agarose, SIGMA) in normal serum (10% FBS, Ns) or in nutrient restriction (2.5% FBS, N-res) and quickly overlaid on a previously gelified bottom agar medium (RPMI + 10% FBS, 0.8% low melting agarose, SIGMA). This assay was performed in six-well tissue culture plates, in triplicate. After two weeks, the volume/number of colonies were counted in 5 randomly chosen fields (4x, magnification) using ImageJ software.

Autophagosome assay was performed in control, miR-15a or Fra-2 silenced AsPC-1 cells, seeded into 96 well/plate (4000 cells/well) and cultured in N-dep (0% FBS). After 72 hours, cells were released or not with IGF1 (80 ng/ml, 2 hours) or Bafilomycin A1 (0.2 μM, 1 hour). Measurement of autophagosome fluorescence was performed using the Autophagy Assay kit (Cat. #MAK138, Sigma-Aldrich, USA), following the instructions provided by the manufacturer.

To evaluate the sensitivity to Linsitinib (OSI-906, Cat. #HY-10191, MedChemExpress, USA), AsPC-1 parental cells, Fra-2^KO^ transfected or not with IGF1R were and seeded into 96-well culture plate and then were cultured in normal serum (Ns) (2500 cells/well) or in nutrient restriction (N-res) (4000 cells/well) for 72hr as indicated. Cell viability was assayed using CellTiter 96® AQueous One Solution Cell Proliferation Assay kit (Promega, USA).

**Molecular biology experiments**

**Western blot analysis**

Pancreatic cancer cell lines were lysed in NP-40 cell lysis buffer (Cat. #J60766-AP, ThermoFisher Scientific, USA) supplemented with Protease Inhibitor Cocktail Set III, EDTA-Free – Calbiochem (Cat. #539134, Millipore, USA). PDAC murine samples were homogenized with grinders, on ice. Protein concentration was determined by Protein Assay Dye Reagent Concentrate (Cat. #5000006, Bio-Rad, USA), following the manufacturer’s instructions. Protein lysates were separated in 4-20% Criterion^TM^ TGX Stain-Free^TM^ Protein Gel (Cat. #5678094 and #5678095, Bio-Rad, USA) and transferred onto a nitrocellulose membrane (HybondC, Amersham, UK). Membranes were incubated at 4°C overnight with the indicated primary antibodies: anti-β-Actin (Cat. #A228, Sigma-Aldrich, USA), anti-p-AKT (S473) (Cat. #9271, Cell Signaling Technology, USA), anti-CyclinA (Cat. #sc-271682, Santa Cruz Biotechnology, USA), anti-Fra-2 human (Cat. #19967, Cell Signaling Technology, USA), anti-Fra-2 mouse (Cat. #HPA004817, Sigma-Aldrich, USA), anti-GAPDH (Cat. #GTX100118, Genetex, USA), anti-IGF1 (Cat. #73034, Cell Signaling Technology, USA), anti-IGF1R (Cat. #9750, Cell Signaling Technology, USA), anti-p-IGF1R (T1161/1165/1166) (Cat. #ABE332, Millipore Sigma, USA), anti-LC3A/B (Cat. #12741, Cell Signaling Technology, USA), anti-mTOR (Cat. #2983, Cell Signaling Technology, USA), anti-p-mTOR (S2448) (Cat. #2971, Cell Signaling Technology, USA), anti-Cleaved PARP (D214) (Cat. #9541, Cell Signaling Technology, USA), anti-p27 (Cat. #554069, BD Biosciences, USA) anti-p70S6K (Cat. #2708, Cell Signaling Technology, USA), anti-p-p70S6K (T421/S424) (Cat. #9204, Cell Signaling Technology, USA), anti-pS6 (S235/236) (Cat. #4858, Cell Signaling Technology, USA), anti-S6 (Cat. #2217, Cell Signaling Technology, USA), anti-ULK1 (Cat. #8054, Cell Signaling Technology, USA), anti-p-ULK1 (S757) (Cat. #14202, Cell Signaling Technology, USA), anti-Vinculin (Cat. #18058, Abcam, UK), anti-4E-BP1 (Cat. #9644, Cell Signaling Technology, USA), anti-p-4E-BP1 (T37/46) (Cat. #2855, Cell Signaling Technology, USA).

After incubation with appropriate horseradish peroxidase-conjugated secondary antibodies (Cat. #NA931V and #NA934V, GE Healthcare, USA), signal was detected using Immobilon Forte HPR detection reagent (Cat. #WBLUF0500, Millipore, USA). Densitometry plots and signal intensity quantification were obtained using ImageJ software (U. S. National Institutes of Health, Bethesda, Maryland, USA).

**Chromatin immunoprecipitation assay**

AsPC-1 and MIA PaCa-2 cell lines cultured in normal serum (10% FBS) and nutrient deprivation (0%FBS) for 72 hours were crosslinked with 1% formaldehyde (Cat. #F79-500, Fisher Chemicals, USA) and chromatin was prepared using MNase enzymatic digestion according to the protocol. Chromatin immunoprecipitation (ChIP) was performed using SimpleChIP Enzymatic Chromatin ImmunoPrecipitation Kit (Magnetic Beads, Cat. #9005S, Cell Signaling Technology, USA). The obtained chromatin samples were incubated at 4°C overnight with the following antibodies: normal rabbit IgG (Cat. #2729, Cell Signaling technology, USA), anti-Fra-2 (Cat. #19967, Cell Signaling Technology, USA) and anti-H3 (Cat. #4620, Cell Signaling Technology, USA) as positive control. Immunoprecipitated chromatin was purified and analyzed by the real-time quantitative PCR using SimpleChIP Universal qPCR Master Mix (Cat. #88989, Cell Signaling Technology, USA). Data were analyzed with the fold enrichment method compared to an unrelated antibody (normal rabbit IgG). Primers used to amplify the indicated IGF1R and IRS2 promoters are listed in Supplementary Table 6.

**RNA isolation and quantitative Real-time PCR**

Total RNA was isolated from cell lines and murine samples using TRIzol^TM^ Reagent (Cat. #15596026, Invitrogen, USA), following the instructions provided by the manufacturer. Pancreatic cancer and liver tissues from mice were fragmented with grinders on ice. RNA was retro-transcribed using the High-Capacity cDNA Reverse Transcription Kit (Cat. #4368813, Applied Biosystems, ThermoFisher Scientific, USA). For miR-15a analysis on our cohort of patients, RNA was extracted from paraffin-embedded PDAC samples using RecoverAll^TM^ Total Nucleic Acid Isolation (Cat. #AM1975, Invitrogen by ThermoFisher Scientific, USA), according to the manufacturer’s instructions.

For qRT-PCR, TaqMan® miRNA assay (Assay ID 000389, ThermoFisher Scientific, USA) was used to detect mature miR-15a-5p. RNU44 (Assay ID 001094, ThermoFisher Scientific, USA) and snoRNA234 (Assay ID 001234, ThermoFisher Scientific, USA) were used as normalizers for human and mouse samples, respectively. TaqMan® gene expression assays (ThermoFisher Scientific, USA) were used to detect mRNA expression of: human FOSL2/Fra-2 (Assay ID Hs01050117_m1), human IGF1R (Assay ID Hs00609566_m1), human IRS2 (Assay ID Hs00275843_s1), mouse Fosl2/Fra-2 (Assay ID Mm0048442_m1), mouse Igf1r (Assay ID Mm00802831_m1), mouse Irs2 (Assay ID Mm03038438_m1), mouse Mapk8 (Assay ID Mm00489514_m1), mouse Igf1 (assay ID Mm00439560_m1) and mouse Krt19 (Assay ID Mm00492980_m1). Human ACTB (Assay ID Hs03023943_g1) and mouse Pgk1 (Assay ID Mm00435617_m1) were used as normalizers.

**Dual-Luciferase reporter assay**

The predicted Fra-2 binding sequences on IGF1R and IRS2 promoter were amplified by PCR using specific primers. After digestion with digested with NheI and XhoI (New England Biolabs), PCR products were cloned into pGL3-promoter vector (Promega). The mutants of Fra-2 binding sequence on IGF1R and IRS2 promoter were generated using QuikChange II XL Site-Directed Mutagenesis Kit (Agilent), according to the manufacturer’s protocol. AsPC-1 cells were co-transfected with 1 μg of pGL3-promoter constructs and 100 ng of pRL-TK (Renilla luciferase control reporter, Promega) in 12-well plate using Lipofectamine 2000 (Life Technologies) according to manufacturer’s recommendations. After 4 hours from transfection, cells were cultured in normal serum (10% FBS) or in nutrient deprivation (0% FBS). After 72hr, Dual-Luciferase Assay (Promega) was performed to measure the reporter activity.

Primers are listed in Supplementary Table 6.

***In vivo* experiments (GL generation and nude mice)**

KPP mice^1^ (Kras^LSL-G12D^, Ptf1a^Cre-ERTM^, Pten^flox^) were purchased from The Jackson Laboratory, USA. GL model was generated crossbreeding KPP and Mir15a^KO^ mice^2^. GL mice were viable, efficiently reproduced, and, at birth, had no macroscopic abnormalities. Correct Mendelian ratios were observed among littermates.

At 6-8 weeks of age, KPP and GL mice were injected 3x times, one every other day, with 9 mg/40 gr of Tamoxifen (Cat. #S1238, Selleckchem, USA) resuspended in corn oil (Cat. #S6701, Selleckchem, USA). After the induction, mice were randomly distributed in two different cohorts, fed with either control diet (C-diet, TD.91352, Envigo, USA) or low protein diet (LP-diet, TD.99168, Envigo, USA) for 60 days as described (Fig. 4a).

At the endpoint, mice fasted for 4 hours and blood samples were collected in EDTA-coated tubes through intracardiac bleed. Complete blood samples were further centrifuged, and sera were used to assess the circulating levels of IGF1 with Mouse/Rat IGF1 Quantikine ELISA kit (Cat. #MG100, R&D Systems, USA) according to the manufacturer’s protocol.

To evaluate the tumor growth and onset of PDAC cells, primary tumors were established by subcutaneous injection of 1.5 × 10^6^ wild-type (17 mice) and Fra-2^KO^ (16 mice) AsPC-1 cells into the flanks of female athymic nude mice (The Jackson Laboratory, USA). At the tumor onset (Day 0), mice were randomly distributed in two different cohorts, fed with either control diet (C-diet, TD.91352, Envigo, USA) or low protein diet (LP-diet, TD.99168, Envigo, USA) for 3 weeks as described (Supplementary Fig. S10a). At days 4 after the tumor onset, mice were further distributed in two groups treated orally and daily with 50 mg/kg of Linsitinib (OSI-906, Cat. #HY-10191, MedChemExpress, USA) dissolved in a 25 mM tartaric acid solution (Cat. #HY-Y0293, MedChemExpress, USA) or with vehicle alone. Growth of primary tumors was monitored by measuring tumor width (W) and length (L) with a caliper two times per week and calculating tumor volume based on the formula: Tumor volume (mm3) = (W^2^ × L)/2.

**Bioinformatics analyses**

Genes (fragments per kilobase of exon per million mapped fragments - FPKM) and miRNA isoforms (reads per million mapped reads or counts per million mapped reads - RPM) L3 expression data, along with patients' clinical (e.g., patient survival) data from the TCGA-PDAC dataset, were downloaded from the Genomic Data Commons Data Portal (https://portal.gdc.cancer.gov/). In this work, we considered a cumulative expression for hsa-miR-15a-5p, generated by the contribution of all miRNA isoforms having up to one nucleotide added at 5'-end. As quality control, levels of tumor purity in TCGA samples were evaluated as described in our previous work^3^ and leveraging the ESTIMATE method^4^. Differences in the amount of neoplastic cells in relation to miR-15a expression were excluded.

The analysis for Clariom™ S Human Affymetrix panel (of ~21K genes) was carried out by Transcriptome Analysis Console (TAC) software (v4 - ThermoFisher Scientific) and consisted of three main steps: 1) data quality control; 2) normalization (signal space transformation robust multiple-array average); 3) differentially expression analysis employing the eBayes method from *limma* R package included in TAC software. Upregulated genes with a Fold-Change>2.0 and a pvalue <0.01 were considered for the downstream analyses. The analysis of the Clariom™ S Mouse Affymetrix panel (of ~22K genes) followed the same steps described above. Differential expressed genes with |Fold-Change|>1.5 and pvalue<0.05 were considered for the downstream analyses.

Predicted target genes for miR-15a-5p, considered in Fig. 1a, were taken from the TargetScan tool (v7.2)^5^.

Functional enrichment analyses were performed by using Ingenuity® Pathway Analysis (IPA®) software (v90348151). Settings used included experimentally observed data for the human species for the enrichment analyses present in Fig. 1a and observed data for the mouse species for the enrichment analyses present in Fig. 4d.

The heat map presented in Fig. 4b was generated by using the *pheatmap* (v1.0.12) R (v4.2.2) package.

**Survival and statistical analysis**

Survival analysis of the TCGA dataset and the independent cohort of PDAC. miR-15a, Fra-2 and IGF1R expression levels were obtained by The Cancer Genome Atlas (TCGA) from 176 PDAC tissue samples. The expression of miR-15a, Fra-2 and IGF1R was assessed by qRT-PCR and IHC staining in an independent cohort of 38 PDAC tissue samples. In 1 case, clinical information was not available and the patient was excluded from survival analysis. For each cohort, the Kaplan–Meier method was performed to generate survival curves and the statistical significance of the difference between survival curves of high- vs. low-expression groups was evaluated using the log-rank test. The cut-off point for the two groups was changed iteratively, and the cut-off that reached the most significant p-value was selected.

Graphs and statistical analyses were performed using PRISM (version 9, GraphPad, Inc.). In all experiments, differences were considered significant when p-value was < 0.05. Statistical analyses including Kaplan–Meyer survival analyses, paired and unpaired t-tests, Mann–Whitney unpaired t-test and Spearman correlation test and two-way ANOVA test were used as appropriate and as specified in the legend of each figure.

**SUPPLEMENTARY REFERENCES**

1. Talbert, E. E. *et al.* Modeling Human Cancer-induced Cachexia. *Cell Rep* **28**, 1612-1622.e4 (2019).

2. Klein, U. *et al.* The DLEU2/miR-15a/16-1 cluster controls B cell proliferation and its deletion leads to chronic lymphocytic leukemia. *Cancer Cell* **17**, 28–40 (2010).

3. Distefano, R. *et al.* Pan-Cancer Analysis of Canonical and Modified miRNAs Enhances the Resolution of the Functional miRNAome in Cancer. *Cancer Res* **82**, 3687–3700 (2022).

4. Yoshihara, K. *et al.* Inferring tumour purity and stromal and immune cell admixture from expression data. *Nat Commun* **4**, 2612 (2013).

5. Agarwal, V., Bell, G. W., Nam, J.-W. & Bartel, D. P. Predicting effective microRNA target sites in mammalian mRNAs. *eLife* **4**, e05005 (2015).
