# Supplementary material for: Nutrient restriction-activated Fra-2 promotes tumor progression via IGF1R in miR-15a downmodulated pancreatic ductal adenocarcinoma
Source: Signal Transduct Target Ther. 2024 Feb 12;9:31. doi: 10.1038/s41392-024-01740-4 (PMC10859382; doi:10.1038/s41392-024-01740-4)
Supplement: Supplementary file 2 — Supplementary Table 1 [file 41392_2024_1740_MOESM2_ESM.pdf]

**Canonical Pathways**	**Negatively correlated with miR15a in PDAC - TCGA**	**Positively correlated with Fra-2 in PDAC - TCGA**	**enriched in N-dep AsPC-1 and MiaPaca-2 cell lines**
Ferroptosis Signaling Pathway	1.579798294	1.668003109	3.667411762
IGF-1 Signaling	1.634074133	9.103712686	3.60671449
Hepatic Fibrosis Signaling Pathway	1.560665118	21.26896901	2.410953964
Natural Killer Cell Signaling	1.667074632	9.923428635	1.952351001
Regulation Of The Epithelial Mesenchymal Transition By Growth Factors Pathway	1.627507027	13.12920439	1.87157652
Autophagy	0.886530286	4.083257217	5.982432706
CLEAR Signaling Pathway	1.079258226	3.777478167	5.25678232
NUR77 Signaling in T Lymphocytes	0	4.918279043	4.490601269
PD-1, PD-L1 cancer immunotherapy pathway	0.949073214	9.474355306	3.969771432
Pancreatic Adenocarcinoma Signaling	0.285790988	5.486350271	3.433764292
Type II Diabetes Mellitus Signaling	1.292362528	1.680115799	3.337646657
Caveolar-mediated Endocytosis Signaling	0	5.681791078	3.246008088
Senescence Pathway	1.06592474	8.24658156	3.074604001
IL-3 Signaling	0.4322881	9.924646281	3.009527043
Growth Hormone Signaling	1.231243716	6.870332132	2.920445095
IL-7 Signaling Pathway	0	5.248574563	2.920445095
MSP-RON Signaling In Macrophages Pathway	0.317640776	8.055538776	2.907358176
UVA-Induced MAPK Signaling	0.358072377	2.933679329	2.904055338
T Cell Exhaustion Signaling Pathway	1.164560036	10.78979066	2.868253539
IL-9 Signaling	0.741561883	4.832224491	2.7900062
Virus Entry via Endocytic Pathways	0.303802675	5.016109931	2.667596704
Insulin Receptor Signaling	0.737406623	3.360271853	2.63919905
Role Of Osteoblasts In Rheumatoid Arthritis Signaling Pathway	0.493627343	15.29423694	2.634329467
Thrombopoietin Signaling	0.515489894	9.010444254	2.547485762
Docosahexaenoic Acid (DHA) Signaling	0	2.914010839	2.517459053

Cytotoxic T Lymphocyte-mediated Apoptosis of Target Cells	0	1.532226472	2.517459053
IL-15 Production	0	5.472723645	2.411864835
ERBB4 Signaling	1.243150786	4.963911631	2.370961957
Role Of Chondrocytes In Rheumatoid Arthritis Signaling Pathway	0.278516157	7.458559316	2.334135107
Prostate Cancer Signaling	0.314803835	5.632982191	2.319195111
PTEN Signaling	0.670055118	11.56869316	2.228290349
CNTF Signaling	0.55386719	6.268639487	2.222076939
Multiple Sclerosis Signaling Pathway	0	3.261684076	2.211615815
Nitric Oxide Signaling in the Cardiovascular System	0.341693383	1.910182206	2.188220277
EGF Signaling	0	7.235720573	2.17806361
Chronic Myeloid Leukemia Signaling	0.329355565	5.637539447	2.151618783
Th1 Pathway	0.335443459	7.348520587	2.127427705
Neuroinflammation Signaling Pathway	0.330332322	9.979345436	2.09856271
Oxytocin In Brain Signaling Pathway	0	3.676740478	2.080004754
Thyroid Cancer Signaling	1.153383382	8.559925724	2.061464804
Macrophage Alternative Activation Signaling Pathway	0.219345635	6.395100307	2.060004835
Ribonucleotide Reductase Signaling Pathway	0	4.574775968	2.060004835
Acute Myeloid Leukemia Signaling	1.004419369	6.834304564	2.056847365
Glioma Signaling	1.442674157	5.874091766	2.037461673
Role of JAK1, JAK2 and TYK2 in Interferon Signaling	0	2.059836475	2.029568002
Role of PI3K/AKT Signaling in the Pathogenesis of Influenza	0	2.546611498	1.983908015
Role of JAK1 and JAK3 in γc Cytokine Signaling	1.280188102	9.375613586	1.973950798
Systemic Lupus Erythematosus In B Cell Signaling Pathway	0.396409196	14.4391756	1.972920722
ERB2-ERBB3 Signaling	0.509536967	5.626066531	1.936052377
ERBB Signaling	0.98007245	6.698921496	1.934405071
Non-Small Cell Lung Cancer Signaling	0.372014253	4.509950671	1.934405071

Estrogen-Dependent Breast Cancer Signaling	0.427816633	3.941498423	1.925103691
Role of Tissue Factor in Cancer	0.314803835	9.905508461	1.92286944
Regulation of the Epithelial-Mesenchymal Transition Pathway	0.528115121	11.22849319	1.891457533
Tumor Microenvironment Pathway	0.595374904	12.70670372	1.883436037
Erythropoietin Signaling Pathway	0.230141718	9.473716732	1.881019303
ILK Signaling	0.521621309	7.968728912	1.87157652
Calcium-induced T Lymphocyte Apoptosis	0	4.298512035	1.862909994
FGF Signaling	0.410632518	5.844362642	1.860917374
UVB-Induced MAPK Signaling	0	2.953823335	1.854151612
Dendritic Cell Maturation	0	7.100762698	1.84171846
FLT3 Signaling in Hematopoietic Progenitor Cells	0.419087853	7.303415251	1.829758885
IL-17A Signaling in Airway Cells	0	3.861903505	1.827606196
Neuregulin Signaling	0.314803835	8.845197378	1.824681917
JAK/STAT Signaling	1.074507277	9.369535856	1.799201097
T Cell Receptor Signaling	0.518412749	14.49986808	1.794069702
IL-23 Signaling Pathway	0.666805464	4.103299896	1.741589571
Role of Pattern Recognition Receptors in Recognition of Bacteria and Viruses	0	8.702025263	1.70274235
NGF Signaling	0.803164329	7.225522646	1.70274235
Corticotropin Releasing Hormone Signaling	0.255986023	2.468082232	1.702670653
Lymphotoxin β Receptor Signaling	0	3.374126583	1.697685907
GM-CSF Signaling	1.196745626	8.470309089	1.694207953
Systemic Lupus Erythematosus In T Cell Signaling Pathway	0.445705449	13.68174328	1.687577121
Endocannabinoid Cancer Inhibition Pathway	0.707420035	5.176998173	1.681055004
PKCθ Signaling in T Lymphocytes	0.618953072	9.143628792	1.669452281
OX40 Signaling Pathway	0	2.162182108	1.661204587
IL-10 Signaling	0.24562048	10.6270736	1.617870482
Aldosterone Signaling in Epithelial Cells	0	2.595255563	1.610753899
GDNF Family Ligand-Receptor Interactions	1.163957852	4.991232688	1.572175396

Endometrial Cancer Signaling	0.534081817	4.959553946	1.557438446
SPINK1 General Cancer Pathway	0.527758087	2.59083832	1.557438446
NRF2-mediated Oxidative Stress Response	0	1.657902062	1.546389301
Neurotrophin/TRK Signaling	0.446154342	7.111919213	1.543284257
Angiopoietin Signaling	1.153383382	4.799981898	1.543284257
CDX Gastrointestinal Cancer Signaling Pathway	0	9.820385753	1.53564743
Allograft Rejection Signaling	0	2.62013278	1.530274697
VEGF Signaling	0.98007245	5.330150022	1.523424827
Melanocyte Development and Pigmentation Signaling	0.375624936	5.078244063	1.523424827
IL-15 Signaling	0.436833514	6.633219091	1.515002083
Role of JAK family kinases in IL-6-type Cytokine Signaling	0	1.974240211	1.515002083
Superpathway of Cholesterol Biosynthesis	0	0	15.04544137
Cholesterol Biosynthesis III (via Desmosterol)	0	0	9.74937762
Cholesterol Biosynthesis I	0	0	9.74937762
Cholesterol Biosynthesis II (via 24,25-dihydrolanosterol)	0	0	9.74937762
Superpathway of Geranylgeranyldiphosphate Biosynthesis I (via Mevalonate)	0	0	5.614994793
Mevalonate Pathway I	0	0	5.226065317
Zymosterol Biosynthesis	0	0	4.419089902
Endoplasmic Reticulum Stress Pathway	0	0.642527787	2.99087922
Myelination Signaling Pathway	0	0	2.973131849
Apelin Pancreas Signaling Pathway	0.648116238	1.015259289	2.857112351
Epoxysqualene Biosynthesis	0	0	2.782004239
Coronavirus Pathogenesis Pathway	0	1.161428354	2.674772506
Unfolded protein response	0	0	2.674162973
Antigen Presentation Pathway	0	0.309502522	2.648448828

Acetylcholine Receptor Signaling Pathway	0	0	2.62084281
Chaperone Mediated Autophagy Signaling Pathway	0	0	2.430579678
DHCR24 Signaling Pathway	0	0	2.362481983
Leucine Degradation I	0	0	2.3296526
Amyotrophic Lateral Sclerosis Signaling	0	1.281126351	2.283117684
Retinoic acid Mediated Apoptosis Signaling	0	1.091618727	2.228558457
Insulin Secretion Signaling Pathway	0	1.498745672	2.151618783
Valine Degradation I	0	0	2.10738902
Ketogenesis	0	0	2.062866404
Interferon Signaling	0	0.641480729	2.051872657
Cachexia Signaling Pathway	0	0	2.037247348
Trans, trans-farnesyl Diphosphate Biosynthesis	0	0	2.027616807
Fatty Acid Activation	0	0.236862423	1.951093342
γ-glutamyl Cycle	0	0	1.951093342
UDP-N-acetyl-D-glucosamine Biosynthesis II	0	1.151980215	1.81758773
Acetate Conversion to Acetyl-CoA	0	0.32615211	1.81758773
NAD Biosynthesis from 2-amino-3-carboxymuconate Semialdehyde	0	0	1.81758773
WNK Renal Signaling Pathway	0	0	1.79272572
EIF2 Signaling	0	0	1.70465881
NAD Signaling Pathway	0.269198937	1.445381635	1.681055004
Stearate Biosynthesis I (Animals)	0	0	1.661204587
Urea Cycle	0	0	1.653274348
LXR/RXR Activation	0	0	1.613185306
Mitochondrial L-carnitine Shuttle Pathway	0	0	1.597695673
γ-linolenate Biosynthesis II (Animals)	0	0	1.597695673
Phagosome Maturation	0.253867712	0	1.597348572
Fatty Acid β-oxidation I	0	0	1.541744375
Oleate Biosynthesis II (Animals)	0	0	1.518882168
Orexin Signaling Pathway	0	0	1.513778739
Integrin Signaling	1.502680394	13.50847144	1.255664255

Pulmonary Fibrosis Idiopathic Signaling Pathway	2.679247437	26.053347	1.253955813
Prolactin Signaling	1.778274044	9.301886041	1.132922051
PAK Signaling	1.558928095	7.57226506	1.117607322
Cardiac Hypertrophy Signaling (Enhanced)	2.066328424	16.55310958	0.975392727
Oxytocin Signaling Pathway	2.434656036	6.29534032	0.927790365
PPARα/RXRα Activation	1.659049029	8.113147301	0.775000195
Wound Healing Signaling Pathway	2.052108835	16.21206501	0.660466683
Regulation of Actin-based Motility by Rho	1.622969442	4.761369053	0.616395149
Semaphorin Neuronal Repulsive Signaling Pathway	2.077292414	6.754190836	0.526562827
Pulmonary Healing Signaling Pathway	1.627507027	11.68912813	0.499203109
Axonal Guidance Signaling	1.786089188	14.91372778	0.497474557
CCR3 Signaling in Eosinophils	2.208536587	5.337922298	0.44941257
Adrenomedullin signaling pathway	1.619758465	2.72880986	0.352507221
RHOA Signaling	1.548664005	4.488938532	0.310892065
Actin Cytoskeleton Signaling	3.467487573	13.32972491	0.309479938
TGF-β Signaling	1.778274044	9.301886041	0.307925082
Epithelial Adherens Junction Signaling	4.43039811	9.249230763	0
BMP signaling pathway	1.902031208	8.174059965	0
G-Protein Coupled Receptor Signaling	2.214516955	6.482068334	0
SAPK/JNK Signaling	1.518530749	6.375454493	0
HIPPO signaling	2.79805325	2.464941025	0
Agrin Interactions at Neuromuscular Junction	2.185889965	1.974782348	0
CDC42 Signaling	0.665579643	9.39781707	1.498600323
Role of Osteoblasts, Osteoclasts and Chondrocytes in Rheumatoid Arthritis	0.476013225	17.09074072	1.497716997
ICOS-ICOSL Signaling in T Helper Cells	0.312002206	11.35011204	1.496826748
Role of NFAT in Regulation of the Immune Response	1.004542112	12.99268464	1.487998082
Death Receptor Signaling	0.364945357	2.855444299	1.474099412
IL-4 Signaling	1.077459207	12.77002622	1.463971918

PI3K Signaling in B Lymphocytes	1.339660153	10.93253411	1.463526299
IL-2 Signaling	0.515489894	9.010444254	1.461465499
VEGF Family Ligand-Receptor Interactions	1.112731059	4.956781608	1.460195235
Molecular Mechanisms of Cancer	0.950859736	16.12681029	1.440316508
Type I Diabetes Mellitus Signaling	0.30113586	3.837950058	1.410124688
Role of NFAT in Cardiac Hypertrophy	0	4.626844136	1.404645273
Regulation Of The Epithelial Mesenchymal Transition In Development Pathway	0	4.427834977	1.382136688
Mouse Embryonic Stem Cell Pluripotency	0.354706719	8.137717035	1.380567402
p53 Signaling	0.354706719	5.74377849	1.380567402
Role of PKR in Interferon Induction and Antiviral Response	0	3.191005191	1.368752168
MSP-RON Signaling In Cancer Cells Pathway	0.255986023	10.70701968	1.366447806
RAC Signaling	1.347800373	7.770072524	1.366447806
Renin-Angiotensin Signaling	0.814960411	5.096619437	1.348538646
Glycogen Degradation II	1.190660137	2.368217249	1.308143724
CTLA4 Signaling in Cytotoxic T Lymphocytes	0.544811065	7.50050652	1.298637919
MSP-RON Signaling Pathway	0	1.795682197	1.286921095
IL-13 Signaling Pathway	1.02120969	4.113359331	1.285195381
PDGF Signaling	0.39843278	11.39152656	1.262146266
Estrogen Receptor Signaling	0.718640794	3.573196331	1.250128201
HER-2 Signaling in Breast Cancer	0	7.322564983	1.247784269
Leptin Signaling in Obesity	0	2.408218077	1.237826217
Th1 and Th2 Activation Pathway	0.665579643	12.93501033	1.224350226
RANK Signaling in Osteoclasts	0	6.07167886	1.217387311
IL-6 Signaling	0.278516157	10.36565758	1.197429337
ID1 Signaling Pathway	0.521621309	9.561244455	1.193657983
Role of NANOG in Mammalian Embryonic Stem Cell Pluripotency	0.871422734	10.08795461	1.192441767
Crosstalk between Dendritic Cells and Natural Killer Cells	0	1.821564193	1.18825327

Role of IL-17A in Arthritis	0	2.424224448	1.16927845
Glycogen Degradation III	1.120681974	2.395563203	1.147257585
Macrophage Classical Activation Signaling Pathway	0	7.969226172	1.143045694
FcγRIIB Signaling in B Lymphocytes	0.446154342	3.393435174	1.140906567
PI3K/AKT Signaling	0	9.064981076	1.123612692
Apelin Cardiomyocyte Signaling Pathway	0.364945357	1.782857508	1.112799291
NF-κB Activation by Viruses	0.441454895	6.336206502	1.095654865
Osteoarthritis Pathway	0	18.75380788	1.094605942
Small Cell Lung Cancer Signaling	0.368454771	2.667403791	1.093055064
Th2 Pathway	0.298501225	11.20551761	1.082013889
Apelin Endothelial Signaling Pathway	0.727212565	6.512913071	1.079252717
Autoimmune Thyroid Disease Signaling	0	2.787585821	1.075334649
B Cell Receptor Signaling	0.555157647	13.86191722	1.07402779
Renal Cell Carcinoma Signaling	1.112731059	6.934111585	1.052377733
Phospholipase C Signaling	0.343614862	11.777674	1.041273186
Sperm Motility	0.430173711	3.719000403	0.997972255
Ephrin A Signaling	0.639145234	2.535842036	0.988366834
Role of IL-17F in Allergic Inflammatory Airway Diseases	0	2.003350784	0.988366834
Production of Nitric Oxide and Reactive Oxygen Species in Macrophages	0	4.389951415	0.968386559
CD28 Signaling in T Helper Cells	0.285790988	10.00159707	0.966227958
iNOS Signaling	0	3.510271438	0.961318796
IL-17 Signaling	0.241627666	8.787183759	0.95823904
CD40 Signaling	0	4.684157936	0.94793491
Relaxin Signaling	0.688382159	1.805800226	0.944075097
TEC Kinase Signaling	0.531400964	9.652670776	0.93133152
Telomerase Signaling	0.335443459	5.15133461	0.931014263
fMLP Signaling in Neutrophils	0.774760631	6.35560316	0.920482424
LPS-stimulated MAPK Signaling	0.402437045	5.919952488	0.91493362
IL-12 Signaling and Production in Macrophages	0	2.663439258	0.895591879
STAT3 Pathway	1.364311669	7.259235673	0.891183698
HMGB1 Signaling	0.23389071	8.940896961	0.889412733

Colorectal Cancer Metastasis Signaling	0	8.851494349	0.887209619
Melanoma Signaling	0.597594507	3.869427476	0.885379355
Graft-versus-Host Disease Signaling	0	2.256697176	0.885379355
eNOS Signaling	0.737406623	1.50412624	0.87688085
Systemic Lupus Erythematosus Signaling	1.083369188	3.749445716	0.866782706
HGF Signaling	0.273804424	14.45612577	0.86280349
Role of JAK2 in Hormone-like Cytokine Signaling	0.58234581	2.256697176	0.861672681
Glioma Invasiveness Signaling	0.47090249	5.609646142	0.845770541
Ceramide Signaling	0.383004635	4.194602683	0.845102893
Calcium Signaling	0	1.950619695	0.842934603
HIF1α Signaling	0	7.468146589	0.838817922
Communication between Innate and Adaptive Immune Cells	0	3.168398346	0.826834615
Antiproliferative Role of Somatostatin Receptor 2	1.185633794	2.213269376	0.826834615
Inhibition of Matrix Metalloproteases	0.766191558	4.133719224	0.826048044
Regulation of eIF4 and p70S6K Signaling	0.576633682	2.562754304	0.819699035
Cancer Drug Resistance By Drug Efflux	0.574983797	4.230930354	0.816462827
Activation of IRF by Cytosolic Pattern Recognition Receptors	0	1.795682197	0.816462827
Endothelin-1 Signaling	0.562197993	5.392597096	0.797059532
GNRH Signaling	0.548231855	3.541836904	0.797059532
Gαq Signaling	1.198124289	5.814937493	0.789029921
Adipogenesis pathway	0.722191138	3.617736977	0.770249985
Human Embryonic Stem Cell Pluripotency	0.544811065	12.7013143	0.764183267
Role of Hypercytokinemia/hyperchemokinemia in the Pathogenesis of Influenza	0	2.040839628	0.753708887
Macropinocytosis Signaling	0.441454895	9.889703224	0.738814525
Pyroptosis Signaling Pathway	0	2.729254535	0.738814525
Glioblastoma Multiforme Signaling	0.20432262	6.571822419	0.720878121
G Beta Gamma Signaling	0.293326189	2.388874465	0.70072056

Xenobiotic Metabolism General Signaling Pathway	0.276146787	3.313481445	0.676234264
Signaling by Rho Family GTPases	1.235476373	7.683473563	0.67490691
Pathogen Induced Cytokine Storm Signaling Pathway	0	14.19194481	0.674743212
Paxillin Signaling	0.919618242	8.502389502	0.666905472
Bladder Cancer Signaling	0.905430488	3.168597483	0.653902083
Ovarian Cancer Signaling	0.661145473	6.680422957	0.643811569
Gα12/13 Signaling	0.785941146	5.11259629	0.641060732
Oncostatin M Signaling	0.65733226	9.55150696	0.634021107
p70S6K Signaling	0.747807657	5.959367213	0.629735607
P2Y Purigenic Receptor Signaling Pathway	0.273804424	3.496621765	0.629735607
PEDF Signaling	1.05622972	8.233081358	0.618094947
Clathrin-mediated Endocytosis Signaling	0	3.353852946	0.608878191
Synaptogenesis Signaling Pathway	0	3.353781575	0.607303663
Gap Junction Signaling	1.042926759	4.140441236	0.600711313
NF-κB Signaling	0.551680604	11.66077535	0.582582106
Fc Epsilon RI Signaling	0.323423338	4.922781465	0.58102925
Reelin Signaling in Neurons	0	2.171345467	0.57594212
B Cell Development	0	2.112707765	0.567802125
T Helper Cell Differentiation	0	3.857065102	0.565882582
Sertoli Cell-Sertoli Cell Junction Signaling	0.518412749	5.740117213	0.561293675
CREB Signaling in Neurons	0.276980979	4.853530083	0.555083918
Xenobiotic Metabolism Signaling	0	2.648599803	0.550130792
Neutrophil Extracellular Trap Signaling Pathway	0	1.86517968	0.544492217
CXCR4 Signaling	1.151515044	5.245399697	0.511073542
RAR Activation	0	3.150257324	0.50921674
Iron homeostasis signaling pathway	0	1.777001163	0.506091232
ERK5 Signaling	0.450934044	7.364212656	0.473212701
14-3-3-mediated Signaling	0.785941146	2.885170284	0.46758809
VDR/RXR Activation	0	2.898644386	0.449779568
Role of Macrophages, Fibroblasts and Endothelial Cells in Rheumatoid Arthritis	0.603864353	14.93853994	0.447216383

G Protein Signaling Mediated by Tubby	0.427816633	2.231332229	0.438518689
Phagosome Formation	0.719191542	9.258705008	0.429432441
PPAR Signaling	0.344881198	6.530405966	0.424264215
Apoptosis Signaling	0.912480916	3.285297311	0.424264215
Role Of Osteoclasts In Rheumatoid Arthritis Signaling Pathway	0.324821188	18.37407097	0.421854362
Leukocyte Extravasation Signaling	0	10.65633827	0.397256304
MicroRNA Biogenesis Signaling Pathway	0.548231855	4.343653561	0.397256304
Regulation of Cellular Mechanics by Calpain Protease	0.414826877	4.67817401	0.396305078
4-1BB Signaling in T Lymphocytes	0	1.573756194	0.392390517
ERK/MAPK Signaling	0.473154455	6.488295745	0.375802269
IL-17A Signaling in Fibroblasts	0	1.885737553	0.367390056
p38 MAPK Signaling	0	2.635074831	0.35532271
Basal Cell Carcinoma Signaling	0	4.814903833	0.353201405
Sphingosine-1-phosphate Signaling	0	4.432058918	0.339871395
Hepatic Cholestasis	0	3.822598332	0.333852307
Th17 Activation Pathway	1.219544673	5.537960153	0.332742867
WNT/Ca+ pathway	0.492356299	2.839311288	0.332742867
Cholecystokinin/Gastrin-mediated Signaling	0.839347001	6.993082597	0.33239154
Thrombin Signaling	0.873090773	5.703628166	0.330327028
TREM1 Signaling	0	5.248574563	0.322988144
Protein Kinase A Signaling	1.208030674	7.344465615	0.321027398
Synaptic Long Term Potentiation	0.30113586	1.719185605	0.317905347
Cardiac Hypertrophy Signaling	0.778265134	5.284059388	0.316614851
Inflammasome pathway	0	3.968420488	0.295391933
HOTAIR Regulatory Pathway	0.247649084	6.360141436	0.294976631
Fcγ Receptor-mediated Phagocytosis in Macrophages and Monocytes	0	3.902152305	0.292774525
Glucocorticoid Receptor Signaling	0.260530447	3.673894207	0.289014541
IL-8 Signaling	0.948007444	10.45516886	0.286264971
Ephrin Receptor Signaling	0.973158738	9.224961415	0.286264971

Regulation of IL-2 Expression in Activated and Anergic T Lymphocytes	0.949073214	9.474355306	0.278362717
Endocannabinoid Developing Neuron Pathway	0.28333778	2.78862664	0.265787334
Dilated Cardiomyopathy Signaling Pathway	0.266934944	1.758773416	0.254092481
CSDE1 Signaling Pathway	0	2.787893157	0.223203239
Tumoricidal Function of Hepatic Natural Killer Cells	0	1.669638828	0.210783081
Hepatic Fibrosis / Hepatic Stellate Cell Activation	0.569355701	19.50220906	0
FAK Signaling	0.700498704	14.39254986	0
GP6 Signaling Pathway	0.82701813	12.32218732	0
Factors Promoting Cardiogenesis in Vertebrates	0.717219075	8.292139608	0
S100 Family Signaling Pathway	0	8.184925397	0
Actin Nucleation by ARP-WASP Complex	1.012756895	8.107807842	0
Germ Cell-Sertoli Cell Junction Signaling	0.603098344	7.240399457	0
Chemokine Signaling	0.450934044	6.803443977	0
Granulocyte Adhesion and Diapedesis	0	5.176998173	0
Hematopoiesis from Pluripotent Stem Cells	0	4.799063288	0
RHOGDI Signaling	0.928507655	4.678879008	0
Breast Cancer Regulation by Stathmin1	0	4.310720161	0
Role of WNT/GSK-3β Signaling in the Pathogenesis of Influenza	0	4.176772898	0
FAT10 Cancer Signaling Pathway	0.58234581	3.9458516	0
Primary Immunodeficiency Signaling	0	3.794593455	0
B Cell Activating Factor Signaling	0.648116238	3.642472859	0
Cardiomyocyte Differentiation via BMP Receptors	0.903717167	3.612911614	0
TNFR2 Signaling	0	3.525512206	0

Altered T Cell and B Cell Signaling in Rheumatoid Arthritis	0	3.519519811	0
Acute Phase Response Signaling	0.562197993	3.399774482	0
Toll-like Receptor Signaling	0	3.393435174	0
Semaphorin Signaling in Neurons	1.319356871	3.366377121	0
April Mediated Signaling	0.65733226	3.354471528	0
Agranulocyte Adhesion and Diapedesis	0	3.192025602	0
Antiproliferative Role of TOB in T Cell Signaling	0	2.801056233	0
Airway Pathology in Chronic Obstructive Pulmonary Disease	0.39060373	2.733161188	0
GADD45 Signaling	0.534081817	2.732693387	0
UVC-Induced MAPK Signaling	0.58234581	2.630721247	0
WNT/β-catenin Signaling	0.210834734	2.595255563	0
Opioid Signaling Pathway	0	2.590642331	0
Sumoylation Pathway	0	2.316685926	0
Ephrin B Signaling	1.163957852	2.292781737	0
CD27 Signaling in Lymphocytes	0	2.289859842	0
Inhibition of Angiogenesis by TSP1	0.753665184	2.283231212	0
BEX2 Signaling Pathway	0	2.204335115	0
Remodeling of Epithelial Adherens Junctions	0	2.203782349	0
DNA Methylation and Transcriptional Repression Signaling	0.361484913	2.189338423	0
Hereditary Breast Cancer Signaling	0.247649084	2.151718444	0
Vitamin-C Transport	0	2.141885854	0
IL-1 Signaling	0.368454771	2.115726661	0
CDK5 Signaling	0.851957063	2.106606601	0
ATM Signaling	0	2.080650165	0
Pyridoxal 5'-phosphate Salvage Pathway	1.231243716	2.054265676	0
α-Adrenergic Signaling	0.344881198	1.993955327	0
Hematopoiesis from Multipotent Stem Cells	0	1.975484451	0
PCP (Planar Cell Polarity) Pathway	0.547131418	1.959223339	0
TNFR1 Signaling	0.58234581	1.914233512	0
Dermatan Sulfate Biosynthesis	0	1.631706665	0

CCR5 Signaling in Macrophages	0.335443459	1.537773775	0
Netrin Signaling	3.300884253	0.916680908	0.627428467
Neurovascular Coupling Signaling Pathway	1.741968734	0.49445053	0.454684817
Salvage Pathways of Pyrimidine Ribonucleotides	1.691598613	0.987079661	0.258046648
Apelin Muscle Signaling Pathway	1.502578489	0.39437097	0
Oxytocin In Spinal Neurons Signaling Pathway	1.852035211	0	0
Pyrimidine Ribonucleotides Interconversion	1.701330715	0	0
Pyrimidine Ribonucleotides De Novo Biosynthesis	1.635915722	0	0
Immunogenic Cell Death Signaling Pathway	0.4322881	1.310396155	1.487311343
Glutaminergic Receptor Signaling Pathway (Enhanced)	0	0	1.429383583
Lanosterol Biosynthesis	0	0	1.390673278
Asparagine Biosynthesis I	0	0	1.390673278
L-cysteine Degradation II	0	0	1.390673278
Necroptosis Signaling Pathway	0	1.275230649	1.366447806
Ethanol Degradation IV	0	0.406549326	1.340864962
AMPK Signaling	1.32461107	1.022423446	1.156489432
Role of p14/p19ARF in Tumor Suppression	0	1.309680368	0.984703113
Role of RIG1-like Receptors in Antiviral Innate Immunity	0	1.486375359	0.85493165
Hypoxia Signaling in the Cardiovascular System	0	1.465579871	0.738814525
Apelin Cardiac Fibroblast Signaling Pathway	0.903717167	1.412465543	0.648341128
GDP-glucose Biosynthesis	1.230764701	1.324839458	0.548655069
Role of MAPK Signaling in Inhibiting the Pathogenesis of Influenza	0	1.33400493	0.510789
Circadian Rhythm Signaling	1.279018717	1.43887699	0.468939099
3-phosphoinositide Biosynthesis	0	1.355631981	0.29132265

Aryl Hydrocarbon Receptor Signaling	0	1.330287812	0.289043698
Role of BRCA1 in DNA Damage Response	0	1.446691875	0
Superpathway of D-myo-inositol (1,4,5)-trisphosphate Metabolism	0	1.412465543	0
1D-myo-inositol Hexakisphosphate Biosynthesis II (Mammalian)	0	1.395397874	0
D-myo-inositol (1,3,4)-trisphosphate Biosynthesis	0	1.395397874	0
Superpathway of Inositol Phosphate Compounds	0	1.37854485	0
Purine Ribonucleosides Degradation to Ribose-1-phosphate	1.325094731	1.236869948	0
Ceramide Biosynthesis	1.381794818	0.899448619	0
Sphingomyelin Metabolism	1.381794818	0	0
