# Supplementary material for: Nutrient restriction-activated Fra-2 promotes tumor progression via IGF1R in miR-15a downmodulated pancreatic ductal adenocarcinoma
Source: Signal Transduct Target Ther. 2024 Feb 12;9:31. doi: 10.1038/s41392-024-01740-4 (PMC10859382; doi:10.1038/s41392-024-01740-4)
Supplement: Supplementary file 3 — Supplementary Table 2 [file 41392_2024_1740_MOESM3_ESM.pdf]

AsPC-1 cell line

ID	Gene Symbol	Ns Avg (log2)	N-dep Avg (log2)	Fold Change	P-val	FDR P-val
TC0100009364.hg.1	CSF1	5.0	11.2	74.130880	9.55E-13	1.37E-08
TC0700007434.hg.1	IGFBP1	7.0	14.0	130.846800	1.34E-12	1.37E-08
TC2000007202.hg.1	ACSS2	9.9	15.7	56.966500	1.92E-12	1.37E-08
TC0100010389.hg.1	HSD17B7	8.7	13.9	38.000590	8.16E-12	4.38E-08
TC0400009221.hg.1	MSMO1	14.1	19.1	30.628700	2.65E-11	9.23E-08
TC1700010592.hg.1	ORMDL3	9.9	14.5	24.543990	3.01E-11	9.23E-08
TC0700011953.hg.1	CYP3A5	12.9	17.2	20.498930	3.88E-11	9.41E-08
TC0500010635.hg.1	HMGCS1	11.1	15.7	24.055350	3.95E-11	9.41E-08
TC1600011221.hg.1	MVD	8.8	13.5	25.414040	6.31E-11	9.45E-08
TC0200016403.hg.1	LPIN1; MIR548S	6.9	11.5	24.420180	6.50E-11	9.45E-08
TC0500012567.hg.1	FAXDC2	9.5	14.0	22.166360	7.46E-11	9.45E-08
TC0800007080.hg.1	BNIP3L	10.3	14.6	19.632970	8.32E-11	9.45E-08
TC0X00006799.hg.1	SAT1	6.7	11.1	20.619800	9.38E-11	9.45E-08
TC1000009536.hg.1	IDI1	9.7	14.4	26.844730	9.54E-11	9.45E-08
TC0200009955.hg.1	CYBRD1	11.1	15.2	17.291150	9.70E-11	9.45E-08
TC1400007320.hg.1	DACT1	5.8	10.0	17.962480	1.02E-10	9.45E-08
TC0900010439.hg.1	TRPM6	4.7	10.3	48.665760	1.12E-10	9.45E-08
TC1100011514.hg.1	DHCR7	12.9	16.9	16.326280	1.13E-10	9.45E-08
TC0100015234.hg.1	SLC16A4	9.7	13.8	16.964830	1.18E-10	9.45E-08
TC0600009459.hg.1	ENPP1	6.8	11.0	18.227440	1.21E-10	9.45E-08
TC0100011406.hg.1	CD55	13.0	17.0	16.019990	1.23E-10	9.45E-08
TC0100018508.hg.1	ARHGEF2	8.6	12.7	17.363060	1.23E-10	9.45E-08
TC0400012818.hg.1	CCNG2	7.1	11.3	18.541360	1.50E-10	9.98E-08
TC1800008285.hg.1	NPC1	9.6	13.7	17.324630	1.59E-10	9.98E-08
TC0200014672.hg.1	NR4A2	5.2	9.8	23.891620	1.65E-10	9.98E-08
TC1200012643.hg.1	ERBB3	12.3	16.4	17.409730	1.71E-10	9.98E-08
TC0700011954.hg.1	CYP3A7; CYP3A7-	4.8	9.6	27.716990	2.29E-10	1.20E-07
TC0700011842.hg.1	PDK4	6.7	11.3	22.797140	2.45E-10	1.21E-07
TC1900007012.hg.1	LDLR; MIR6886	12.5	16.2	12.808680	2.48E-10	1.21E-07
TC0700008747.hg.1	HBP1	9.5	13.2	13.002650	2.49E-10	1.21E-07
TC1000006816.hg.1	OPTN	8.2	12.2	15.968970	2.55E-10	1.21E-07
TC0100018451.hg.1	GBP2	5.3	10.2	30.689120	2.66E-10	1.24E-07
TC0500011702.hg.1	STARD4	8.9	13.3	21.528830	2.86E-10	1.27E-07
TC0700007993.hg.1	CLDN4	11.9	15.8	14.948640	2.89E-10	1.27E-07
TC0500009488.hg.1	CREBRF	6.9	10.6	13.690740	2.98E-10	1.27E-07
TC0700009834.hg.1	LOC389602; AC02	7.8	11.4	12.647220	3.13E-10	1.27E-07
TC0600013010.hg.1	MAN1A1	9.1	12.9	14.246050	3.21E-10	1.27E-07
TC1100009306.hg.1	SC5D	7.5	11.6	17.465230	3.53E-10	1.31E-07
TC0300012236.hg.1	MUC13	11.0	14.7	13.312380	3.56E-10	1.31E-07
TC0700012038.hg.1	MOGAT3	7.3	11.3	15.773990	3.69E-10	1.31E-07
TC1200011246.hg.1	PTPRR	4.6	8.4	13.901810	3.75E-10	1.31E-07
TC1200010812.hg.1	CALCOCO1	7.1	11.3	17.817930	3.82E-10	1.31E-07
TC0900011850.hg.1	SLC2A6	9.9	13.6	13.067520	3.85E-10	1.31E-07
TC0300009673.hg.1	KLHL24	5.8	10.4	23.732150	4.14E-10	1.34E-07
TC0800011881.hg.1	NDRG1	7.2	11.4	18.299800	4.35E-10	1.35E-07
TC0500007804.hg.1	HMGCR	11.0	14.6	12.090550	4.51E-10	1.38E-07
TC1500006925.hg.1	THBS1	8.0	12.4	21.809980	4.63E-10	1.38E-07

TC0200007132.hg.1	YPEL5	6.7	11.6	29.299540	5.07E-10	1.48E-07
TC1600008577.hg.1	BCO1	4.8	9.4	23.735330	5.09E-10	1.48E-07
TC0600008109.hg.1	VEGFA	8.8	12.7	15.352150	5.20E-10	1.49E-07
TC0200016471.hg.1	MXD1	8.0	11.4	10.534800	5.26E-10	1.49E-07
TC1200008726.hg.1	TCP11L2	5.2	9.5	19.948020	5.63E-10	1.53E-07
TC0100015598.hg.1	TXNIP	11.6	15.2	11.883260	5.64E-10	1.53E-07
TC0X00010473.hg.1	TSC22D3	4.6	9.0	21.080690	5.83E-10	1.56E-07
TC0300013417.hg.1	LIPH	12.4	16.0	12.030450	6.02E-10	1.57E-07
TC0700009807.hg.1	INSIG1	7.9	12.4	23.293320	6.13E-10	1.58E-07
TC1900011654.hg.1	PNPLA6	11.3	14.7	10.530280	6.37E-10	1.61E-07
TC1400007259.hg.1	PELI2	3.9	8.8	30.058690	6.38E-10	1.61E-07
TC0600008757.hg.1	PNRC1	6.0	10.3	19.447750	6.75E-10	1.66E-07
TC1400007321.hg.1	DACT1	5.2	9.5	18.880710	7.18E-10	1.75E-07
TC1700010200.hg.1	ALDOC	7.9	11.6	12.637100	7.46E-10	1.78E-07
TC0900011300.hg.1	TNFSF15	8.7	12.1	10.711340	7.55E-10	1.78E-07
TC0300012720.hg.1	PLSCR4	5.9	10.0	17.817200	8.00E-10	1.81E-07
TC1200012753.hg.1	KLRC2	8.0	11.7	12.905180	8.70E-10	1.94E-07
TC0100010111.hg.1	EFNA1	5.7	10.3	24.804270	8.99E-10	1.96E-07
TC1700008232.hg.1	PDK2	8.1	11.7	11.420020	9.16E-10	1.96E-07
TC2200009356.hg.1	ARFGAP3	7.8	11.8	15.635940	9.63E-10	2.01E-07
TC1900010625.hg.1	LGALS4	11.8	15.1	10.373850	9.98E-10	2.04E-07
TC0500012294.hg.1	PCDH1	10.2	13.8	11.886320	1.02E-09	2.07E-07
TC1700010590.hg.1	GSDMB	5.2	9.8	24.455430	1.09E-09	2.11E-07
TC0200011097.hg.1	ALPPL2	9.4	13.4	15.851340	1.13E-09	2.16E-07
TC0800010417.hg.1	PCMTD1	10.1	13.5	10.197670	1.17E-09	2.21E-07
TC2000009242.hg.1	WFDC3	7.4	11.5	17.162520	1.18E-09	2.22E-07
TC2000008587.hg.1	RALGAPA2	8.9	12.2	9.708480	1.19E-09	2.22E-07
TC0700013051.hg.1	ATG9B	5.8	10.3	23.051330	1.23E-09	2.24E-07
TC2000007502.hg.1	DNTTIP1	11.1	14.4	10.027100	1.37E-09	2.34E-07
TC1900006678.hg.1	CREB3L3	11.2	14.6	10.914690	1.39E-09	2.36E-07
TC0400012621.hg.1	ACSL1	3.9	8.1	18.618500	1.40E-09	2.36E-07
TC1100009514.hg.1	TMEM45B	11.9	15.1	9.737219	1.41E-09	2.36E-07
TC1300009766.hg.1	IRS2	6.9	10.3	10.593660	1.49E-09	2.43E-07
TC1800006889.hg.1	RIOK3	10.2	13.8	12.442910	1.51E-09	2.43E-07
TC0X00009650.hg.1	WDR45; PRAF2	6.1	10.8	27.139260	1.76E-09	2.64E-07
TC1700010641.hg.1	KRT23	7.5	11.6	17.345420	1.76E-09	2.64E-07
TC1700010891.hg.1	PLEKHM1; MIR431	7.3	10.5	9.143304	1.79E-09	2.64E-07
TC0700009411.hg.1	MGAM2	14.0	17.5	11.097950	1.79E-09	2.64E-07
TC1200012708.hg.1	OAS1	10.7	14.0	9.424960	1.80E-09	2.64E-07
TC0800008783.hg.1	SQLE	8.9	12.8	15.020680	1.82E-09	2.64E-07
TC0100013314.hg.1	IL22RA1	10.7	14.3	11.433770	1.85E-09	2.66E-07
TC1200008133.hg.1	LYZ	12.0	15.1	8.739343	1.90E-09	2.68E-07
TSUnmapped0000012	SLC2A6	8.2	11.6	10.741460	1.91E-09	2.68E-07
TC0700006844.hg.1	ITGB8	11.8	14.9	8.443543	2.02E-09	2.76E-07
TC0600014106.hg.1	CFB	5.0	8.7	13.340660	2.02E-09	2.76E-07
TC1400010390.hg.1	AHNAK2	13.1	16.2	8.636793	2.14E-09	2.87E-07
TC0900011829.hg.1	RALGDS	8.2	11.5	9.563032	2.22E-09	2.95E-07
TC1400006663.hg.1	LRP10	10.7	13.8	8.570450	2.30E-09	3.03E-07
TC0500009594.hg.1	CDHR2	9.6	13.1	11.795550	2.31E-09	3.03E-07
TSUnmapped0000005	SLC2A6	10.5	13.5	8.332343	2.33E-09	3.03E-07

TC0100008417.hg.1	PCSK9	5.7	9.3	11.573920	2.34E-09	3.03E-07
TC0600010057.hg.1	ACAT2	9.0	12.1	8.172392	2.50E-09	3.17E-07
TC1900011679.hg.1	CYP4F3	6.4	10.1	13.007780	2.66E-09	3.35E-07
TC0200016704.hg.1	RNF103	9.5	12.7	9.123174	2.70E-09	3.36E-07
TC0100008145.hg.1	TSPAN1	10.5	13.5	7.963605	2.74E-09	3.40E-07
TC2200009281.hg.1	PNPLA3	4.1	7.6	11.554570	2.79E-09	3.41E-07
TC0300013146.hg.1	TNFSF10	5.1	9.2	17.146730	2.80E-09	3.41E-07
TC1500010369.hg.1	MFGE8	8.8	12.0	8.816379	2.82E-09	3.41E-07
TC0100015629.hg.1	FMO5	6.0	10.3	19.039130	2.88E-09	3.47E-07
TC0200016742.hg.1	PLA2R1	10.6	13.5	7.463780	2.90E-09	3.47E-07
TC0700013584.hg.1	CYP51A1	12.9	16.1	9.341736	2.92E-09	3.47E-07
TC0400008137.hg.1	CCSER1	4.3	7.1	7.271475	3.18E-09	3.73E-07
TC0200015194.hg.1	TFPI	12.3	15.4	8.642335	3.41E-09	3.93E-07
TC1200006787.hg.1	GABARAPL1	6.4	10.1	13.479310	3.45E-09	3.96E-07
TC0100014617.hg.1	SLC44A5	12.1	15.0	7.623718	3.63E-09	4.14E-07
TC0600008622.hg.1	SH3BGRL2	8.5	11.7	8.614698	4.21E-09	4.65E-07
TC1100007776.hg.1	MS4A8	4.9	8.4	10.999170	4.45E-09	4.75E-07
TC0700008577.hg.1	MUC17	11.1	14.1	7.938247	4.47E-09	4.75E-07
TC0500007681.hg.1	OCLN	11.9	14.7	7.280622	4.48E-09	4.75E-07
TC0X00011121.hg.1	GABRE; MIR224; M	12.5	15.4	7.751921	4.81E-09	5.04E-07
TC1800007905.hg.1	LPIN2	8.5	11.4	7.131381	4.82E-09	5.04E-07
TC0800009997.hg.1	KIF13B	12.2	15.0	7.335217	5.14E-09	5.29E-07
TC1900010807.hg.1	CEACAM1	15.3	18.6	9.696379	5.20E-09	5.31E-07
TC0600007862.hg.1	PIM1	11.5	14.5	8.094398	5.24E-09	5.32E-07
TC0X00009981.hg.1	IL2RG	7.2	10.3	8.847775	5.28E-09	5.32E-07
TC1900011464.hg.1	TMEM150B	9.3	12.4	8.432215	5.30E-09	5.32E-07
TC1600008580.hg.1	GAN; MIR4720	9.7	12.6	7.565108	5.31E-09	5.32E-07
TC1400008752.hg.1	CIDEB	7.4	10.5	8.628934	5.41E-09	5.36E-07
TC0600012240.hg.1	LMBRD1	9.6	12.5	7.440080	5.43E-09	5.36E-07
TC0200009219.hg.1	MYO7B	4.6	9.9	39.109440	5.45E-09	5.36E-07
TC0600014196.hg.1	SNX9	10.9	13.8	7.254393	5.47E-09	5.36E-07
TC0200009470.hg.1	HNMT	9.8	12.7	7.557349	5.54E-09	5.40E-07
TC1100011833.hg.1	SYTL2	8.8	12.1	9.563725	5.85E-09	5.61E-07
TC0200009067.hg.1	TMEM37	11.2	14.1	7.192953	6.10E-09	5.74E-07
TC1600008607.hg.1	PLCG2	6.4	9.3	7.208450	6.15E-09	5.76E-07
TC1100008523.hg.1	CAPN5	6.8	9.9	9.027175	6.23E-09	5.81E-07
TC1900011681.hg.1	CYP4F12	6.2	9.7	10.680000	6.39E-09	5.89E-07
TC0200007181.hg.1	YIPF4	9.8	12.5	6.742554	6.49E-09	5.94E-07
TC0100018555.hg.1	LEMD1	4.3	7.6	9.781609	6.51E-09	5.94E-07
TC1700010565.hg.1	FBXL20	6.4	9.8	10.229810	6.65E-09	6.04E-07
TC0200016581.hg.1	C2orf88	7.3	10.8	11.591910	6.74E-09	6.07E-07
TC1700007319.hg.1	WSB1	12.3	15.0	6.545938	6.79E-09	6.07E-07
TC0500010615.hg.1	SEPP1	5.0	10.1	34.883380	6.85E-09	6.08E-07
TC1900009893.hg.1	CYP4F2	5.6	8.5	7.693231	6.86E-09	6.08E-07
TC1100008790.hg.1	C11orf54	8.4	11.2	6.942582	7.04E-09	6.17E-07
TC1100007394.hg.1	CD82	12.8	15.5	6.647089	7.08E-09	6.17E-07
TC1100006966.hg.1	NCR3LG1	8.4	11.5	8.681766	7.21E-09	6.26E-07
TC0600009364.hg.1	NCOA7	7.2	10.0	7.084766	7.34E-09	6.35E-07
TC0800006760.hg.1	FDFT1	13.3	16.1	6.799954	7.45E-09	6.39E-07
TC0100010672.hg.1	RABGAP1L	7.6	10.7	8.574688	7.70E-09	6.52E-07

TC0100016963.hg.1	KDM5B	10.4	13.1	6.712245	8.15E-09	6.80E-07
TC1100008453.hg.1	SLCO2B1	4.6	7.4	6.907707	8.28E-09	6.88E-07
TC0200015790.hg.1	CHPF	9.0	12.5	11.077560	8.36E-09	6.92E-07
TC0400009765.hg.1	MXD4; MIR4800	7.2	10.1	7.337449	8.58E-09	7.08E-07
TC2000009953.hg.1	OSBPL2	10.1	12.8	6.426406	8.88E-09	7.26E-07
TC0400012829.hg.1	ARHGEF38	6.2	9.1	7.269927	9.04E-09	7.35E-07
TC1200012752.hg.1	KLRC3	6.7	9.9	8.921787	9.27E-09	7.47E-07
TC0300013471.hg.1	BCL6	10.1	12.8	6.246441	9.33E-09	7.48E-07
TSUnmapped0000000	SLC2A6	6.5	9.4	7.275347	9.38E-09	7.48E-07
TC0300008563.hg.1	DIRC2	8.0	11.1	9.087912	9.53E-09	7.54E-07
TC0600008569.hg.1	MYO6	8.5	11.2	6.874122	9.81E-09	7.65E-07
TC0900009978.hg.1	GNE	14.2	16.9	6.728672	9.84E-09	7.65E-07
TC1600009952.hg.1	YPEL3	6.8	11.9	33.158280	9.90E-09	7.66E-07
TC2100008496.hg.1	SLC5A3; MRPS6	8.1	10.7	6.225892	1.01E-08	7.73E-07
TC0200011219.hg.1	ACKR3	9.8	12.8	8.014189	1.03E-08	7.92E-07
TC1900009896.hg.1	CYP4F11	4.1	7.1	8.002799	1.05E-08	8.00E-07
TC1900011741.hg.1	CEACAM5	14.0	16.7	6.570423	1.11E-08	8.31E-07
TC0100016296.hg.1	GPA33	11.9	14.5	6.419229	1.16E-08	8.64E-07
TC0400010282.hg.1	SEL1L3	7.5	10.3	6.896959	1.17E-08	8.68E-07
TC0800007040.hg.1	ADAM28	4.5	8.7	18.893340	1.18E-08	8.72E-07
TC1600011375.hg.1	QPRT	8.1	11.3	9.800739	1.19E-08	8.73E-07
TC1100012992.hg.1	HSD17B12	12.3	14.9	6.058370	1.20E-08	8.73E-07
TC1100013040.hg.1	TM7SF2	8.4	11.5	8.664443	1.22E-08	8.85E-07
TC0100011419.hg.1	CD46	12.3	15.0	6.304184	1.26E-08	9.07E-07
TC0600012885.hg.1	LAMA4	10.1	13.0	7.715816	1.31E-08	9.35E-07
TC0100018006.hg.1	ADSS	11.1	13.6	5.779712	1.38E-08	9.74E-07
TC1000008881.hg.1	MXI1	7.4	10.6	9.147252	1.39E-08	9.83E-07
TC1000007990.hg.1	DDIT4	8.0	11.0	8.108987	1.41E-08	9.90E-07
TC1900008141.hg.1	CYP2S1	14.6	17.5	7.492845	1.44E-08	1.00E-06
TC0700013396.hg.1	CLDN12	10.6	13.1	5.647001	1.46E-08	1.02E-06
TC0X00011404.hg.1	IDS	9.7	12.5	6.912077	1.46E-08	1.02E-06
TC0900008953.hg.1	ASS1	4.6	7.6	7.831979	1.50E-08	1.03E-06
TC0700011982.hg.1	GATS	8.3	11.0	6.554220	1.50E-08	1.03E-06
TC0200015721.hg.1	TMBIM1; MIR6513	10.9	13.6	6.716405	1.52E-08	1.04E-06
TC1500010042.hg.1	CYP1A1	4.6	7.5	7.311316	1.53E-08	1.05E-06
TC0X00008481.hg.1	FAM122C	7.0	10.0	8.201242	1.62E-08	1.10E-06
TC1500006972.hg.1	SPINT1	9.0	11.9	7.931506	1.65E-08	1.12E-06
TC0900007552.hg.1	GDA	10.4	13.0	5.927421	1.79E-08	1.19E-06
TC2000008381.hg.1	JAG1	11.1	13.7	6.002571	1.82E-08	1.21E-06
TC1700010358.hg.1	MYO1D	11.5	13.9	5.488263	1.83E-08	1.21E-06
TC0300006655.hg.1	HDAC11	5.0	7.8	7.050830	1.83E-08	1.21E-06
TC0600008571.hg.1	MYO6	7.3	10.3	7.941891	1.91E-08	1.25E-06
TC0200010511.hg.1	NBEAL1	8.7	11.1	5.190211	1.95E-08	1.28E-06
TC0800011601.hg.1	SAMD12	7.8	10.3	5.531808	1.96E-08	1.28E-06
TC0100010112.hg.1	SLC50A1	7.9	10.7	6.723547	2.02E-08	1.30E-06
TC2200008475.hg.1	PIK3IP1	5.0	8.3	9.629022	2.06E-08	1.32E-06
TC1400010765.hg.1	RDH11	8.7	11.2	5.706440	2.07E-08	1.32E-06
TC1700011436.hg.1	ERN1	8.3	10.9	5.875402	2.09E-08	1.32E-06
TC1900012021.hg.1	ZNF841	7.2	9.8	5.761214	2.14E-08	1.34E-06
TC0200016717.hg.1	TBC1D8	9.4	12.4	8.185727	2.14E-08	1.34E-06

TC1700007942.hg.1	NBR1	10.1	12.6	5.635673	2.20E-08	1.36E-06
TC2100008462.hg.1	LSS	9.2	11.8	6.395682	2.22E-08	1.37E-06
TC1300009765.hg.1	IRS2	8.2	11.2	8.425683	2.27E-08	1.39E-06
TC0300006483.hg.1	BHLHE40	12.3	15.2	7.635242	2.29E-08	1.39E-06
TC2000006446.hg.1	RBCK1	8.7	11.9	9.360037	2.34E-08	1.41E-06
TC1500007379.hg.1	FAM63B	6.6	9.3	6.477651	2.38E-08	1.43E-06
TC0200016582.hg.1	NABP1	9.1	11.5	5.318823	2.39E-08	1.43E-06
TC0300006580.hg.1	IRAK2	7.8	10.4	5.807754	2.43E-08	1.44E-06
TC0300011899.hg.1	CBLB	7.7	11.0	9.993711	2.43E-08	1.44E-06
TC0100009195.hg.1	SLC35A3	12.6	15.0	5.498168	2.45E-08	1.44E-06
TC0400008609.hg.1	KIAA1109	9.2	11.7	5.541590	2.50E-08	1.46E-06
TC0500012842.hg.1	DUSP1	4.9	7.6	6.874669	2.50E-08	1.46E-06
TC0300012170.hg.1	GOLGB1	8.5	10.9	5.209715	2.58E-08	1.50E-06
TC1500010106.hg.1	TSPAN3	10.5	13.0	5.537889	2.59E-08	1.50E-06
TC1100008010.hg.1	CDC42EP2	7.7	10.5	6.783309	2.59E-08	1.50E-06
TC0400011920.hg.1	SLC7A11	14.0	16.5	5.751793	2.65E-08	1.52E-06
TC0600013757.hg.1	SOD2	11.2	13.8	5.901479	2.69E-08	1.54E-06
TC0100018507.hg.1	ARHGEF2	13.2	15.8	5.920295	2.74E-08	1.57E-06
TC1400010071.hg.1	CLMN	8.8	11.7	7.445642	2.78E-08	1.59E-06
TC0500007549.hg.1	ZSWIM6	8.5	11.3	6.923598	2.85E-08	1.62E-06
TC1500008986.hg.1	LPCAT4	8.3	11.5	9.350965	2.91E-08	1.64E-06
TC1900007358.hg.1	ARRDC2	8.4	10.8	5.178810	2.99E-08	1.68E-06
TC0600011351.hg.1	TRIM31	7.5	10.8	9.418107	3.04E-08	1.70E-06
TC0800012026.hg.1	SLC45A4	8.6	11.0	5.377227	3.17E-08	1.76E-06
TC0200008536.hg.1	ANKRD36	9.9	12.3	5.291094	3.21E-08	1.78E-06
TC0100013072.hg.1	ATP13A2	9.5	12.6	8.798351	3.32E-08	1.82E-06
TC1300009980.hg.1	LMO7	12.4	15.1	6.374720	3.53E-08	1.91E-06
TC1200008803.hg.1	MVK	6.9	9.4	5.728354	3.56E-08	1.92E-06
TC0800007957.hg.1	RDH10	11.3	13.9	6.345765	3.57E-08	1.92E-06
TC1700012191.hg.1	CD68	11.3	13.7	5.260032	3.67E-08	1.96E-06
TC1500007409.hg.1	GCNT3	14.4	16.8	5.410266	3.68E-08	1.96E-06
TC0100018166.hg.1	CLCN6	8.2	10.6	5.332837	3.69E-08	1.96E-06
TC0100018210.hg.1	SH3D21	8.7	11.8	8.325442	3.72E-08	1.97E-06
TC0200009739.hg.1	GALNT5	10.9	13.2	4.842435	3.73E-08	1.97E-06
TC1700007997.hg.1	GRN	11.5	13.9	5.253154	3.76E-08	1.98E-06
TC0700013382.hg.1	KCTD7	7.7	10.0	4.846740	3.78E-08	1.99E-06
TC0800010506.hg.1	PLAG1	7.1	10.2	8.571176	3.81E-08	2.00E-06
TC1100007507.hg.1	PTPRJ	9.4	11.8	5.136999	4.02E-08	2.10E-06
TC1900012051.hg.1	COX6B2	4.7	7.3	6.105714	4.07E-08	2.10E-06
TC1100013093.hg.1	BCO2; RPS12P21	9.6	12.1	5.816012	4.07E-08	2.10E-06
TC0700013254.hg.1	PTPRN2	7.6	10.2	5.981065	4.17E-08	2.14E-06
TC0200011093.hg.1	ALPP	7.6	10.7	8.352464	4.22E-08	2.15E-06
TC0700008786.hg.1	DNAJB9	6.0	9.3	9.895514	4.24E-08	2.16E-06
TC0100014774.hg.1	C1orf52	8.6	11.0	5.279259	4.26E-08	2.17E-06
TC0700009680.hg.1	TMEM176A	8.4	11.0	6.201100	4.29E-08	2.17E-06
TC0600013186.hg.1	CTGF	6.7	10.5	14.019340	4.33E-08	2.18E-06
TC0100015716.hg.1	MTMR11	10.9	13.9	7.736714	4.39E-08	2.19E-06
TC2000007015.hg.1	PYGB	11.0	13.4	5.120845	4.43E-08	2.21E-06
TC1700007757.hg.1	GRB7	8.3	10.8	5.952888	4.52E-08	2.24E-06
TC2200007495.hg.1	SREBF2	9.8	12.5	6.208178	4.62E-08	2.28E-06

TC0400011144.hg.1	ANTXR2	13.5	15.8	4.897089	4.66E-08	2.28E-06
TC1200009587.hg.1	CACNA2D4	8.1	11.0	7.872010	4.66E-08	2.28E-06
TC1500010723.hg.1	CHAC1	4.8	8.2	10.505670	4.69E-08	2.29E-06
TC0300008561.hg.1	PARP14	9.8	12.6	7.160340	4.80E-08	2.33E-06
TC2100008490.hg.1	BACH1	13.0	15.4	5.388620	4.80E-08	2.33E-06
TC1100013022.hg.1	FADS2	13.5	16.2	6.584128	4.81E-08	2.33E-06
TC0300008371.hg.1	GRAMD1C	4.1	7.6	10.933960	4.95E-08	2.37E-06
TC0800008002.hg.1	HNF4G	10.6	13.0	5.127018	5.04E-08	2.39E-06
TC1400006659.hg.1	MMP14	6.5	9.1	6.271212	5.26E-08	2.47E-06
TC1900011858.hg.1	ZNF846	5.5	8.3	6.935323	5.32E-08	2.50E-06
TC1200009457.hg.1	ULK1	6.2	8.5	5.108947	5.41E-08	2.53E-06
TC0300012943.hg.1	LXN	8.5	11.3	6.889940	5.48E-08	2.55E-06
TC1200009103.hg.1	PRKAB1	10.3	12.7	5.258242	5.49E-08	2.55E-06
TC0500010549.hg.1	RICTOR	11.1	13.3	4.603240	5.53E-08	2.56E-06
TC1800007198.hg.1	SLC14A1	6.2	9.0	7.009243	5.53E-08	2.56E-06
TC0800009868.hg.1	TNFRSF10B	10.3	12.6	4.773054	5.74E-08	2.65E-06
TC0100015194.hg.1	SORT1	7.9	10.2	4.944427	5.82E-08	2.67E-06
TC0100017167.hg.1	LAMB3; MIR4260	10.4	12.9	5.569744	5.84E-08	2.67E-06
TC1700011117.hg.1	LINC00483	7.7	10.0	4.997142	6.08E-08	2.78E-06
TC0100014250.hg.1	TMEM59	10.1	12.4	4.931406	6.30E-08	2.87E-06
TC0700008181.hg.1	GNAI1	12.7	15.2	5.449426	6.38E-08	2.89E-06
TC0600013144.hg.1	C6orf191andARHG	6.4	9.2	6.716938	6.43E-08	2.91E-06
TC0300012323.hg.1	MGLL	7.7	10.0	4.856483	6.51E-08	2.94E-06
TC1100009686.hg.1	CDHR5	6.4	9.2	7.063159	6.66E-08	2.99E-06
TC1600006561.hg.1	MAPK8IP3	8.7	11.1	5.102658	6.87E-08	3.07E-06
TC0100015771.hg.1	SEMA6C	3.6	6.5	7.409543	6.94E-08	3.09E-06
TC1200011251.hg.1	TSPAN8	11.0	13.5	5.734556	6.99E-08	3.11E-06
TC0200010800.hg.1	VIL1	10.0	12.4	5.044082	7.02E-08	3.12E-06
TC1000008643.hg.1	SCD	15.7	18.1	5.083519	7.22E-08	3.19E-06
TC0300009139.hg.1	TM4SF4	13.4	15.6	4.715790	7.34E-08	3.22E-06
TC1100011192.hg.1	ATG2A	9.5	11.8	4.950852	7.52E-08	3.28E-06
TC1700009498.hg.1	ATP2A3	7.8	9.9	4.460254	7.53E-08	3.28E-06
TC2000006444.hg.1	TRIB3	6.3	10.2	14.236000	7.58E-08	3.29E-06
TC0700013605.hg.1	PNPLA8	6.5	9.0	5.774399	7.71E-08	3.33E-06
TC0200010907.hg.1	ACSL3	10.9	13.3	5.117226	8.06E-08	3.46E-06
TC0600011536.hg.1	TAPBP	12.9	15.0	4.399911	8.22E-08	3.51E-06
TC0700010751.hg.1	AOAH	5.0	8.6	11.944590	8.26E-08	3.51E-06
TC0200010837.hg.1	ANKZF1	8.2	11.3	8.567138	8.29E-08	3.51E-06
TC0100012097.hg.1	LGALS8	8.3	10.6	5.135236	8.30E-08	3.51E-06
TC0100015752.hg.1	CTSS	4.2	6.4	4.774490	8.31E-08	3.51E-06
TC0400006578.hg.1	SH3BP2	11.0	13.2	4.511953	8.33E-08	3.52E-06
TC2000009956.hg.1	PCMTD2	9.4	11.5	4.420160	8.41E-08	3.55E-06
TC0100014849.hg.1	GBP3	9.0	11.8	7.420203	8.45E-08	3.55E-06
TC0800011683.hg.1	FBXO32	4.3	7.3	8.072709	8.69E-08	3.62E-06
TC0700013047.hg.1	TMEM176B	9.0	11.4	5.197061	8.73E-08	3.63E-06
TC0300013812.hg.1	TRAK1	9.3	11.4	4.253804	8.74E-08	3.63E-06
TC0500007881.hg.1	JMY	7.1	9.4	4.728515	8.83E-08	3.66E-06
TC0200015578.hg.1	IDH1	11.1	13.5	5.094901	8.92E-08	3.68E-06
TC2000009461.hg.1	ATP9A	10.1	12.8	6.394389	8.92E-08	3.68E-06
TC0200010443.hg.1	CFLAR	14.6	16.7	4.314806	9.08E-08	3.73E-06

TC1400010584.hg.1	IRF9	7.1	9.2	4.305742	9.35E-08	3.81E-06
TC0700013441.hg.1	IFRD1	10.8	13.3	5.403738	9.39E-08	3.82E-06
TC1700007360.hg.1	TMEM97	11.3	13.5	4.771446	9.60E-08	3.90E-06
TC1900012027.hg.1	ZNF320	3.4	6.9	11.129480	9.62E-08	3.90E-06
TC0100011533.hg.1	ATF3	4.0	6.7	6.409620	9.70E-08	3.92E-06
TC1500010788.hg.1	CHD2; MIR3175	9.2	11.3	4.250081	9.77E-08	3.94E-06
TC0100017449.hg.1	CAPN8	8.8	10.8	4.116307	9.95E-08	4.00E-06
TC0800011150.hg.1	TP53INP1	3.3	7.4	17.223780	1.01E-07	4.02E-06
TC1400009337.hg.1	DHRS7	6.3	8.5	4.397893	1.02E-07	4.08E-06
TC0900008811.hg.1	STXBP1	10.3	12.6	5.050444	1.03E-07	4.09E-06
TC1200011495.hg.1	LUM	8.8	11.1	5.073535	1.04E-07	4.12E-06
TC1000010831.hg.1	HERC4	11.6	13.6	4.134266	1.05E-07	4.16E-06
TC0100018510.hg.1	GLMP	8.5	10.5	4.125387	1.08E-07	4.26E-06
TC0300013855.hg.1	NFKBIZ	9.8	12.6	6.805566	1.11E-07	4.34E-06
TC0500008785.hg.1	EGR1	9.9	12.4	5.684297	1.11E-07	4.34E-06
TC1700009861.hg.1	TVP23C; CDRT4; T	9.5	11.6	4.269452	1.13E-07	4.40E-06
TC0500009706.hg.1	SQSTM1	13.0	15.1	4.324957	1.14E-07	4.42E-06
TC1000012561.hg.1	JMJD1C	11.4	13.6	4.587835	1.15E-07	4.44E-06
TC1100007366.hg.1	TTC17	11.4	13.4	3.954804	1.16E-07	4.46E-06
TC0100010123.hg.1	FDPS	12.0	14.2	4.656765	1.20E-07	4.57E-06
TC0500007368.hg.1	ITGA2	12.4	14.7	4.698137	1.20E-07	4.58E-06
TC1400009732.hg.1	C14orf1	13.5	15.7	4.717433	1.21E-07	4.59E-06
TC0600007530.hg.1	HLA-E	7.6	10.8	9.043019	1.24E-07	4.67E-06
TC1500010869.hg.1	CCPG1; MIR628	7.0	9.8	7.121723	1.25E-07	4.70E-06
TC1900010856.hg.1	PLAUR	10.1	12.2	4.117000	1.25E-07	4.70E-06
TC0600007301.hg.1	BTN3A2	6.3	8.4	4.395966	1.27E-07	4.76E-06
TC0700011835.hg.1	PON2	13.5	15.5	3.947598	1.29E-07	4.82E-06
TC0700008003.hg.1	CLIP2	9.8	12.4	5.996976	1.31E-07	4.87E-06
TC1700010604.hg.1	NR1D1	8.6	10.9	5.169180	1.40E-07	5.17E-06
TC0300007161.hg.1	VIPR1	8.1	10.7	5.732924	1.40E-07	5.17E-06
TC2000009522.hg.1	CYP24A1	7.1	9.6	5.462795	1.41E-07	5.19E-06
TC1200012859.hg.1	RHOF	11.0	13.3	4.764003	1.42E-07	5.22E-06
TC1000012522.hg.1	BEND7	6.8	9.4	6.256079	1.48E-07	5.37E-06
TC1600008209.hg.1	CDH1	11.2	13.4	4.423503	1.48E-07	5.37E-06
TC0900009470.hg.1	ERMP1	10.2	12.1	3.888050	1.48E-07	5.37E-06
TC1000008234.hg.1	FAM213A	8.2	11.1	7.588055	1.50E-07	5.41E-06
TC0900012219.hg.1	CDKN2A	8.3	11.1	7.204943	1.51E-07	5.42E-06
TC1200010968.hg.1	DDIT3	3.8	6.7	7.531382	1.51E-07	5.42E-06
TC0200014990.hg.1	CIR1	8.5	10.7	4.552318	1.56E-07	5.56E-06
TC2000009964.hg.1	SDCBP2	6.1	8.9	6.917763	1.56E-07	5.56E-06
TC0200008930.hg.1	SLC20A1	13.6	15.5	3.924554	1.57E-07	5.57E-06
TC1900006508.hg.1	ABCA7	9.1	11.3	4.611255	1.57E-07	5.58E-06
TC0400011005.hg.1	RASSF6	6.2	8.5	4.768180	1.59E-07	5.62E-06
TC1700011007.hg.1	TTLL6	5.2	8.2	7.693709	1.62E-07	5.67E-06
TC1600007982.hg.1	HERPUD1	9.7	11.8	4.373674	1.66E-07	5.77E-06
TC1100012318.hg.1	IL18	11.0	13.1	4.325748	1.66E-07	5.77E-06
TC1700011773.hg.1	WBP2	10.6	12.7	4.384856	1.66E-07	5.77E-06
TC0200008300.hg.1	RMND5A	11.3	13.3	3.959378	1.68E-07	5.81E-06
TC0X00007919.hg.1	SRPX2	13.1	15.1	3.974648	1.68E-07	5.81E-06
TC2000006861.hg.1	RIN2	12.8	14.9	4.433229	1.69E-07	5.81E-06

TC0700009526.hg.1	CTAGE8; CTAGE4	6.6	9.1	5.707423	1.72E-07	5.91E-06
TC0700009682.hg.1	AOC1	7.0	9.8	7.246237	1.73E-07	5.92E-06
TC1100006492.hg.1	PNPLA2	4.3	7.0	6.342329	1.73E-07	5.92E-06
TC1500008988.hg.1	GOLGA8A; GOLGA	10.3	12.4	4.243743	1.76E-07	5.99E-06
TC1000009612.hg.1	KLF6	13.7	15.8	4.190042	1.77E-07	6.01E-06
TC1700008357.hg.1	DGKE	7.9	10.1	4.586925	1.82E-07	6.16E-06
TC1500007097.hg.1	B2M	13.7	15.8	4.393384	1.83E-07	6.19E-06
TC0500011125.hg.1	ANKRA2	5.9	8.1	4.669323	1.89E-07	6.33E-06
TC0100007705.hg.1	AZIN2	4.5	6.4	3.879227	1.89E-07	6.33E-06
TC0900012167.hg.1	GSN	10.1	12.1	3.846552	1.89E-07	6.33E-06
TC1600011505.hg.1	NPIPB4	15.1	17.1	3.940496	1.93E-07	6.43E-06
TC1000008904.hg.1	PDCD4; MIR4680	8.6	11.0	5.424510	1.94E-07	6.44E-06
TC0800007086.hg.1	DPYSL2	13.8	15.8	3.989066	1.94E-07	6.44E-06
TC0100017223.hg.1	LPGAT1	11.0	13.2	4.671891	1.95E-07	6.45E-06
TC0500009609.hg.1	FGFR4	8.2	10.4	4.707910	1.99E-07	6.59E-06
TC0800010138.hg.1	RAB11FIP1	10.2	12.3	4.123302	2.03E-07	6.67E-06
TC0200015211.hg.1	SLC40A1	6.8	9.9	8.956319	2.07E-07	6.80E-06
TC0600014074.hg.1	HIVEP1	9.3	11.1	3.665351	2.09E-07	6.82E-06
TC1300008229.hg.1	ZMYM5	8.3	10.8	5.699638	2.09E-07	6.82E-06
TC0300012728.hg.1	PLSCR1	9.1	11.0	3.852644	2.10E-07	6.83E-06
TC1700010276.hg.1	TMIGD1	5.9	8.8	7.349909	2.13E-07	6.91E-06
TC1500008983.hg.1	SLC12A6	8.7	10.7	3.903875	2.14E-07	6.91E-06
TC1500008995.hg.1	GOLGA8B	10.3	12.4	4.297931	2.16E-07	6.95E-06
TC2200009266.hg.1	GTPBP1	7.7	9.7	4.079408	2.17E-07	6.99E-06
TC0700008023.hg.1	GATSL2	7.3	9.4	4.231263	2.18E-07	6.99E-06
TC2200007196.hg.1	TOM1	6.8	8.9	4.280945	2.20E-07	7.05E-06
TC0100015212.hg.1	EPS8L3	9.2	11.5	4.888186	2.32E-07	7.41E-06
TC0200008291.hg.1	KDM3A	6.1	8.3	4.319744	2.33E-07	7.42E-06
TC0X00008759.hg.1	NSDHL	9.5	11.5	3.949424	2.41E-07	7.64E-06
TC0200009078.hg.1	EPB41L5	9.6	11.5	3.645232	2.45E-07	7.75E-06
TC1700011121.hg.1	TOB1	12.6	14.6	3.895476	2.47E-07	7.80E-06
TC0900008314.hg.1	FSD1L	8.5	10.5	3.852171	2.47E-07	7.80E-06
TC1800008578.hg.1	EPG5	7.5	9.9	5.280880	2.48E-07	7.80E-06
TC0200016743.hg.1	ITGB6	8.7	10.5	3.606825	2.50E-07	7.86E-06
TC1300010030.hg.1	N4BP2L2	11.2	13.1	3.894464	2.63E-07	8.25E-06
TC0900008148.hg.1	TDRD7	6.5	9.0	5.665319	2.68E-07	8.38E-06
TC0500013064.hg.1	MGAT4B	11.4	13.4	4.045547	2.68E-07	8.38E-06
TC2000008299.hg.1	GPCPD1	7.5	9.6	4.153018	2.71E-07	8.46E-06
TSUnmapped0000078	PADI1	5.6	7.8	4.680034	2.77E-07	8.59E-06
TC1700008920.hg.1	ITGB4	14.8	16.9	4.162398	2.78E-07	8.59E-06
TC1200007713.hg.1	SP1	14.2	16.1	3.707888	2.84E-07	8.72E-06
TC1500010727.hg.1	CCNDBP1	6.8	9.7	7.414224	2.84E-07	8.72E-06
TC0600014143.hg.1	PHF3	11.4	13.3	3.909975	2.85E-07	8.73E-06
TC0100009441.hg.1	CTTNBP2NL	9.6	11.6	4.079327	2.85E-07	8.73E-06
TC1600009958.hg.1	NPIPB4	14.4	16.4	4.017466	2.85E-07	8.73E-06
TC1600009855.hg.1	NPIPB6	12.0	14.2	4.540298	2.86E-07	8.73E-06
TC1400009839.hg.1	STON2	7.8	9.8	3.988060	2.93E-07	8.91E-06
TC0100009331.hg.1	STXBP3	8.3	10.2	3.545055	2.96E-07	8.97E-06
TC1900007382.hg.1	PGPEP1	7.5	10.4	7.260000	2.98E-07	9.03E-06
TC0700011318.hg.1	ERV3-1; ZNF117	7.0	9.6	5.711228	3.02E-07	9.13E-06

TC1500008245.hg.1	ABHD2	15.9	17.8	3.909009	3.04E-07	9.16E-06
TC1200010415.hg.1	SLC2A13	7.0	9.3	4.947541	3.09E-07	9.28E-06
TC1000012490.hg.1	PI4K2A	8.6	10.4	3.639735	3.09E-07	9.28E-06
TC0100014081.hg.1	PDZK1IP1	5.7	9.9	18.540350	3.10E-07	9.31E-06
TC1600010316.hg.1	TOX3	7.0	9.3	5.120252	3.11E-07	9.33E-06
TC1000007176.hg.1	MAP3K8	8.4	10.6	4.574021	3.14E-07	9.39E-06
TC0400007280.hg.1	WDR19	7.0	9.4	5.152135	3.15E-07	9.39E-06
TC1000012603.hg.1	FAM53B	8.3	10.2	3.745670	3.16E-07	9.40E-06
TC0300008488.hg.1	NR1I2	7.8	10.4	5.954912	3.19E-07	9.49E-06
TC1600011501.hg.1	NPIPB3	14.5	16.5	4.081373	3.25E-07	9.60E-06
TC1400010737.hg.1	KLHL28	7.2	9.3	4.189198	3.25E-07	9.60E-06
TC1000010956.hg.1	C10orf54	6.9	9.2	4.898195	3.28E-07	9.66E-06
TC1200007861.hg.1	LRP1	6.5	9.5	7.879633	3.28E-07	9.67E-06
TC0600007701.hg.1	PHF1	7.1	9.3	4.781047	3.29E-07	9.68E-06
TC1600011386.hg.1	ORAI3	8.2	10.4	4.641777	3.33E-07	9.75E-06
TC0X00007191.hg.1	EBP	12.2	14.5	5.139374	3.40E-07	9.90E-06
TC2200007655.hg.1	PPARA	9.7	11.9	4.423220	3.43E-07	9.97E-06
TC0400012366.hg.1	DDX60	8.0	10.5	5.838192	3.44E-07	1.00E-05
TC0500013399.hg.1	PANK3	10.5	12.6	4.391246	3.48E-07	1.01E-05
TC0900010388.hg.1	TMEM2	7.0	9.2	4.525159	3.52E-07	1.02E-05
TC0700010321.hg.1	ETV1	9.5	11.4	3.708442	3.56E-07	1.03E-05
TC0100013305.hg.1	FUCA1	6.5	8.4	3.515023	3.57E-07	1.03E-05
TC0500007337.hg.1	PARP8	11.2	13.0	3.505007	3.58E-07	1.03E-05
TC1700012216.hg.1	LGALS9	5.0	7.8	6.767173	3.60E-07	1.03E-05
TC2200009257.hg.1	TCN2	7.2	9.4	4.517360	3.63E-07	1.04E-05
TC1600008609.hg.1	HSD17B2	6.4	8.8	5.236801	3.63E-07	1.04E-05
TC1400009697.hg.1	NPC2; MIR4709	7.4	9.5	4.278141	3.65E-07	1.04E-05
TC0800011249.hg.1	RNF19A	8.7	10.6	3.671861	3.70E-07	1.05E-05
TC0300006994.hg.1	FBXL2	5.6	8.1	5.817228	3.71E-07	1.05E-05
TC1200011773.hg.1	APPL2	8.1	9.9	3.519198	3.74E-07	1.06E-05
TC0400010260.hg.1	SEPSECS	7.2	9.5	4.907725	3.75E-07	1.06E-05
TC1200008678.hg.1	EID3	8.0	10.3	5.071229	3.77E-07	1.06E-05
TC0100018355.hg.1	RHOU	9.2	10.9	3.400799	3.80E-07	1.07E-05
TC0700009536.hg.1	ARHGEF5	10.4	12.7	4.650538	3.82E-07	1.07E-05
TC1600007353.hg.1	NPIPB8	12.2	14.3	4.477308	3.84E-07	1.08E-05
TC1600009916.hg.1	NPIPB11	12.9	15.0	4.377597	3.85E-07	1.08E-05
TC0600009862.hg.1	AKAP12	14.5	16.7	4.671489	3.88E-07	1.08E-05
TC1600009147.hg.1	PRSS22	4.7	7.3	5.993947	3.94E-07	1.09E-05
TC0300010930.hg.1	SLC6A20	10.8	12.8	3.869560	3.94E-07	1.09E-05
TC0500012497.hg.1	TNIP1	9.7	11.5	3.504763	3.97E-07	1.10E-05
TC0800009752.hg.1	ASAH1	10.5	12.5	3.997050	3.99E-07	1.10E-05
TC0700010281.hg.1	THSD7A	3.9	6.9	7.927995	4.00E-07	1.10E-05
TC0100015975.hg.1	RIT1	8.2	9.9	3.392080	4.01E-07	1.10E-05
TC0700012911.hg.1	CTAGE8	6.7	8.9	4.605951	4.17E-07	1.14E-05
TC0600013054.hg.1	SERINC1	10.6	12.4	3.480392	4.28E-07	1.17E-05
TC1900008824.hg.1	MYADM	12.3	14.2	3.819170	4.34E-07	1.18E-05
TC1000011660.hg.1	MGEA5	7.2	9.2	3.930640	4.35E-07	1.18E-05
TC1600008407.hg.1	NPIPB15	11.5	13.6	4.198289	4.37E-07	1.18E-05
TC0500007780.hg.1	ARHGEF28	8.3	10.3	4.262015	4.38E-07	1.18E-05
TC1400010604.hg.1	MIA2	7.5	9.5	4.112090	4.41E-07	1.19E-05

TC0X00008836.hg.1	PLXNA3	6.4	8.4	4.111650	4.54E-07	1.22E-05
TC1700011128.hg.1	SPAG9	10.9	12.7	3.437631	4.56E-07	1.22E-05
TC0100011267.hg.1	ATP2B4	11.1	12.9	3.427846	4.60E-07	1.23E-05
TC0600007092.hg.1	FAM8A1	8.4	10.5	4.325124	4.60E-07	1.23E-05
TC1300006481.hg.1	ZMYM2	7.7	9.7	3.966541	4.63E-07	1.23E-05
TC0800011764.hg.1	FAM84B	12.8	14.7	3.702646	4.65E-07	1.24E-05
TC1600008307.hg.1	CHST4	9.7	11.5	3.584336	4.75E-07	1.26E-05
TC0200012727.hg.1	PUS10	6.7	8.8	4.403028	4.82E-07	1.27E-05
TC0300013949.hg.1	SATB1	6.9	8.7	3.507059	4.82E-07	1.27E-05
TC0800010427.hg.1	RB1CC1	7.8	10.2	5.291886	4.92E-07	1.29E-05
TC1400007644.hg.1	ACOT1	5.8	7.9	4.150157	4.93E-07	1.29E-05
TC0700009243.hg.1	TMEM140	5.9	9.4	11.647970	5.05E-07	1.31E-05
TC0100017500.hg.1	TMEM63A	8.8	10.7	3.744104	5.07E-07	1.31E-05
TC1500010783.hg.1	SEMA4B	13.1	14.9	3.584813	5.09E-07	1.32E-05
TC0900010570.hg.1	FRMD3	6.3	8.3	3.953516	5.10E-07	1.32E-05
TC1700009236.hg.1	SLC16A3; MIR6787	8.1	10.0	3.651012	5.10E-07	1.32E-05
TC0500011672.hg.1	PJA2	10.7	12.6	3.751362	5.13E-07	1.32E-05
TC0100015397.hg.1	CD58	9.2	11.2	3.830388	5.33E-07	1.37E-05
TC1500006994.hg.1	CHP1	14.6	16.3	3.353740	5.38E-07	1.38E-05
TC1900011452.hg.1	PTPRH	8.1	10.0	3.638038	5.41E-07	1.38E-05
TC1600011442.hg.1	MAP1LC3B	8.9	10.7	3.478730	5.43E-07	1.38E-05
TC1100009667.hg.1	ANO9	5.9	8.2	4.621320	5.54E-07	1.41E-05
TC1200006472.hg.1	ADIPOR2	10.5	12.6	4.502866	5.57E-07	1.41E-05
TC0X00011382.hg.1	ACSL4	9.2	11.4	4.537682	5.61E-07	1.42E-05
TC1200007251.hg.1	KIAA1551	9.7	11.7	3.819738	5.66E-07	1.43E-05
TC0200015976.hg.1	SP110	6.2	8.4	4.457669	5.73E-07	1.44E-05
TC0200013079.hg.1	DUSP11	10.4	12.3	3.709352	5.86E-07	1.47E-05
TC0500008691.hg.1	UBE2B	9.9	11.8	3.701879	5.90E-07	1.47E-05
TC1400010012.hg.1	LGMN	12.1	14.1	4.049710	5.93E-07	1.48E-05
TC0300013972.hg.1	SLC26A6; MIR6824	5.5	7.6	4.018851	5.99E-07	1.49E-05
TC0400008557.hg.1	USP53	9.3	11.4	4.228139	5.99E-07	1.49E-05
TC1600009954.hg.1	MAPK3	11.6	13.6	3.900420	6.03E-07	1.49E-05
TC1800007013.hg.1	DSG3	10.5	12.6	4.221451	6.09E-07	1.50E-05
TC1700012079.hg.1	PCYT2	5.2	6.9	3.269671	6.12E-07	1.51E-05
TC1400006529.hg.1	ANG; RNASE4	3.6	6.7	8.488475	6.26E-07	1.54E-05
TC1900006994.hg.1	DNM2	10.6	12.4	3.623471	6.31E-07	1.55E-05
TC0700010899.hg.1	POLR2J4	4.4	7.5	8.606151	6.32E-07	1.55E-05
TC1400010100.hg.1	ATG2B	6.2	8.9	6.505222	6.39E-07	1.56E-05
TC2000009180.hg.1	OSER1	7.2	9.6	5.251297	6.44E-07	1.57E-05
TC1500010234.hg.1	EFTUD1	8.3	10.0	3.240322	6.53E-07	1.59E-05
TC0100012089.hg.1	GPR137B	6.0	8.8	7.301366	6.62E-07	1.61E-05
TC0700010965.hg.1	IGFBP3	15.6	17.3	3.310499	6.81E-07	1.64E-05
TC1100007457.hg.1	ATG13	10.3	12.2	3.808989	6.90E-07	1.66E-05
TC1700007890.hg.1	ATP6V0A1; MIR50	11.0	12.8	3.371736	7.04E-07	1.69E-05
TC0600007552.hg.1	DDR1; MIR4640	11.5	13.3	3.478494	7.11E-07	1.70E-05
TC0100016000.hg.1	MEF2D	9.4	11.5	4.329299	7.18E-07	1.71E-05
TC0200016707.hg.1	ZNF514	6.8	8.7	3.781096	7.22E-07	1.72E-05
TC0400008175.hg.1	PDLIM5	14.2	15.9	3.346598	7.23E-07	1.72E-05
TC1600011409.hg.1	PDP2	6.6	8.3	3.271159	7.27E-07	1.73E-05
TC0700013400.hg.1	GATAD1	11.1	12.8	3.160838	7.38E-07	1.75E-05

TC0100011487.hg.1	TRAF5	9.9	11.6	3.289895	7.39E-07	1.75E-05
TC1200008184.hg.1	TBC1D15	8.7	10.4	3.291969	7.41E-07	1.75E-05
TC0200007717.hg.1	AHSA2	9.6	11.3	3.159468	7.44E-07	1.76E-05
TC1700012093.hg.1	FASN	10.1	12.2	4.169638	7.50E-07	1.76E-05
TC1600011364.hg.1	NPIPB5	14.3	16.0	3.221906	7.50E-07	1.76E-05
TSUnmapped0000053	RASA3	6.9	8.7	3.563838	7.68E-07	1.80E-05
TC0100013636.hg.1	RNF19B	9.4	11.3	3.887872	7.72E-07	1.80E-05
TC0300011757.hg.1	PROS1	5.9	7.9	4.232468	7.72E-07	1.80E-05
TC0600013361.hg.1	CITED2	7.9	9.7	3.491968	7.77E-07	1.81E-05
TC0300013751.hg.1	BDH1	8.5	11.0	5.666719	7.79E-07	1.81E-05
TC0500007249.hg.1	C5orf51	10.7	12.4	3.244547	7.87E-07	1.83E-05
TC0300008142.hg.1	TBC1D23	9.1	10.8	3.291274	7.87E-07	1.83E-05
TC0700010582.hg.1	HIBADH	6.0	8.5	5.825764	8.01E-07	1.86E-05
TC0600012814.hg.1	CD164	11.9	13.7	3.571155	8.05E-07	1.86E-05
TC0100012895.hg.1	MTHFR	7.1	10.1	7.706465	8.10E-07	1.87E-05
TC1700010811.hg.1	HDAC5	5.7	8.4	6.624478	8.17E-07	1.88E-05
TC0500010842.hg.1	PDE4D	7.5	9.5	3.805797	8.20E-07	1.88E-05
TC0X00007529.hg.1	IGBP1	8.8	10.9	4.115469	8.21E-07	1.88E-05
TC0100015258.hg.1	DRAM2	8.8	10.5	3.190261	8.22E-07	1.88E-05
TC0900010445.hg.1	NMRK1	7.0	9.3	4.707400	8.31E-07	1.90E-05
TC1200008081.hg.1	DYRK2	9.0	10.7	3.371562	8.40E-07	1.92E-05
TC1600011365.hg.1	NPIPB9	11.5	13.6	4.356646	8.46E-07	1.93E-05
TC1600011551.hg.1	TK2	6.6	8.4	3.415181	8.47E-07	1.93E-05
TC1600007147.hg.1	TMEM159	8.6	10.5	3.842725	8.49E-07	1.93E-05
TC0700012798.hg.1	KDM7A	6.2	8.1	3.610213	8.55E-07	1.94E-05
TC0200010980.hg.1	CCL20	4.9	6.7	3.372531	8.93E-07	2.02E-05
TC0600006793.hg.1	CDYL	8.3	10.3	3.907304	9.06E-07	2.04E-05
TSUnmapped0000013	SLC2A6	3.8	6.4	5.797426	9.16E-07	2.06E-05
TC0300009625.hg.1	TTC14	8.4	10.1	3.238999	9.28E-07	2.08E-05
TC1700008175.hg.1	CALCOCO2	11.2	13.1	3.561477	9.29E-07	2.08E-05
TC1900011894.hg.1	CACNA1A	5.5	8.1	5.917426	9.30E-07	2.08E-05
TC0700011876.hg.1	ASNS	9.4	11.2	3.431652	9.49E-07	2.12E-05
TC1700011208.hg.1	TRIM25; MIR3614	12.0	13.7	3.227021	9.52E-07	2.13E-05
TC0100018280.hg.1	FAM231D; LINC00	11.0	12.8	3.511853	9.61E-07	2.14E-05
TC0100013349.hg.1	RSRP1	9.3	11.2	3.838181	9.73E-07	2.16E-05
TC0100011197.hg.1	SHISA4	3.7	6.7	8.231073	9.79E-07	2.17E-05
TC1200010397.hg.1	CPNE8	11.6	13.4	3.543631	9.83E-07	2.17E-05
TC1200010006.hg.1	PLBD1	12.8	14.5	3.265832	9.86E-07	2.18E-05
TC1300008253.hg.1	CRYL1	8.5	10.3	3.399729	9.87E-07	2.18E-05
TC0100007645.hg.1	TINAGL1	11.8	13.5	3.243427	9.94E-07	2.19E-05
TC0500008494.hg.1	CSNK1G3	8.3	10.3	4.185803	1.01E-06	2.22E-05
TC0100017212.hg.1	SLC30A1	10.9	12.8	3.812023	1.02E-06	2.24E-05
TC1300009810.hg.1	ANKRD10	11.1	12.8	3.293586	1.03E-06	2.25E-05
TC1200010284.hg.1	DENND5B	10.3	12.1	3.513463	1.04E-06	2.27E-05
TC1200008269.hg.1	NAV3	8.9	10.5	3.151197	1.04E-06	2.27E-05
TC0200012956.hg.1	GFPT1	12.6	14.3	3.161908	1.04E-06	2.27E-05
TC1300007135.hg.1	ITM2B	12.0	13.7	3.411628	1.06E-06	2.30E-05
TC0200011023.hg.1	SP100	8.2	9.9	3.133420	1.08E-06	2.32E-05
TC0800009331.hg.1	ERICH1	10.7	12.5	3.560746	1.08E-06	2.32E-05
TC0800012308.hg.1	ADHFE1; C8orf46	4.4	6.7	5.098702	1.08E-06	2.33E-05

TC0100017471.hg.1	WDR26; MIR4742	9.8	11.5	3.272960	1.09E-06	2.34E-05
TC0100007638.hg.1	SERINC2	10.9	12.5	3.049404	1.09E-06	2.34E-05
TC0500007972.hg.1	VCAN	15.3	17.1	3.404859	1.10E-06	2.36E-05
TC0300009782.hg.1	EIF4A2; SNORA63	11.3	13.2	3.810878	1.10E-06	2.36E-05
TC0900009933.hg.1	FAM214B	6.4	8.9	5.524757	1.12E-06	2.38E-05
TC1200011470.hg.1	DUSP6	11.2	13.1	3.791044	1.14E-06	2.43E-05
TC0100014772.hg.1	SYDE2	6.9	8.8	3.626958	1.14E-06	2.43E-05
TC1200007897.hg.1	TSPAN31	11.0	12.8	3.389052	1.15E-06	2.43E-05
TC1700012321.hg.1	TBC1D3B; TBC1D3	5.3	7.2	3.809401	1.15E-06	2.43E-05
TC1600006519.hg.1	SSTR5	6.3	8.1	3.480708	1.15E-06	2.43E-05
TC0500012285.hg.1	FCHSD1	7.0	8.8	3.677238	1.17E-06	2.46E-05
TC1000010727.hg.1	ANK3	8.7	10.7	4.119990	1.18E-06	2.48E-05
TC0200007296.hg.1	GALM	6.1	8.5	5.322815	1.19E-06	2.48E-05
TC1200008933.hg.1	TPCN1	14.1	15.9	3.362022	1.19E-06	2.48E-05
TC0200007954.hg.1	PCYOX1	13.7	15.3	3.105734	1.19E-06	2.48E-05
TC0500013247.hg.1	PCDHB14	4.7	6.7	3.890246	1.20E-06	2.50E-05
TC1100011286.hg.1	SLC29A2	9.0	10.8	3.425287	1.21E-06	2.51E-05
TC1400007094.hg.1	KLHDC2	8.6	10.5	3.487717	1.21E-06	2.52E-05
TC0200010624.hg.1	PIKFYVE	10.0	11.7	3.244275	1.22E-06	2.53E-05
TC1100013061.hg.1	NADSYN1	9.6	11.3	3.188971	1.22E-06	2.54E-05
TC2100007999.hg.1	DNAJC28	4.5	6.2	3.154452	1.23E-06	2.56E-05
TC0500013322.hg.1	NAIP	8.1	10.1	3.975626	1.27E-06	2.64E-05
TC1600010864.hg.1	WDR59	10.3	11.9	3.131959	1.28E-06	2.65E-05
TC1300009732.hg.1	FAM155A	4.1	6.5	5.266538	1.29E-06	2.66E-05
TC1800007298.hg.1	LIPG	3.8	6.4	6.046864	1.29E-06	2.66E-05
TC1700012460.hg.1	ABCA5	5.0	6.9	3.683141	1.29E-06	2.66E-05
TC0100015803.hg.1	TDRKH	5.7	8.2	6.016657	1.29E-06	2.66E-05
TC1000011807.hg.1	SMNDC1	9.6	11.3	3.284328	1.29E-06	2.66E-05
TC1100009244.hg.1	PDZD3	6.7	8.7	4.157866	1.30E-06	2.67E-05
TC2000007197.hg.1	TP53INP2	5.3	8.2	7.284236	1.31E-06	2.68E-05
TC1000011736.hg.1	OBFC1	9.7	11.4	3.179063	1.31E-06	2.68E-05
TC0400012945.hg.1	HSD17B11	11.2	12.8	2.971127	1.32E-06	2.70E-05
TC1400007558.hg.1	SUSD6	6.7	8.4	3.265712	1.33E-06	2.71E-05
TC0800007460.hg.1	HGSNAT	11.3	13.3	4.145442	1.33E-06	2.71E-05
TC1300009731.hg.1	FAM155A	4.7	6.8	4.411091	1.33E-06	2.71E-05
TC1700008661.hg.1	PRKCA	7.5	9.4	3.694313	1.33E-06	2.71E-05
TC0200013943.hg.1	SLC35F5	13.0	15.0	3.933848	1.33E-06	2.71E-05
TC1300009994.hg.1	ERCC5	12.2	13.8	3.097548	1.34E-06	2.72E-05
TC0600010066.hg.1	IGF2R	10.0	11.7	3.169590	1.35E-06	2.74E-05
TC1700009377.hg.1	INPP5K	5.8	7.8	3.825239	1.36E-06	2.74E-05
TC1000009155.hg.1	DMBT1	6.1	8.0	3.648538	1.36E-06	2.74E-05
TC1700008378.hg.1	MSI2	8.6	10.8	4.415714	1.37E-06	2.77E-05
TC0700010581.hg.1	HIBADH	12.0	13.5	2.944785	1.38E-06	2.78E-05
TC1900006804.hg.1	TNFSF9	6.4	8.6	4.633398	1.38E-06	2.78E-05
TC0100008912.hg.1	CYR61	7.7	10.4	6.746221	1.39E-06	2.80E-05
TC1700006741.hg.1	KCTD11	7.6	9.7	4.227614	1.41E-06	2.83E-05
TC1600010740.hg.1	PDXDC2P	11.5	13.3	3.339672	1.42E-06	2.85E-05
TC0900012209.hg.1	TTC39B	5.8	8.0	4.727558	1.43E-06	2.86E-05
TC0300012155.hg.1	HGD	6.4	8.3	3.959537	1.45E-06	2.90E-05
TC0600011315.hg.1	ZFP57	9.3	11.0	3.191513	1.46E-06	2.92E-05

TC0800009878.hg.1	ENTPD4; LOXL2	10.3	12.2	3.574418	1.48E-06	2.94E-05
TC1600008231.hg.1	NFAT5	12.4	14.4	4.144310	1.49E-06	2.95E-05
TC0X00006671.hg.1	MOSPD2	7.7	9.3	3.118513	1.49E-06	2.96E-05
TC0300012665.hg.1	XRN1	8.9	10.8	3.797163	1.49E-06	2.96E-05
TC0200007618.hg.1	EML6	4.1	6.0	3.929544	1.51E-06	2.99E-05
TC0700013342.hg.1	CHN2	5.6	7.2	3.087981	1.52E-06	2.99E-05
TC1200009903.hg.1	KLRC4-KLRK1; KL	7.3	10.1	7.247649	1.52E-06	3.00E-05
TC0X00008831.hg.1	ATP6AP1	10.7	12.3	3.072832	1.53E-06	3.00E-05
TC1200010984.hg.1	CTDSP2	9.9	11.8	3.560025	1.53E-06	3.01E-05
TC1400009629.hg.1	ZFYVE1	5.1	7.0	3.734354	1.53E-06	3.01E-05
TC0700007067.hg.1	MTURN	3.8	5.6	3.346832	1.55E-06	3.04E-05
TC0600012952.hg.1	FRK	9.5	11.2	3.153193	1.56E-06	3.05E-05
TC0400012922.hg.1	TLR6	12.1	13.8	3.157144	1.58E-06	3.08E-05
TC0700007024.hg.1	TAX1BP1	9.8	11.6	3.288933	1.59E-06	3.08E-05
TC0600007512.hg.1	TRIM15	7.0	9.0	3.816483	1.59E-06	3.09E-05
TC1600011450.hg.1	SPIRE2	6.6	9.4	6.989825	1.60E-06	3.10E-05
TC2100008494.hg.1	IFNAR2	10.5	12.4	3.765317	1.61E-06	3.11E-05
TC0100011253.hg.1	BTG2	6.1	8.9	7.372474	1.62E-06	3.12E-05
TC0600012072.hg.1	ELOVL5	13.8	15.4	3.087602	1.62E-06	3.12E-05
TC0100013983.hg.1	TMEM53	6.4	9.0	6.328861	1.63E-06	3.13E-05
TC1200007844.hg.1	RBMS2	9.7	11.5	3.397523	1.63E-06	3.13E-05
TC0100018388.hg.1	SLC35E2B	10.8	12.6	3.458056	1.63E-06	3.14E-05
TC1300008359.hg.1	PARP4	11.2	12.7	2.910363	1.64E-06	3.15E-05
TC0100012921.hg.1	DHRS3; MIR6730	12.2	13.9	3.182210	1.64E-06	3.15E-05
TC1500009669.hg.1	VPS13C	6.9	8.7	3.544554	1.65E-06	3.15E-05
TC1500008840.hg.1	TJP1	12.7	14.3	3.138278	1.65E-06	3.16E-05
TC0200016684.hg.1	TIA1	9.7	11.3	3.116286	1.66E-06	3.16E-05
TC0100008536.hg.1	INADL	8.4	10.0	2.977842	1.66E-06	3.17E-05
TC0X00007213.hg.1	CCDC120	6.8	8.7	3.574532	1.68E-06	3.19E-05
TC0900007435.hg.1	CBWD5	13.6	15.4	3.388754	1.69E-06	3.21E-05
TC1500007739.hg.1	THSD4	8.2	10.0	3.456614	1.70E-06	3.23E-05
TC0800012285.hg.1	HMBOX1	9.5	11.1	3.091920	1.73E-06	3.26E-05
TC0400011695.hg.1	SEC24D	7.6	9.6	3.935183	1.75E-06	3.31E-05
TC1500008485.hg.1	IGF1R	10.8	12.4	2.901132	1.78E-06	3.34E-05
TC0600008212.hg.1	C6orf141	10.7	12.8	4.268177	1.81E-06	3.40E-05
TC2000008130.hg.1	TBC1D20	9.2	10.9	3.059064	1.84E-06	3.46E-05
TC0600012413.hg.1	IBTK	10.7	12.5	3.533060	1.88E-06	3.51E-05
TC0700011692.hg.1	KIAA1324L	6.8	8.5	3.211931	1.88E-06	3.51E-05
TC1100010705.hg.1	AMBRA1	12.7	14.3	2.935076	1.88E-06	3.52E-05
TC1200009789.hg.1	APOBEC1	3.4	5.2	3.553514	1.88E-06	3.52E-05
TC1100009168.hg.1	SIDT2	7.9	9.9	4.016412	1.89E-06	3.54E-05
TC1300008819.hg.1	TPT1; SNORA31	13.4	15.0	3.032222	1.91E-06	3.55E-05
TC0700009399.hg.1	TAS2R4	6.9	8.9	4.181120	1.91E-06	3.55E-05
TC0800008145.hg.1	WWP1	9.4	10.9	2.929534	1.92E-06	3.56E-05
TC0700012906.hg.1	ARHGEF35	9.8	11.4	3.047276	1.94E-06	3.59E-05
TC0300009035.hg.1	TRPC1	6.8	8.8	3.994062	1.94E-06	3.59E-05
TC1200009724.hg.1	SCNN1A	10.5	13.7	9.008363	1.94E-06	3.59E-05
TC0700011813.hg.1	BET1	9.3	10.9	2.971458	1.95E-06	3.60E-05
TC0900006758.hg.1	DENND4C	8.4	10.2	3.429236	1.97E-06	3.63E-05
TC1700008455.hg.1	YPEL2	3.6	5.9	4.981769	1.97E-06	3.63E-05

TC1900010009.hg.1	JUND	6.7	9.6	7.368137	1.99E-06	3.64E-05
TC0600013409.hg.1	HIVEP2	5.2	7.5	5.048700	2.00E-06	3.66E-05
TC1700010798.hg.1	DUSP3	8.8	10.4	2.951060	2.07E-06	3.77E-05
TC1700010738.hg.1	EZH1	7.4	9.2	3.668720	2.08E-06	3.79E-05
TC1400009123.hg.1	SOS2	7.5	9.7	4.447950	2.08E-06	3.79E-05
TC1200008466.hg.1	NUDT4	14.9	16.4	2.960028	2.09E-06	3.79E-05
TC0500007610.hg.1	ERBB2IP	10.9	12.4	2.877928	2.09E-06	3.80E-05
TC1700012249.hg.1	MIR4728; ERBB2	7.2	8.9	3.323572	2.10E-06	3.80E-05
TC1600011465.hg.1	ERVK13-1	8.2	9.9	3.311481	2.12E-06	3.84E-05
TC1500010764.hg.1	CLK3	7.4	9.0	3.091055	2.12E-06	3.85E-05
TC0600007847.hg.1	CDKN1A	6.3	8.3	4.052195	2.15E-06	3.89E-05
TC2000008140.hg.1	SLC52A3	6.8	8.6	3.470299	2.16E-06	3.89E-05
TC0100012278.hg.1	SCCPDH	7.4	9.0	3.164040	2.17E-06	3.92E-05
TC1500007645.hg.1	MAP2K5	5.5	7.2	3.193652	2.17E-06	3.92E-05
TC1100006598.hg.1	SLC22A18	5.9	8.0	4.128505	2.18E-06	3.92E-05
TC1900011399.hg.1	MBOAT7	11.6	13.1	2.914757	2.19E-06	3.93E-05
TC0600014111.hg.1	SYNGAP1; MIR500	7.8	9.7	3.883506	2.19E-06	3.93E-05
TC1500009606.hg.1	MYO1E	4.7	6.6	3.700789	2.20E-06	3.94E-05
TC0200016750.hg.1	NFE2L2	10.9	12.7	3.464694	2.21E-06	3.95E-05
TSUnmapped0000025	DYRK1B	4.4	6.3	3.698131	2.21E-06	3.95E-05
TC0400010681.hg.1	LRRC66	7.5	9.3	3.427685	2.22E-06	3.96E-05
TC0500011225.hg.1	LHFPL2	10.4	11.9	2.850641	2.25E-06	4.02E-05
TC0100015932.hg.1	PBXIP1	5.7	8.0	5.083284	2.27E-06	4.04E-05
TC0200013750.hg.1	FHL2	9.5	11.2	3.099113	2.28E-06	4.05E-05
TC0700008031.hg.1	GTF2IRD2B	5.0	6.7	3.219816	2.30E-06	4.08E-05
TC0100018390.hg.1	SLC35E2	11.1	12.9	3.438813	2.30E-06	4.09E-05
TC0500012032.hg.1	AFF4	12.3	13.9	2.926388	2.31E-06	4.09E-05
TC1000008020.hg.1	FAM149B1	7.1	8.6	2.872740	2.35E-06	4.15E-05
TC1100012350.hg.1	DRD2	3.8	6.3	5.985151	2.36E-06	4.16E-05
TC0100013908.hg.1	SLC2A1	15.1	16.6	2.944420	2.36E-06	4.17E-05
TC1400006851.hg.1	ARHGAP5	10.0	11.6	3.054077	2.37E-06	4.18E-05
TC0600013126.hg.1	PTPRK	13.0	14.5	2.826674	2.38E-06	4.18E-05
TC0800009745.hg.1	FGL1	4.6	6.4	3.345133	2.38E-06	4.19E-05
TC0100012008.hg.1	KCNK1	8.5	10.2	3.188106	2.42E-06	4.23E-05
TC1900011777.hg.1	CYTH2	10.7	12.2	2.903896	2.42E-06	4.23E-05
TC0400009033.hg.1	TRIM2	10.3	12.3	3.830661	2.43E-06	4.23E-05
TC1700007451.hg.1	CPD	9.7	11.4	3.230810	2.44E-06	4.23E-05
TC0100012473.hg.1	CCNL2	11.0	12.5	2.947789	2.44E-06	4.23E-05
TC2100007803.hg.1	APP	15.1	16.5	2.755837	2.46E-06	4.26E-05
TC2100008030.hg.1	RCAN1	6.8	8.2	2.694035	2.48E-06	4.29E-05
TC1300008137.hg.1	TMCO3	9.6	11.3	3.167350	2.48E-06	4.29E-05
TC1100011257.hg.1	EFEMP2	5.4	7.7	4.647858	2.48E-06	4.29E-05
TC0700011821.hg.1	SGCE	6.3	8.1	3.495708	2.49E-06	4.29E-05
TC1600007887.hg.1	RBL2	10.5	12.0	2.840040	2.52E-06	4.32E-05
TC1200010926.hg.1	BAZ2A	10.8	12.4	3.001365	2.52E-06	4.33E-05
TC1100011126.hg.1	PLA2G16	10.3	12.0	3.126164	2.55E-06	4.38E-05
TC1700012346.hg.1	CLDN7	10.5	12.3	3.258799	2.59E-06	4.43E-05
TC1200007070.hg.1	ETNK1	11.1	12.8	3.335757	2.62E-06	4.48E-05
TC0200008971.hg.1	CBWD2	12.2	13.7	2.781628	2.64E-06	4.51E-05
TC0200010445.hg.1	CASP10	6.5	8.7	4.562597	2.66E-06	4.52E-05

TC0100011664.hg.1	2-Mar	5.9	8.1	4.527296	2.67E-06	4.54E-05
TC0100015754.hg.1	CTSK	5.6	7.3	3.180076	2.70E-06	4.59E-05
TC1600009731.hg.1	COG7	8.4	10.0	3.030317	2.71E-06	4.59E-05
TC1500009804.hg.1	DENND4A	9.6	11.3	3.134958	2.71E-06	4.61E-05
TC0900009825.hg.1	DDX58	6.3	7.9	2.998544	2.76E-06	4.68E-05
TC0200015087.hg.1	SESTD1	9.1	11.3	4.509868	2.78E-06	4.70E-05
TC1200010538.hg.1	AMIGO2	9.4	11.0	3.015476	2.80E-06	4.72E-05
TC0200010362.hg.1	COQ10B	8.9	10.4	2.892577	2.80E-06	4.72E-05
TC2100007996.hg.1	TMEM50B	9.0	10.6	3.041225	2.82E-06	4.74E-05
TC1600007096.hg.1	TMC7	12.1	13.8	3.185967	2.83E-06	4.75E-05
TC1200012617.hg.1	FGD4	10.3	11.8	2.805608	2.84E-06	4.76E-05
TC0100012006.hg.1	KCNK1	11.2	12.7	2.918635	2.84E-06	4.76E-05
TC0900011655.hg.1	ZER1	6.5	9.1	6.069767	2.86E-06	4.79E-05
TC1500009452.hg.1	GNB5	7.6	9.4	3.532630	2.86E-06	4.79E-05
TC0200007096.hg.1	FOSL2	7.4	9.0	3.077105	2.89E-06	4.81E-05
TC0200011980.hg.1	ITSN2	10.5	12.1	2.935169	2.92E-06	4.84E-05
TC0600013203.hg.1	VNN1	11.4	13.1	3.383947	2.93E-06	4.86E-05
TC1900006652.hg.1	TJP3	7.6	9.5	3.855596	2.96E-06	4.91E-05
TC0700012909.hg.1	OR2A7; ARHGEF3	9.0	10.5	2.766445	2.96E-06	4.91E-05
TC1800007471.hg.1	PMAIP1	5.8	7.4	3.159231	3.01E-06	4.97E-05
TC0600010609.hg.1	PXDC1	8.4	9.9	2.727489	3.01E-06	4.97E-05
TC0100007505.hg.1	WDTC1	7.8	9.5	3.218963	3.02E-06	4.98E-05
TC1000008276.hg.1	CCSER2	11.2	12.8	3.089610	3.05E-06	5.02E-05
TC1800008418.hg.1	GAREM1	8.3	10.1	3.436937	3.09E-06	5.07E-05
TC2100008390.hg.1	PTTG1IP	14.5	16.1	3.174844	3.10E-06	5.09E-05
TC1300006601.hg.1	SPATA13; C1QTNF	5.2	6.8	3.095987	3.11E-06	5.10E-05
TC0200016768.hg.1	PECR	9.1	10.7	3.107867	3.11E-06	5.10E-05
TC0900009346.hg.1	CBWD1	11.0	12.5	2.726243	3.13E-06	5.12E-05
TC1800008231.hg.1	ESCO1	8.2	9.8	3.024230	3.14E-06	5.13E-05
TC1900009459.hg.1	INSR	6.6	8.8	4.630438	3.15E-06	5.14E-05
TC1400008591.hg.1	TMEM55B	6.1	7.6	2.857157	3.18E-06	5.19E-05
TC0100014349.hg.1	JUN	7.9	10.0	4.412429	3.19E-06	5.20E-05
TC0100009641.hg.1	NBPF8	5.8	7.3	2.792395	3.21E-06	5.22E-05
TC0700008246.hg.1	DMTF1	14.0	15.7	3.135514	3.30E-06	5.33E-05
TC0500011448.hg.1	ARRDC3	11.2	12.8	2.961034	3.31E-06	5.34E-05
TC1100013191.hg.1	POLD4	8.7	10.3	3.028340	3.32E-06	5.35E-05
TC0600011173.hg.1	GUSBP2	13.8	15.2	2.612155	3.35E-06	5.38E-05
TC1600008128.hg.1	CMTM3	5.5	7.3	3.462493	3.35E-06	5.38E-05
TC0900007475.hg.1	CBWD3	12.1	13.6	2.923717	3.37E-06	5.39E-05
TC1900011794.hg.1	ZNF701; ZNF137P	11.2	12.8	2.867155	3.39E-06	5.41E-05
TC1000008896.hg.1	SMC3	14.5	15.9	2.683297	3.40E-06	5.42E-05
TC1100009309.hg.1	SORL1	13.3	15.0	3.314225	3.40E-06	5.42E-05
TC1900011774.hg.1	EMP3	9.9	11.3	2.730910	3.42E-06	5.44E-05
TC0700011497.hg.1	MLXIPL	5.7	8.5	7.170018	3.43E-06	5.45E-05
TC0200015876.hg.1	SERPINE2	15.9	17.5	3.175025	3.50E-06	5.53E-05
TC0700012734.hg.1	CREB3L2	9.0	10.5	2.915627	3.54E-06	5.59E-05
TC1000011005.hg.1	P4HA1	9.1	10.7	2.937926	3.54E-06	5.59E-05
TC0700011944.hg.1	ZNF394	7.1	8.7	3.071888	3.55E-06	5.60E-05
TC0400012943.hg.1	C4orf36	7.4	9.3	3.982532	3.56E-06	5.61E-05
TC0700013423.hg.1	ZNF655	7.6	9.2	3.139020	3.62E-06	5.69E-05

TC0100013348.hg.1	SYF2	7.9	9.4	2.922551	3.62E-06	5.69E-05
TC1600010861.hg.1	FA2H	9.6	11.1	2.676110	3.65E-06	5.72E-05
TC1500007034.hg.1	SNAP23	10.3	11.8	2.810147	3.67E-06	5.74E-05
TC0500013015.hg.1	PHYKPL	6.7	8.8	4.146594	3.71E-06	5.79E-05
TC0100009760.hg.1	NUDT4P1; NUDT4I	15.5	16.9	2.676245	3.72E-06	5.79E-05
TC0100009833.hg.1	NUDT4; NUDT4P1	15.5	16.9	2.676245	3.72E-06	5.79E-05
TC1700006721.hg.1	SLC16A13	8.3	10.0	3.281891	3.73E-06	5.80E-05
TC2200008831.hg.1	PMM1	4.0	5.9	3.730597	3.74E-06	5.81E-05
TC0600009368.hg.1	HINT3	8.9	10.6	3.182955	3.75E-06	5.81E-05
TC1900011926.hg.1	ZNF91	7.9	9.4	2.730117	3.75E-06	5.82E-05
TC1600009396.hg.1	TNP2	4.7	6.5	3.582321	3.78E-06	5.86E-05
TC0600009712.hg.1	UTRN	9.2	10.9	3.294008	3.81E-06	5.90E-05
TC0200010791.hg.1	PNKD; MIR6810	5.9	7.6	3.332385	3.82E-06	5.91E-05
TC0600014277.hg.1	HLA-DMA	5.8	8.0	4.595253	3.83E-06	5.92E-05
TC1900007384.hg.1	GDF15	4.4	6.0	3.129633	3.84E-06	5.93E-05
TC0100006771.hg.1	H6PD	6.7	8.1	2.631431	3.86E-06	5.95E-05
TC1100008673.hg.1	TMEM135	8.6	10.2	3.106642	3.89E-06	5.98E-05
TC2200008856.hg.1	NAGA	9.0	10.6	2.911113	3.89E-06	5.98E-05
TC1900008252.hg.1	ZNF224	6.9	9.0	4.452157	3.94E-06	6.05E-05
TC0100013128.hg.1	AKR7L	8.9	10.4	2.884585	3.95E-06	6.05E-05
TC0100018433.hg.1	RRAGC	8.5	10.5	3.979617	3.98E-06	6.09E-05
TC0300009815.hg.1	LPP	11.9	13.4	2.847764	4.00E-06	6.13E-05
TC2200007505.hg.1	3-Sep	5.2	6.7	2.830597	4.02E-06	6.16E-05
TC1600008739.hg.1	FOXL1	9.2	10.8	2.895675	4.03E-06	6.16E-05
TC1900007836.hg.1	FXYD3; MIR6887	8.8	10.6	3.496611	4.06E-06	6.19E-05
TC1600011445.hg.1	SPG7	8.0	9.7	3.098699	4.10E-06	6.23E-05
TC0700010189.hg.1	CYTH3	8.6	10.1	2.826136	4.11E-06	6.24E-05
TC1700007017.hg.1	SNORD49A; SNOF	13.6	15.3	3.162374	4.14E-06	6.29E-05
TC2000008295.hg.1	GPCPD1	7.3	8.7	2.714092	4.16E-06	6.31E-05
TC0200016775.hg.1	ATG9A	11.8	13.3	2.855158	4.18E-06	6.33E-05
TC1700012277.hg.1	CDK5RAP3	10.2	12.1	3.656330	4.18E-06	6.33E-05
TC0X00010793.hg.1	ZDHHC9	12.4	13.9	2.839207	4.19E-06	6.34E-05
TC0300008989.hg.1	SLC25A36	10.3	11.8	2.868321	4.19E-06	6.34E-05
TC0500011751.hg.1	FEM1C	9.7	11.1	2.745332	4.23E-06	6.38E-05
TC1100010063.hg.1	TMEM41B	11.0	12.6	2.940935	4.23E-06	6.38E-05
TC1700006772.hg.1	KDM6B	7.4	9.0	3.015894	4.26E-06	6.42E-05
TC0100012055.hg.1	GGPS1	8.5	10.1	2.912919	4.27E-06	6.43E-05
TC0600008146.hg.1	RUNX2	8.8	10.7	3.642070	4.29E-06	6.44E-05
TC1800008234.hg.1	ABHD3	8.9	10.5	2.928410	4.29E-06	6.44E-05
TC1700008499.hg.1	BCAS3	5.2	7.1	3.643987	4.30E-06	6.45E-05
TC1900008134.hg.1	CYP2B6	8.7	10.7	3.872667	4.32E-06	6.48E-05
TC0400012857.hg.1	RAPGEF2	8.7	10.4	3.369084	4.35E-06	6.51E-05
TC0700011518.hg.1	GTF2IRD2; GTF2IF	9.7	11.1	2.717624	4.42E-06	6.60E-05
TC0100010755.hg.1	RALGPS2	11.9	13.4	2.973919	4.50E-06	6.70E-05
TC2000007572.hg.1	NCOA3	14.0	15.3	2.587154	4.50E-06	6.70E-05
TC0X00009386.hg.1	SRPX	4.3	6.0	3.296312	4.53E-06	6.73E-05
TC2200009352.hg.1	LOC400927; CSNK	8.0	10.0	3.863043	4.54E-06	6.74E-05
TC1800006891.hg.1	C18orf8	7.2	8.9	3.284880	4.55E-06	6.75E-05
TC2200009346.hg.1	C1QTNF6	5.4	7.6	4.565739	4.58E-06	6.77E-05
TSUnmapped00000401	INPP5D	7.6	9.1	2.981543	4.58E-06	6.77E-05

TC0600009669.hg.1	ADGRG6	10.9	12.7	3.457059	4.61E-06	6.81E-05
TC0900006815.hg.1	DMRTA1	4.6	7.2	6.279923	4.63E-06	6.84E-05
TC0200015764.hg.1	CNPPD1	10.1	11.7	2.946569	4.66E-06	6.88E-05
TC0100018519.hg.1	F11R	13.9	15.4	2.762651	4.74E-06	6.98E-05
TC0X00007134.hg.1	USP11	9.8	11.4	2.923803	4.75E-06	6.99E-05
TC0900008250.hg.1	ZNF189	7.3	8.8	2.746744	4.76E-06	7.00E-05
TC1200011573.hg.1	NR2C1	9.8	11.3	2.693527	4.76E-06	7.00E-05
TC0600011957.hg.1	ADGRF1	14.3	15.9	3.137872	4.80E-06	7.05E-05
TC1200006688.hg.1	NANOG	4.6	6.1	2.929018	4.80E-06	7.05E-05
TC1400009538.hg.1	DCAF5	8.2	9.7	2.806830	4.83E-06	7.08E-05
TC0600009333.hg.1	SMPDL3A	9.1	10.5	2.701239	4.84E-06	7.08E-05
TC0700011962.hg.1	AZGP1	6.2	8.7	5.797155	4.89E-06	7.13E-05
TC1700011910.hg.1	CYTH1	7.8	9.4	2.974499	4.91E-06	7.15E-05
TC1600009066.hg.1	HAGH	7.2	8.9	3.230816	4.95E-06	7.21E-05
TC0500008890.hg.1	PCDHGC3; PCDHG	4.9	7.1	4.684092	5.00E-06	7.27E-05
TC1700007138.hg.1	TVP23B	10.4	11.9	2.780049	5.04E-06	7.30E-05
TC0600011406.hg.1	POU5F1	5.5	7.2	3.252692	5.04E-06	7.30E-05
TC1900009670.hg.1	DOCK6	8.2	10.1	3.659017	5.10E-06	7.38E-05
TC1600010752.hg.1	AARS	12.3	13.7	2.716853	5.19E-06	7.50E-05
TC0900008441.hg.1	UGCG	8.7	10.4	3.101006	5.20E-06	7.51E-05
TC2000009196.hg.1	SERINC3	14.6	16.1	2.783523	5.24E-06	7.56E-05
TC1200010009.hg.1	GUCY2C	4.1	7.0	7.864692	5.25E-06	7.56E-05
TC1900011933.hg.1	ZNF585A	6.7	8.2	2.728993	5.28E-06	7.60E-05
TC0600007833.hg.1	PNPLA1	4.5	6.1	3.109044	5.31E-06	7.64E-05
TC0X00007573.hg.1	OGT	13.9	15.3	2.534283	5.34E-06	7.67E-05
TC0200009596.hg.1	ACVR2A	8.4	9.8	2.700324	5.41E-06	7.76E-05
TC0400012826.hg.1	HERC3	6.4	7.8	2.691489	5.44E-06	7.79E-05
TC2000008445.hg.1	FLRT3	10.9	12.5	2.942129	5.46E-06	7.81E-05
TC1700012275.hg.1	EFCAB13	7.4	9.2	3.280796	5.57E-06	7.95E-05
TC0700009333.hg.1	UBN2	10.9	12.2	2.562001	5.59E-06	7.98E-05
TC1200009160.hg.1	P2RX4	5.2	7.7	5.694034	5.61E-06	7.99E-05
TC0X00007889.hg.1	DIAPH2	10.2	11.9	3.223610	5.64E-06	8.02E-05
TC0100010546.hg.1	BLZF1	9.6	11.1	2.756275	5.66E-06	8.05E-05
TC0300006691.hg.1	FGD5	4.9	7.1	4.636293	5.70E-06	8.10E-05
TC2100008495.hg.1	IL10RB	8.4	9.9	2.976279	5.71E-06	8.10E-05
TC1900012020.hg.1	ZNF432	6.6	8.4	3.432156	5.72E-06	8.10E-05
TC0300007596.hg.1	FLNB	12.5	14.1	2.839717	5.74E-06	8.12E-05
TC0100016356.hg.1	F5	7.1	9.3	4.431817	5.76E-06	8.14E-05
TC1700007915.hg.1	WNK4	7.5	9.4	3.737034	5.77E-06	8.14E-05
TC0700013524.hg.1	MACC1	4.9	7.0	4.334225	5.92E-06	8.35E-05
TC1700009982.hg.1	USP32P2; FAM106	7.2	8.7	2.880799	5.94E-06	8.37E-05
TC0X00007559.hg.1	GJB1	5.9	7.3	2.728307	5.95E-06	8.37E-05
TC0200010236.hg.1	GULP1	9.3	11.0	3.347849	5.96E-06	8.38E-05
TC1500010744.hg.1	GCOM1; MYZAP; F	7.6	9.5	3.654098	5.96E-06	8.38E-05
TC0600012647.hg.1	FBXL4	9.0	10.5	2.851278	5.98E-06	8.41E-05
TC1700010230.hg.1	TIAF1; MYO18A	10.9	12.6	3.297535	6.00E-06	8.41E-05
TC0700013594.hg.1	TRIM4	9.3	11.0	3.097108	6.06E-06	8.48E-05
TC0800010765.hg.1	TRAM1	13.9	15.3	2.600856	6.07E-06	8.50E-05
TC1600011427.hg.1	IST1	11.6	13.0	2.646791	6.11E-06	8.55E-05
TC0300009500.hg.1	FNDC3B	15.6	16.9	2.469056	6.13E-06	8.57E-05

TC0500009319.hg.1	CCNG1	9.9	11.5	3.008090	6.17E-06	8.61E-05
TC2200009252.hg.1	SEC14L2	7.6	9.2	2.901620	6.18E-06	8.62E-05
TC1400010730.hg.1	EGLN3	4.4	6.3	3.704187	6.22E-06	8.68E-05
TC0800010749.hg.1	NCOA2	10.8	12.2	2.684270	6.24E-06	8.69E-05
TC0200008335.hg.1	THNSL2	7.5	9.2	3.159341	6.25E-06	8.71E-05
TC0600012655.hg.1	PNISR	9.8	11.2	2.621423	6.29E-06	8.74E-05
TC1100011083.hg.1	AHNAK	13.3	14.6	2.539165	6.33E-06	8.80E-05
TC0600007887.hg.1	ZFAND3	10.6	12.0	2.601349	6.36E-06	8.82E-05
TC0100015049.hg.1	FRRS1	8.9	10.6	3.330423	6.41E-06	8.88E-05
TSUnmapped0000001	DYRK1B	5.3	6.9	3.219956	6.44E-06	8.91E-05
TC0600007852.hg.1	C6orf89	10.4	11.8	2.632863	6.51E-06	8.99E-05
TC1700009640.hg.1	ZBTB4	6.2	7.9	3.436710	6.54E-06	9.02E-05
TC0200013516.hg.1	TMEM127	13.6	15.0	2.702852	6.55E-06	9.02E-05
TC1600008865.hg.1	ACSF3	8.2	9.8	2.977824	6.58E-06	9.06E-05
TC0100012221.hg.1	C1orf101	5.3	6.9	3.084275	6.59E-06	9.06E-05
TC0900009003.hg.1	POMT1	9.7	11.2	2.964864	6.61E-06	9.08E-05
TC1000009880.hg.1	NMT2	10.4	11.7	2.425861	6.68E-06	9.15E-05
TC0300013787.hg.1	TTLL3	7.7	9.1	2.718647	6.68E-06	9.15E-05
TC0200009978.hg.1	PDK1	9.7	11.3	2.911434	6.69E-06	9.15E-05
TC2200008055.hg.1	PI4KA	8.0	9.5	2.758517	6.73E-06	9.20E-05
TC1000011596.hg.1	DNMBP	13.1	14.5	2.703146	6.74E-06	9.20E-05
TC0500010511.hg.1	C5orf42	8.5	10.1	2.999978	6.76E-06	9.22E-05
TC0300010123.hg.1	CRBN	7.1	8.6	2.991951	6.77E-06	9.23E-05
TC1600006483.hg.1	MSLN	10.3	11.6	2.502525	6.79E-06	9.25E-05
TC0X00008330.hg.1	XIAP	10.9	12.4	2.864719	6.81E-06	9.26E-05
TC0600012434.hg.1	ME1	7.7	9.2	2.751826	6.81E-06	9.26E-05
TC0X00007668.hg.1	UPRT	6.8	8.2	2.623820	6.82E-06	9.26E-05
TC0X00007194.hg.1	TBC1D25	8.7	10.1	2.581102	6.83E-06	9.27E-05
TC1900008057.hg.1	ZFP36	10.6	11.9	2.619671	6.85E-06	9.29E-05
TC0500013342.hg.1	ERAP1	10.4	11.8	2.658545	6.86E-06	9.29E-05
TC2200009314.hg.1	RTN4R	6.5	8.1	3.103280	6.87E-06	9.30E-05
TC0500012210.hg.1	TMEM173	9.1	10.8	3.435575	6.88E-06	9.30E-05
TC1300009348.hg.1	SPRY2	6.8	8.3	2.841634	6.89E-06	9.31E-05
TC1100009241.hg.1	HINFP	9.7	11.1	2.597400	6.92E-06	9.34E-05
TC2000007224.hg.1	SPAG4	4.9	6.5	3.148746	6.93E-06	9.34E-05
TC0600014300.hg.1	GCLC	10.6	12.1	2.864996	6.93E-06	9.34E-05
TC0100017548.hg.1	CDC42BPA	9.5	11.4	3.634007	6.96E-06	9.37E-05
TC0300011834.hg.1	FILIP1L	6.1	8.0	3.827159	6.97E-06	9.37E-05
TC0500010930.hg.1	TRIM23	7.6	9.4	3.516683	7.06E-06	9.47E-05
TC0500006737.hg.1	6-Mar	13.6	15.0	2.508938	7.09E-06	9.51E-05
TC1800008781.hg.1	ATP8B1	11.3	12.7	2.574270	7.10E-06	9.52E-05
TC0400011464.hg.1	MANBA	8.2	10.2	3.943575	7.14E-06	9.53E-05
TC1200010518.hg.1	SLC38A2	16.2	17.8	3.124575	7.14E-06	9.53E-05
TC0500013351.hg.1	FNIP1	10.8	12.6	3.441571	7.17E-06	9.56E-05
TC1900011919.hg.1	ZNF708	8.4	9.8	2.700924	7.18E-06	9.58E-05
TC0300012048.hg.1	ZBTB20; MIR568	10.3	11.7	2.659103	7.19E-06	9.58E-05
TC0100018286.hg.1	NBPF19	8.9	10.6	3.232559	7.20E-06	9.59E-05
TC1400008940.hg.1	NFKBIA	8.7	10.3	3.036691	7.21E-06	9.60E-05
TC2000009504.hg.1	ZNF217	13.4	14.8	2.636854	7.25E-06	9.63E-05
TC1900007085.hg.1	ZNF791	7.8	9.5	3.216200	7.26E-06	9.64E-05

TC1900006819.hg.1	TRIP10	12.3	13.8	2.776675	7.31E-06	9.69E-05
TC0100018403.hg.1	ERRFI1	15.4	16.7	2.582283	7.31E-06	9.69E-05
TC0500008849.hg.1	CYSTM1	11.4	12.9	2.732703	7.33E-06	9.71E-05
TC0200010840.hg.1	DNAJB2	6.0	8.6	5.915559	7.36E-06	9.74E-05
TC1100013194.hg.1	CORO1B	10.0	11.5	2.803985	7.39E-06	9.78E-05
TC0300013520.hg.1	CLDN1	10.1	11.7	2.917572	7.44E-06	9.83E-05
TC1400008118.hg.1	AK7	8.8	10.7	3.821341	7.45E-06	9.83E-05
TC0100015561.hg.1	NBPF15	10.9	12.3	2.531445	7.53E-06	9.90E-05
TC1900011940.hg.1	ECH1	12.8	14.1	2.461749	7.54E-06	9.91E-05
TC0100018454.hg.1	ARHGAP29	10.9	12.4	2.970054	7.63E-06	0.00010013
TC0600011464.hg.1	SLC44A4	4.1	6.3	4.549160	7.66E-06	0.00010051
TC1700011779.hg.1	ACOX1	10.6	12.0	2.543978	7.78E-06	0.0001019
TC0700010796.hg.1	VPS41	12.5	13.8	2.570171	7.90E-06	0.00010336
TC0100014755.hg.1	CTBS	8.0	9.3	2.566444	8.02E-06	0.0001047
TC0500013301.hg.1	DAB2	5.4	7.3	3.695420	8.16E-06	0.00010643
TC2200008710.hg.1	JOSD1	9.4	10.7	2.604951	8.20E-06	0.00010676
TC1900008433.hg.1	GLTSCR2; SNORD	6.9	8.3	2.714513	8.22E-06	0.00010687
TC1400010777.hg.1	TC2N	7.9	9.3	2.721883	8.25E-06	0.0001072
TC0500013020.hg.1	CLK4	8.5	10.0	2.775177	8.31E-06	0.00010784
TC1900011793.hg.1	ZNF808; RPL39P3	9.3	10.8	2.834148	8.34E-06	0.00010813
TC1100006820.hg.1	SWAP70	8.0	9.4	2.707428	8.34E-06	0.00010813
TC0600013998.hg.1	THBS2	7.5	9.1	3.082387	8.36E-06	0.0001083
TC0900010971.hg.1	TBC1D2	7.6	9.2	2.975117	8.37E-06	0.0001083
TC0200014717.hg.1	WDSUB1	6.3	7.8	2.701644	8.37E-06	0.0001083
TC0100018521.hg.1	TSTD1	6.9	8.8	3.841683	8.46E-06	0.00010901
TC1600010227.hg.1	N4BP1	10.9	12.3	2.553922	8.61E-06	0.00011079
TC0100015945.hg.1	THBS3	7.4	8.8	2.650890	8.70E-06	0.00011168
TC0200013902.hg.1	RGPD5; RGPD8	11.9	13.2	2.390851	8.77E-06	0.00011246
TC0700010019.hg.1	TMEM184A	9.5	10.9	2.666717	8.80E-06	0.00011266
TC1700011205.hg.1	C17orf67	6.6	8.5	3.558594	8.80E-06	0.00011266
TC0200008534.hg.1	ANKRD36	12.4	13.8	2.584897	8.80E-06	0.00011266
TC0400007270.hg.1	FAM114A1	3.5	5.2	3.408161	8.92E-06	0.00011387
TC0600010921.hg.1	ATXN1	9.1	10.4	2.476779	8.93E-06	0.00011396
TC1900009600.hg.1	FBXL12	5.7	7.8	4.103666	8.99E-06	0.00011465
TC0900010624.hg.1	ZCCHC6	10.0	11.4	2.695224	9.01E-06	0.00011482
TC0200013567.hg.1	ANKRD36B	11.0	12.5	2.890857	9.03E-06	0.00011506
TC1000009092.hg.1	INPP5F	8.7	10.0	2.370293	9.04E-06	0.00011509
TC0100014344.hg.1	MYSM1	11.5	12.9	2.536709	9.10E-06	0.00011557
TC0600011714.hg.1	BTBD9	5.0	6.9	3.815596	9.11E-06	0.00011557
TC0500007868.hg.1	SCAMP1	8.9	10.5	2.886089	9.11E-06	0.00011557
TC0600013740.hg.1	RSPH3	7.9	9.3	2.738869	9.11E-06	0.0001156
TC1600011516.hg.1	AC009133.12; PAG	5.6	7.2	3.094750	9.13E-06	0.0001157
TC1000007954.hg.1	SLC29A3	6.0	7.8	3.481676	9.17E-06	0.00011616
TC1000011726.hg.1	SH3PXD2A	6.4	7.9	2.786743	9.23E-06	0.00011677
TCUn_GL000219v100(	LOC283788; AL592	6.0	7.6	3.021070	9.26E-06	0.00011699
TC0200010897.hg.1	SGPP2	9.5	11.1	3.072485	9.35E-06	0.00011808
TSUnmapped0000005	ZNF780B	7.4	8.7	2.445293	9.38E-06	0.00011826
TSUnmapped0000017	ZNF780B	7.4	8.7	2.445293	9.38E-06	0.00011826
TC2200008687.hg.1	TMEM184B	11.4	12.8	2.663626	9.41E-06	0.00011861
TC0400011108.hg.1	CNOT6L	9.4	10.7	2.567411	9.43E-06	0.00011865

TC0300009651.hg.1	ATP11B	7.5	8.8	2.512657	9.49E-06	0.00011918
TC0300013123.hg.1	PLD1	11.0	12.4	2.626987	9.50E-06	0.0001192
TC1000010961.hg.1	PSAP	13.8	15.4	3.002142	9.51E-06	0.00011932
TC0500012354.hg.1	YIPF5	10.8	12.1	2.458601	9.55E-06	0.0001196
TC0900011819.hg.1	TSC1	11.5	12.7	2.384558	9.61E-06	0.00012027
TC1200011767.hg.1	SLC41A2	11.9	13.2	2.478532	9.63E-06	0.00012045
TC1500009358.hg.1	SECISBP2L	11.3	12.7	2.553920	9.68E-06	0.0001211
TC0700006618.hg.1	WIPI2	10.4	11.8	2.619141	9.77E-06	0.00012193
TC2000009239.hg.1	WFDC10B	4.2	6.0	3.312276	9.80E-06	0.00012219
TC0500007831.hg.1	F2R	8.4	9.9	2.787929	9.84E-06	0.00012264
TC0500009707.hg.1	SQSTM1	4.0	6.8	6.941874	1.00E-05	0.00012474
TC1100010760.hg.1	AGBL2	7.0	8.4	2.650423	1.00E-05	0.00012482
TC0300012195.hg.1	HSPBAP1	4.2	5.8	2.949074	1.01E-05	0.00012509
TC1100011867.hg.1	FZD4	6.0	7.3	2.502828	1.02E-05	0.00012593
TC0600012432.hg.1	PGM3	8.2	10.2	3.935688	1.02E-05	0.00012593
TC1100007220.hg.1	DEPDC7	7.5	8.8	2.533224	1.02E-05	0.00012671
TC2000009957.hg.1	LINC00266-1	7.1	8.7	2.991414	1.02E-05	0.00012688
TC1500010712.hg.1	GOLGA8N	6.4	7.7	2.529024	1.03E-05	0.00012746
TC0100008874.hg.1	PRKACB	10.4	11.7	2.404579	1.03E-05	0.00012762
TC1200010185.hg.1	ITPR2	11.4	12.9	2.860158	1.04E-05	0.00012794
TC2200008680.hg.1	BAIAP2L2	6.1	7.8	3.312369	1.04E-05	0.00012794
TC0100016406.hg.1	VAMP4	8.0	9.5	2.908478	1.04E-05	0.00012794
TC0200009905.hg.1	DHRS9	8.9	10.2	2.400570	1.04E-05	0.00012794
TC1600011354.hg.1	NPIPA2	9.8	11.1	2.496355	1.04E-05	0.00012794
TC0400008345.hg.1	SGMS2	8.4	9.8	2.487451	1.04E-05	0.00012794
TC0800011312.hg.1	RRM2B	8.4	10.1	3.207213	1.05E-05	0.00012876
TC0X00006789.hg.1	FAM3C2	15.3	16.7	2.694839	1.05E-05	0.0001288
TC1500010743.hg.1	MAPK6	11.8	13.1	2.477947	1.05E-05	0.00012922
TC1400010632.hg.1	GPATCH2L	9.8	11.2	2.576380	1.05E-05	0.00012922
TC1700012228.hg.1	LRRC37B	7.4	9.0	3.062941	1.05E-05	0.00012941
TC1500008893.hg.1	MTMR10	9.6	11.0	2.698125	1.06E-05	0.00012992
TC1000011892.hg.1	CCDC186; MIR211	9.3	10.7	2.685040	1.06E-05	0.00013022
TC1400007442.hg.1	ZBTB1	9.7	11.3	3.091325	1.07E-05	0.00013073
TC1900008240.hg.1	ZNF283	11.0	12.5	2.943532	1.07E-05	0.0001308
TC0700006488.hg.1	GPER1	8.7	10.3	3.078758	1.07E-05	0.00013093
TC0200013376.hg.1	EIF2AK3	7.3	9.0	3.223778	1.08E-05	0.00013143
TC1700008452.hg.1	GDPD1	3.5	5.2	3.270390	1.09E-05	0.00013236
TC1900008103.hg.1	PLD3	8.6	10.3	3.299368	1.09E-05	0.00013295
TC0100015594.hg.1	ITGA10	4.5	6.6	4.292202	1.10E-05	0.00013345
TC1500007084.hg.1	CASC4	10.8	12.1	2.397474	1.10E-05	0.00013345
TC1700010762.hg.1	VAT1	11.3	13.1	3.453281	1.10E-05	0.00013379
TC1700008032.hg.1	ACBD4	6.5	8.0	2.997415	1.11E-05	0.00013413
TC1100011723.hg.1	RSF1	10.4	11.8	2.631969	1.11E-05	0.00013421
TC1000012227.hg.1	CLRN3	8.2	9.6	2.571559	1.11E-05	0.00013429
TC1400009186.hg.1	ERO1A	15.5	16.8	2.524058	1.11E-05	0.0001347
TC0600008120.hg.1	TMEM63B	8.3	9.6	2.433223	1.12E-05	0.00013491
TC1600010006.hg.1	PRSS8	5.6	7.9	4.762634	1.13E-05	0.00013583
TC0700008574.hg.1	MUC3A	9.6	10.9	2.422912	1.13E-05	0.00013608
TC1400009184.hg.1	TXNDC16	3.9	6.3	5.047690	1.14E-05	0.00013692
TC0100015627.hg.1	PRKAB2	8.2	10.0	3.406099	1.15E-05	0.00013774

TC0700012165.hg.1	ATXN7L1	6.7	8.2	2.777920	1.15E-05	0.00013774
TC0100011692.hg.1	MIA3	7.8	9.1	2.351000	1.15E-05	0.00013774
TC1400007307.hg.1	ARID4A	8.4	10.1	3.166568	1.15E-05	0.0001381
TC1800007155.hg.1	PIK3C3	7.7	9.6	3.589294	1.16E-05	0.00013868
TC1400007093.hg.1	KLHDC1	4.8	6.4	3.080749	1.16E-05	0.00013957
TC0300011103.hg.1	HYAL1	7.3	9.0	3.391471	1.17E-05	0.00013992
TC1000007199.hg.1	ZEB1	11.5	13.1	3.018810	1.17E-05	0.00014032
TC1200011099.hg.1	GNS	13.6	14.9	2.477374	1.17E-05	0.00014032
TC1200010038.hg.1	EPS8	12.9	14.3	2.670852	1.18E-05	0.00014059
TC0700006795.hg.1	AHR	13.6	14.9	2.482495	1.18E-05	0.00014062
TC1600009322.hg.1	CARHSP1	7.1	8.3	2.431534	1.18E-05	0.00014064
TC1600010780.hg.1	CMTR2	10.3	11.7	2.714145	1.18E-05	0.00014079
TC0700008849.hg.1	FOXP2	7.4	8.8	2.729981	1.20E-05	0.00014221
TC1000012516.hg.1	AKR1C2	8.5	9.8	2.468079	1.20E-05	0.00014248
TC0600008303.hg.1	LRRC1	11.0	12.3	2.413147	1.21E-05	0.00014333
TC0700008517.hg.1	ZKSCAN1	11.8	13.4	2.914217	1.21E-05	0.00014361
TC0200007609.hg.1	SPTBN1	14.5	15.8	2.502536	1.22E-05	0.00014436
TC1000007625.hg.1	PRKG1	4.7	6.7	3.757988	1.22E-05	0.00014447
TC2000007283.hg.1	SRC	9.4	11.2	3.381417	1.23E-05	0.00014521
TC0100008621.hg.1	LEPR; LEPROT	7.8	9.6	3.377475	1.23E-05	0.00014521
TC1000008984.hg.1	FAM160B1	7.1	8.6	2.886639	1.25E-05	0.00014773
TC0700012115.hg.1	NAPEPLD	9.5	10.8	2.390053	1.25E-05	0.00014789
TC1900009754.hg.1	DNASE2	6.4	8.0	2.995394	1.26E-05	0.00014849
TC0100009442.hg.1	WNT2B	5.2	7.2	3.813420	1.26E-05	0.00014883
TC1900011755.hg.1	ZNF226	5.5	7.4	3.637720	1.27E-05	0.00014892
TC0800012422.hg.1	VCPIP1	9.8	11.1	2.602652	1.27E-05	0.00014894
TC2000008985.hg.1	RBM39	10.6	12.0	2.648719	1.27E-05	0.00014894
TC1600006537.hg.1	BAIAP3	5.2	7.0	3.464428	1.27E-05	0.00014967
TC0400007933.hg.1	ANXA3	8.8	10.2	2.699850	1.28E-05	0.00014967
TC0100006818.hg.1	PGD	10.6	12.1	2.803440	1.28E-05	0.00014972
TC1100008985.hg.1	ATM	12.8	14.1	2.381901	1.29E-05	0.00015048
TC1500010886.hg.1	CALML4	10.1	11.4	2.415524	1.29E-05	0.00015048
TC0700011796.hg.1	SAMD9	5.7	7.9	4.526358	1.29E-05	0.00015081
TC0100018356.hg.1	RHOU	8.3	9.7	2.651480	1.29E-05	0.00015081
TC0200008911.hg.1	ZC3H6	5.9	7.3	2.618217	1.30E-05	0.00015126
TC1100011643.hg.1	ARRB1	14.1	15.5	2.638320	1.32E-05	0.00015342
TC0200011445.hg.1	FAM110C	7.6	8.9	2.579216	1.32E-05	0.00015342
TC0200007048.hg.1	MAPRE3	5.2	6.7	2.885286	1.32E-05	0.00015375
TC1300007104.hg.1	LRCH1	9.3	10.5	2.271479	1.32E-05	0.00015384
TC1200010615.hg.1	LMBR1L	5.9	7.9	4.197106	1.34E-05	0.0001553
TC0100015872.hg.1	S100A14	5.4	7.5	4.161324	1.34E-05	0.00015545
TC1500009460.hg.1	FAM214A	3.6	5.5	3.695354	1.34E-05	0.00015573
TC0200007473.hg.1	CRIPT	10.0	11.3	2.459546	1.34E-05	0.00015573
TC1200008790.hg.1	USP30	8.5	10.3	3.418619	1.36E-05	0.00015696
TC0300010770.hg.1	CSRNP1	4.0	5.6	2.996435	1.36E-05	0.00015701
TC1000007471.hg.1	ALOX5	6.9	8.5	3.038670	1.36E-05	0.00015701
TC0X00007556.hg.1	FOXO4	3.9	5.9	4.032790	1.36E-05	0.00015701
TC1300009992.hg.1	BIVM-ERCC5	6.9	8.4	2.810823	1.36E-05	0.00015755
TC2100008560.hg.1	DONSON; CRYZL1	8.2	9.6	2.597548	1.37E-05	0.00015787
TC0900011286.hg.1	DFNB31	4.4	6.1	3.376126	1.37E-05	0.00015787

TC0Y00006882.hg.1	SLC25A6	13.1	14.4	2.395819	1.37E-05	0.00015787
TC0500009097.hg.1	SYNPO	4.7	6.3	3.048531	1.38E-05	0.00015878
TC1100012037.hg.1	MAML2	11.3	12.9	2.988728	1.38E-05	0.000159
TC1000012389.hg.1	ADAM8	6.9	8.8	3.708873	1.39E-05	0.00015923
TC0X00006966.hg.1	SYTL5	9.3	10.7	2.560234	1.39E-05	0.00015923
TC0200015559.hg.1	PLEKHM3	6.7	8.2	2.809823	1.40E-05	0.0001601
TC1200011599.hg.1	LTA4H	10.6	11.8	2.395674	1.41E-05	0.00016137
TC1100009962.hg.1	FAM160A2	11.3	12.6	2.519085	1.41E-05	0.00016137
TC0300013831.hg.1	SEMA3B; MIR6872	5.0	7.3	4.872339	1.42E-05	0.0001623
TC0100007102.hg.1	PADI1	6.3	8.1	3.445908	1.44E-05	0.00016439
TC0100013313.hg.1	MYOM3	5.7	8.0	4.948945	1.47E-05	0.00016701
TC0900008851.hg.1	DNM1	12.0	13.3	2.501834	1.48E-05	0.00016832
TC0800009673.hg.1	LONRF1	5.1	7.0	3.652063	1.53E-05	0.0001729
TC0100011566.hg.1	PROX1	3.2	4.9	3.047910	1.53E-05	0.0001735
TC0700006727.hg.1	TMEM106B	11.1	12.6	2.922560	1.53E-05	0.0001735
TC1900011327.hg.1	ZNF160	8.6	10.5	3.748334	1.54E-05	0.00017433
TC2200007037.hg.1	MTMR3	8.3	9.5	2.226539	1.55E-05	0.00017495
TC0X00006891.hg.1	GK	3.5	5.8	4.710760	1.55E-05	0.00017495
TC0100017368.hg.1	BPNT1	12.2	13.8	2.980118	1.55E-05	0.00017531
TC2000007184.hg.1	ITCH	11.9	13.2	2.492806	1.56E-05	0.00017566
TC1700008851.hg.1	GPRC5C	5.2	7.2	4.086840	1.56E-05	0.00017566
TC0100015254.hg.1	LRIF1	5.5	7.1	2.846078	1.56E-05	0.00017566
TC0300007474.hg.1	DNAH1	8.0	10.0	4.013567	1.56E-05	0.00017566
TC1500009597.hg.1	MYO1E	10.9	12.4	2.849270	1.57E-05	0.0001768
TC1000012429.hg.1	PRPF18	7.1	9.2	4.215012	1.58E-05	0.00017744
TC1700009528.hg.1	CXCL16	9.8	11.0	2.368384	1.58E-05	0.00017746
TC0300012216.hg.1	CCDC14	11.7	13.1	2.715274	1.58E-05	0.00017766
TC0200009902.hg.1	NOSTRIN	8.0	9.3	2.361512	1.58E-05	0.00017766
TC0300014095.hg.1	TM4SF19	5.5	6.8	2.439503	1.59E-05	0.00017861
TC2200007337.hg.1	MAFF	6.4	7.7	2.396487	1.60E-05	0.00017903
TC1900008562.hg.1	TBC1D17; MIR475(	6.9	8.9	3.842613	1.60E-05	0.00017937
TC0400011087.hg.1	CCNI	13.9	15.2	2.551000	1.61E-05	0.00017964
TC1000010101.hg.1	ABI1	11.0	12.4	2.611172	1.61E-05	0.00017971
TC0100010674.hg.1	GPR52	6.9	8.4	2.845869	1.61E-05	0.00017977
TC1600009620.hg.1	SMG1	14.9	16.1	2.243533	1.61E-05	0.00017994
TC1600010406.hg.1	AMFR	9.5	10.7	2.214410	1.61E-05	0.00018007
TC0100014392.hg.1	TM2D1	8.4	9.8	2.599767	1.63E-05	0.00018175
TC2100007072.hg.1	TTC3	10.9	12.2	2.442315	1.64E-05	0.0001818
TC0200011022.hg.1	SP140L	4.6	6.3	3.077113	1.64E-05	0.00018202
TC0200007803.hg.1	LGALSL	7.8	9.3	2.732693	1.64E-05	0.00018213
TC0300011403.hg.1	MAGI1	8.8	10.0	2.316703	1.64E-05	0.00018213
TC0300010913.hg.1	CDCP1	14.3	15.5	2.303498	1.64E-05	0.00018213
TC1100011222.hg.1	SLC25A45	7.2	9.2	4.169382	1.67E-05	0.00018498
TC0800007529.hg.1	SPIDR	9.7	11.0	2.515370	1.68E-05	0.00018557
TC0600013179.hg.1	OR2A4	4.8	6.5	3.111845	1.69E-05	0.00018601
TC0200016454.hg.1	KIAA1841	8.4	9.7	2.486191	1.69E-05	0.00018627
TC0300008602.hg.1	KALRN	5.0	6.5	2.900526	1.70E-05	0.00018673
TC1700010693.hg.1	ACLY	12.6	13.8	2.424096	1.70E-05	0.00018725
TC0900011259.hg.1	HDHD3	6.6	8.0	2.698517	1.71E-05	0.0001878
TC1600011360.hg.1	SYT17	7.9	9.7	3.612427	1.72E-05	0.00018844

TC0400011208.hg.1	FAM175A	8.6	9.9	2.378706	1.72E-05	0.00018859
TC0900010769.hg.1	NFIL3	6.1	7.4	2.450549	1.73E-05	0.00018951
TC1500008187.hg.1	AKAP13; MIR7706	11.1	12.4	2.469235	1.73E-05	0.00018951
TC0700013323.hg.1	SUN1	13.5	14.7	2.362830	1.74E-05	0.00018981
TC1800006710.hg.1	CHMP1B	9.3	10.5	2.306881	1.74E-05	0.00018982
TC1100011829.hg.1	CREBZF	9.5	10.8	2.477001	1.74E-05	0.00018982
TC0900012220.hg.1	CDKN2B	6.4	8.6	4.752073	1.74E-05	0.00019001
TC0800009872.hg.1	TNFRSF10D	10.8	12.0	2.386106	1.74E-05	0.00019001
TC0700012812.hg.1	MKRN1	11.6	12.9	2.468920	1.75E-05	0.00019084
TC0600012883.hg.1	TUBE1	9.7	10.9	2.310318	1.76E-05	0.00019161
TC0900011203.hg.1	PTGR1	8.3	10.0	3.106632	1.76E-05	0.00019161
TC0300007083.hg.1	WDR48	8.3	9.7	2.598412	1.77E-05	0.00019254
TC1300007592.hg.1	NDFIP2	9.7	11.4	3.403247	1.78E-05	0.00019304
TC0400011580.hg.1	CFI	7.2	8.8	3.207866	1.78E-05	0.00019364
TC0200013351.hg.1	RGPD1; RGPD2	9.4	10.7	2.523605	1.79E-05	0.00019435
TC0600011535.hg.1	RGL2	8.9	10.1	2.342079	1.80E-05	0.00019474
TC0100013049.hg.1	NBPF1	9.9	11.3	2.653902	1.81E-05	0.0001967
TC0800008801.hg.1	TRIB1	13.3	14.5	2.361120	1.83E-05	0.0001977
TC0800010945.hg.1	SLC10A5	4.9	6.5	2.942879	1.83E-05	0.00019777
TC1300008892.hg.1	LPAR6	5.5	6.9	2.662999	1.83E-05	0.00019785
TC0500008539.hg.1	GRAMD3	8.3	9.8	2.906068	1.83E-05	0.00019787
TC0200010263.hg.1	INPP1	10.1	11.3	2.342766	1.83E-05	0.00019813
TC0800006975.hg.1	BMP1	9.4	10.8	2.777039	1.84E-05	0.00019855
TC1700008326.hg.1	TOM1L1	8.6	9.9	2.358490	1.85E-05	0.00019888
TC0800010703.hg.1	ARFGEF1	8.1	9.4	2.522404	1.85E-05	0.00019895
TC1900010886.hg.1	ZNF235	6.0	7.2	2.393248	1.85E-05	0.00019972
TC0300007206.hg.1	TCAIM	10.2	11.5	2.413726	1.86E-05	0.00019982
TC1200012707.hg.1	ALDH2	10.7	12.0	2.315232	1.87E-05	0.00020112
TC0300006565.hg.1	IL17RC	8.1	9.5	2.585959	1.88E-05	0.00020148
TC1200006850.hg.1	ETV6	9.6	10.9	2.460584	1.88E-05	0.00020148
TC1000010497.hg.1	8-Mar	7.9	9.5	3.070288	1.89E-05	0.00020291
TC0800011861.hg.1	LRRC6	5.0	6.4	2.582376	1.89E-05	0.00020291
TC0700010604.hg.1	CPVL	9.9	11.3	2.615404	1.89E-05	0.0002031
TC0700008597.hg.1	CUX1	11.4	12.8	2.719101	1.91E-05	0.00020442
TC1200011873.hg.1	MMAB	9.1	10.4	2.483825	1.91E-05	0.00020456
TC0800011285.hg.1	ZNF706	11.2	12.5	2.460301	1.94E-05	0.00020769
TC0200006738.hg.1	LPIN1	4.0	6.6	6.298287	1.97E-05	0.00020978
TC1800008235.hg.1	ABHD3	5.9	7.4	2.776979	1.99E-05	0.00021221
TC1700007851.hg.1	EIF1	14.2	15.4	2.450163	2.01E-05	0.0002141
TC0100015580.hg.1	NBPF20	9.3	10.8	2.938043	2.03E-05	0.00021606
TC0700011503.hg.1	CLDN3	6.0	7.8	3.387053	2.04E-05	0.00021608
TC2000008123.hg.1	NRSN2-AS1	6.8	8.5	3.250293	2.04E-05	0.00021617
TC1900010521.hg.1	ZFP14	4.5	5.8	2.530707	2.05E-05	0.00021693
TC0X00008136.hg.1	ALG13	9.4	10.7	2.554178	2.05E-05	0.0002171
TC1400009134.hg.1	SAV1	7.9	9.1	2.300671	2.06E-05	0.00021768
TC0600008697.hg.1	NT5E	14.0	15.2	2.397362	2.06E-05	0.00021805
TC1100007808.hg.1	SDHAF2	7.0	8.4	2.680200	2.07E-05	0.00021866
TC1300008691.hg.1	FOXO1	5.0	7.0	4.194318	2.07E-05	0.00021866
TC0200008900.hg.1	TMEM87B	12.8	14.0	2.352236	2.07E-05	0.00021878
TC1400009649.hg.1	NUMB	11.6	12.9	2.305563	2.08E-05	0.00021878

TC0200007032.hg.1	HADHB	10.2	11.4	2.279945	2.08E-05	0.00021912
TC0700010749.hg.1	KIAA0895	6.9	8.5	3.023114	2.08E-05	0.00021913
TC0300006791.hg.1	KAT2B	6.4	8.0	3.010503	2.09E-05	0.00022009
TC0600012123.hg.1	DST	14.6	15.8	2.326923	2.10E-05	0.00022044
TC0700011562.hg.1	TMEM120A	8.7	10.3	2.903817	2.10E-05	0.00022054
TC1500008304.hg.1	GDPGP1	8.0	9.6	2.906888	2.11E-05	0.00022149
TC1300009725.hg.1	FAM155A	6.8	8.2	2.715838	2.13E-05	0.00022328
TC0900009859.hg.1	AQP7	3.5	5.1	2.917285	2.15E-05	0.00022459
TC1200012734.hg.1	ZNF26	5.8	7.4	3.193356	2.15E-05	0.0002247
TC1000008088.hg.1	SAMD8	9.4	10.7	2.348274	2.16E-05	0.00022518
TC1700008603.hg.1	CEP95	7.9	9.0	2.201216	2.19E-05	0.00022827
TC0X00008332.hg.1	STAG2	13.1	14.4	2.440007	2.20E-05	0.00022938
TC0200010489.hg.1	BMPR2	10.5	11.9	2.573895	2.20E-05	0.00022938
TC0500011896.hg.1	ZNF608	8.9	10.2	2.497360	2.21E-05	0.00022993
TC0600014256.hg.1	GABBR1	7.9	9.2	2.489088	2.23E-05	0.00023176
TC0100016035.hg.1	ETV3	10.7	12.2	2.785948	2.23E-05	0.0002323
TC0700013388.hg.1	STAG3L1	12.8	14.1	2.477297	2.24E-05	0.00023253
TC1600008164.hg.1	FAM65A	11.3	12.6	2.369732	2.24E-05	0.00023253
TC1600011355.hg.1	NPIPA1	9.6	11.0	2.653930	2.25E-05	0.00023324
TC1100012574.hg.1	SORL1	9.2	10.5	2.536765	2.28E-05	0.00023651
TC1700008690.hg.1	PITPNC1	10.2	11.6	2.621255	2.31E-05	0.0002386
TC2200008630.hg.1	TST	6.5	8.0	2.803253	2.32E-05	0.00023906
TC1100013088.hg.1	BIRC2	9.0	10.5	2.849841	2.33E-05	0.00024045
TC1900011752.hg.1	ZNF223	4.6	6.5	3.824250	2.34E-05	0.00024067
TC2000010009.hg.1	BLCAP	7.5	8.9	2.567311	2.34E-05	0.00024095
TC1700012242.hg.1	LOC101060389; TE	6.7	8.4	3.244049	2.36E-05	0.00024348
TC1000012511.hg.1	PRAP1	8.4	10.1	3.258987	2.37E-05	0.00024369
TC0700013629.hg.1	BRAF	10.7	12.1	2.621468	2.37E-05	0.00024409
TC1900011312.hg.1	ZNF611	12.1	13.3	2.386350	2.38E-05	0.00024447
TC0100012816.hg.1	CLSTN1	11.9	13.1	2.314410	2.39E-05	0.00024556
TC0400009029.hg.1	FHDC1	5.7	7.3	2.997983	2.39E-05	0.00024556
TC0200015865.hg.1	SCG2	6.1	7.5	2.673915	2.40E-05	0.00024622
TC0X00008908.hg.1	SLC25A6	13.2	14.3	2.181857	2.40E-05	0.00024622
TC0400012856.hg.1	RAPGEF2	5.9	7.2	2.464777	2.42E-05	0.00024819
TC0700010692.hg.1	NT5C3A	10.7	12.0	2.444252	2.43E-05	0.00024836
TC1200009796.hg.1	SLC2A14	6.6	7.7	2.145642	2.44E-05	0.00024932
TC0200014719.hg.1	BAZ2B	9.9	11.2	2.562933	2.44E-05	0.00024979
TC0500007201.hg.1	OSMR	10.4	11.6	2.204507	2.45E-05	0.00025041
TC0200013111.hg.1	DQX1	5.2	7.5	5.041635	2.46E-05	0.00025041
TC1100013209.hg.1	PAK1	8.6	9.8	2.275465	2.48E-05	0.00025288
TC0100013129.hg.1	AKR7A3	8.4	9.7	2.596312	2.49E-05	0.00025377
TC1200012745.hg.1	C1RL	7.5	9.5	4.004984	2.50E-05	0.00025436
TC0300012233.hg.1	ITGB5	11.2	12.4	2.265782	2.52E-05	0.00025651
TC0100017472.hg.1	WDR26	5.9	7.1	2.315197	2.53E-05	0.00025719
TC0500008919.hg.1	RNF14	10.0	11.2	2.312082	2.53E-05	0.00025741
TC2200008449.hg.1	DUSP18	5.8	7.2	2.806444	2.54E-05	0.00025793
TC0X00010329.hg.1	TSPAN6	11.1	12.5	2.513128	2.55E-05	0.00025892
TC1000007333.hg.1	ZNF33A	9.9	11.1	2.380910	2.57E-05	0.00026006
TC1300010006.hg.1	MCF2L	5.5	7.2	3.289781	2.58E-05	0.00026103
TC0200013836.hg.1	10-Sep	10.5	11.7	2.319299	2.59E-05	0.00026136

TC0900010284.hg.1	ANKRD20A1; ANKI	5.7	7.1	2.591877	2.59E-05	0.00026136
TC0100010903.hg.1	RNF2	8.0	9.3	2.392636	2.60E-05	0.00026171
TC0700008690.hg.1	KMT2E	11.7	12.9	2.342233	2.60E-05	0.00026207
TC1900011172.hg.1	VRK3	10.3	11.5	2.245521	2.62E-05	0.00026366
TC0100018499.hg.1	MUC1	10.4	11.5	2.181011	2.63E-05	0.00026416
TC1600007037.hg.1	NPIPA7	10.8	12.2	2.622787	2.63E-05	0.00026444
TC0800011216.hg.1	STK3	10.5	11.8	2.440383	2.64E-05	0.00026495
TC0900010762.hg.1	AUH	8.7	9.9	2.304837	2.65E-05	0.00026601
TC0800009231.hg.1	GRINA	8.3	10.3	4.117615	2.66E-05	0.00026696
TC0100011205.hg.1	ELF3	10.6	12.1	2.947947	2.68E-05	0.00026818
TC1700006646.hg.1	MINK1	7.2	8.5	2.412561	2.69E-05	0.00026979
TC0X00006715.hg.1	SCML1	6.5	8.7	4.766325	2.70E-05	0.00027012
TC1100012165.hg.1	CASP4	8.2	9.9	3.074211	2.72E-05	0.00027167
TC1200006445.hg.1	B4GALNT3	6.3	7.8	2.813269	2.76E-05	0.00027548
TC0700008582.hg.1	SERPINE1	6.5	8.0	2.840474	2.76E-05	0.00027569
TC1100013205.hg.1	ARAP1	5.3	6.8	2.758167	2.78E-05	0.00027735
TC0200007401.hg.1	DYNC2LI1	5.7	7.4	3.249089	2.78E-05	0.00027735
TC0200012734.hg.1	USP34	10.4	11.7	2.460484	2.79E-05	0.00027748
TC1100007257.hg.1	CAT	6.8	8.0	2.204975	2.79E-05	0.00027781
TC0100015786.hg.1	POGZ	12.5	13.6	2.232099	2.79E-05	0.00027797
TC0200011362.hg.1	RNPEPL1	8.4	9.8	2.608279	2.80E-05	0.00027876
TC2000007498.hg.1	SPINT4	3.6	4.8	2.274772	2.81E-05	0.00027896
TC1300009933.hg.1	RASA3	9.9	11.8	3.656511	2.82E-05	0.00027987
TC0300012118.hg.1	TMEM39A	10.2	11.3	2.176874	2.82E-05	0.00027988
TC1300007197.hg.1	DLEU1	6.2	8.2	3.805680	2.82E-05	0.00027988
TC0200008894.hg.1	MERTK	7.2	9.0	3.602377	2.82E-05	0.00027988
TC1100009287.hg.1	ARHGEF12	12.8	13.9	2.114622	2.84E-05	0.00028106
TC0300011019.hg.1	PLXNB1	4.8	6.0	2.223936	2.85E-05	0.00028217
TC0200007825.hg.1	CEP68	7.6	9.0	2.551203	2.90E-05	0.00028629
TC0200016611.hg.1	GPR35	7.3	8.7	2.569739	2.90E-05	0.00028667
TC1400006742.hg.1	KHNYN	8.5	9.8	2.448120	2.91E-05	0.00028701
TC0500009039.hg.1	FBXO38	9.8	11.2	2.529130	2.91E-05	0.00028701
TC0700013578.hg.1	SEMA3A	12.1	13.5	2.694553	2.91E-05	0.00028701
TC1100011050.hg.1	FADS1; MIR1908	7.8	9.4	3.022610	2.92E-05	0.00028732
TC1000010642.hg.1	A1CF	9.6	10.7	2.140161	2.92E-05	0.00028785
TC0700013050.hg.1	KCNH2	5.7	7.2	2.906143	2.96E-05	0.00029133
TC0700011519.hg.1	STAG3L2	13.0	14.2	2.258225	2.98E-05	0.00029274
TC1000009516.hg.1	DIP2C	7.6	8.8	2.261346	2.98E-05	0.00029346
TC0400011685.hg.1	PRSS12	9.9	11.0	2.247239	2.99E-05	0.00029438
TC0100018438.hg.1	MOB3C	5.5	6.6	2.143345	3.00E-05	0.00029473
TC1900011683.hg.1	HSH2D	4.2	5.8	2.999860	3.02E-05	0.00029614
TC1900008245.hg.1	ZNF221	3.6	4.9	2.482434	3.04E-05	0.00029738
TC1900011652.hg.1	ZNF358	7.3	8.8	2.736742	3.04E-05	0.00029751
TC0800010016.hg.1	SARAF	8.0	9.4	2.502451	3.04E-05	0.00029768
TC0100013611.hg.1	BSDC1	7.4	8.8	2.508566	3.05E-05	0.000298
TC0500010085.hg.1	CMBL	11.0	12.1	2.148166	3.07E-05	0.00029992
TC0900008945.hg.1	GPR107	10.5	11.6	2.149948	3.07E-05	0.00029992
TC0X00010671.hg.1	LAMP2	11.2	12.3	2.146207	3.07E-05	0.00029994
TC0300007165.hg.1	NKTR	12.1	13.3	2.331791	3.08E-05	0.00030005
TC0700006735.hg.1	SCIN	3.1	5.6	5.472891	3.08E-05	0.00030005

TC0100014214.hg.1	ECHDC2	9.5	11.1	3.185776	3.08E-05	0.00030005
TC1100010268.hg.1	TSG101	10.1	11.2	2.244851	3.08E-05	0.00030005
TC0300013991.hg.1	TWF2	8.4	9.8	2.605676	3.10E-05	0.00030199
TC0500013205.hg.1	CAST	10.6	11.8	2.256355	3.11E-05	0.00030258
TC0700013428.hg.1	PILRB; STAG3L5P	9.5	10.8	2.552902	3.12E-05	0.00030259
TC0200016402.hg.1	RSAD2	5.1	6.4	2.443090	3.17E-05	0.00030771
TC1800007911.hg.1	MYOM1	4.0	5.4	2.641919	3.17E-05	0.00030774
TC1000012575.hg.1	ATAD1	11.6	12.8	2.442933	3.19E-05	0.00030962
TC1700010563.hg.1	CACNB1	7.0	9.0	4.046569	3.20E-05	0.00030962
TC0300011450.hg.1	TMF1	8.2	9.6	2.538959	3.22E-05	0.00031144
TC0700011710.hg.1	SLC25A40	10.7	11.8	2.187210	3.25E-05	0.00031443
TC0100007784.hg.1	AGO4	6.2	7.9	3.156809	3.26E-05	0.00031489
TC1700012243.hg.1	TBC1D3E	7.9	9.7	3.623260	3.27E-05	0.00031526
TC1900007777.hg.1	CEBPG	9.9	11.1	2.239105	3.29E-05	0.00031729
TC0100011153.hg.1	C1orf106	10.8	12.0	2.304155	3.30E-05	0.00031786
TC0900011163.hg.1	PTPN3	10.0	11.3	2.408609	3.31E-05	0.00031816
TC2000008473.hg.1	KIF16B	10.2	11.4	2.279624	3.31E-05	0.00031843
TC0100013998.hg.1	HECTD3	7.8	9.2	2.759765	3.31E-05	0.00031882
TC1400006652.hg.1	ABHD4	5.1	6.4	2.586001	3.34E-05	0.000321
TC0300007610.hg.1	PXK	7.6	9.3	3.150662	3.34E-05	0.000321
TC0700013599.hg.1	ACHE	6.8	8.5	3.278414	3.35E-05	0.0003211
TC1400009006.hg.1	TRAPPC6B	9.2	10.7	2.731658	3.36E-05	0.00032188
TC1100013145.hg.1	COPB1	10.5	11.7	2.238613	3.36E-05	0.00032193
TC0100015793.hg.1	POGZ	6.7	7.8	2.150689	3.38E-05	0.00032322
TC1100009666.hg.1	SIGIRR	7.9	9.2	2.518947	3.39E-05	0.00032455
TC1300009714.hg.1	ARGLU1	10.5	11.6	2.237635	3.43E-05	0.00032734
TC0100008336.hg.1	SCP2	10.2	11.2	2.095664	3.43E-05	0.00032734
TC1700006642.hg.1	TM4SF5	6.1	7.7	2.908659	3.44E-05	0.00032776
TC1000008758.hg.1	CNNM2	6.8	9.0	4.818600	3.48E-05	0.00033122
TC0900012225.hg.1	RNF38	11.9	13.0	2.233745	3.50E-05	0.00033249
TC0800010944.hg.1	IMPA1	9.2	10.3	2.138207	3.50E-05	0.00033249
TC1900008543.hg.1	FCGRT	7.0	8.8	3.495108	3.51E-05	0.00033355
TC1500007833.hg.1	PML	8.3	9.5	2.284407	3.57E-05	0.00033818
TC1500008933.hg.1	GOLGA8O	5.6	6.8	2.303934	3.58E-05	0.00033934
TC1900008860.hg.1	LENG8	12.5	13.5	2.130326	3.61E-05	0.00034217
TC0100017116.hg.1	C1orf116	10.5	11.8	2.352727	3.62E-05	0.00034264
TC0900007006.hg.1	PRSS3	10.5	11.6	2.218864	3.63E-05	0.00034341
TC1000008587.hg.1	ZFYVE27	8.8	10.0	2.340152	3.65E-05	0.00034495
TC0100009199.hg.1	MFSD14A	10.6	11.9	2.374149	3.65E-05	0.00034533
TC0400011791.hg.1	ANKRD50	10.3	11.5	2.346997	3.68E-05	0.00034741
TC0800012312.hg.1	SGK3	7.7	9.0	2.347255	3.69E-05	0.00034755
TC1000008447.hg.1	TNKS2	11.0	12.2	2.389853	3.75E-05	0.00035261
TC0900010063.hg.1	CNTNAP3	5.3	6.5	2.299685	3.75E-05	0.00035305
TC1200011719.hg.1	CCDC53	8.5	9.9	2.496074	3.75E-05	0.00035305
TC2200008734.hg.1	CBX7	5.7	7.0	2.450060	3.76E-05	0.00035316
TC2000010002.hg.1	NCOA6	11.1	12.3	2.223490	3.79E-05	0.0003566
TC0900009437.hg.1	AK3	8.3	9.6	2.472832	3.80E-05	0.00035688
TC0200008099.hg.1	HK2	7.5	9.0	2.759485	3.80E-05	0.00035699
TC1500008100.hg.1	WHAMM	6.9	8.5	2.955987	3.81E-05	0.00035742
TC0500008700.hg.1	SEC24A	9.9	11.0	2.102720	3.84E-05	0.00035997

TC0600008665.hg.1	RWDD2A	4.6	6.1	2.655657	3.84E-05	0.00035997
TC1400010728.hg.1	HEATR5A	6.8	7.9	2.088875	3.84E-05	0.00036
TC1000008396.hg.1	IFIT2	6.8	8.5	3.234677	3.87E-05	0.00036172
TC0200010328.hg.1	SLC39A10	11.8	13.2	2.624040	3.89E-05	0.00036384
TC1100012949.hg.1	IFITM1	10.6	11.8	2.302318	3.91E-05	0.00036523
TC2000009966.hg.1	FKBP1A-SDCBP2	7.6	9.8	4.795890	3.92E-05	0.00036552
TC1100013148.hg.1	PIK3C2A	11.8	12.9	2.187156	3.97E-05	0.00036982
TC2000008499.hg.1	RRBP1	10.1	11.5	2.774523	3.97E-05	0.00036988
TC0600007495.hg.1	HLA-A	11.7	13.1	2.594333	3.99E-05	0.00037129
TC0300010521.hg.1	THRB	8.1	9.1	2.119557	3.99E-05	0.00037141
TC0100015990.hg.1	PAQR6	4.1	5.6	2.941720	3.99E-05	0.00037141
TC0900011151.hg.1	TMEM245	8.3	9.6	2.425885	4.01E-05	0.00037301
TC1000010844.hg.1	RUFY2	11.9	13.2	2.499409	4.03E-05	0.00037407
TC0700010437.hg.1	RAPGEF5	9.1	10.8	3.228356	4.04E-05	0.00037497
TC1200010971.hg.1	DCTN2	9.1	10.8	3.134052	4.06E-05	0.00037643
TC0500011049.hg.1	NAIP	7.4	9.1	3.265204	4.08E-05	0.00037778
TC0700010005.hg.1	ZFAND2A	7.6	8.8	2.238724	4.08E-05	0.00037778
TC0300013420.hg.1	IGF2BP2	11.6	12.7	2.238976	4.11E-05	0.00038034
TC1700010640.hg.1	KRT20	5.4	6.9	2.879845	4.15E-05	0.00038347
TC0100013999.hg.1	ZSWIM5	9.0	10.1	2.154564	4.16E-05	0.00038414
TC0X00009648.hg.1	TFE3	7.6	8.7	2.179689	4.17E-05	0.00038423
TC1900010851.hg.1	ETHE1	9.3	10.3	2.033595	4.19E-05	0.00038599
TC0100011272.hg.1	ZBED6; ZC3H11A	13.5	14.7	2.165626	4.20E-05	0.000387
TC0600007402.hg.1	ZKSCAN8	9.6	10.8	2.426186	4.20E-05	0.00038724
TC2100006967.hg.1	IFNGR2	13.2	14.5	2.466694	4.21E-05	0.00038749
TC0700008321.hg.1	ANKIB1	12.2	13.4	2.205366	4.21E-05	0.00038749
TC0800012206.hg.1	DGAT1; MIR6848	14.2	15.3	2.087297	4.22E-05	0.00038795
TC0600014264.hg.1	DDAH2	9.4	10.6	2.250019	4.23E-05	0.00038854
TC1600011353.hg.1	NPIPA3	9.8	10.9	2.136433	4.23E-05	0.00038872
TC0700013575.hg.1	STAG3L3; STAG3L	12.7	13.9	2.277963	4.25E-05	0.00039042
TC0900007547.hg.1	C9orf85	8.2	9.4	2.301950	4.27E-05	0.00039197
TC2000007157.hg.1	CBFA2T2	8.2	9.7	2.847818	4.28E-05	0.00039211
TC1000010411.hg.1	ZNF33B	9.3	10.7	2.545339	4.28E-05	0.00039237
TC0100009770.hg.1	NBPF12	10.3	11.9	3.041642	4.30E-05	0.00039411
TC0200016475.hg.1	INO80B	7.6	8.9	2.361574	4.31E-05	0.00039438
TC1100012959.hg.1	TIMM10B	10.8	12.1	2.476427	4.32E-05	0.00039551
TC0700009174.hg.1	MKLN1	11.5	12.7	2.287732	4.33E-05	0.00039614
TC0600011661.hg.1	STK38	10.1	11.2	2.269556	4.34E-05	0.00039698
TC1400009711.hg.1	NEK9	10.1	11.1	2.077863	4.35E-05	0.00039698
TC0X00008055.hg.1	TBC1D8B	8.6	10.1	2.715246	4.36E-05	0.00039784
TC0X00008235.hg.1	LONRF3	6.4	7.8	2.554284	4.36E-05	0.00039813
TC2100008165.hg.1	BRWD1	9.7	11.1	2.622741	4.37E-05	0.0003989
TC1100011539.hg.1	NUMA1	12.0	13.3	2.586281	4.38E-05	0.0003992
TC1800007354.hg.1	POLI	5.5	6.8	2.597565	4.43E-05	0.00040293
TC1200009111.hg.1	CCDC64	5.2	7.0	3.468915	4.43E-05	0.00040313
TC2200007356.hg.1	KDELR3	8.2	9.6	2.531268	4.46E-05	0.00040461
TC1600009524.hg.1	NPIPA5	9.7	10.8	2.187467	4.46E-05	0.00040471
TC0400007345.hg.1	LIMCH1	7.1	9.1	3.811694	4.46E-05	0.00040471
TC0600012839.hg.1	CDK19	10.0	11.4	2.700680	4.47E-05	0.00040471
TC1700008263.hg.1	ABCC3	14.2	15.4	2.317630	4.47E-05	0.00040471

TC0600008069.hg.1	KLC4	5.8	7.4	3.094049	4.48E-05	0.00040596
TC0200015009.hg.1	ATF2	13.4	14.7	2.526918	4.49E-05	0.00040596
TC1400010015.hg.1	ITPK1	12.5	13.6	2.049055	4.50E-05	0.00040705
TC0200015506.hg.1	INO80D	11.1	12.3	2.237389	4.54E-05	0.00041007
TC1600011483.hg.1	LITAF	9.8	10.9	2.112566	4.54E-05	0.00041013
TC0400006990.hg.1	MED28	9.5	10.8	2.405365	4.57E-05	0.0004126
TC0700013338.hg.1	GLCCI1	5.1	6.8	3.204189	4.60E-05	0.00041495
TC0900011803.hg.1	SETX	8.8	10.1	2.408627	4.63E-05	0.0004175
TC0X00009638.hg.1	OTUD5	8.8	10.0	2.194807	4.63E-05	0.0004175
TC1900008143.hg.1	AXL	12.0	13.2	2.178015	4.63E-05	0.00041787
TC0600007084.hg.1	RBM24	6.9	8.6	3.302969	4.65E-05	0.00041862
TC1300006923.hg.1	NHLRC3	8.0	9.6	2.955872	4.65E-05	0.00041863
TC0800011105.hg.1	TRIQK	8.4	10.3	3.711120	4.68E-05	0.00042052
TC0800012474.hg.1	AGO2	11.4	12.6	2.287745	4.69E-05	0.00042164
TC0200007237.hg.1	CRIM1	12.2	13.5	2.333629	4.71E-05	0.00042302
TC1500007633.hg.1	SMAD3	13.5	14.5	2.125364	4.72E-05	0.00042398
TC0700008450.hg.1	LMTK2	14.9	16.1	2.225870	4.75E-05	0.00042643
TC1100010216.hg.1	PLEKHA7	6.6	8.1	2.819265	4.76E-05	0.00042752
TC1400009579.hg.1	ADAM20	3.1	5.1	3.834701	4.80E-05	0.00043005
TC0700007137.hg.1	BBS9	4.0	6.5	5.647091	4.87E-05	0.0004354
TC1200011567.hg.1	TMCC3; MIR7844	8.7	9.8	2.163398	4.88E-05	0.00043598
TC1500008982.hg.1	KATNBL1	7.7	9.2	2.857954	4.90E-05	0.00043715
TC1000007761.hg.1	ARID5B	10.0	11.2	2.253443	4.91E-05	0.00043862
TC1600010792.hg.1	PHLPP2	11.1	12.6	2.758708	4.95E-05	0.00044122
TC0700006567.hg.1	TTYH3	10.2	11.3	2.150711	4.96E-05	0.00044223
TC0X00009153.hg.1	CTPS2	9.6	10.6	2.100503	4.99E-05	0.00044405
TC1100008886.hg.1	TMEM133	7.8	8.9	2.214080	5.00E-05	0.00044521
TC0500013392.hg.1	CCNJL	4.6	6.3	3.064411	5.03E-05	0.0004472
TC0800009094.hg.1	DENND3	7.4	9.2	3.294596	5.03E-05	0.00044721
TC1600010184.hg.1	ITFG1	7.0	8.2	2.206676	5.06E-05	0.00044965
TC0200009049.hg.1	INSIG2	10.5	11.6	2.178876	5.11E-05	0.00045228
TC1500009623.hg.1	BNIP2	7.3	8.7	2.673796	5.13E-05	0.00045412
TC2000007895.hg.1	RAB22A	11.9	13.0	2.166336	5.16E-05	0.00045646
TC1000008556.hg.1	LCOR	11.5	12.6	2.146086	5.18E-05	0.00045816
TC0X00010462.hg.1	RBM41	8.9	10.2	2.479236	5.18E-05	0.00045816
TC1700011319.hg.1	APPBP2	8.4	9.5	2.171410	5.19E-05	0.00045835
TC0300008705.hg.1	ABTB1	4.6	7.2	5.893835	5.19E-05	0.00045884
TC2200007242.hg.1	APOL1	4.6	7.5	7.305171	5.20E-05	0.00045896
TC1900008498.hg.1	NUCB1	11.4	12.5	2.155027	5.23E-05	0.00046117
TC0100007574.hg.1	EPB41	8.4	9.8	2.575767	5.24E-05	0.00046184
TC1700012344.hg.1	GABARAP	10.0	11.1	2.082287	5.27E-05	0.00046353
TC1800006487.hg.1	SMCHD1	10.1	11.2	2.079502	5.27E-05	0.00046353
TC1000010172.hg.1	SVIL	6.6	8.0	2.633245	5.29E-05	0.0004651
TC1000011026.hg.1	USP54	9.4	10.4	2.003960	5.29E-05	0.00046518
TC1700008254.hg.1	ACSF2	6.1	7.5	2.653830	5.31E-05	0.00046659
TC2000007509.hg.1	CTSA	15.5	16.7	2.225834	5.31E-05	0.00046666
TC1000010117.hg.1	ACBD5	11.3	12.4	2.097688	5.36E-05	0.0004699
TC0200015434.hg.1	ALS2	8.1	9.2	2.136626	5.40E-05	0.00047312
TC0600014154.hg.1	SLC35A1	7.1	8.4	2.506059	5.43E-05	0.00047564
TC1300007774.hg.1	MBNL2	11.9	13.1	2.371326	5.44E-05	0.00047619

TC0100007372.hg.1	NCMAP	6.5	7.9	2.642929	5.46E-05	0.00047784
TC0700012296.hg.1	C7orf60	4.5	6.4	3.643068	5.47E-05	0.00047784
TC0700007596.hg.1	EGFR	14.2	15.4	2.189362	5.48E-05	0.00047885
TC1000012427.hg.1	AKR1C1	10.2	11.6	2.672553	5.50E-05	0.00048017
TC0100015680.hg.1	NBPF9	9.0	10.2	2.328671	5.50E-05	0.00048054
TC0600009836.hg.1	IYD	3.6	5.1	2.811842	5.51E-05	0.00048056
TC1800007797.hg.1	ATP9B	7.7	8.8	2.163240	5.54E-05	0.00048298
TC0700012427.hg.1	FAM3C	16.1	17.4	2.483647	5.57E-05	0.00048493
TC1600006628.hg.1	KCTD5	14.4	15.6	2.196045	5.58E-05	0.00048575
TC0900008587.hg.1	TLR4	6.9	9.0	4.169558	5.61E-05	0.00048825
TC0700013383.hg.1	RABGEF1	11.6	12.7	2.278437	5.61E-05	0.00048836
TC1200008739.hg.1	TMEM263	7.1	8.4	2.473156	5.63E-05	0.00048903
TC1000009063.hg.1	FAM45A	9.6	11.0	2.581436	5.64E-05	0.00048999
TC1600008228.hg.1	CYB5B	16.1	17.3	2.243587	5.65E-05	0.00049026
TC1100009453.hg.1	ST3GAL4	8.5	9.6	2.119407	5.68E-05	0.00049264
TC0900010886.hg.1	PTCH1	9.1	10.2	2.106895	5.73E-05	0.00049697
TC0800008146.hg.1	WWP1	7.2	9.0	3.607287	5.76E-05	0.00049899
TC0700011504.hg.1	WBSCR27	4.7	6.1	2.563868	5.76E-05	0.00049899
TC0800012256.hg.1	CLN8	7.8	9.2	2.717338	5.79E-05	0.00050051
TC2000008755.hg.1	ZNF337	7.0	9.3	4.722156	5.79E-05	0.00050087
TC0100010381.hg.1	UAP1	8.4	9.7	2.458024	5.82E-05	0.00050329
TC1100006484.hg.1	EPS8L2	9.7	11.0	2.511934	5.87E-05	0.00050711
TC0100007403.hg.1	TMEM57	9.1	10.2	2.124056	5.87E-05	0.00050711
TC0800011129.hg.1	CDH17	16.7	17.8	2.294268	5.88E-05	0.00050711
TC0100011549.hg.1	FLVCR1	9.3	10.5	2.342014	5.88E-05	0.00050767
TC1600011378.hg.1	MVP; PAGR1	11.0	12.2	2.364231	5.91E-05	0.00050921
TC0400011057.hg.1	SCARB2	8.1	9.3	2.310567	5.91E-05	0.00050921
TC0100009959.hg.1	TUFT1	7.2	8.8	2.894592	5.94E-05	0.00051162
TC0100014457.hg.1	JAK1	11.2	12.2	2.017385	5.98E-05	0.00051468
TC1900012058.hg.1	ZNF417	10.1	11.2	2.055539	5.99E-05	0.00051495
TC0600008050.hg.1	UBR2	10.2	11.5	2.523741	6.02E-05	0.00051728
TC1000012121.hg.1	OAT	8.6	10.0	2.614217	6.04E-05	0.00051871
TC0500008014.hg.1	RASA1	9.7	10.8	2.059358	6.04E-05	0.00051871
TC0X00009869.hg.1	ARHGEF9; ARHGE	11.7	12.8	2.214572	6.08E-05	0.00052148
TC1600011440.hg.1	OSGIN1	7.9	9.1	2.367334	6.09E-05	0.0005219
TC0100017834.hg.1	GNG4	12.2	13.4	2.278754	6.11E-05	0.00052351
TC0200015922.hg.1	TM4SF20	4.2	6.1	3.694327	6.14E-05	0.00052528
TC0400011815.hg.1	MFSD8	9.6	10.7	2.120018	6.16E-05	0.0005269
TC1100012681.hg.1	SIAE	11.0	12.2	2.365288	6.17E-05	0.00052716
TC1600007030.hg.1	NPIPA7; NPIPA8; F	10.3	11.5	2.202687	6.19E-05	0.00052821
TC0200013875.hg.1	RGPD8; RGPD6	11.6	12.7	2.132289	6.19E-05	0.00052821
TC1400010779.hg.1	ATXN3	5.3	6.6	2.431696	6.20E-05	0.00052854
TC1000006924.hg.1	ARL5B	11.3	12.7	2.601344	6.21E-05	0.00052942
TC0100015794.hg.1	POGZ	5.8	6.9	2.141424	6.23E-05	0.00053086
TC1800008287.hg.1	ANKRD29	3.9	5.0	2.120822	6.25E-05	0.00053191
TC0100007552.hg.1	SESN2	5.2	6.3	2.156789	6.27E-05	0.00053349
TC1200007137.hg.1	FGFR1OP2	8.3	9.3	2.112600	6.28E-05	0.00053459
TC0400008668.hg.1	LARP1B	8.0	9.4	2.669046	6.29E-05	0.00053459
TC1900011934.hg.1	ZNF585B	10.4	11.6	2.414888	6.33E-05	0.0005383
TC0200011688.hg.1	ADAM17	10.4	11.6	2.283675	6.36E-05	0.0005405

TC1600006636.hg.1	PRSS21	11.6	12.7	2.084451	6.37E-05	0.00054089
TC0800010299.hg.1	RNF170; MIR4469	8.4	9.8	2.616732	6.38E-05	0.00054089
TC1700011922.hg.1	LGALS3BP	11.9	13.1	2.346636	6.39E-05	0.00054193
TC2000007114.hg.1	KIF3B	10.2	11.2	2.051989	6.39E-05	0.00054206
TC1600011315.hg.1	FAM234A; ARHGD	14.9	16.0	2.147440	6.44E-05	0.00054552
TC1200007820.hg.1	RAB5B	11.2	12.3	2.118575	6.45E-05	0.00054552
TC1100010089.hg.1	RNF141	12.4	13.6	2.287664	6.45E-05	0.00054584
TC1000007024.hg.1	KIAA1217	8.3	9.5	2.212622	6.53E-05	0.00055101
TC2100007039.hg.1	DOPEY2	6.2	7.8	3.146528	6.54E-05	0.00055195
TC0300012918.hg.1	CCNL1	10.4	11.5	2.133487	6.55E-05	0.00055224
TC0700008004.hg.1	GTF2IRD1	8.6	9.8	2.236722	6.56E-05	0.00055328
TC1200007653.hg.1	NR4A1	5.8	8.7	7.285694	6.57E-05	0.00055343
TC1100013060.hg.1	MYEOV	10.2	11.3	2.016670	6.59E-05	0.00055486
TC0300009597.hg.1	PIK3CA	9.8	11.4	2.953827	6.63E-05	0.00055774
TC0500011759.hg.1	ATG12	10.4	11.7	2.458000	6.63E-05	0.00055774
TC0X00009218.hg.1	RPS6KA3	10.9	12.3	2.545314	6.65E-05	0.00055871
TC0100013272.hg.1	HTR1D	11.5	12.6	2.072661	6.71E-05	0.00056307
TC0500013245.hg.1	PCDHB9	4.9	6.7	3.439136	6.78E-05	0.00056797
TSUnmapped0000036	ZNF780B	6.8	8.1	2.421149	6.79E-05	0.00056858
TC0400007572.hg.1	EXOC1	9.8	11.3	2.892873	6.83E-05	0.00057097
TSUnmapped0000026	LRP6	9.5	10.5	2.019306	6.83E-05	0.00057097
TC1900007950.hg.1	ZNF567	6.1	7.3	2.270639	6.83E-05	0.00057097
TC0200013046.hg.1	EXOC6B	9.0	10.2	2.349221	6.86E-05	0.00057232
TC0300012881.hg.1	SLC33A1	7.8	8.8	2.017475	6.88E-05	0.00057435
TC1200012758.hg.1	TAS2R14	7.0	8.5	2.967786	6.92E-05	0.00057627
TC0X00008668.hg.1	FMR1	11.7	12.7	2.056458	6.97E-05	0.0005794
TC1900010375.hg.1	RHPN2	9.2	10.2	2.089030	6.97E-05	0.0005794
TC1300008497.hg.1	UBL3	7.9	9.0	2.235039	6.98E-05	0.00058001
TC0100015645.hg.1	NBPF11	8.4	9.7	2.501740	7.01E-05	0.00058207
TC0800012173.hg.1	EPPK1	6.9	8.3	2.638030	7.10E-05	0.00058846
TC1900011398.hg.1	TMC4	6.0	7.5	2.967628	7.11E-05	0.00058852
TC1800006786.hg.1	RNMT	9.3	10.6	2.435627	7.11E-05	0.00058852
TC2000008746.hg.1	NINL	6.0	7.1	2.138289	7.11E-05	0.00058852
TC1100013129.hg.1	CTSD	12.3	13.3	2.055995	7.15E-05	0.00059104
TC0200016767.hg.1	MREG	4.5	5.6	2.104867	7.15E-05	0.00059104
TC1000010362.hg.1	ZNF248	6.8	8.5	3.323605	7.16E-05	0.00059113
TC0X00008833.hg.1	FAM50A	8.3	9.4	2.230146	7.19E-05	0.0005931
TC0400011643.hg.1	CAMK2D	12.8	13.9	2.148444	7.20E-05	0.00059388
TC0700013102.hg.1	KMT2C	11.1	12.2	2.150599	7.20E-05	0.00059409
TSUnmapped0000020	ZDHHC3	4.7	6.1	2.518968	7.21E-05	0.00059432
TC1500010093.hg.1	ETFA	8.7	9.7	2.091078	7.28E-05	0.00059877
TC1900007456.hg.1	ZNF253	9.7	10.7	2.036095	7.33E-05	0.00060315
TC0700012896.hg.1	CTAGE6	6.1	7.6	2.778613	7.36E-05	0.00060456
TC0800008943.hg.1	PHF20L1	5.7	7.2	2.907391	7.36E-05	0.00060467
TC0700007121.hg.1	AVL9	11.1	12.5	2.481398	7.38E-05	0.00060582
TC0X00008794.hg.1	SLC6A8	13.4	14.5	2.199644	7.41E-05	0.00060748
TC1600006995.hg.1	BFAR	12.4	13.4	2.003610	7.44E-05	0.00060974
TC1000008668.hg.1	SFXN3	10.6	11.7	2.067667	7.47E-05	0.00061209
TC0900010543.hg.1	TLE1	7.5	8.7	2.217967	7.47E-05	0.00061209
TC1200007170.hg.1	CCDC91	8.2	9.6	2.723966	7.51E-05	0.00061399

TC0600007711.hg.1	ITPR3	10.2	11.3	2.152486	7.53E-05	0.0006151
TC0700012797.hg.1	PARP12	6.9	8.4	2.663843	7.54E-05	0.00061521
TC0200007533.hg.1	FOXN2	12.5	13.5	2.099370	7.54E-05	0.00061521
TC1400007647.hg.1	ACOT2	7.1	8.3	2.367211	7.54E-05	0.00061521
TC1200007418.hg.1	ANO6	14.6	15.6	2.056962	7.56E-05	0.00061641
TC1000008891.hg.1	DUSP5	4.3	5.8	2.809073	7.57E-05	0.00061648
TC1900006968.hg.1	C19orf66	5.0	6.3	2.340604	7.57E-05	0.00061651
TC0300006847.hg.1	NR1D2	9.4	10.5	2.171612	7.59E-05	0.00061761
TC0800012310.hg.1	C8orf44	6.5	7.8	2.428903	7.61E-05	0.00061863
TC1700006877.hg.1	ADPRM	5.6	6.6	2.084262	7.62E-05	0.00061884
TC1900011880.hg.1	ZNF44	6.3	7.4	2.202325	7.63E-05	0.00061935
TC0100009064.hg.1	FNBP1L	10.3	11.4	2.192490	7.63E-05	0.00061935
TC0800009529.hg.1	PPP1R3B	7.8	9.3	2.721585	7.67E-05	0.00062199
TC0300009033.hg.1	PLS1	10.3	11.4	2.069460	7.69E-05	0.00062331
TC1100013049.hg.1	BBS1	6.8	7.8	2.125830	7.71E-05	0.0006247
TC1200006604.hg.1	CD9	13.9	14.9	2.017449	7.76E-05	0.0006278
TC1700010686.hg.1	JUP	13.1	14.2	2.149367	7.77E-05	0.00062824
TC0600008972.hg.1	PRDM1	4.1	6.3	4.435303	7.79E-05	0.00062993
TC1700006719.hg.1	RNASEK; C17orf49	13.3	14.5	2.236584	7.80E-05	0.00063071
TC1900011925.hg.1	ZNF91	7.2	8.6	2.570277	7.82E-05	0.00063165
TC0300007115.hg.1	EIF1B	8.9	10.0	2.186895	7.84E-05	0.00063288
TC0600006967.hg.1	EDN1	8.9	10.0	2.209764	7.85E-05	0.00063328
TC0400011299.hg.1	FAM13A	5.1	6.8	3.246977	7.86E-05	0.00063439
TC1700009650.hg.1	SAT2	4.4	5.5	2.276292	7.90E-05	0.00063661
TC0500012238.hg.1	HBEGF	5.9	8.4	5.615007	7.92E-05	0.00063726
TC0200014550.hg.1	ORC4	9.0	10.0	2.017617	8.00E-05	0.00064296
TC0200010330.hg.1	SLC39A10	5.1	6.4	2.327421	8.04E-05	0.00064566
TC0200008803.hg.1	GCC2	10.1	11.9	3.333892	8.08E-05	0.00064913
TC1100012187.hg.1	KBTBD3	4.0	5.3	2.332183	8.13E-05	0.00065186
TC1600009580.hg.1	XYLT1	9.6	11.4	3.475631	8.16E-05	0.00065357
TC1700008984.hg.1	SEC14L1; SCARNA	9.9	11.0	2.167983	8.22E-05	0.00065828
TC0100014794.hg.1	ODF2L	5.5	6.6	2.202364	8.25E-05	0.00066026
TSUnmapped00000801	VPS11	8.8	9.9	2.083671	8.27E-05	0.00066133
TC1200009399.hg.1	PIWIL1	5.4	6.8	2.739393	8.28E-05	0.00066188
TC1600009943.hg.1	SEZ6L2	7.1	8.3	2.299252	8.31E-05	0.00066422
TC0200012098.hg.1	RBKS; BRE-AS1	5.2	6.7	2.842104	8.34E-05	0.0006658
TC1900011883.hg.1	ZNF799	6.7	8.0	2.426161	8.50E-05	0.00067617
TC0200016751.hg.1	LOC100130691; AC	3.6	4.7	2.079083	8.53E-05	0.00067809
TC1600009530.hg.1	KIAA0430; MIR650	8.6	9.9	2.323725	8.56E-05	0.00068008
TC0900007100.hg.1	NPR2	6.0	7.1	2.135077	8.63E-05	0.00068536
TC1200011968.hg.1	HECTD4; MIR6861	10.1	11.3	2.195078	8.65E-05	0.00068606
TC0100018478.hg.1	NBPF10	10.6	11.8	2.328416	8.68E-05	0.00068845
TC0100006861.hg.1	FBXO44	6.0	7.4	2.691153	8.69E-05	0.00068845
TC2000007191.hg.1	DYNLRB1	10.0	11.1	2.077702	8.72E-05	0.0006904
TC0100008271.hg.1	RNF11	6.0	7.3	2.575403	8.74E-05	0.00069188
TC0500011435.hg.1	LYSMD3	9.6	10.7	2.113312	8.75E-05	0.00069188
TC1900011382.hg.1	MYADM	6.0	7.3	2.436481	8.83E-05	0.00069832
TC0200011803.hg.1	NBAS	6.4	7.5	2.084952	8.94E-05	0.00070614
TC1100012474.hg.1	MPZL2	7.1	8.5	2.598048	9.06E-05	0.00071378
TC0100010078.hg.1	IL6R	4.3	5.6	2.379009	9.09E-05	0.00071634

TC0500013204.hg.1	CAST	12.6	13.7	2.082181	9.10E-05	0.00071668
TC1400008333.hg.1	PPP2R5C	13.1	14.3	2.266770	9.11E-05	0.00071668
TC1500010160.hg.1	CTSH	5.8	7.3	2.711396	9.14E-05	0.00071888
TC1600008189.hg.1	PLA2G15	9.0	10.1	2.224714	9.22E-05	0.00072475
TC1100009981.hg.1	TPP1	10.8	11.9	2.160316	9.29E-05	0.00072938
TC1100013055.hg.1	CARNS1	5.5	6.7	2.306127	9.31E-05	0.00073073
TC0X00007310.hg.1	TSPYL2	7.4	8.8	2.704108	9.33E-05	0.00073173
TC0200010164.hg.1	SSFA2	10.7	11.8	2.155311	9.34E-05	0.00073219
TC0200008469.hg.1	FAHD2A	5.0	6.8	3.458826	9.36E-05	0.00073392
TC1200012755.hg.1	PRR4	4.7	6.0	2.508582	9.37E-05	0.00073392
TC0100008803.hg.1	FAM73A	8.9	10.1	2.260736	9.40E-05	0.000736
TC1700012240.hg.1	TBC1D3H; TBC1D3	6.8	8.3	2.817422	9.44E-05	0.00073931
TC0200008823.hg.1	RGPD6; RGPD5	11.6	12.7	2.059043	9.47E-05	0.00074114
TC0100011663.hg.1	C1orf115	4.8	5.9	2.125238	9.50E-05	0.00074258
TC0700013401.hg.1	AC007566.10; GAT	5.2	7.0	3.587383	9.53E-05	0.00074469
TC0300012403.hg.1	TMCC1	6.3	7.3	2.033207	9.61E-05	0.00074971
TC0X00007480.hg.1	HEPH	4.5	5.8	2.412084	9.71E-05	0.00075692
TC0500009521.hg.1	CPEB4	6.8	8.1	2.398997	9.73E-05	0.00075762
TC0900007618.hg.1	PCSK5	10.0	11.1	2.172291	9.80E-05	0.00076232
TC0900012126.hg.1	ANKRD20A3; ANKI	4.7	6.3	2.856878	9.80E-05	0.00076232
TC1000008397.hg.1	IFIT3	6.9	7.9	2.112114	9.81E-05	0.00076232
TC0200016710.hg.1	ANKRD36C	12.1	13.9	3.408083	9.82E-05	0.00076263
TC0300010632.hg.1	OSBPL10	11.4	12.4	2.071756	9.84E-05	0.00076431
TC1400010727.hg.1	RIPK3	3.4	5.0	2.979669	9.96E-05	0.00077289
TC1600011362.hg.1	ACSM3	6.2	7.6	2.646050	9.97E-05	0.00077289
TC1900007622.hg.1	ZNF254	8.0	9.5	2.776123	0.00010004	0.00077486
TC0600009821.hg.1	ULBP1	4.6	6.0	2.539272	0.00010025	0.00077624
TC0500010766.hg.1	PLPP1; RNF138P1	7.0	8.4	2.796244	0.00010047	0.00077762
TC1100011590.hg.1	RAB6A	14.6	15.7	2.115682	0.00010122	0.00078254
TC1700012296.hg.1	PRKAR1A; ARSG	9.4	11.0	2.989017	0.00010125	0.00078254
TC1500007752.hg.1	THSD4	4.8	6.1	2.434028	0.00010334	0.00079732
TC0X00010169.hg.1	BRWD3	8.9	10.4	2.828012	0.0001035	0.00079819
TC0600011876.hg.1	YIPF3	9.8	10.9	2.228927	0.00010376	0.00079942
TC0200007535.hg.1	PPP1R21	7.2	8.5	2.371564	0.00010377	0.00079942
TC1700010967.hg.1	PRR15L	4.0	5.6	2.871901	0.00010409	0.00080134
TC2200007406.hg.1	ATF4	11.3	12.3	2.012655	0.00010458	0.0008048
TC0900006969.hg.1	CHMP5	9.0	10.0	2.012926	0.00010521	0.00080906
TC0600011067.hg.1	TDP2	7.3	8.3	2.065022	0.00010554	0.0008113
TC0200016484.hg.1	RGPD2; RGPD1	7.3	8.8	2.757668	0.00010574	0.00081226
TC0600006918.hg.1	GCNT2	6.3	7.6	2.525483	0.00010591	0.00081333
TC0700009398.hg.1	TAS2R3	6.3	7.9	3.180337	0.000106	0.00081367
TC0100013302.hg.1	GALE	8.2	9.3	2.139159	0.00010627	0.00081551
TC0100012544.hg.1	MMEL1	4.4	5.8	2.625847	0.00010691	0.00081981
TC1000008926.hg.1	ACSL5	14.3	15.4	2.082592	0.00010711	0.00082055
TC0100006812.hg.1	KIF1B	8.3	9.5	2.366988	0.00010733	0.00082185
TC0300010714.hg.1	TRANK1	4.0	5.6	3.017191	0.00010797	0.00082617
TC1800008743.hg.1	CCDC68	8.6	9.8	2.400267	0.0001081	0.00082674
TC1100013206.hg.1	STARD10	5.7	7.1	2.625039	0.00010812	0.00082674
TC0300007466.hg.1	ALAS1	9.7	10.8	2.124698	0.00010855	0.0008294
TC0500011648.hg.1	EFNA5	12.6	13.7	2.194893	0.00010868	0.00083015

TC0500010932.hg.1	SGTB	7.2	8.3	2.184741	0.00010943	0.00083498
TC0700009487.hg.1	TMEM139	9.2	10.3	2.138190	0.00011054	0.00084225
TC1200012181.hg.1	SPPL3	10.3	11.4	2.192528	0.00011079	0.00084387
TC0400008119.hg.1	TIGD2	6.2	7.5	2.313811	0.00011093	0.00084463
TC0500012663.hg.1	SLU7	8.1	9.3	2.268559	0.000111	0.00084483
TC1500009036.hg.1	MEIS2	10.8	11.8	2.008556	0.0001112	0.00084604
TC0600012299.hg.1	SLC17A5	7.0	8.1	2.281159	0.00011131	0.00084631
TC0900007101.hg.1	TMEM8B	4.4	5.8	2.651929	0.00011135	0.00084631
TC1900009576.hg.1	ZNF699	6.3	7.9	3.117054	0.00011235	0.00085325
TC0600011733.hg.1	KCNK5	10.1	11.5	2.592171	0.00011251	0.00085423
TC0300010955.hg.1	ALS2CL	5.1	6.4	2.353453	0.00011309	0.00085827
TC1900007508.hg.1	ZNF430	8.4	9.5	2.027318	0.00011316	0.00085851
TC2000007269.hg.1	TLDC2	8.1	9.4	2.397689	0.00011344	0.00086002
TC1100009237.hg.1	VPS11	7.7	8.8	2.105562	0.00011374	0.00086174
TC1900007096.hg.1	JUNB	5.0	6.3	2.381087	0.0001141	0.00086414
TC0X00006816.hg.1	PDK3	3.3	4.7	2.637964	0.00011432	0.00086547
TC0900012202.hg.1	KIAA2026	7.2	8.4	2.296110	0.00011449	0.00086644
TC1900011305.hg.1	ZNF83	5.7	7.6	3.531932	0.00011462	0.00086714
TC1700012193.hg.1	NDEL1	9.6	10.6	2.007062	0.00011473	0.0008677
TC0200011268.hg.1	RAMP1	9.2	11.2	4.047110	0.00011525	0.00087101
TC0200014991.hg.1	GPR155	4.3	5.4	2.187169	0.00011563	0.00087325
TC0300013970.hg.1	PFKFB4; MIR6823	3.3	6.2	7.670277	0.00011599	0.00087569
TC0200008092.hg.1	DOK1	6.0	7.1	2.134366	0.00011669	0.00088034
TC1200007887.hg.1	MBD6	7.7	8.8	2.113174	0.00011676	0.00088057
TC1200006610.hg.1	PLEKHG6	5.9	7.0	2.152165	0.00011695	0.00088107
TC0600008255.hg.1	EFHC1	6.6	7.7	2.161230	0.00011791	0.00088763
TC0200006969.hg.1	NCOA1	7.0	8.5	2.714477	0.00011849	0.00089167
TC0100013676.hg.1	ZMYM6NB; ZMYM6	8.0	9.2	2.243694	0.00011866	0.00089237
TC0300014020.hg.1	CLDND1	9.6	10.9	2.455924	0.00011874	0.00089267
TC0700010728.hg.1	HERPUD2	12.8	13.8	2.030301	0.0001191	0.00089508
TC1500007695.hg.1	PAQR5	5.7	7.1	2.554633	0.0001196	0.00089847
TC1000012577.hg.1	LIPA	12.4	13.4	2.004158	0.00012013	0.00090216
TC1000006995.hg.1	COMMD3-BMI1; BM	7.4	8.7	2.500063	0.00012066	0.00090523
TC0100011421.hg.1	CD46	6.3	7.6	2.527645	0.00012084	0.00090622
TC2100008568.hg.1	TMPRSS2	3.3	4.5	2.246127	0.00012138	0.00090931
TC1200009967.hg.1	DUSP16	6.4	7.8	2.618394	0.00012266	0.00091791
TC0100013303.hg.1	HMGCL	6.5	7.9	2.707263	0.0001228	0.00091837
TC0200009774.hg.1	TANC1	10.2	11.3	2.131719	0.00012301	0.0009196
TC0400008427.hg.1	AP1AR	8.9	10.3	2.696787	0.00012311	0.00091999
TC0500010869.hg.1	ELOVL7	6.9	8.0	2.110185	0.00012331	0.00092058
TC0400012458.hg.1	HPGD	5.8	7.1	2.409906	0.00012336	0.00092058
TC0800009113.hg.1	PTP4A3	7.1	8.2	2.182832	0.00012357	0.00092185
TC0500012822.hg.1	UBTD2	7.0	8.2	2.229913	0.00012463	0.00092786
TC0100018344.hg.1	LYPLAL1	8.9	10.1	2.289574	0.00012474	0.00092833
TC0800010949.hg.1	SNX16	5.7	7.0	2.413366	0.00012594	0.00093692
TC0X00010895.hg.1	ZNF75D	6.9	8.1	2.271440	0.00012656	0.00094121
TC0200011990.hg.1	PTRHD1	8.8	9.8	2.002097	0.00012661	0.00094128
TC2200008654.hg.1	MFNG	5.0	6.2	2.316729	0.0001287	0.0009555
TC1500009921.hg.1	UACA	13.2	14.3	2.054380	0.00012927	0.00095871
TC1000007103.hg.1	RAB18	10.4	11.5	2.122374	0.00012952	0.00096021

TC0200015601.hg.1	KANSL1L	6.7	7.8	2.201644	0.00013005	0.00096314
TC1500009429.hg.1	DMXL2	7.9	9.0	2.053819	0.00013029	0.00096428
TC0100017118.hg.1	YOD1	7.7	8.9	2.251898	0.00013044	0.00096471
TC1300009993.hg.1	BIVM	7.9	9.0	2.051401	0.00013085	0.00096678
TC1000009140.hg.1	TACC2	7.6	9.0	2.535596	0.00013126	0.00096942
TC0100018484.hg.1	MCL1	14.2	15.3	2.078954	0.00013135	0.00096979
TC0100010798.hg.1	QSOX1	10.4	11.5	2.268219	0.00013162	0.00097144
TC1200011471.hg.1	POC1B; POC1B-G/	7.1	8.4	2.352693	0.00013181	0.00097175
TC0200015314.hg.1	STK17B	11.3	12.4	2.130989	0.000133	0.00097822
TC0X00008509.hg.1	ZNF449	7.2	8.3	2.181451	0.00013336	0.00098021
TC0300006784.hg.1	RAB5A	14.0	15.0	2.017813	0.00013432	0.00098662
TC0100015797.hg.1	POGZ	7.5	8.6	2.145329	0.00013467	0.00098881
TC0900007077.hg.1	UNC13B	7.9	9.0	2.080999	0.00013568	0.00099557
TC0800008371.hg.1	SPAG1	4.3	5.6	2.420468	0.00013706	0.00100398
TC1600009944.hg.1	KCTD13	9.3	10.4	2.107768	0.00013755	0.00100557
TC2100006968.hg.1	IFNGR2	7.4	8.7	2.382610	0.00013756	0.00100557
TC0300011485.hg.1	FOXP1	7.9	9.3	2.542860	0.00014009	0.00102268
TC0Y00006433.hg.1	PLCXD1	4.0	5.2	2.365105	0.00014207	0.00103608
TC0100014930.hg.1	EVI5	5.9	8.0	4.422238	0.0001424	0.00103782
TC0100007206.hg.1	CDA	6.8	7.8	2.092744	0.00014241	0.00103782
TC1100006475.hg.1	RASSF7	8.1	9.4	2.502803	0.00014296	0.00104149
TC0200014772.hg.1	IFIH1	4.0	5.2	2.401525	0.00014401	0.00104843
TC2000010026.hg.1	TMEM189	11.0	12.0	2.051859	0.00014463	0.0010519
TC0300013854.hg.1	NXPE3	4.6	5.8	2.348711	0.00014555	0.00105691
TC0700011665.hg.1	SEMA3A	7.3	8.5	2.329028	0.00014557	0.00105691
TC0500008702.hg.1	CAMLG	8.3	9.8	2.851772	0.00014669	0.00106432
TC1900011690.hg.1	ZNF493	3.6	5.0	2.729060	0.00014886	0.00107824
TC0600014101.hg.1	MICA	8.6	9.9	2.448352	0.00014908	0.0010795
TC0400012854.hg.1	TMEM144	8.0	9.6	3.070687	0.00014938	0.0010812
TC1200011608.hg.1	CDK17	11.2	12.3	2.148237	0.00014942	0.0010812
TC0700009827.hg.1	RBM33	10.3	11.4	2.021826	0.00014951	0.00108154
TC1700012241.hg.1	TBC1D3L; TBC1D3	5.4	7.2	3.305178	0.00015005	0.00108436
TC2200007277.hg.1	MPST	5.4	6.5	2.138372	0.0001511	0.00109154
TC2200009273.hg.1	APOBEC3F	3.7	5.0	2.356498	0.00015179	0.00109569
TC0100006681.hg.1	RNF207	7.4	8.5	2.122018	0.00015212	0.00109746
TC1200011255.hg.1	ZFC3H1	12.4	13.4	2.015600	0.000153	0.00110243
TC1200012584.hg.1	TAPBPL	6.0	7.0	2.086807	0.00015352	0.00110527
TC1300007228.hg.1	WDFY2	6.8	8.5	3.122875	0.00015417	0.00110961
TC1400007328.hg.1	DAAM1	6.4	7.8	2.573919	0.00015643	0.00112221
TC0200006715.hg.1	PQLC3	8.7	10.1	2.706229	0.00015664	0.00112322
TC1900011754.hg.1	ZNF225	4.1	5.2	2.230210	0.00015791	0.00113086
TC1800006679.hg.1	NAPG	9.3	10.4	2.080309	0.00016017	0.00114508
TC0600013180.hg.1	CTAGE9	6.7	8.0	2.508435	0.00016072	0.00114865
TC2000007452.hg.1	HNF4A	11.2	12.3	2.136443	0.00016102	0.00114965
TC0100012272.hg.1	CNST	6.6	7.9	2.526225	0.00016133	0.00115146
TC1000007020.hg.1	OTUD1	5.5	6.8	2.462170	0.00016418	0.00117028
TC0100010518.hg.1	DCAF6	11.6	12.7	2.040423	0.00016578	0.0011805
TC1700011448.hg.1	POLG2	8.7	10.0	2.393881	0.00016596	0.00118135
TC1600010966.hg.1	CDYL2	7.0	8.8	3.343242	0.00016678	0.00118526
TC0100011861.hg.1	OBSCN	4.8	6.3	2.860673	0.00016725	0.00118741

TC0900007457.hg.1	CNTNAP3P2; CNT	6.4	7.8	2.600932	0.00016784	0.00119081
TC0900006655.hg.1	LURAP1L	4.2	5.5	2.457088	0.00017071	0.00120797
TC0X00006433.hg.1	PLCXD1	3.7	4.9	2.281769	0.00017086	0.00120865
TC1500009574.hg.1	ADAM10	12.4	13.5	2.157613	0.00017178	0.00121435
TC1500007513.hg.1	LACTB	6.3	7.8	2.846071	0.00017238	0.00121767
TC1700009949.hg.1	SREBF1	11.5	12.7	2.330060	0.00017242	0.00121767
TC0600011996.hg.1	MUT	7.1	8.3	2.283973	0.00017272	0.00121929
TC1900009431.hg.1	SLC25A23	7.0	9.1	4.386338	0.00017282	0.00121929
TC2000009516.hg.1	BCAS1	4.0	5.8	3.598678	0.00017282	0.00121929
TC1200008675.hg.1	TXNRD1	14.4	15.5	2.126543	0.00017322	0.00122129
TC1700011465.hg.1	LRRC37A3	8.3	9.8	2.699712	0.00017345	0.00122198
TC1500010691.hg.1	NIPA1	11.2	12.2	2.090892	0.00017395	0.00122483
TC1100013057.hg.1	TCIRG1	10.8	11.8	2.065861	0.00017504	0.00123093
TC0700013567.hg.1	LINC00174	9.2	10.3	2.152917	0.00017697	0.00124253
TC1200011591.hg.1	NTN4	7.4	8.6	2.335776	0.00017773	0.00124658
TC1300008688.hg.1	FOXO1	6.9	8.1	2.395528	0.00017797	0.00124745
TC0X00009208.hg.1	CXorf23	4.8	6.1	2.519868	0.00017955	0.00125604
TC0200011449.hg.1	SH3YL1	7.8	9.4	2.893371	0.00017986	0.00125775
TC1900010673.hg.1	DYRK1B	4.4	5.6	2.434220	0.0001807	0.00126284
TC0600009059.hg.1	FOXO3	10.1	11.4	2.502803	0.00018229	0.00127268
TC0100013267.hg.1	LUZP1	10.4	11.5	2.041973	0.00018259	0.00127437
TC0200013261.hg.1	RETSAT	11.3	12.4	2.134354	0.00018365	0.00127946
TC0X00011339.hg.1	ARSE	4.8	6.1	2.400288	0.00018368	0.00127946
TC0600007303.hg.1	BTN3A1	6.7	7.8	2.165617	0.00018479	0.00128471
TC1900006866.hg.1	SNAPC2	6.0	7.0	2.021024	0.00018492	0.00128523
TC1100011234.hg.1	LTBP3	5.2	6.4	2.365166	0.00018521	0.001286
TC0X00009929.hg.1	OPHN1	7.0	8.2	2.310076	0.00018544	0.00128673
TC0300014054.hg.1	PIK3CB	9.5	10.6	2.047578	0.00018713	0.00129766
TC2100007597.hg.1	HSPA13	8.1	9.3	2.253858	0.00018721	0.00129777
TC0300012945.hg.1	RARRES1	4.8	7.3	5.693378	0.00018743	0.00129806
TC1600006733.hg.1	GLIS2	8.5	9.8	2.376299	0.00018765	0.00129911
TC1400009256.hg.1	ATG14	8.6	9.7	2.032103	0.00018789	0.00130038
TC0100018238.hg.1	DNAJB4	6.9	7.9	2.026206	0.00018903	0.00130741
TC1400007029.hg.1	C14orf28	4.6	6.3	3.138913	0.00018919	0.00130777
TC1100006860.hg.1	USP47	9.2	10.2	2.063154	0.00018959	0.00130998
TC1400007628.hg.1	PSEN1	11.3	12.3	2.027822	0.00019248	0.00132704
TC1400006890.hg.1	SRP54	7.9	9.2	2.473315	0.00019433	0.00133806
TC0500010779.hg.1	SLC38A9	6.8	7.9	2.066447	0.00019552	0.00134408
TC1200012788.hg.1	RAPGEF3	5.5	6.7	2.265102	0.00019581	0.00134565
TC1600006593.hg.1	RAB26	6.2	7.7	2.801631	0.00019628	0.00134843
TC1100011103.hg.1	STX5	6.4	8.0	3.077193	0.00019725	0.00135465
TC0300009974.hg.1	MUC20; SDHAP2;	3.1	4.3	2.317386	0.00019734	0.00135483
TC1700011561.hg.1	WIPI1	5.1	6.4	2.516503	0.00019836	0.00136099
TC0600006441.hg.1	DUSP22	6.6	8.2	3.012561	0.0001985	0.00136108
TC0700013493.hg.1	WDR60	6.5	7.8	2.351885	0.00019868	0.00136184
TC0700009400.hg.1	TAS2R5	5.7	7.1	2.693441	0.0001993	0.00136393
TC1400009973.hg.1	RPS6KA5	5.1	6.6	2.864351	0.0002016	0.00137873
TC0500008384.hg.1	AP3S1	11.6	12.7	2.118339	0.00020162	0.00137873
TC0700012584.hg.1	UBE2H	14.0	15.0	2.048338	0.00020208	0.00138064
TC0300007454.hg.1	PARP3	6.0	7.0	2.123502	0.00020213	0.00138064

TC1400007562.hg.1	SRSF5	14.0	15.1	2.098248	0.00020343	0.00138869
TC1000008727.hg.1	NFKB2	7.5	9.0	2.801807	0.00020459	0.0013957
TC0900008684.hg.1	GPR21; RABGAP1	9.5	10.8	2.352003	0.00020479	0.00139659
TC0600013280.hg.1	MAP3K5	8.8	10.0	2.262526	0.00020565	0.001402
TC0400010614.hg.1	CNGA1	5.4	7.0	3.016770	0.00020585	0.00140252
TC0900008891.hg.1	LRRC8A	7.4	8.5	2.090953	0.0002146	0.00145566
TC0900011501.hg.1	NR6A1	8.9	10.0	2.133822	0.00021496	0.00145761
TC0500008431.hg.1	DMXL1	9.4	10.8	2.693619	0.00021581	0.00145967
TC0500009181.hg.1	CNOT8	10.9	12.0	2.205866	0.00021654	0.00146369
TC0500011752.hg.1	FEM1C	7.2	8.8	3.200200	0.00021674	0.00146461
TC0200006757.hg.1	TRIB2	8.8	10.0	2.347376	0.00021896	0.00147822
TC1300009583.hg.1	DOCK9	9.6	10.8	2.350672	0.00021942	0.00148036
TC1000012056.hg.1	ATE1	9.9	10.9	2.012206	0.00022076	0.00148803
TC1100006899.hg.1	ARNTL	6.5	7.9	2.578573	0.00022317	0.00150212
TC0700010216.hg.1	ZNF12	8.3	9.4	2.135722	0.0002232	0.00150212
TC1200006862.hg.1	BCL2L14	5.2	6.2	2.096507	0.00022409	0.00150718
TC0X00008829.hg.1	RPL10; SNORA70	16.5	17.5	2.054091	0.00022526	0.00151328
TC1100011372.hg.1	CHKA	11.6	12.8	2.224462	0.00022807	0.00152771
TC1200007895.hg.1	OS9	12.6	13.7	2.128515	0.00022822	0.0015282
TSUnmapped0000012	KIAA1143	9.7	10.8	2.283497	0.0002283	0.00152828
TC1100011744.hg.1	GAB2	3.1	4.4	2.437595	0.00022897	0.00153219
TC0500009093.hg.1	NDST1	6.7	8.0	2.478665	0.00022916	0.0015326
TSUnmapped0000041	PADI1	3.6	4.8	2.174197	0.00022943	0.00153345
TC1500007901.hg.1	SNX33	6.8	8.0	2.353166	0.0002297	0.00153475
TC0400009543.hg.1	TLR3	3.7	5.2	2.752908	0.00023096	0.00154223
TC2100008506.hg.1	MORC3	8.2	9.3	2.266778	0.00023182	0.00154747
TC0400008053.hg.1	AFF1	13.0	14.1	2.079304	0.00023297	0.00155356
TC0500012312.hg.1	SPRY4	11.7	12.7	2.085589	0.00023347	0.00155607
TC1400008588.hg.1	TEP1	6.8	8.0	2.254452	0.00023357	0.00155629
TC0100011621.hg.1	TGFB2; TGFB2-OT	10.5	11.6	2.127314	0.00023665	0.00157385
TC0900008793.hg.1	ZBTB34	4.8	6.4	2.999311	0.00023783	0.00157971
TC0300013933.hg.1	THUMPD3-AS1	8.1	9.5	2.617204	0.00023958	0.00158861
TC0200016477.hg.1	INO80B-WBP1	8.4	9.4	2.022708	0.0002407	0.00159388
TC0300013703.hg.1	RNF168	9.3	10.3	2.015168	0.00024139	0.00159797
TC0300007050.hg.1	C3orf35	4.3	6.1	3.404605	0.00024235	0.00160384
TC2000007297.hg.1	VSTM2L	5.4	6.9	2.676739	0.00024366	0.001611
TC1400008382.hg.1	TNFAIP2	8.1	9.5	2.584349	0.00024428	0.00161411
TC0300011081.hg.1	UBA7; MIR5193	3.6	5.1	2.726205	0.00024916	0.0016448
TC0400010636.hg.1	OCIAD2	9.1	10.1	2.020152	0.00024961	0.00164725
TC0100015764.hg.1	FAM63A	5.3	6.7	2.615588	0.0002502	0.00165017
TC0100014009.hg.1	TESK2	2.7	4.7	3.804483	0.00025262	0.00166407
TC1300007861.hg.1	PCCA	7.4	8.6	2.280963	0.00025382	0.00167095
TC0100009958.hg.1	CGN	8.0	9.3	2.415546	0.0002547	0.00167517
TC1900009445.hg.1	GPR108; MIR6791	11.4	12.4	2.047591	0.00025581	0.00168201
TC1900011821.hg.1	ZNF776	8.8	9.8	2.070995	0.00025624	0.00168428
TC0500010628.hg.1	ANXA2R	7.0	8.0	2.000695	0.00025734	0.00169099
TC2000007504.hg.1	SNX21	5.6	7.0	2.517919	0.00025809	0.00169544
TC0100007471.hg.1	RPS6KA1	4.6	6.3	3.175589	0.00025884	0.00169829
TC0200010834.hg.1	FAM134A	9.2	10.4	2.332172	0.00026179	0.0017154
TC1100008514.hg.1	TSKU	10.9	12.1	2.210526	0.00026342	0.00172248

TC1700008128.hg.1	NPEPPS	10.6	11.8	2.302265	0.00026512	0.00173154
TC0100013000.hg.1	CASP9	5.1	6.3	2.253683	0.00026835	0.00175081
TC0200016452.hg.1	REL	10.2	11.3	2.215113	0.00026922	0.00175453
TC1000007483.hg.1	FAM21C	12.5	13.6	2.051237	0.00026946	0.00175507
TC1000009296.hg.1	PTPRE	5.2	6.2	2.120049	0.00026998	0.00175692
TC0100007261.hg.1	CELA3B	7.0	8.1	2.139198	0.00026999	0.00175692
TC1000010993.hg.1	MICU1	9.6	10.8	2.213627	0.00027477	0.00178312
TC0400008429.hg.1	ALPK1	7.4	8.6	2.327557	0.00027496	0.00178332
TC1700012369.hg.1	FLCN	6.0	7.3	2.422201	0.00027702	0.0017934
TC1100011966.hg.1	SLC36A4	6.3	7.5	2.296923	0.00027724	0.00179428
TC1600009252.hg.1	PPL	4.9	6.2	2.419747	0.00027914	0.00180438
TC1100011236.hg.1	SSSCA1-AS1	5.7	6.8	2.074652	0.00028098	0.00181337
TC0100010514.hg.1	MPZL1	11.9	12.9	2.012900	0.00028411	0.00183046
TC1700008738.hg.1	PRKAR1A	3.1	4.2	2.118142	0.00028536	0.00183688
TC1000008663.hg.1	SEMA4G	8.4	9.9	2.807657	0.00028669	0.00184412
TC1900011825.hg.1	ZNF587	9.2	10.3	2.045760	0.00028827	0.00185222
TC1800009215.hg.1	ANKRD12	8.7	9.9	2.243879	0.00028987	0.00186087
TC0100013432.hg.1	WASF2	12.2	13.4	2.199130	0.00029033	0.00186323
TC1600007829.hg.1	CYLD	5.8	7.2	2.753918	0.00029116	0.00186801
TC1800006506.hg.1	MYL12A	11.0	12.0	2.018021	0.00029134	0.00186864
TC0300010933.hg.1	FYCO1	7.0	8.1	2.217013	0.00029463	0.00188575
TC0600009753.hg.1	STXBP5	8.4	9.6	2.168526	0.00029576	0.00189076
TC0X00010880.hg.1	MOSPD1	9.5	10.7	2.154896	0.0002981	0.00190342
TC1600010798.hg.1	ZNF821	4.6	5.7	2.121769	0.0002985	0.0019054
TC1700008372.hg.1	SCPEP1	7.8	9.1	2.438700	0.00029858	0.0019054
TC1500010941.hg.1	LYSMD4	4.1	5.2	2.147838	0.00029944	0.00190873
TC1400007354.hg.1	PPM1A	9.3	10.4	2.191206	0.00029946	0.00190873
TC1600006757.hg.1	SMIM22	6.1	7.3	2.378375	0.00030307	0.00192941
TC0700007157.hg.1	NPSR1	10.6	11.6	2.048119	0.00030363	0.00193187
TC0700008318.hg.1	AKAP9	9.0	10.0	2.079011	0.00030726	0.00195089
TC0700006520.hg.1	MAFK	8.1	9.5	2.634974	0.00030771	0.00195258
TC1300008272.hg.1	LATS2	6.8	7.9	2.134174	0.00030855	0.00195733
TC0500013227.hg.1	CDC42SE2	5.8	6.9	2.061399	0.00030873	0.00195794
TC0200014697.hg.1	ACVR1	7.7	8.9	2.262030	0.000311	0.00196997
TC1900011753.hg.1	ZNF284	4.6	5.6	2.015620	0.00031608	0.00199801
TC1500008470.hg.1	ARRDC4	5.7	7.0	2.484543	0.00031959	0.00201781
TC0700011714.hg.1	SRI	12.0	13.1	2.196160	0.00031985	0.00201887
TC0100018480.hg.1	NBPF14	11.5	12.8	2.528680	0.00032206	0.00202883
TC1400009684.hg.1	ALDH6A1	4.9	6.5	2.848694	0.0003292	0.00206873
TC1100009196.hg.1	TMPRSS4	3.6	4.9	2.324783	0.00033066	0.00207736
TC0300006564.hg.1	IL17RE	5.1	6.5	2.647937	0.00033548	0.00210148
TC0900007105.hg.1	HRCT1	6.3	7.8	2.918822	0.00033548	0.00210148
TC1400009395.hg.1	SGPP1	5.5	6.8	2.482604	0.00033595	0.00210378
TC0600009095.hg.1	MICAL1	3.8	5.0	2.275170	0.00033639	0.00210438
TC1900010499.hg.1	PRODH2	4.8	6.0	2.176798	0.00033881	0.0021149
TC0100017022.hg.1	GOLT1A	4.9	6.0	2.241778	0.00034097	0.00212773
TC1000010498.hg.1	8-Mar	4.4	5.6	2.344321	0.00034453	0.00214561
TC1900010943.hg.1	RTN2	6.9	8.2	2.489925	0.00034727	0.00216036
TC1000007603.hg.1	FAM21A	12.5	13.6	2.143984	0.00034941	0.00217282
TC0800012005.hg.1	PTK2	6.0	7.2	2.399000	0.00035298	0.00219267

TC0600009628.hg.1	HECA	7.1	9.2	4.448884	0.00035301	0.00219267
TC0300013696.hg.1	ZDHHC19	3.9	4.9	2.041219	0.00035823	0.0022193
TC1900011914.hg.1	ZNF506	6.0	7.4	2.699861	0.00036161	0.00223834
TC1100006635.hg.1	STIM1; MIR4687	9.6	10.7	2.044068	0.00036281	0.0022438
TC0300009724.hg.1	VPS8	7.8	8.9	2.133034	0.00036461	0.00225232
TC0100018219.hg.1	PPCS; CCDC30	10.4	11.5	2.155097	0.00036696	0.00226624
TC2000009923.hg.1	SYS1	9.4	10.7	2.390172	0.00036716	0.00226678
TC1200010653.hg.1	CERS5	7.7	8.8	2.154999	0.00037163	0.00229109
TC1700011815.hg.1	MXRA7	6.8	8.0	2.315577	0.00037367	0.00230172
TC2000008946.hg.1	GGT7	4.2	5.3	2.120816	0.00037388	0.00230231
TC0900006538.hg.1	JAK2	5.5	6.5	2.044875	0.00037911	0.00232982
TC1900011916.hg.1	ZNF626	6.6	7.6	2.094228	0.00037966	0.00233256
TC0800010081.hg.1	RNF122	4.8	6.0	2.258210	0.00037995	0.00233367
TC0200010253.hg.1	ANKAR	6.7	7.7	2.099782	0.00038012	0.00233393
TC1900007328.hg.1	SLC27A1	7.4	8.6	2.389948	0.00038113	0.00233888
TC0500008086.hg.1	NR2F1	7.1	8.3	2.267593	0.00038481	0.00235948
TC0100006550.hg.1	PRKCZ	9.4	10.8	2.705236	0.0003878	0.00237509
TC0200010854.hg.1	TMEM198	4.8	6.2	2.756573	0.00039537	0.00241664
TC1900008341.hg.1	GIPR	3.2	4.2	2.050100	0.00039751	0.0024269
TC0100018568.hg.1	ARID4B	8.7	9.8	2.224132	0.00040587	0.00246882
TC0400006519.hg.1	CRIPAK	5.2	6.6	2.530199	0.00041177	0.00250045
TC0100007207.hg.1	PINK1; MIR6084	6.5	8.0	2.727404	0.00041528	0.00252035
TC1700012440.hg.1	RNFT1	7.5	8.5	2.036406	0.00041849	0.00253842
TC0700013435.hg.1	CDHR3	6.2	7.6	2.478676	0.00042906	0.00259589
TC0100010369.hg.1	ATF6	10.2	11.3	2.159575	0.00043162	0.00260625
TC1100012810.hg.1	NFRKB	5.1	6.4	2.414857	0.00043832	0.00264148
TSUnmapped0000001	LRP6	5.7	6.9	2.307239	0.00043873	0.00264323
TC0100013713.hg.1	STK40	8.6	9.9	2.427419	0.00044938	0.00269668
TC0500013336.hg.1	SSBP2	7.8	8.9	2.023119	0.00045126	0.00270589
TC0700013442.hg.1	LSMEM1	4.6	6.0	2.599657	0.00045128	0.00270589
TC0700013343.hg.1	ZNRF2	9.2	10.2	2.042512	0.00045259	0.00271304
TC1600006658.hg.1	IL32	5.9	7.5	3.024086	0.00045858	0.00274509
TC1600011487.hg.1	NPIPA8	9.9	11.1	2.253126	0.00046687	0.00278855
TC1300009740.hg.1	LIG4	5.7	7.0	2.549518	0.00046746	0.00279042
TC0200013106.hg.1	CCDC142; MRPL5	7.1	8.1	2.084146	0.00046789	0.00279223
TC0300007044.hg.1	GOLGA4	10.2	11.3	2.178061	0.00046947	0.00279935
TC0600014257.hg.1	HLA-C	5.0	6.5	2.827599	0.00047052	0.00280403
TC0900007085.hg.1	TESK1; MIR4667	12.0	13.2	2.220915	0.00047168	0.00280859
TC1700007779.hg.1	MSL1	9.0	10.2	2.248923	0.00047512	0.00282676
TC1000012512.hg.1	PAOX	5.4	6.4	2.059105	0.00047609	0.00283092
TSUnmapped0000028	LRP6	6.3	7.5	2.189828	0.00047847	0.00284132
TC1700008912.hg.1	MYO15B	5.2	6.5	2.362532	0.00048346	0.00286918
TC0600008722.hg.1	ZNF292	7.9	9.1	2.410607	0.00048546	0.00287947
TC0600008114.hg.1	C6orf223	3.7	4.9	2.296488	0.00049088	0.00290598
TC0600011525.hg.1	RXRB	8.5	9.7	2.283629	0.00049259	0.00291528
TC1500009946.hg.1	MYO9A	8.7	9.8	2.152401	0.00049275	0.00291546
TC0200011382.hg.1	SNED1	4.1	5.5	2.524915	0.00050036	0.00295315
TC0900012140.hg.1	PGM5	2.9	3.9	2.018014	0.00050318	0.00296651
TC0200016719.hg.1	SNORD89; RNF14	7.2	8.2	2.049052	0.00050695	0.00298303
TC0400010886.hg.1	UBA6	9.1	10.1	2.005061	0.00050916	0.00299272

TC1900012028.hg.1	ZNF320	9.6	10.7	2.018476	0.0005098	0.00299485
TC0300010217.hg.1	CIDEC	6.2	7.4	2.383704	0.00051648	0.00302995
TC1900008691.hg.1	ZNF766; MIR643	7.9	9.0	2.046624	0.0005168	0.00303011
TC1700007630.hg.1	TBC1D3G	5.1	6.4	2.365130	0.00052089	0.00304992
TC0600007518.hg.1	HLA-L	8.3	9.5	2.296699	0.00052102	0.00304992
TC0500011655.hg.1	FBXL17	9.0	10.0	2.023802	0.00052236	0.00305615
TC0100007532.hg.1	STX12	7.3	8.4	2.110594	0.00052445	0.00306581
TC0100006884.hg.1	VPS13D	6.1	7.2	2.117589	0.0005249	0.00306756
TC1500010915.hg.1	GOLGA6L5P; GOL	5.6	6.8	2.353264	0.00053472	0.0031072
TC1500006666.hg.1	GOLGA8F; GOLGA	7.3	8.4	2.176776	0.00053866	0.00312669
TC0800011597.hg.1	EXT1; spawla	6.2	7.5	2.436907	0.00054487	0.00315591
TC0600013275.hg.1	MAP7	7.7	8.8	2.157932	0.00055258	0.00319368
TC0X00007382.hg.1	RRAGB	5.1	6.7	2.974007	0.00055622	0.00320954
TC0X00007557.hg.1	MED12	10.5	11.5	2.085514	0.00056967	0.00327921
TC0X00006477.hg.1	CD99	9.2	10.5	2.339955	0.00057337	0.00329605
TC0900006597.hg.1	KDM4C	7.3	8.3	2.033469	0.00057424	0.00330018
TC1100012994.hg.1	CRY2	6.0	7.1	2.125108	0.0005802	0.00332907
TC1000009869.hg.1	CDNF	4.5	5.8	2.489255	0.00058379	0.00334609
TC0600006554.hg.1	FOXC1	6.0	7.0	2.019454	0.00058554	0.00335342
TC1200012190.hg.1	OASL	9.0	10.2	2.313891	0.00058651	0.00335809
TC1000008380.hg.1	LIPK	3.6	4.7	2.165767	0.00058719	0.0033602
TC1100009833.hg.1	OSBPL5	4.2	5.3	2.111855	0.00058893	0.00336655
TC0500013166.hg.1	ZNF131	7.2	8.2	2.005292	0.00059222	0.00338179
TC0400009132.hg.1	ETFDH	7.3	8.5	2.302613	0.00059486	0.00339415
TC0400007128.hg.1	TBC1D19	6.7	7.9	2.366033	0.00059919	0.00341612
TC1900012023.hg.1	ZNF836	5.1	6.2	2.185925	0.00060152	0.00342666
TC0600009436.hg.1	L3MBTL3	7.5	8.7	2.309255	0.00060474	0.0034386
TC1200010686.hg.1	SLC11A2	9.4	10.4	2.037611	0.00060537	0.00344075
TC0600008678.hg.1	MRAP2	6.5	7.6	2.076362	0.0006056	0.00344075
TC0200011040.hg.1	ITM2C	11.7	12.7	2.089842	0.00060831	0.0034534
TC1900011106.hg.1	NUCB1	6.1	7.2	2.058573	0.00062387	0.00353145
TC1100008139.hg.1	AIP	7.7	8.8	2.149366	0.00063163	0.00356789
TC0300008017.hg.1	ZNF654	7.2	8.4	2.285098	0.00063195	0.00356875
TC1900011989.hg.1	FAM83E	4.0	5.1	2.155231	0.00063322	0.00357309
TC1900008257.hg.1	ZNF227	6.5	8.2	3.099359	0.00063815	0.00359241
TC0200007363.hg.1	MTA3	8.8	9.9	2.193387	0.00064508	0.00362541
TC1700011313.hg.1	USP32	7.7	8.9	2.371898	0.00064631	0.00362787
TC0900007082.hg.1	RUSC2	8.3	9.4	2.181956	0.00066209	0.00369514
TC0700008579.hg.1	TRIM56	8.7	9.8	2.199301	0.00066904	0.00372812
TC1100008669.hg.1	PRSS23	4.8	6.7	3.572219	0.00066953	0.00372891
TC0300010720.hg.1	EPM2AIP1	5.9	7.3	2.509377	0.00067026	0.00373203
TC0500006803.hg.1	TRIO	8.7	9.7	2.064185	0.00067254	0.00374081
TC0500012588.hg.1	HAVCR1	5.5	6.8	2.412049	0.00068743	0.00381472
TC0800012313.hg.1	SGK3; C8orf44-SG	7.6	8.6	2.006349	0.00069613	0.00385605
TC1900011751.hg.1	ZNF222	7.1	8.1	2.121713	0.00069646	0.00385687
TC0900012278.hg.1	ST6GALNAC6	7.3	8.5	2.187368	0.00070084	0.00387315
TC0400012921.hg.1	TLR1	4.7	6.4	3.141021	0.00070557	0.00389423
TC1000009221.hg.1	ZRANB1	9.6	10.8	2.190959	0.00070654	0.00389858
TC1300009273.hg.1	COMMD6	8.4	9.5	2.151824	0.00070973	0.00391017
TC0900008847.hg.1	LCN2	7.6	8.6	2.013426	0.00071413	0.00393036

TC1100007472.hg.1	NR1H3	8.1	9.4	2.447466	0.00071598	0.00393853
TC1200011245.hg.1	PTPRB	4.7	5.9	2.353304	0.00072808	0.0039946
TC1700007387.hg.1	TRAF4	6.4	7.5	2.150018	0.00073004	0.00400153
TC0Y00006476.hg.1	CD99	8.9	10.2	2.373256	0.00073917	0.00404639
TC1100008541.hg.1	AAMDC	5.2	6.4	2.191734	0.00074933	0.00409571
TC0200011670.hg.1	KIDINS220	5.2	6.2	2.070549	0.00074962	0.00409626
TC0900009894.hg.1	KIAA1161	10.2	11.3	2.131751	0.00075047	0.00409778
TC0900008312.hg.1	SLC44A1	10.1	11.4	2.405910	0.00075735	0.00413324
TC1500008175.hg.1	PDE8A	6.7	7.7	2.022271	0.00076778	0.00418057
TC0100018509.hg.1	GLMP	5.4	6.7	2.400743	0.0007691	0.0041867
TC1700012395.hg.1	TBC1D3L	7.7	9.3	3.162982	0.00077034	0.00419134
TC2200009345.hg.1	C1QTNF6	5.3	6.4	2.133990	0.00077087	0.00419317
TC2000007817.hg.1	FAM210B	7.1	8.7	3.033206	0.00077305	0.00420395
TC0300009855.hg.1	IL1RAP	5.9	7.3	2.626348	0.00077426	0.00420946
TC1300006979.hg.1	RGCC	4.3	5.5	2.313493	0.00078055	0.0042376
TC1300007549.hg.1	CLN5	6.6	8.0	2.616508	0.00078082	0.0042376
TC0200011975.hg.1	TP53I3	6.8	7.9	2.123944	0.00078503	0.00425724
TC1700009850.hg.1	PMP22	4.7	6.7	4.025000	0.0008017	0.0043345
TC1700012396.hg.1	TBC1D3C	6.1	7.6	2.769936	0.00081804	0.00440838
TC1400008908.hg.1	EAPP	7.0	8.2	2.392497	0.00081867	0.00441067
TC0200012075.hg.1	ZNF513	7.0	8.4	2.645131	0.00083041	0.00446457
TC0600008632.hg.1	BCKDHB	10.7	11.7	2.055320	0.00084062	0.00450965
TC0100009149.hg.1	PTBP2	7.3	8.6	2.474921	0.000867	0.00463151
TC1100013062.hg.1	NADSYN1	4.7	5.9	2.291452	0.00086838	0.00463771
TC0900007472.hg.1	ANKRD20A1	4.7	5.7	2.027499	0.00087104	0.00465075
TC0700013603.hg.1	RASA4	4.6	5.9	2.543324	0.0008762	0.00467131
TSUnmapped0000010	DUSP16	5.2	6.6	2.612227	0.00088008	0.00468621
TC1700010515.hg.1	TBC1D3	6.6	8.7	4.092414	0.00088174	0.00469384
TC1900006605.hg.1	ZNF57	7.9	8.9	2.009918	0.00088912	0.00472796
TC1500008232.hg.1	ISG20	6.4	7.8	2.759050	0.00088925	0.00472796
TC0100017597.hg.1	C1orf145	4.4	5.7	2.465749	0.00089215	0.00473952
TC2000007497.hg.1	WFDC13	3.6	5.2	2.889320	0.00089367	0.00474322
TC0300012654.hg.1	TFDP2	9.9	11.2	2.407996	0.00089897	0.00476666
TC1400010716.hg.1	HOMEZ	8.4	9.5	2.098690	0.00090272	0.00478534
TC1000011659.hg.1	MGEA5	8.9	10.1	2.315826	0.00090688	0.00480504
TC0100009449.hg.1	MOV10	5.8	7.0	2.380073	0.00092092	0.00487192
TC1900007342.hg.1	FCHO1	4.6	5.8	2.252061	0.00092132	0.00487192
TC1900008745.hg.1	ZNF525	6.6	7.6	2.008454	0.00092358	0.00488028
TC0300007472.hg.1	GLYCTK	7.0	8.0	2.085712	0.00092837	0.00490196
TC0500012017.hg.1	IRF1	5.5	6.8	2.321598	0.00094882	0.00499026
TC1700011768.hg.1	GALK1	7.4	8.6	2.379188	0.00099649	0.00519973
TC0100013315.hg.1	IFNLR1	6.7	8.0	2.467533	0.00099665	0.00519973
TC0700009231.hg.1	AKR1B15	5.7	7.3	2.992790	0.00100546	0.00523804
TC1100009917.hg.1	HBB	3.0	4.1	2.113315	0.00101476	0.00527657
TC1900007819.hg.1	ZNF302	7.6	8.7	2.064013	0.00101878	0.0052882
TC1000007421.hg.1	ZNF487	4.7	6.2	2.784507	0.00102112	0.00529647
TC0100009468.hg.1	LRIG2	7.4	8.5	2.111152	0.00103669	0.00536689
TC1900010952.hg.1	FBXO46	5.7	6.9	2.265095	0.00105575	0.00544319
TC1200008165.hg.1	KCNMB4	4.6	5.7	2.159320	0.00105726	0.00544836
TC0X00009942.hg.1	PJA1	6.9	7.9	2.030267	0.0010758	0.00553326

TC0700013436.hg.1	CDHR3	3.9	4.9	2.011942	0.00110382	0.00565028
TC0X00006865.hg.1	IL1RAPL1	3.8	5.5	3.130283	0.00112864	0.0057677
TC0700013291.hg.1	ESYT2	11.5	12.6	2.200729	0.00113698	0.00580203
TC0100009109.hg.1	SLC44A3	6.8	7.8	2.048246	0.00114357	0.00583287
TC0600014258.hg.1	HLA-B	7.1	8.1	2.043908	0.00115037	0.00585919
TC0X00007744.hg.1	SH3BGRL	7.5	8.6	2.087032	0.00117039	0.00594423
TC1000012430.hg.1	RP11-295P9.3; PR	3.2	4.8	2.942061	0.00119428	0.00604698
TC1200009928.hg.1	TAS2R19	4.8	5.9	2.100782	0.00120673	0.00610569
TC0300013334.hg.1	MCCC1	7.7	8.7	2.082938	0.00121394	0.00613492
TC1400007899.hg.1	SPATA7	5.4	6.5	2.098871	0.00124365	0.00625998
TC0700008626.hg.1	ORAI2	8.2	9.3	2.077939	0.00125147	0.00629345
TC0500009669.hg.1	ZNF354B	7.3	8.4	2.119650	0.00126468	0.00634351
TC0100012760.hg.1	RERE	7.9	9.0	2.212863	0.00127595	0.0063848
TC0400007276.hg.1	KLHL5	6.3	7.5	2.324158	0.00129792	0.00647541
TC1900009586.hg.1	ZNF426	7.1	8.2	2.225028	0.00132708	0.00660397
TSUnmapped0000006	MLXIP	4.6	5.6	2.026448	0.00133623	0.00664356
TC1200009921.hg.1	TAS2R13	4.7	5.7	2.096480	0.00135131	0.00670434
TC0100011554.hg.1	RPS6KC1	6.5	7.9	2.504189	0.00135469	0.00671645
TC0100016122.hg.1	IGSF8	4.2	5.3	2.205096	0.00135859	0.00673421
TC0700011905.hg.1	BAIAP2L1	16.0	17.0	2.009294	0.00136998	0.00678285
TC1000010982.hg.1	ASCC1	6.2	7.3	2.096215	0.00137654	0.00681219
TC1400010621.hg.1	CHURC1	9.9	11.0	2.078879	0.00137793	0.00681592
TC2100006980.hg.1	ITSN1	3.5	4.7	2.272884	0.00138162	0.00682787
TSUnmapped0000017	CACFD1	4.0	5.7	3.383428	0.00138868	0.00685489
TC2200009357.hg.1	PACSIN2	10.8	11.9	2.057921	0.00141926	0.00697373
TC1900011066.hg.1	CCDC114	4.6	6.4	3.290110	0.00146246	0.00714345
TC0800012245.hg.1	ZNF250	6.8	7.8	2.041544	0.00148331	0.00723124
TC0600012061.hg.1	ICK	7.7	8.8	2.208510	0.00151899	0.00737087
TC1000012130.hg.1	FAM53B	5.8	7.7	3.641037	0.00153236	0.0074257
TSUnmapped0000026	BCL2L14	4.9	6.1	2.354316	0.0015566	0.00751461
TC1100012445.hg.1	BACE1	5.3	6.5	2.337989	0.00161575	0.00775964
TC0X00008394.hg.1	BCORL1	8.3	9.3	2.118849	0.00163824	0.00783783
TC0100017836.hg.1	LYST	8.8	9.8	2.004892	0.00167731	0.00800332
TC1600006539.hg.1	GNPTG	8.5	9.6	2.110075	0.00168154	0.00801992
TC0200014738.hg.1	RBMS1	5.3	6.5	2.262371	0.0016826	0.00802321
TC0600013193.hg.1	STX7	7.6	8.6	2.076236	0.00171754	0.00816442
TC0400011706.hg.1	FABP2	3.2	4.2	2.095337	0.00176179	0.00834149
TC1000008364.hg.1	RP11-380G5.2; PT	8.0	9.2	2.299495	0.001766	0.00835772
TC0200014848.hg.1	ABCB11	2.7	3.8	2.079377	0.00176913	0.00836885
TC0100015873.hg.1	S100A13	7.1	8.4	2.384837	0.00180117	0.00849604
TC1600006637.hg.1	ZG16B	6.4	7.8	2.615457	0.00181155	0.00854125
TC2000006864.hg.1	RIN2	5.4	6.5	2.122638	0.00182127	0.00858139
TC1700006645.hg.1	PLD2	8.0	9.2	2.289638	0.00187595	0.00881003
TC0100010121.hg.1	HCN3	5.6	7.2	2.958931	0.0018986	0.00889438
TC1900010802.hg.1	LIPE	3.4	5.6	4.468593	0.00195246	0.00910424
TC0100009101.hg.1	ABCD3	5.0	6.1	2.151795	0.00196097	0.00913726
TC0300011853.hg.1	SENP7	5.3	7.1	3.462852	0.00197343	0.00917841
TC0200007194.hg.1	BIRC6	6.5	7.5	2.013793	0.00199381	0.00925813
TC1700009992.hg.1	FAM83G	6.6	7.9	2.420979	0.00202866	0.00938797
TC1200010908.hg.1	STAT2	6.3	7.4	2.054603	0.00203362	0.00940277

TC0100008697.hg.1	CTH	4.5	5.6	2.156480	0.00204456	0.00944674
TC0400006491.hg.1	IDUA	6.1	7.4	2.493597	0.00206783	0.00953578
TC0200015226.hg.1	HIBCH	7.5	9.1	2.995861	0.00207368	0.00954978
TC1500007977.hg.1	HYKK	5.5	6.5	2.049776	0.00211792	0.00970416
TC1100006509.hg.1	MUC5B	6.8	8.1	2.400808	0.002168	0.00990189
TC1500008046.hg.1	CEMIP	6.0	7.0	2.018385	0.0021787	0.00993596
TC1700012143.hg.1	RAB40B	5.9	7.3	2.718997	0.00219572	0.00999656
TC1000011025.hg.1	PPP3CB	7.6	8.6	2.006283	0.00219978	0.01001291
TC1000011520.hg.1	OPALIN	4.0	5.3	2.446635	0.00222698	0.01011312
TC0100017793.hg.1	IRF2BP2	9.7	10.7	2.025770	0.00224904	0.01018741
TC1600008199.hg.1	ZFP90	8.2	9.3	2.137524	0.00227539	0.01028504
TC0500013130.hg.1	TRIM52	7.2	8.2	2.014690	0.00238818	0.01074787
TC0600008043.hg.1	HCRP1	4.5	5.5	2.052918	0.00251951	0.01122994
TC0800008279.hg.1	PLEKHF2	7.5	8.8	2.370288	0.00259299	0.01150008
TC0400010715.hg.1	CHIC2	5.3	6.5	2.306076	0.00259874	0.0115232
TC0400011978.hg.1	TBC1D9	6.1	7.2	2.166442	0.00270439	0.0119338
TC0300013962.hg.1	ANO10	8.2	9.2	2.016939	0.00272075	0.01199725
TC1900011819.hg.1	ZNF211	4.8	6.1	2.338186	0.00278505	0.0122241
TC0400007495.hg.1	DCUN1D4	9.2	10.3	2.163469	0.00278803	0.01223107
TSUnmapped0000058	BCL2L14	4.9	6.0	2.209767	0.00280113	0.01228349
TC0100014201.hg.1	CC2D1B	4.6	5.9	2.349511	0.00283988	0.01243055
TC0900009779.hg.1	C9orf72	3.6	4.7	2.064245	0.00284671	0.01245539
TC1400007500.hg.1	ARG2	3.7	4.8	2.144533	0.00286298	0.0125189
TC1600008496.hg.1	NUDT7	5.4	6.8	2.704940	0.00290541	0.01267461
TC1900011814.hg.1	ZNF17	7.1	8.4	2.555988	0.00293946	0.01278555
TSUnmapped0000031	ZNF780A	6.3	7.5	2.238197	0.0029915	0.01297245
TC0700010257.hg.1	ICA1	2.9	3.9	2.048267	0.00302071	0.01308324
TC1100007482.hg.1	SLC39A13	6.8	8.0	2.284054	0.00302668	0.01310313
TC0100015796.hg.1	POGZ	8.0	9.1	2.141484	0.00312236	0.01344205
TC1700010221.hg.1	DHRS13	5.5	6.5	2.090640	0.00316698	0.01359595
TC2000007670.hg.1	SNAI1	4.2	5.2	2.040783	0.00319708	0.01370595
TC1700009436.hg.1	MNT	6.7	8.1	2.593251	0.00321878	0.01377971
TC1900006904.hg.1	MYO1F	5.2	6.7	2.787386	0.00323372	0.01382986
TC2000009886.hg.1	PANK2	4.3	5.7	2.590253	0.00323863	0.01384811
TC2000008650.hg.1	THBD	6.8	8.2	2.608191	0.00328039	0.01399318
TC1600009486.hg.1	PLA2G10	3.8	5.1	2.450831	0.00335477	0.01428777
TC1700010456.hg.1	CCL23	4.0	5.3	2.493875	0.00336032	0.01430855
TC1700010982.hg.1	SKAP1	5.2	6.3	2.152184	0.00340217	0.01446091
TC1000009665.hg.1	TUBAL3	5.4	6.9	2.880115	0.00342421	0.01453564
TSUnmapped0000022	ZNF780A	6.3	7.4	2.138484	0.00345641	0.01465651
TC1600009165.hg.1	MMP25-AS1	4.0	5.0	2.131478	0.00360662	0.01519145
TC0500006664.hg.1	ADCY2	6.1	7.1	2.111574	0.00362224	0.01523927
TC1900011884.hg.1	ZNF443	6.8	8.3	2.919960	0.00362914	0.01525631
TC1000009276.hg.1	DOCK1	9.1	10.2	2.131887	0.00373039	0.01561158
TC0800012306.hg.1	MTFR1	6.4	7.6	2.270731	0.0037428	0.01565607
TC0400009684.hg.1	ZNF721	7.3	8.5	2.230690	0.00381807	0.0159319
TC0100010775.hg.1	SOAT1	7.2	8.3	2.116534	0.00382873	0.01597016
TC1900007419.hg.1	SLC25A42	4.3	5.3	2.039987	0.00399321	0.01652089
TC0100016539.hg.1	ANGPTL1	3.3	4.5	2.418296	0.00399388	0.01652089
TC0100007694.hg.1	HPCA	5.8	7.1	2.542082	0.00413302	0.01700788

TC0100017420.hg.1	DUSP10	3.1	4.3	2.286176	0.0042008	0.01724048
TC0500010780.hg.1	SLC38A9	5.2	6.4	2.313570	0.00421785	0.0173039
TC1500009127.hg.1	PPP1R14D	3.9	4.9	2.018722	0.0042984	0.01757379
TC0100007162.hg.1	PQLC2	7.6	9.1	2.740379	0.00446933	0.01816526
TC0800006892.hg.1	NAT2	3.4	4.5	2.066216	0.00455099	0.01844822
TC0300012377.hg.1	RAB43	4.6	5.7	2.196339	0.00463955	0.01874347
TC0200016660.hg.1	C2orf61	4.6	5.8	2.299411	0.00505213	0.02008286
TC1500010049.hg.1	FAM219B	4.5	5.7	2.305362	0.00507214	0.02014951
TC0300012038.hg.1	CCDC191	5.2	6.4	2.275729	0.00517354	0.02045763
TC2000009815.hg.1	PTK6	7.8	8.8	2.058468	0.00536721	0.02108738
TC0100009035.hg.1	KIAA1107	3.5	4.7	2.305271	0.0054303	0.02130402
TC1900011077.hg.1	LMTK3	4.5	5.6	2.087341	0.00554707	0.02165931
TC1700010827.hg.1	GRN	7.1	8.4	2.400132	0.00574653	0.02236145
TC0900008222.hg.1	STX17	6.5	7.6	2.107755	0.00577349	0.02245326
TC0200010473.hg.1	FZD7	6.8	7.9	2.197353	0.00580227	0.02254476
TC0100013076.hg.1	PADI2	5.2	6.2	2.069498	0.00580476	0.02255034
TC2200007614.hg.1	FAM118A	7.7	8.8	2.048216	0.00597808	0.02310648
TC0600008022.hg.1	TOMM6; PRICKLE[illegible]	5.3	6.3	2.054136	0.00598728	0.02312953
TC1100007221.hg.1	TCP11L1	7.2	8.8	3.095307	0.00625495	0.0240036
TC0100018399.hg.1	PLEKHG5	7.8	8.8	2.030717	0.00631988	0.02423543
TC0400006930.hg.1	CPEB2	6.1	7.6	2.834827	0.0063383	0.02429737
TC0X00007205.hg.1	HDAC6	7.9	9.1	2.249005	0.00646707	0.02469392
TC0100010010.hg.1	LCE1A	5.1	6.3	2.222826	0.00651209	0.02485256
TC0500013025.hg.1	ZNF354A	6.5	7.6	2.155048	0.00665649	0.02530456
TC0300013125.hg.1	PLD1	4.9	6.0	2.171068	0.00672006	0.02550103
TC1100012473.hg.1	MPZL3	6.1	7.5	2.643756	0.00690231	0.02609014
TC1800009218.hg.1	ANKRD20A5P; RH[illegible]	4.8	5.9	2.160993	0.00729876	0.02740134
TC1300010011.hg.1	LINC00452	7.3	8.4	2.056338	0.00809925	0.02987834
TC0200015424.hg.1	ALS2CR12	4.7	5.8	2.192563	0.00817577	0.0301347
TC1500010946.hg.1	TM2D3	3.3	4.6	2.396543	0.00855703	0.03132999
TC0300010931.hg.1	LZTFL1	7.2	8.3	2.112370	0.0086281	0.03153101
TC1700010676.hg.1	KRT15; MIR6510	6.2	7.5	2.473835	0.0089362	0.03241393
TC1100011294.hg.1	CTSF	4.1	5.2	2.197975	0.00926326	0.0334138
TC0500009027.hg.1	SCGB3A2	4.0	5.4	2.551877	0.00928325	0.03346337

MIA PaCa-2 cell line

ID	Gene Symbol	Ns Avg (log2)	N-dep Avg (log2)	Fold Change	P-val	FDR P-val
TC0100015598.hg.1	TXNIP	7.9	17.1	592.7853	1.08E-09	2.31E-05
TC0500010635.hg.1	HMGCS1	10.0	17.3	150.2119	5.37E-09	5.02E-05
TC2000007202.hg.1	ACSS2	8.3	15.8	187.364	9.36E-09	5.02E-05
TC0500012567.hg.1	FAXDC2	4.8	10.6	53.98195	3.00E-08	8.28E-05
TC1200009800.hg.1	SLC2A3	10.5	15.8	38.42677	4.04E-08	8.28E-05
TC1600011221.hg.1	MVD	8.0	13.0	31.41637	5.42E-08	8.28E-05
TC1200009796.hg.1	SLC2A14	12.2	17.4	34.91875	5.69E-08	8.28E-05
TC0300013471.hg.1	BCL6	7.7	12.7	30.41971	5.86E-08	8.28E-05
TC1000009536.hg.1	IDI1	9.3	14.2	29.54024	6.10E-08	8.28E-05
TC0100008697.hg.1	CTH	5.9	10.8	30.27584	6.18E-08	8.28E-05
TC0400009221.hg.1	MSMO1	13.8	18.9	33.53207	6.69E-08	8.44E-05
TC1200006787.hg.1	GABARAPL1	6.0	11.7	51.34689	9.20E-08	0.00010958
TC0300009673.hg.1	KLHL24	7.1	12.2	33.37902	1.48E-07	0.00012438
TC1100010893.hg.1	SLC43A1	7.5	12.7	35.91764	1.53E-07	0.00012438
TC0X00006799.hg.1	SAT1	8.9	13.3	20.78678	1.66E-07	0.00012438
TC1200008726.hg.1	TCP11L2	4.2	9.0	26.2906	2.25E-07	0.00013368
TC1700010200.hg.1	ALDOC	7.2	11.8	24.09956	2.29E-07	0.00013368
TC1600007982.hg.1	HERPUD1	9.6	13.7	16.93629	2.46E-07	0.0001383
TC0X00010473.hg.1	TSC22D3	4.9	10.9	64.45052	2.89E-07	0.00014761
TC0800006760.hg.1	FDFT1	12.8	16.7	14.92631	3.39E-07	0.00016518
TC0600008757.hg.1	PNRC1	7.8	11.7	14.72438	3.99E-07	0.00017514
TC1600009952.hg.1	YPEL3	5.1	10.2	34.14375	4.07E-07	0.00017514
TC0700011876.hg.1	ASNS	10.4	14.3	15.0753	4.14E-07	0.00017514
TC0700008747.hg.1	HBP1	9.5	13.5	15.80156	4.18E-07	0.00017514
TC0700012798.hg.1	KDM7A	6.4	10.4	15.92681	6.48E-07	0.00021374
TC0700009807.hg.1	INSIG1	9.9	13.4	11.53052	7.04E-07	0.00021974
TC1700008920.hg.1	ITGB4	10.2	13.7	11.63618	7.60E-07	0.00022343
TC2100007451.hg.1	COL6A2	6.5	10.2	12.81175	8.01E-07	0.0002303
TC1900008057.hg.1	ZFP36	6.4	10.9	22.54014	8.16E-07	0.0002303
TC1700011561.hg.1	WIPI1	5.6	9.6	16.45079	8.38E-07	0.0002305
TC1800007298.hg.1	LIPG	7.4	11.7	19.95371	9.29E-07	0.00023969
TC0500009488.hg.1	CREBRF	5.4	10.2	27.38255	9.39E-07	0.00023969
TC0900008902.hg.1	PPP2R4	11.7	15.1	10.54977	1.00E-06	0.00024493
TC0600009821.hg.1	ULBP1	6.4	10.5	16.36941	1.10E-06	0.00025709
TC1200011770.hg.1	ALDH1L2	8.2	12.4	18.95202	1.17E-06	0.00026745
TC1600008005.hg.1	ADGRG1	5.6	10.1	21.88666	1.20E-06	0.00026995
TC0500007881.hg.1	JMY	7.1	10.7	11.59519	1.25E-06	0.00027314
TC0300014095.hg.1	TM4SF19	3.6	7.3	12.66864	1.27E-06	0.00027314
TC0300014049.hg.1	ACAD11	8.1	11.6	11.28907	1.34E-06	0.00027314
TC1100013012.hg.1	STX3	7.2	10.6	10.93791	1.41E-06	0.00028286
TC0600007862.hg.1	PIM1	8.0	11.5	11.37276	1.48E-06	0.00028899
TC1400008118.hg.1	AK7	6.8	10.0	8.994451	1.60E-06	0.0003004
TC0900008953.hg.1	ASS1	7.8	11.7	15.19142	1.67E-06	0.0003004
TC0600012883.hg.1	TUBE1	10.1	13.2	8.623298	1.69E-06	0.0003004
TC0800007080.hg.1	BNIP3L	9.4	12.6	8.709262	1.69E-06	0.0003004
TC1500010723.hg.1	CHAC1	6.1	9.5	10.26502	1.77E-06	0.00031106
TC1000006816.hg.1	OPTN	6.8	10.2	10.41182	1.85E-06	0.00032085

TC1700008455.hg.1	YPEL2	3.1	6.2	8.555546	2.00E-06	0.0003343
TC1700009397.hg.1	TLCD2	5.8	9.2	11.13963	2.03E-06	0.00033731
TC1100009306.hg.1	SC5D	6.2	9.6	10.31181	2.14E-06	0.00034305
TC0200012956.hg.1	GFPT1	11.6	14.6	8.188674	2.15E-06	0.00034305
TC0100011406.hg.1	CD55	9.3	12.5	9.030212	2.19E-06	0.00034305
TC0600008109.hg.1	VEGFA	10.6	14.1	11.38321	2.21E-06	0.0003439
TC0200007132.hg.1	YPEL5	7.0	10.7	12.59754	2.30E-06	0.00035441
TC1600010978.hg.1	PKD1L2	6.3	10.2	15.41545	2.31E-06	0.00035441
TC1000006924.hg.1	ARL5B	11.3	14.3	8.200914	2.37E-06	0.00035746
TC0900012209.hg.1	TTC39B	9.2	12.1	7.78043	2.39E-06	0.00035867
TC0700013441.hg.1	IFRD1	11.8	14.9	8.453889	2.43E-06	0.00036226
TC0600007530.hg.1	HLA-E	8.2	11.6	10.71853	2.60E-06	0.00037128
TC0700013579.hg.1	SEMA3A	10.0	13.3	9.82522	2.87E-06	0.00039222
TC0100009621.hg.1	PHGDH	8.7	11.9	9.104912	3.18E-06	0.00042261
TC1700010762.hg.1	VAT1	10.4	13.4	8.121969	3.19E-06	0.00042261
TC0700008003.hg.1	CLIP2	6.2	9.4	9.257836	3.36E-06	0.00043157
TC0400007280.hg.1	WDR19	9.2	12.4	9.375432	3.44E-06	0.00043632
TC0300014048.hg.1	NPHP3-ACAD11	9.4	12.3	7.424299	3.46E-06	0.00043662
TC1700010811.hg.1	HDAC5	6.0	9.9	15.45423	3.77E-06	0.00046553
TC1400010644.hg.1	SERPINA3	13.9	16.7	6.883321	3.84E-06	0.00047026
TC0600012299.hg.1	SLC17A5	7.7	10.7	7.957362	4.02E-06	0.00048041
TC0900011523.hg.1	HSPA5	14.5	17.2	6.771756	4.10E-06	0.00048041
TC0100008145.hg.1	TSPAN1	12.0	14.9	7.023376	4.17E-06	0.00048416
TC2200008103.hg.1	YPEL1	5.9	8.8	7.770623	4.18E-06	0.00048416
TC2100007446.hg.1	COL6A1	9.8	12.6	6.714732	4.22E-06	0.00048605
TC0X00007310.hg.1	TSPYL2	10.8	14.3	11.24565	4.49E-06	0.00050649
TC2200007318.hg.1	H1F0	9.8	12.6	7.117224	4.63E-06	0.0005185
TC0400012818.hg.1	CCNG2	5.9	9.2	9.879411	4.64E-06	0.0005185
TC0800009868.hg.1	TNFRSF10B	10.3	13.0	6.498132	4.77E-06	0.00052571
TC1900006508.hg.1	ABCA7	8.3	11.5	8.609324	4.81E-06	0.00052653
TC0600008632.hg.1	BCKDHB	10.6	13.3	6.605281	4.98E-06	0.00053942
TC0300012482.hg.1	RAB6B	7.1	10.0	7.813208	5.04E-06	0.00054305
TC1900011817.hg.1	ZNF773	7.4	10.1	6.712662	5.21E-06	0.00055181
TC1200007137.hg.1	FGFR1OP2	9.0	11.6	6.435024	5.41E-06	0.00056036
TC1700006524.hg.1	SERPINF1	12.0	14.7	6.580857	5.53E-06	0.00056569
TC0X00011277.hg.1	CA5B	9.4	12.0	6.28795	5.62E-06	0.00056569
TC1700010738.hg.1	EZH1	8.1	10.8	6.850551	5.68E-06	0.0005669
TC0200015578.hg.1	IDH1	10.3	13.2	7.355893	5.82E-06	0.00057429
TC0200016471.hg.1	MXD1	7.5	10.5	8.159213	6.03E-06	0.00059058
TC0200015790.hg.1	CHPF	4.6	7.2	6.275738	6.37E-06	0.00061747
TC2000006577.hg.1	PRND	4.6	7.9	9.589861	6.38E-06	0.00061747
TC1800007091.hg.1	ELP2	10.2	12.8	6.247915	6.48E-06	0.00061747
TC0100018507.hg.1	ARHGEF2	11.6	14.7	8.371988	6.51E-06	0.00061747
TC0400010282.hg.1	SEL1L3	5.3	8.9	12.80358	6.52E-06	0.00061747
TC1900007945.hg.1	ZNF529-AS1	6.0	8.6	6.430808	6.55E-06	0.00061747
TC0100010112.hg.1	SLC50A1	9.5	12.4	7.133721	6.59E-06	0.00061747
TC0300006994.hg.1	FBXL2	7.0	9.5	6.032139	6.65E-06	0.00061747
TC2200009281.hg.1	PNPLA3	7.0	10.4	10.26666	6.65E-06	0.00061747
TC1400006663.hg.1	LRP10	10.5	13.2	6.546799	6.73E-06	0.00061841
TC0100014617.hg.1	SLC44A5	7.5	10.5	7.863164	7.18E-06	0.00065251

TC0300014092.hg.1	TM4SF19-TCTEX1	5.2	8.0	7.018973	7.28E-06	0.00065815
TC0700008582.hg.1	SERPINE1	11.9	14.6	6.49633	7.35E-06	0.00065933
TC0100014774.hg.1	C1orf52	8.8	11.4	6.315951	7.41E-06	0.00066247
TC0800008783.hg.1	SQLE	8.3	11.0	6.653022	7.46E-06	0.00066373
TC0700009399.hg.1	TAS2R4	7.0	9.6	6.180291	7.68E-06	0.00066633
TC0600007701.hg.1	PHF1	7.6	10.5	7.227942	7.68E-06	0.00066633
TC0X00006671.hg.1	MOSPD2	7.1	10.0	7.1292	7.69E-06	0.00066633
TC1800009222.hg.1	RNF125	7.1	9.9	7.02148	7.84E-06	0.00067377
TC1900006602.hg.1	ZNF555	8.7	11.3	6.021698	7.91E-06	0.00067377
TC2000006444.hg.1	TRIB3	6.4	9.5	8.300537	8.10E-06	0.00068201
TC0700013584.hg.1	CYP51A1	12.8	15.5	6.394826	8.11E-06	0.00068201
TC1200010615.hg.1	LMBR1L	5.8	8.5	6.140389	8.25E-06	0.00069061
TC1700010565.hg.1	FBXL20	8.8	11.3	5.594813	8.67E-06	0.00069711
TC2000006446.hg.1	RBCK1	8.8	11.4	6.194616	8.73E-06	0.00069711
TC0600007480.hg.1	HLA-F	8.5	11.2	6.587061	8.96E-06	0.00070485
TC0100007552.hg.1	SESN2	7.5	10.2	6.591331	8.97E-06	0.00070485
TC1400010732.hg.1	EGLN3	4.7	7.7	8.161474	8.97E-06	0.00070485
TC1700010590.hg.1	GSDMB	5.8	9.2	10.12619	9.21E-06	0.00071573
TC0600011821.hg.1	GUCA1B	4.8	9.4	23.34651	9.42E-06	0.00072883
TC0100018166.hg.1	CLCN6	10.0	12.8	6.946421	9.62E-06	0.00073418
TC1100013148.hg.1	PIK3C2A	12.5	15.0	5.565263	9.85E-06	0.00074075
TC0300008989.hg.1	SLC25A36	11.4	14.0	6.331579	9.85E-06	0.00074075
TC1200007861.hg.1	LRP1	5.9	9.0	8.36665	9.86E-06	0.00074075
TC1800006513.hg.1	TGIF1	10.7	13.1	5.387573	9.88E-06	0.00074075
TC1000011660.hg.1	MGEA5	8.1	11.3	8.882865	9.93E-06	0.00074187
TC0800010417.hg.1	PCMTD1	9.1	11.8	6.615545	1.01E-05	0.00074647
TC1000006604.hg.1	AKR1E2	11.3	13.7	5.274759	1.06E-05	0.00076901
TC0600014318.hg.1	FAM46A	6.9	9.5	6.144902	1.11E-05	0.00078897
TC0500010932.hg.1	SGTB	8.1	10.5	5.346873	1.11E-05	0.00078897
TC1900008103.hg.1	PLD3	10.8	13.3	5.792095	1.13E-05	0.00079456
TC0500008785.hg.1	EGR1	11.0	13.3	5.165829	1.16E-05	0.00080338
TC1100008790.hg.1	C11orf54	7.8	10.5	6.543625	1.17E-05	0.00080778
TC1700008151.hg.1	NFE2L1	12.6	15.2	5.862111	1.19E-05	0.00081478
TC0200014991.hg.1	GPR155	6.7	9.5	6.815762	1.21E-05	0.00082013
TC0900012167.hg.1	GSN	12.5	14.9	5.295733	1.24E-05	0.00083533
TC1200010292.hg.1	AMN1	5.4	7.8	5.125651	1.28E-05	0.00084444
TC2100007362.hg.1	LRRC3	6.9	9.6	6.420275	1.30E-05	0.00085085
TC0600014371.hg.1	RNASET2	6.7	9.4	6.152019	1.31E-05	0.00085085
TC0300012048.hg.1	ZBTB20; MIR568	4.5	7.2	6.271801	1.32E-05	0.00085475
TC0400011695.hg.1	SEC24D	8.6	11.1	5.90043	1.36E-05	0.0008762
TC0900009825.hg.1	DDX58	6.5	8.9	5.315296	1.37E-05	0.00087873
TC1100009817.hg.1	PHLDA2	7.2	9.7	5.593824	1.37E-05	0.00087873
TC2100008560.hg.1	DONSON; CRYZL1	10.0	12.4	5.37487	1.40E-05	0.00088928
TC0200014990.hg.1	CIR1	8.3	10.7	5.263327	1.41E-05	0.00089106
TC1600010752.hg.1	AARS	13.7	16.1	5.518555	1.43E-05	0.00089359
TC0X00007573.hg.1	OGT	14.7	17.0	4.910756	1.43E-05	0.00089359
TC1100010063.hg.1	TMEM41B	12.1	14.7	5.841724	1.44E-05	0.00089359
TC1100011514.hg.1	DHCR7	14.2	16.6	5.155725	1.45E-05	0.00089408
TC0700007098.hg.1	GARS	12.4	14.7	5.1079	1.47E-05	0.00089863
TC0200008894.hg.1	MERTK	6.5	9.1	6.290829	1.49E-05	0.00090791

TC2000009504.hg.1	ZNF217	14.1	16.7	5.979088	1.50E-05	0.00091162
TC1900010574.hg.1	ZNF571	7.2	9.5	4.987561	1.52E-05	0.00092243
TC0400011087.hg.1	CCNI	12.9	15.2	5.063412	1.53E-05	0.00092379
TC1100012959.hg.1	TIMM10B	11.1	13.5	5.258587	1.53E-05	0.00092379
TC0300013828.hg.1	RBM6	10.4	12.7	4.984142	1.54E-05	0.00092547
TC0700008786.hg.1	DNAJB9	8.3	11.0	6.368021	1.57E-05	0.00093656
TC0500011672.hg.1	PJA2	10.4	13.1	6.610066	1.57E-05	0.00093656
TC0100016678.hg.1	FAM129A	3.7	6.8	8.505818	1.59E-05	0.00094171
TC0X00008481.hg.1	FAM122C	6.6	9.5	7.450693	1.60E-05	0.00094599
TC0600014111.hg.1	SYNGAP1; MIR500	10.0	12.4	5.293455	1.61E-05	0.00095059
TC0800008062.hg.1	ZBTB10	6.6	9.0	5.348984	1.65E-05	0.00096423
TC0X00011404.hg.1	IDS	8.7	11.0	4.827196	1.74E-05	0.000997
TC0500009706.hg.1	SQSTM1	11.3	13.6	4.751079	1.78E-05	0.00100608
TC1500010869.hg.1	CCPG1; MIR628	7.3	9.8	5.684388	1.90E-05	0.00105354
TC0600011197.hg.1	ZNF184	8.0	10.4	5.069592	1.90E-05	0.00105405
TC0200006674.hg.1	KLF11	6.7	9.2	5.589948	1.92E-05	0.00106211
TC1600010740.hg.1	PDXDC2P	9.2	11.6	5.251266	1.95E-05	0.00106815
TC0200007471.hg.1	RHOQ	8.2	10.4	4.855382	1.99E-05	0.00108161
TC1100010897.hg.1	UBE2L6	7.8	10.1	4.855499	2.00E-05	0.00108161
TC0600014256.hg.1	GABBR1	8.6	10.8	4.619432	2.02E-05	0.00108684
TC1700008082.hg.1	MAPT	7.3	9.8	5.761941	2.04E-05	0.00109031
TC1900011816.hg.1	ZNF419	7.1	9.4	4.844607	2.04E-05	0.00109031
TC1200009524.hg.1	ZNF84	9.1	11.4	4.924542	2.07E-05	0.00109512
TC0200009978.hg.1	PDK1	8.6	11.2	5.881343	2.07E-05	0.0010961
TC1900007012.hg.1	LDLR; MIR6886	13.4	15.8	5.538191	2.10E-05	0.00110291
TC1000008881.hg.1	MXI1	6.8	9.4	6.097805	2.10E-05	0.00110291
TC1200010812.hg.1	CALCOCO1	7.4	10.0	5.978837	2.11E-05	0.00110522
TC1100011052.hg.1	FADS3	7.8	10.0	4.725085	2.16E-05	0.0011177
TC1100007366.hg.1	TTC17	11.3	13.6	4.744774	2.22E-05	0.00114432
TC0200007401.hg.1	DYNC2LI1	6.5	9.0	5.304531	2.23E-05	0.00114825
TC0800008243.hg.1	PDP1	7.6	10.0	5.025893	2.25E-05	0.00114964
TC1700007997.hg.1	GRN	12.4	14.7	4.871901	2.26E-05	0.00115192
TC2200009236.hg.1	GGT1	10.2	12.4	4.562374	2.26E-05	0.00115192
TC1700007757.hg.1	GRB7	5.7	8.3	5.925964	2.29E-05	0.00116149
TC1000007020.hg.1	OTUD1	3.4	5.7	5.163539	2.30E-05	0.00116523
TC1400010780.hg.1	ATXN3	7.5	9.8	5.08055	2.33E-05	0.00117228
TC0600011880.hg.1	GTPBP2	9.3	11.8	5.562236	2.34E-05	0.00117308
TC0700012203.hg.1	COG5	13.2	15.4	4.685213	2.35E-05	0.0011761
TC0600014258.hg.1	HLA-B	9.3	12.3	8.127037	2.37E-05	0.00118014
TC1900011153.hg.1	RRAS	6.7	9.7	8.098389	2.38E-05	0.00118014
TC0700008072.hg.1	RHBDD2	10.7	13.4	6.202178	2.39E-05	0.00118014
TC1700012282.hg.1	SPATA20	6.1	8.7	6.056545	2.39E-05	0.00118014
TC0900007667.hg.1	PSAT1	13.5	15.8	4.85619	2.41E-05	0.00118059
TC0600011463.hg.1	NEU1	7.5	9.7	4.615093	2.42E-05	0.00118059
TC0600007518.hg.1	HLA-L	7.9	10.4	5.410869	2.42E-05	0.00118059
TC1200008184.hg.1	TBC1D15	8.7	11.1	5.415202	2.44E-05	0.0011856
TC1300007228.hg.1	WDFY2	6.3	8.6	4.957422	2.46E-05	0.0011882
TC0600007495.hg.1	HLA-A	12.8	15.3	5.575961	2.48E-05	0.00118845
TC1300010030.hg.1	N4BP2L2	11.7	13.9	4.695701	2.48E-05	0.00118845
TC1400006652.hg.1	ABHD4	5.3	8.1	6.883082	2.50E-05	0.00119126

TC1900009201.hg.1	MKNK2	9.4	12.4	7.959399	2.54E-05	0.00120113
TC0900011300.hg.1	TNFSF15	3.9	6.0	4.365313	2.55E-05	0.00120379
TC1400009842.hg.1	SEL1L	11.2	13.5	4.833723	2.58E-05	0.00121512
TC0100009339.hg.1	TMEM167B	8.0	10.2	4.586981	2.61E-05	0.00121843
TC0100015594.hg.1	ITGA10	5.3	8.4	8.444196	2.65E-05	0.00122106
TC1900008343.hg.1	QPCTL	8.5	11.1	5.876045	2.65E-05	0.00122164
TC2000008946.hg.1	GGT7	5.1	8.6	11.66496	2.68E-05	0.00122698
TC1100013121.hg.1	BET1L	7.6	9.9	4.893478	2.70E-05	0.00123522
TC0100018508.hg.1	ARHGEF2	6.2	8.5	4.822212	2.80E-05	0.00126227
TC0100018261.hg.1	GSTM4	6.0	8.3	4.922956	2.82E-05	0.00126899
TC1900007829.hg.1	GRAMD1A	9.7	11.9	4.720614	2.83E-05	0.00127347
TC2200009337.hg.1	GATSL3	6.8	9.5	6.781785	2.84E-05	0.00127391
TC1100007394.hg.1	CD82	6.7	8.8	4.225714	2.88E-05	0.00128515
TC0900010580.hg.1	GKAP1	5.5	7.9	5.276312	2.89E-05	0.00128515
TC1500010787.hg.1	LINC01578	9.7	12.3	5.853199	2.89E-05	0.00128515
TC1000010961.hg.1	PSAP	13.8	15.9	4.239356	2.89E-05	0.00128515
TC1400007227.hg.1	LGALS3	11.0	13.2	4.537146	2.92E-05	0.00129213
TC0100016000.hg.1	MEF2D	8.5	10.8	4.882297	2.98E-05	0.00129911
TC1400009337.hg.1	DHRS7	7.6	9.7	4.213414	3.03E-05	0.00131304
TC0500008558.hg.1	PRRC1	10.8	13.0	4.563465	3.03E-05	0.00131304
TC0X00009256.hg.1	KLHL15	11.7	13.8	4.172421	3.10E-05	0.00132649
TC1100009374.hg.1	VWA5A	5.4	7.9	5.580136	3.10E-05	0.00132649
TC0900010886.hg.1	PTCH1	7.4	9.6	4.57912	3.12E-05	0.00132734
TC1600011427.hg.1	IST1	11.5	13.5	4.177022	3.15E-05	0.00133158
TC1900007947.hg.1	ZNF382	6.3	8.5	4.451171	3.16E-05	0.00133224
TC1700007851.hg.1	EIF1	14.5	16.6	4.161145	3.16E-05	0.00133342
TC1700009861.hg.1	TVP23C; CDRT4; T	11.8	14.0	4.547394	3.18E-05	0.00133732
TC0500010869.hg.1	ELOVL7	3.7	5.8	4.186501	3.20E-05	0.00134162
TC0800006978.hg.1	POLR3D	10.1	12.1	4.243354	3.21E-05	0.00134162
TC0100018451.hg.1	GBP2	6.0	8.1	4.349682	3.22E-05	0.00134547
TC1900011327.hg.1	ZNF160	9.0	11.2	4.703405	3.23E-05	0.0013457
TC1900007777.hg.1	CEBPG	9.4	11.9	5.772214	3.35E-05	0.00138481
TC0400012150.hg.1	TMEM154	7.1	9.2	4.314116	3.43E-05	0.00140038
TC0700012584.hg.1	UBE2H	11.6	14.2	5.883256	3.43E-05	0.00140038
TC1900007382.hg.1	PGPEP1	7.0	9.6	6.220324	3.52E-05	0.00142077
TC1100009824.hg.1	CARS	9.6	12.1	5.31572	3.57E-05	0.00143425
TC2200009336.hg.1	NIPSNAP1	7.0	9.0	4.055951	3.57E-05	0.00143425
TC1400010071.hg.1	CLMN	6.1	8.7	6.224248	3.65E-05	0.00145337
TC1100007948.hg.1	PRDX5	9.2	11.3	4.23677	3.66E-05	0.00145509
TC0100011419.hg.1	CD46	12.4	14.5	4.067536	3.68E-05	0.00146058
TC1500009200.hg.1	TTBK2	7.4	9.4	4.046641	3.72E-05	0.00147064
TC1600011409.hg.1	PDP2	10.2	12.3	4.089064	3.73E-05	0.00147064
TC1400009524.hg.1	ZFP36L1	14.6	16.7	4.416609	3.79E-05	0.00148209
TC1900010947.hg.1	EML2; MIR330	7.4	9.4	4.029735	3.84E-05	0.00149579
TC0400011994.hg.1	INPP4B	8.7	10.7	4.055008	3.84E-05	0.00149648
TC0600007092.hg.1	FAM8A1	10.6	12.6	3.998379	3.91E-05	0.0015094
TC1600010375.hg.1	CRNDE	10.3	12.3	4.186995	3.94E-05	0.00151794
TC0800010458.hg.1	TCEA1	11.2	13.6	5.146655	3.96E-05	0.001518
TC0900012156.hg.1	PALM2	4.3	6.4	4.267053	3.98E-05	0.001518
TC1000009612.hg.1	KLF6	13.4	15.5	4.41698	4.20E-05	0.00157236

TC1500010369.hg.1	MFGE8	9.8	12.0	4.576074	4.22E-05	0.00157236
TC0600009368.hg.1	HINT3	10.0	12.0	4.161562	4.22E-05	0.00157236
TC0700006629.hg.1	FSCN1	10.5	12.7	4.537522	4.24E-05	0.00157774
TC1100009168.hg.1	SIDT2	9.4	11.4	4.045604	4.35E-05	0.00160676
TC1100011192.hg.1	ATG2A	9.4	11.5	4.157366	4.45E-05	0.00163544
TC1800007276.hg.1	CTIF; MIR4743	3.9	6.0	4.355837	4.51E-05	0.00165272
TC1100012949.hg.1	IFITM1	12.8	15.1	4.962167	4.57E-05	0.00166661
TC1700010592.hg.1	ORMDL3	10.7	12.7	4.048733	4.64E-05	0.00168406
TC0700013396.hg.1	CLDN12	9.5	11.6	4.206906	4.65E-05	0.00168406
TC0700006619.hg.1	SLC29A4	7.8	10.6	6.895857	4.67E-05	0.00168775
TC0400011464.hg.1	MANBA	7.8	10.1	4.802032	4.85E-05	0.00171986
TC2200008475.hg.1	PIK3IP1	5.6	7.6	4.154025	4.87E-05	0.00171986
TC0900010352.hg.1	PTAR1	11.1	13.3	4.595192	4.89E-05	0.00172189
TC0100013949.hg.1	SLC6A9	9.9	12.1	4.757489	4.90E-05	0.00172358
TC1500010890.hg.1	HEXA	10.3	12.2	3.796355	4.94E-05	0.00172698
TC1200007881.hg.1	INHBE	4.3	6.3	3.933245	4.98E-05	0.00173553
TC0500009319.hg.1	CCNG1	11.0	13.2	4.675126	5.04E-05	0.00174576
TC0200012068.hg.1	SLC30A3	6.1	8.1	4.137158	5.05E-05	0.00174631
TC0700008517.hg.1	ZKSCAN1	12.7	14.6	3.847377	5.08E-05	0.00174992
TC0X00010671.hg.1	LAMP2	11.9	13.9	3.868689	5.10E-05	0.00174992
TC1200010968.hg.1	DDIT3	5.2	7.5	4.827379	5.11E-05	0.00174992
TC0X00006581.hg.1	TBL1X	9.9	11.9	4.058918	5.12E-05	0.00174992
TC0900010762.hg.1	AUH	8.7	10.8	4.312949	5.16E-05	0.00174992
TC1900007955.hg.1	ZNF345	4.5	6.7	4.573664	5.17E-05	0.00174992
TC1000007990.hg.1	DDIT4	10.0	12.3	4.793487	5.17E-05	0.00174992
TC1700007017.hg.1	SNORD49A; SNOR	13.4	15.3	3.780298	5.19E-05	0.00174992
TC1700007138.hg.1	TVP23B	12.5	14.4	3.87892	5.20E-05	0.00174992
TC0700007067.hg.1	MTURN	5.3	8.0	6.443904	5.21E-05	0.00174992
TC0300012765.hg.1	HLTF	7.3	9.7	5.083423	5.22E-05	0.00175177
TC1600011440.hg.1	OSGIN1	8.2	10.3	4.337834	5.23E-05	0.00175204
TC2100007491.hg.1	CBS	10.8	12.7	3.80406	5.27E-05	0.00176195
TC0900012158.hg.1	PALM2-AKAP2	8.0	10.0	4.030364	5.30E-05	0.00176846
TC0300010714.hg.1	TRANK1	3.8	5.9	4.325854	5.43E-05	0.00179084
TC1000010482.hg.1	C10orf10	6.6	8.5	3.757452	5.45E-05	0.00179448
TC1800008285.hg.1	NPC1	9.7	11.7	4.024807	5.47E-05	0.00180072
TC1700008175.hg.1	CALCOCO2	11.5	13.4	3.723192	5.49E-05	0.00180084
TC0200015350.hg.1	RFTN2	3.8	5.7	3.71596	5.50E-05	0.00180141
TC0100009344.hg.1	SARS	10.4	12.9	5.823051	5.54E-05	0.00180584
TC0300008563.hg.1	DIRC2	9.0	11.3	4.888578	5.71E-05	0.00183441
TC0600012544.hg.1	RRAGD	7.5	9.6	4.495027	5.75E-05	0.00183441
TC1100010077.hg.1	SBF2	10.1	12.0	3.692109	5.75E-05	0.00183441
TC2000008130.hg.1	TBC1D20	10.1	12.0	3.794197	5.88E-05	0.00186966
TC0700007633.hg.1	SUMF2	12.1	14.1	3.864148	5.94E-05	0.0018831
TC1700007319.hg.1	WSB1	12.3	14.2	3.861257	5.98E-05	0.00189126
TC1900008564.hg.1	ATF5; MIR4751	8.8	11.4	6.180058	6.01E-05	0.00189836
TC1700010563.hg.1	CACNB1	8.4	10.5	4.222538	6.09E-05	0.00191535
TC1900010941.hg.1	ERCC1	11.5	13.5	3.785202	6.15E-05	0.00192818
TC1700011448.hg.1	POLG2	9.4	11.6	4.478808	6.17E-05	0.00192961
TC1300009766.hg.1	IRS2	5.0	7.1	4.074124	6.18E-05	0.00192961
TC0700012479.hg.1	POT1	9.6	11.6	3.835595	6.24E-05	0.00193876

TC1100013040.hg.1	TM7SF2	9.2	11.4	4.373961	6.35E-05	0.00195964
TC0200016193.hg.1	COL6A3	9.7	11.7	3.970097	6.37E-05	0.00196
TC0100014794.hg.1	ODF2L	7.4	9.3	3.737116	6.38E-05	0.00196
TC1100010688.hg.1	PHF21A	11.1	12.9	3.635856	6.40E-05	0.00196015
TC1900009754.hg.1	DNASE2	8.2	10.5	4.972686	6.50E-05	0.00198455
TC0400011749.hg.1	BBS7	8.5	10.4	3.640357	6.51E-05	0.00198646
TC0600014143.hg.1	PHF3	12.1	14.0	3.831303	6.59E-05	0.00199609
TC0X00009541.hg.1	SLC9A7	8.9	10.8	3.813696	6.60E-05	0.00199609
TC1500010842.hg.1	GOLGA8R	9.3	11.2	3.693247	6.76E-05	0.00203394
TC0300010664.hg.1	GLB1; TMPPE	9.9	11.9	3.945587	6.77E-05	0.00203423
TC0900009779.hg.1	C9orf72	3.8	6.4	6.313697	6.79E-05	0.00203507
TC1200012190.hg.1	OASL	6.5	8.8	4.721092	6.83E-05	0.00203507
TC0100010798.hg.1	QSOX1	10.7	12.7	3.984448	6.84E-05	0.00203507
TC1700012460.hg.1	ABCA5	7.4	9.4	3.824099	6.90E-05	0.00205036
TC0600011536.hg.1	TAPBP	13.4	15.4	3.995789	6.93E-05	0.00205101
TC0500011194.hg.1	F2RL2	10.6	12.5	3.693427	6.96E-05	0.00205489
TC1000007950.hg.1	UNC5B	4.1	6.0	3.873909	7.08E-05	0.00208108
TC0300010038.hg.1	SENP5	6.9	8.7	3.56465	7.10E-05	0.00208268
TC1000012577.hg.1	LIPA	15.5	17.3	3.531265	7.14E-05	0.00208952
TC0700011562.hg.1	TMEM120A	9.5	11.4	3.843605	7.18E-05	0.00209744
TC2200007037.hg.1	MTMR3	7.5	9.3	3.543439	7.26E-05	0.00211811
TC1300009765.hg.1	IRS2	5.4	7.3	3.769975	7.42E-05	0.00215306
TC1400009973.hg.1	RPS6KA5	5.7	8.2	5.463158	7.49E-05	0.0021637
TC2000009250.hg.1	PLTP	7.8	10.1	4.998309	7.50E-05	0.0021637
TC0800010016.hg.1	SARAF	8.0	10.1	4.152945	7.58E-05	0.00218204
TC1100011590.hg.1	RAB6A	13.7	15.5	3.534657	7.64E-05	0.00219592
TC0300012918.hg.1	CCNL1	10.7	12.8	4.209503	7.66E-05	0.00220041
TC0200016751.hg.1	LOC100130691; AC	5.2	7.1	3.736934	7.77E-05	0.00222504
TC0500008702.hg.1	CAMLG	8.9	10.8	3.892306	7.84E-05	0.00223738
TC1900011946.hg.1	FBXO27	11.3	13.2	3.61985	7.84E-05	0.00223738
TC0100007574.hg.1	EPB41	11.9	14.0	4.369838	7.95E-05	0.00225953
TC0200014772.hg.1	IFIH1	4.7	6.7	4.113824	8.00E-05	0.00226964
TC0700009398.hg.1	TAS2R3	5.6	7.5	3.627373	8.03E-05	0.00227311
TC1900009164.hg.1	PCSK4	5.2	7.4	4.626737	8.14E-05	0.00229355
TC0X00011198.hg.1	DNASE1L1	8.2	10.3	4.279627	8.20E-05	0.00230463
TC0100008536.hg.1	INADL	7.6	9.8	4.629799	8.22E-05	0.00230898
TC2200007406.hg.1	ATF4	10.7	12.5	3.681952	8.27E-05	0.00231715
TC1900011894.hg.1	CACNA1A	9.0	10.8	3.507177	8.28E-05	0.00231715
TC1400009732.hg.1	C14orf1	13.4	15.3	3.770772	8.29E-05	0.00231801
TC1600009865.hg.1	NUPR1	5.6	9.2	12.15481	8.32E-05	0.00232166
TC1900011084.hg.1	DBP	6.5	8.4	3.658418	8.40E-05	0.00233616
TC2100008285.hg.1	CBS	11.0	12.9	3.592991	8.58E-05	0.00236546
TC2100007996.hg.1	TMEM50B	11.2	13.3	4.091926	8.58E-05	0.00236546
TC0800011150.hg.1	TP53INP1	3.8	6.8	7.975417	8.65E-05	0.00237742
TC1600011186.hg.1	SLC7A5	15.2	17.0	3.462863	8.67E-05	0.00237742
TC0700007870.hg.1	STAG3L4	9.5	11.5	4.02365	8.68E-05	0.00237742
TC0500011125.hg.1	ANKRA2	6.7	8.6	3.736973	8.72E-05	0.00238094
TC0800010158.hg.1	PLPP5	7.3	9.6	5.058379	8.75E-05	0.00238183
TC1700012191.hg.1	CD68	8.6	10.8	4.551541	8.81E-05	0.00239103
TC0900006758.hg.1	DENND4C	8.5	10.5	4.183048	8.82E-05	0.00239132

TC1600011527.hg.1	VKORC1	13.2	15.0	3.504358	8.86E-05	0.00239308
TC0300013962.hg.1	ANO10	8.7	10.6	3.663213	8.89E-05	0.00239894
TC0100009032.hg.1	BTBD8	7.0	9.5	5.689631	8.97E-05	0.00240128
TC1700011773.hg.1	WBP2	10.2	12.1	3.727375	9.03E-05	0.00240928
TC1700007777.hg.1	THRA	9.4	11.2	3.530162	9.03E-05	0.00240928
TC0200013079.hg.1	DUSP11	10.9	12.8	3.658951	9.04E-05	0.00240928
TC0400011440.hg.1	PPP3CA	4.3	6.8	6.007917	9.08E-05	0.00241351
TC0600007103.hg.1	KDM1B	10.4	12.2	3.410102	9.17E-05	0.0024296
TC0600013054.hg.1	SERINC1	11.8	13.6	3.481453	9.18E-05	0.0024296
TC1400009684.hg.1	ALDH6A1	6.5	8.3	3.566131	9.20E-05	0.0024296
TC1900010782.hg.1	ATP1A3	6.9	9.2	4.979203	9.21E-05	0.0024296
TC1500007379.hg.1	FAM63B	7.7	9.5	3.626518	9.22E-05	0.0024296
TC1900011940.hg.1	ECH1	11.1	13.1	3.962703	9.31E-05	0.00243922
TC0200009955.hg.1	CYBRD1	14.2	16.1	3.575908	9.34E-05	0.00243965
TC0100017849.hg.1	ERO1B	6.2	8.7	5.693398	9.34E-05	0.00243965
TC1200008900.hg.1	ERP29	8.7	10.6	3.921361	9.41E-05	0.00245595
TC0900008811.hg.1	STXBP1	11.7	13.6	3.657429	9.45E-05	0.00246093
TC0700006928.hg.1	CCDC126	7.6	9.4	3.393161	9.47E-05	0.00246093
TC0200012433.hg.1	PREPL	10.1	12.0	3.625348	9.48E-05	0.00246097
TC1700012353.hg.1	PER1; MIR6883	6.8	10.2	10.62934	9.59E-05	0.00247711
TC0100012089.hg.1	GPR137B	7.0	8.8	3.438264	9.64E-05	0.00248764
TC1700011261.hg.1	4-Sep	4.7	6.8	4.081333	9.72E-05	0.0024972
TC0500007758.hg.1	FCHO2	8.4	10.3	3.742884	9.79E-05	0.0025081
TC2200009248.hg.1	KREMEN1	7.5	9.2	3.356867	9.81E-05	0.00251181
TC2000007197.hg.1	TP53INP2	3.8	5.7	3.516171	0.00010125	0.00257603
TC0500011752.hg.1	FEM1C	6.3	8.3	4.046627	0.00010156	0.0025779
TC0200007999.hg.1	ZNF638	8.6	10.4	3.309757	0.00010194	0.00258318
TC1600009524.hg.1	NPIPA5	9.9	11.8	3.988566	0.00010217	0.00258401
TC0X00011382.hg.1	ACSL4	9.2	11.0	3.466573	0.00010271	0.00259483
TC1700012200.hg.1	CCDC144A	9.8	11.8	3.993378	0.00010317	0.00260326
TC1800006786.hg.1	RNMT	10.4	12.2	3.319096	0.00010402	0.00262174
TC0400007857.hg.1	AREG	5.8	7.7	3.74627	0.00010444	0.00262704
TC0400008609.hg.1	KIAA1109	9.5	11.6	4.339684	0.00010609	0.00265484
TC2000008852.hg.1	NOL4L	5.7	7.9	4.446746	0.00010684	0.00265913
TC2200009356.hg.1	ARFGAP3	7.7	9.6	3.62447	0.00010719	0.00266401
TC0100010123.hg.1	FDPS	10.8	12.8	3.841687	0.00010845	0.00268913
TC1700010707.hg.1	DHX58	6.9	8.9	3.909015	0.00010924	0.00269308
TC1900006480.hg.1	PALM	5.9	7.6	3.305091	0.00011118	0.00272215
TC1700011305.hg.1	HEATR6	9.8	11.9	4.122515	0.0001117	0.00272548
TC0600011173.hg.1	GUSBP2	13.9	15.6	3.256743	0.00011202	0.00272718
TC0600007402.hg.1	ZKSCAN8	11.2	13.1	3.604616	0.00011281	0.00274211
TC0600007301.hg.1	BTN3A2	8.0	10.3	4.792987	0.00011355	0.00274238
TC1800007595.hg.1	CCDC102B	5.5	8.0	5.64209	0.00011364	0.00274238
TC1900011759.hg.1	APOC1	8.6	10.3	3.32514	0.00011368	0.00274238
TC1500008933.hg.1	GOLGA8O	10.3	12.0	3.345403	0.00011443	0.00275138
TC0300012118.hg.1	TMEM39A	10.9	12.7	3.527924	0.00011474	0.00275578
TC1300008253.hg.1	CRYL1	6.6	8.8	4.904274	0.00011563	0.00276975
TC0100015932.hg.1	PBXIP1	4.7	6.6	3.654794	0.00011571	0.00276975
TC1800007155.hg.1	PIK3C3	8.2	10.3	4.080271	0.00011608	0.00277254
TC1200006730.hg.1	RIMKLB	8.6	10.5	3.561204	0.00011632	0.00277413

TC1900011305.hg.1	ZNF83	5.3	7.6	4.917705	0.00011641	0.00277413
TC0500007668.hg.1	CDK7	10.4	12.2	3.368006	0.00011719	0.00277736
TC1700007585.hg.1	SLFN5	6.6	8.6	3.914993	0.00011849	0.00278959
TC2100007072.hg.1	TTC3	12.3	14.0	3.238341	0.00011969	0.00281166
TC0200008071.hg.1	MTHFD2	13.8	15.5	3.419546	0.00012078	0.0028325
TC1100010761.hg.1	FNBP4	13.2	15.0	3.573705	0.00012088	0.0028325
TC1400010195.hg.1	WARS	15.6	17.3	3.278055	0.00012097	0.0028325
TC0600012647.hg.1	FBXL4	10.9	12.6	3.240272	0.000123	0.00287696
TC1200007170.hg.1	CCDC91	10.1	11.9	3.4181	0.00012521	0.00290965
TC0100015434.hg.1	GDAP2	10.0	11.9	3.794354	0.00012558	0.00291504
TC1300007197.hg.1	DLEU1	6.8	8.5	3.27626	0.00012581	0.00291715
TC1300009332.hg.1	RBM26	6.7	8.9	4.550625	0.00012667	0.00292768
TC1900008015.hg.1	SPRED3	5.0	6.9	3.540701	0.00012785	0.00294847
TC0400012941.hg.1	THAP9-AS1	10.5	12.3	3.399835	0.00012875	0.00296598
TC1900011449.hg.1	TNNT1	5.3	7.6	4.917075	0.00012959	0.00297693
TC1700010604.hg.1	NR1D1	10.3	12.2	3.710093	0.00012964	0.00297693
TC1200011573.hg.1	NR2C1	9.6	11.3	3.31737	0.00013012	0.00298175
TC1900008562.hg.1	TBC1D17; MIR475(	6.9	9.1	4.707317	0.0001307	0.00298853
TC0200011670.hg.1	KIDINS220	4.5	7.2	6.562043	0.00013185	0.00300843
TC2200008370.hg.1	XBP1	9.7	11.5	3.406086	0.00013331	0.00302394
TC0400008106.hg.1	HERC5	4.0	6.0	3.910193	0.00013403	0.00302929
TC1400008750.hg.1	DHRS1	3.8	5.5	3.215058	0.00013518	0.00304879
TC0100010369.hg.1	ATF6	11.4	13.1	3.241732	0.00013639	0.0030645
TC0600013258.hg.1	AHI1	8.8	10.8	3.977964	0.00013645	0.0030645
TC0800009673.hg.1	LONRF1	4.1	6.3	4.805345	0.00013694	0.00306962
TC0500008691.hg.1	UBE2B	9.8	11.9	4.357044	0.00013785	0.00308298
TC2200006517.hg.1	CECR2	6.7	8.9	4.581564	0.00013864	0.00308787
TC1200010592.hg.1	ADCY6; MIR4701	7.8	9.5	3.137249	0.00014038	0.00311049
TC1600009954.hg.1	MAPK3	11.8	13.5	3.201593	0.00014255	0.00314874
TC0500011924.hg.1	ALDH7A1	12.7	14.3	3.188638	0.00014287	0.00315258
TC0100017836.hg.1	LYST	11.1	12.9	3.509742	0.00014424	0.00317948
TC1600009530.hg.1	KIAA0430; MIR650	6.2	8.0	3.523014	0.00014499	0.00318609
TC1900007328.hg.1	SLC27A1	7.5	9.8	4.840664	0.00014558	0.00318609
TC1900008860.hg.1	LENG8	12.4	14.0	3.141253	0.00014746	0.00321088
TC2100006968.hg.1	IFNGR2	8.4	10.2	3.442639	0.00014843	0.00322321
TC1400010584.hg.1	IRF9	6.2	8.7	5.45695	0.00014884	0.00322774
TC1100008847.hg.1	CEP57	9.1	11.1	3.906834	0.00014929	0.00323435
TC1700007942.hg.1	NBR1	10.0	11.7	3.375462	0.00015139	0.00327382
TC2200007196.hg.1	TOM1	6.4	8.1	3.349695	0.00015172	0.00327695
TC0900008474.hg.1	SNX30	6.9	8.7	3.35347	0.00015196	0.00327893
TC1700009982.hg.1	USP32P2; FAM106	11.0	12.6	3.111532	0.0001529	0.00328791
TC1800007071.hg.1	ZNF397	8.9	10.7	3.525665	0.00015494	0.00331987
TC2100006618.hg.1	USP25	10.9	12.6	3.18251	0.00015539	0.00332606
TC1200012451.hg.1	STX2	11.8	13.5	3.145807	0.00015639	0.00333089
TC0100007505.hg.1	WDTC1	7.4	9.2	3.468568	0.00015725	0.00334262
TC0300007115.hg.1	EIF1B	7.8	9.8	3.953746	0.00015795	0.00335362
TC0100007486.hg.1	SFN	4.2	6.7	5.666626	0.00015834	0.00335585
TC2000007943.hg.1	CDH26	4.5	6.1	3.106597	0.00015964	0.00337664
TC0400008628.hg.1	SPRY1	12.9	14.5	3.210561	0.00016013	0.00338036
TC0700009079.hg.1	TSPAN33	6.7	8.5	3.516963	0.00016044	0.00338352

TSUnmapped0000007	NDUFA10	10.8	12.4	3.072026	0.00016108	0.0033937
TC0200010624.hg.1	PIKFYVE	11.6	13.3	3.207926	0.00016206	0.00339712
TC0200014717.hg.1	WDSUB1	7.6	9.4	3.520414	0.00016209	0.00339712
TC0600007552.hg.1	DDR1; MIR4640	7.8	9.7	3.842285	0.00016237	0.00339765
TC0X00007951.hg.1	ARMCX3	8.8	10.5	3.224902	0.00016419	0.00342906
TC1800007797.hg.1	ATP9B	9.7	11.4	3.155732	0.00016802	0.00348847
TC1200011255.hg.1	ZFC3H1	12.4	14.1	3.312027	0.00016872	0.00349679
TC0200012374.hg.1	KCNG3	6.7	8.3	3.10112	0.00016893	0.00349679
TC1000008744.hg.1	TRIM8	6.0	7.8	3.463246	0.0001697	0.00349996
TC1900009190.hg.1	KLF16	9.4	11.2	3.365556	0.00017188	0.00352773
TC1700010447.hg.1	CCL5	4.5	6.7	4.78721	0.00017231	0.00353311
TC1000008727.hg.1	NFKB2	7.0	8.8	3.604828	0.0001735	0.00354477
TC1100012722.hg.1	CDON	6.9	8.9	3.867658	0.00017383	0.00354477
TC0100016963.hg.1	KDM5B	11.8	13.4	3.131305	0.00017399	0.00354477
TC0700006603.hg.1	FOXK1	11.2	12.9	3.073015	0.00017403	0.00354477
TC0400009352.hg.1	CEP44	7.8	9.6	3.409654	0.00017618	0.00358172
TC0600014271.hg.1	NOTCH4	6.0	8.2	4.786811	0.00017655	0.00358235
TC1200009724.hg.1	SCNN1A	11.4	13.1	3.390094	0.00017843	0.00361377
TC0300012195.hg.1	HSPBAP1	4.8	6.8	3.887924	0.00017927	0.00362386
TC1900007963.hg.1	ZNF383	8.3	10.1	3.352157	0.00018242	0.00367371
TC0700013603.hg.1	RASA4	5.0	6.6	3.085149	0.000183	0.00367656
TC0500013399.hg.1	PANK3	11.4	13.3	3.734732	0.00018306	0.00367656
TC0700013338.hg.1	GLCCI1	4.2	6.0	3.575574	0.00018353	0.00367883
TC2100008462.hg.1	LSS	10.6	12.2	3.149899	0.0001844	0.0036894
TC0200013376.hg.1	EIF2AK3	7.6	9.3	3.203702	0.00018645	0.00372506
TC1200012712.hg.1	MAP1LC3B2	2.9	4.7	3.449746	0.00018664	0.00372506
TC0900010910.hg.1	SLC35D2	10.6	12.3	3.103175	0.00018903	0.00376106
TC1800008903.hg.1	KDSR	10.7	12.4	3.232096	0.00019326	0.00382551
TC1200011313.hg.1	NAP1L1	12.0	13.7	3.148883	0.00019334	0.00382551
TC0X00007267.hg.1	MAGED1	9.7	11.3	3.111382	0.00019457	0.00384616
TC1100013032.hg.1	DNAJC4	7.2	8.8	3.055048	0.00019524	0.00385596
TC0700011813.hg.1	BET1	8.0	9.6	2.964164	0.00019553	0.0038581
TC2000007015.hg.1	PYGB	12.7	14.4	3.185237	0.00020206	0.00395411
TC2000007157.hg.1	CBFA2T2	8.3	9.8	2.960406	0.00020477	0.00398279
TC0600008569.hg.1	MYO6	7.8	9.4	3.067695	0.00020493	0.00398279
TC1900012021.hg.1	ZNF841	7.8	9.4	3.089954	0.00021124	0.00407793
TC0100018247.hg.1	LRRC8D	10.6	12.2	3.003126	0.00021164	0.00408122
TC1600008932.hg.1	LUC7L	7.1	8.8	3.233283	0.00021238	0.00408906
TC0600014086.hg.1	ZNF391	7.0	8.6	3.042558	0.00021417	0.0041123
TC1000012011.hg.1	RGS10	8.8	10.5	3.132513	0.00021463	0.00411746
TC0600007684.hg.1	SLC39A7	11.8	13.4	3.066663	0.00021516	0.00412385
TC0600007487.hg.1	HLA-G	8.4	10.1	3.350938	0.00021542	0.00412385
TC0200013261.hg.1	RETSAT	11.8	13.5	3.376023	0.00021554	0.00412385
TC1700010190.hg.1	IFT20	7.5	9.2	3.391702	0.00021656	0.00413604
TC0200010362.hg.1	COQ10B	9.9	11.5	2.904671	0.00021774	0.00415261
TC0100016492.hg.1	RFWD2	8.4	9.9	2.911938	0.00021801	0.00415274
TC0600013794.hg.1	AGPAT4	4.5	6.6	4.242942	0.00022234	0.0042276
TC2000008213.hg.1	DDRGK1	8.8	10.3	2.920084	0.00022366	0.0042452
TC0100016129.hg.1	COPA	13.2	14.7	2.965992	0.00022416	0.00425089
TC1900007290.hg.1	TMEM38A	7.2	8.9	3.214368	0.00022474	0.00425809

TC0900006583.hg.1	UHRF2	14.1	15.6	2.946576	0.00022755	0.00430375
TC1700006772.hg.1	KDM6B	8.2	9.9	3.131708	0.00022779	0.00430448
TC1200012643.hg.1	ERBB3	6.3	8.1	3.308988	0.00022911	0.00432191
TC0800010949.hg.1	SNX16	6.9	8.7	3.698512	0.00023474	0.00440863
TC1600008034.hg.1	NDRG4	5.8	7.4	3.043697	0.00023632	0.00442294
TC1900008341.hg.1	GIPR	3.9	5.7	3.398893	0.00024033	0.0044667
TC1300009810.hg.1	ANKRD10	10.2	11.9	3.240878	0.00024325	0.00449533
TC0300013991.hg.1	TWF2	7.9	9.4	2.929896	0.00024343	0.00449533
TC1600009731.hg.1	COG7	8.2	9.7	2.935833	0.0002443	0.00450535
TC1700012093.hg.1	FASN	12.2	13.8	2.882328	0.00024606	0.00453393
TC0700010899.hg.1	POLR2J4	6.6	8.2	2.89003	0.00024785	0.00455914
TC0600013740.hg.1	RSPH3	8.3	10.4	4.16768	0.00024924	0.00457046
TC1600007505.hg.1	HSD3B7	8.0	9.6	2.939304	0.00024975	0.00457448
TC1500008986.hg.1	LPCAT4	7.6	9.5	3.703848	0.00025124	0.00459778
TC0600010825.hg.1	ADTRP	9.4	11.0	3.006198	0.00025182	0.00460449
TC1900008988.hg.1	ZNF805	5.9	7.7	3.552809	0.00025244	0.0046079
TC0500012425.hg.1	CSNK1A1	13.8	15.3	2.889871	0.00025353	0.00461997
TC1500007695.hg.1	PAQR5	3.4	5.1	3.08158	0.00025459	0.00462755
TC0400012990.hg.1	DDX60L	7.3	8.9	3.148348	0.00025523	0.0046312
TC1000008643.hg.1	SCD	17.1	18.7	3.06317	0.00025582	0.00463807
TC0X00007026.hg.1	USP9X	11.8	13.3	2.838472	0.00025607	0.00463867
TC1500008982.hg.1	KATNBL1	8.2	9.9	3.291408	0.00025638	0.00464044
TC1600011375.hg.1	QPRT	3.7	5.4	3.259047	0.00025821	0.0046569
TC1700008539.hg.1	MRC2	6.4	8.8	5.320345	0.00025878	0.0046603
TC0600011535.hg.1	RGL2	8.7	10.2	2.870606	0.00025945	0.00466273
TC1700012395.hg.1	TBC1D3L	9.1	10.7	2.971519	0.00026023	0.00467061
TC0100007789.hg.1	AGO3	10.3	12.0	3.35102	0.00026124	0.00467708
TC0100015627.hg.1	PRKAB2	7.8	9.3	2.925189	0.00026492	0.00472316
TC1700012344.hg.1	GABARAP	9.4	11.0	3.013009	0.000268	0.00476617
TC1400009184.hg.1	TXNDC16	6.6	8.2	3.02371	0.00026855	0.00477207
TC1700009122.hg.1	SLC26A11	6.6	8.1	2.914836	0.00027206	0.00481858
TC1400010390.hg.1	AHNAK2	12.7	14.2	2.817411	0.00027764	0.00489711
TC1700011313.hg.1	USP32	10.2	11.7	2.861755	0.00027843	0.00490687
TC0600010057.hg.1	ACAT2	8.7	10.4	3.382265	0.00027894	0.00490963
TC0300011936.hg.1	CD47	7.4	9.3	3.64643	0.00027904	0.00490963
TC0100007378.hg.1	CLIC4	11.2	12.7	2.832478	0.00028009	0.00492404
TC2000009957.hg.1	LINC00266-1	7.4	9.0	2.955291	0.00028054	0.00492798
TC0900007457.hg.1	CNTNAP3P2; CNT	8.4	9.9	2.850263	0.0002813	0.00493727
TC1200007895.hg.1	OS9	12.8	14.4	2.925452	0.00028256	0.00495534
TC1900007096.hg.1	JUNB	4.0	6.1	4.044286	0.00028331	0.00496322
TC1600010855.hg.1	GLG1	13.8	15.3	2.918728	0.00028347	0.00496322
TC0600008146.hg.1	RUNX2	9.3	11.2	3.616816	0.00028394	0.00496735
TC0700013400.hg.1	GATAD1	10.7	12.3	2.858335	0.00028478	0.00497007
TC0100015945.hg.1	THBS3	8.9	10.5	3.118635	0.00028522	0.00497007
TC1600007723.hg.1	GPT2	7.5	9.1	3.074454	0.00028524	0.00497007
TC1100008673.hg.1	TMEM135	8.8	10.3	2.835711	0.00028548	0.00497007
TC0200011688.hg.1	ADAM17	11.6	13.2	3.071461	0.00028671	0.00497524
TC0600012240.hg.1	LMBRD1	9.5	11.1	2.966733	0.00028725	0.0049806
TC0200014550.hg.1	ORC4	9.3	10.8	2.822894	0.00029222	0.00502612
TC1200010265.hg.1	CAPRIN2	14.5	16.1	2.990165	0.00029276	0.00503129

TC1200010006.hg.1	PLBD1	13.2	14.6	2.770911	0.00029368	0.00503905
TC1700012484.hg.1	PYCR1	9.2	10.7	2.805776	0.00029621	0.00506229
TC0600009102.hg.1	FIG4	8.3	9.8	2.897503	0.00029704	0.00506841
TC0200007805.hg.1	AFTPH	9.3	10.7	2.789991	0.0002996	0.00510399
TC1900009970.hg.1	BST2	2.4	4.6	4.459617	0.00030303	0.00515065
TC1200007897.hg.1	TSPAN31	7.6	9.6	3.906198	0.00030402	0.00515595
TC0700009827.hg.1	RBM33	10.6	12.1	2.754289	0.0003041	0.00515595
TC1900009382.hg.1	PTPRS	11.9	13.4	2.900678	0.00031022	0.00523912
TC2000008200.hg.1	PCED1A	8.3	9.8	2.842331	0.00031378	0.00527845
TC0100006486.hg.1	AGRN	5.8	7.5	3.296797	0.00031632	0.00530313
TC1300006914.hg.1	UFM1	8.5	10.0	2.75897	0.00031654	0.00530313
TC0100016833.hg.1	ZBTB41	9.2	10.8	2.901954	0.00031673	0.00530313
TC1400010737.hg.1	KLHL28	6.8	8.5	3.226789	0.00031938	0.00533086
TC1700011953.hg.1	CBX4	8.5	10.0	2.79538	0.00032306	0.00537311
TC2200007356.hg.1	KDELR3	7.9	9.5	3.164857	0.00032307	0.00537311
TC0200016332.hg.1	KIF1A	6.9	8.4	2.87658	0.00032914	0.00545132
TC0X00011364.hg.1	ZNF630	5.1	7.0	3.801451	0.00033073	0.00547332
TC1700011919.hg.1	CEP295NL; TIMP2	13.7	15.6	3.692338	0.00033331	0.00551175
TC1900011869.hg.1	RGL3	6.6	8.4	3.345496	0.00033374	0.00551379
TC0900011613.hg.1	FAM102A	7.1	9.0	3.837309	0.00033593	0.00553806
TC1000008758.hg.1	CNNM2	8.7	10.4	3.279464	0.00034061	0.00558087
TC1900009137.hg.1	CBARP	5.8	7.7	3.757648	0.00034072	0.00558087
TC0700009400.hg.1	TAS2R5	6.2	7.8	3.02802	0.00034087	0.00558087
TC0100006771.hg.1	H6PD	8.9	10.6	3.291249	0.00034431	0.00562002
TC0600008571.hg.1	MYO6	6.8	8.8	4.090126	0.00034512	0.00562896
TC0600007485.hg.1	HLA-V	7.9	9.3	2.699626	0.00035053	0.00570423
TC0300009597.hg.1	PIK3CA	9.3	10.7	2.733719	0.00035437	0.00574928
TC0200015397.hg.1	CLK1	6.5	8.0	2.806311	0.00035485	0.00575268
TC1000012090.hg.1	IKZF5	9.4	10.9	2.770555	0.00035856	0.00579747
TC1900011726.hg.1	C19orf33	9.3	10.9	3.020107	0.00035922	0.00579747
TC0300013948.hg.1	TBC1D5	8.6	10.0	2.730423	0.00035971	0.00579747
TC0100012734.hg.1	TNFRSF9	5.6	7.8	4.346322	0.00036565	0.00587649
TC1100009981.hg.1	TPP1	11.2	12.7	2.780096	0.0003663	0.00588049
TC0100012097.hg.1	LGALS8	7.7	9.3	2.975764	0.00036728	0.00589186
TC1400008746.hg.1	TINF2	6.4	8.5	4.461688	0.00037125	0.00594225
TC0100018519.hg.1	F11R	7.9	9.4	2.8025	0.00037181	0.00594667
TC0800012396.hg.1	FBXO16	7.4	8.9	2.809533	0.00037539	0.00597712
TC0800009113.hg.1	PTP4A3	8.3	9.8	2.946679	0.00037566	0.00597712
TC1200010984.hg.1	CTDSP2	9.7	11.1	2.735158	0.000377	0.00599229
TC0900007085.hg.1	TESK1; MIR4667	9.5	11.1	3.163935	0.00037717	0.00599229
TC0900008684.hg.1	GPR21; RABGAP1	11.3	13.0	3.189167	0.00037786	0.00599875
TC1200010612.hg.1	RHEBL1	4.5	6.0	2.852991	0.00038124	0.00603363
TC1600011578.hg.1	FBXO31	10.8	12.3	2.85054	0.00038129	0.00603363
TC0300011000.hg.1	MAP4	7.4	8.9	2.810585	0.00038143	0.00603363
TC2200007982.hg.1	UFD1L	10.6	12.1	2.726481	0.00038207	0.00603875
TC0500011208.hg.1	WDR41	9.9	11.3	2.689979	0.00038325	0.00605293
TC0100018433.hg.1	RRAGC	10.3	11.9	2.967103	0.00038428	0.0060602
TC1900009670.hg.1	DOCK6	7.7	9.9	4.424533	0.00038455	0.0060602
TC0200011040.hg.1	ITM2C	12.5	14.0	2.69527	0.0003884	0.00608942
TC0200013283.hg.1	TMEM150A	7.6	9.5	3.571796	0.00038919	0.00609194

TC0100014457.hg.1	JAK1	10.0	11.5	2.824137	0.00039121	0.00611523
TC0400011144.hg.1	ANTXR2	13.2	14.6	2.695531	0.00039173	0.00611523
TC0100016366.hg.1	KIFAP3	7.0	8.4	2.764071	0.00039204	0.00611523
TC0200007115.hg.1	CLIP4	9.1	10.6	2.828477	0.00039279	0.00611996
TC0700009700.hg.1	NUB1	11.0	12.4	2.661547	0.00039291	0.00611996
TC0800007436.hg.1	IKBKB	3.8	5.7	3.714363	0.00039407	0.00613361
TC1900008240.hg.1	ZNF283	11.8	13.2	2.697084	0.0003975	0.00616444
TC0600008071.hg.1	PTK7	7.2	8.7	2.802132	0.00039753	0.00616444
TC0800012285.hg.1	HMBOX1	9.2	10.8	2.947394	0.00039755	0.00616444
TC1000009841.hg.1	PHYH	7.5	9.1	2.964205	0.00039778	0.00616444
TC0100009195.hg.1	SLC35A3	11.9	13.3	2.653855	0.00039866	0.00616919
TC0100015975.hg.1	RIT1	8.2	9.7	2.772389	0.00040102	0.0061968
TC0100014927.hg.1	EVI5	8.3	9.8	2.786743	0.00040264	0.00621725
TC0200010511.hg.1	NBEAL1	8.9	10.4	2.805251	0.00040425	0.00623768
TC2200007495.hg.1	SREBF2	9.7	11.2	2.943851	0.00040642	0.0062577
TC0900009588.hg.1	TTC39B	5.1	6.5	2.645875	0.00040902	0.00629321
TC0500007804.hg.1	HMGCR	14.7	16.2	2.747777	0.00041262	0.0063267
TC0200015559.hg.1	PLEKHM3	7.2	9.1	3.661793	0.00041275	0.0063267
TC1000007890.hg.1	HKDC1	7.1	9.0	3.872433	0.00041541	0.00635919
TC0800009331.hg.1	ERICH1	9.5	11.3	3.476498	0.00041598	0.00635919
TC0100013323.hg.1	STPG1	6.1	7.7	3.103261	0.00041912	0.00639349
TC0300013914.hg.1	TPRG1	5.5	7.1	3.072259	0.00042116	0.00641913
TC0100015634.hg.1	ACP6	6.6	8.2	2.851487	0.0004241	0.00644176
TC1200009160.hg.1	P2RX4	7.6	9.1	2.950308	0.00042511	0.00644817
TC1900011933.hg.1	ZNF585A	8.4	10.1	3.244026	0.00042736	0.00647769
TC0900012157.hg.1	AKAP2	6.2	7.7	2.730395	0.00042783	0.00648031
TC0900008945.hg.1	GPR107	11.3	12.7	2.709553	0.00042832	0.00648308
TC1600009512.hg.1	NTAN1	6.4	7.9	2.797194	0.00042904	0.0064895
TC1700008844.hg.1	TTYH2	8.4	9.8	2.617111	0.00042984	0.00649702
TC1200008771.hg.1	ISCU	8.6	10.1	2.817781	0.0004323	0.00652959
TC0X00007363.hg.1	MAGED2	6.3	8.2	3.876636	0.00043503	0.00655693
TC1400008036.hg.1	COX8C	5.2	6.8	2.892035	0.0004362	0.00655955
TC0700012836.hg.1	KIAA1147	9.3	10.7	2.644583	0.00043633	0.00655955
TC1100008627.hg.1	ANKRD42	7.4	9.2	3.497587	0.00043849	0.00657673
TC1000008904.hg.1	PDCD4; MIR4680	9.6	11.0	2.673482	0.00044246	0.00662137
TC0300013787.hg.1	TTLL3	7.2	8.8	3.230439	0.0004427	0.00662137
TC0900010607.hg.1	AGTPBP1	8.3	10.3	3.909787	0.00044435	0.00663743
TC1700009123.hg.1	RNF213	10.9	12.4	2.710868	0.00044684	0.00665541
TC0700006662.hg.1	C1GALT1	8.6	10.5	3.759499	0.00044819	0.00667087
TC1900011884.hg.1	ZNF443	7.2	8.7	2.772534	0.00045017	0.00669568
TC1500007097.hg.1	B2M	13.3	14.7	2.596998	0.00045296	0.00672508
TC1900010032.hg.1	CERS1; GDF1	6.9	8.3	2.653828	0.00045298	0.00672508
TC1000011585.hg.1	GOT1	11.3	12.7	2.645948	0.00045347	0.00672508
TC1800007193.hg.1	SLC14A2	3.9	5.4	2.746358	0.00045371	0.00672508
TC1200012761.hg.1	TAS2R31	9.2	10.6	2.621513	0.00045983	0.00679699
TC0100011096.hg.1	NEK7	8.5	10.1	2.983199	0.00046228	0.00682381
TC1900006932.hg.1	ZNF559-ZNF177; Z	8.6	10.1	2.771813	0.00046358	0.00683358
TC0X00009855.hg.1	ZXDA	5.5	7.0	2.810847	0.00047082	0.00691179
TC1400008583.hg.1	CCNB1IP1; SNORI	8.8	10.2	2.695765	0.00047316	0.00692929
TC1900006605.hg.1	ZNF57	9.5	10.9	2.669436	0.00047377	0.00692929

TC0800010138.hg.1	RAB11FIP1	7.1	8.7	2.999932	0.00047393	0.00692929
TC0100014392.hg.1	TM2D1	10.1	11.4	2.615434	0.00047409	0.00692929
TC0400012940.hg.1	SEC31A	12.5	13.9	2.625732	0.00047427	0.00692929
TC1200008739.hg.1	TMEM263	8.4	9.9	2.909527	0.00047512	0.00693108
TC0700009065.hg.1	CCDC136	6.8	8.3	2.770344	0.00047611	0.00693728
TC0100014910.hg.1	TGFBR3	10.1	11.5	2.65085	0.00047709	0.00693877
TC1500007513.hg.1	LACTB	6.6	8.1	2.700291	0.00047719	0.00693877
TC0400012900.hg.1	FBXL5	8.6	10.0	2.710831	0.00047865	0.00695539
TC0600014074.hg.1	HIVEP1	11.3	13.2	3.78216	0.00047957	0.00696397
TC1900008496.hg.1	PPP1R15A	6.8	8.8	3.85755	0.00048016	0.00696787
TC1900009692.hg.1	ZNF823	8.1	9.8	3.134217	0.00048134	0.00698022
TC1900006866.hg.1	SNAPC2	8.0	9.6	3.057347	0.00048184	0.00698277
TC0700006618.hg.1	WIPI2	11.3	12.7	2.596648	0.0004834	0.0069853
TC0200007591.hg.1	ERLEC1	8.7	10.2	2.775216	0.00048363	0.0069853
TC1000007700.hg.1	UBE2D1	9.2	10.7	2.718442	0.0004838	0.0069853
TC1800008655.hg.1	DYM	11.5	13.0	2.781359	0.00048397	0.0069853
TC0700013605.hg.1	PNPLA8	8.1	10.1	4.060296	0.00048496	0.00699494
TC1200007653.hg.1	NR4A1	5.4	7.2	3.615675	0.00048825	0.00702814
TC0600010066.hg.1	IGF2R	11.3	12.8	2.843169	0.00049	0.00704392
TC0X00010793.hg.1	ZDHHC9	11.6	13.0	2.614029	0.00049389	0.00707355
TC1000008452.hg.1	BTAF1	11.5	13.1	2.958344	0.00049413	0.00707355
TC1900009651.hg.1	YIPF2	7.2	9.1	3.726188	0.00049474	0.00707411
TC0100014214.hg.1	ECHDC2	9.5	11.1	3.079807	0.00049531	0.00707759
TC0900008960.hg.1	FUBP3	6.1	7.6	2.706597	0.00049734	0.00709711
TC1200010518.hg.1	SLC38A2	17.4	18.9	2.889309	0.00050002	0.00711644
TC1100007139.hg.1	METTL15	10.1	11.5	2.589071	0.00050041	0.00711719
TC2100006967.hg.1	IFNGR2	14.2	15.6	2.678555	0.0005033	0.0071536
TC0300012170.hg.1	GOLGB1	9.6	11.0	2.571135	0.00050523	0.0071715
TC1500010924.hg.1	AP3S2; MIR5009	10.5	11.9	2.682606	0.0005064	0.00718341
TC0200008870.hg.1	BCL2L11	9.4	11.0	3.00551	0.00050741	0.00718537
TC2000008666.hg.1	NAPB	8.6	10.0	2.729242	0.00050755	0.00718537
TC0800008531.hg.1	EMC2	9.1	10.5	2.61528	0.00050818	0.00718964
TC0500008296.hg.1	MAN2A1	9.1	10.7	2.981287	0.00051072	0.00721125
TC0600013193.hg.1	STX7	6.4	7.9	2.754365	0.00051123	0.00721368
TC0500013015.hg.1	PHYKPL	7.2	8.6	2.68388	0.00051216	0.00722203
TC0900011829.hg.1	RALGDS	8.2	9.6	2.779403	0.00051368	0.00723408
TC1200012745.hg.1	C1RL	8.5	10.2	3.139675	0.00051585	0.0072395
TC1700008228.hg.1	ITGA3	13.2	14.7	2.768962	0.00051631	0.0072395
TC1100007876.hg.1	TMEM179B; MIR67	12.6	14.1	2.734061	0.00051666	0.0072395
TC1900008012.hg.1	CATSPERG	5.3	6.8	2.957319	0.00051669	0.0072395
TC1100013131.hg.1	IGF2	4.6	6.4	3.505682	0.00051843	0.00725559
TC0100006861.hg.1	FBXO44	8.1	9.5	2.665618	0.00051859	0.00725559
TC0900008314.hg.1	FSD1L	9.0	10.4	2.737199	0.00051944	0.00726219
TC0400012143.hg.1	FBXW7	6.3	7.8	2.670065	0.00052004	0.00726219
TC0300009724.hg.1	VPS8	9.2	10.9	3.067334	0.00052058	0.00726219
TC1400007133.hg.1	ABHD12B	3.7	5.1	2.657335	0.00052088	0.00726219
TC0200008291.hg.1	KDM3A	6.0	7.5	2.963568	0.00052195	0.00726466
TC2100008306.hg.1	HSF2BP	4.5	5.9	2.564078	0.00052307	0.00727551
TC1700011795.hg.1	PRPSAP1	9.7	11.0	2.563057	0.00052435	0.00728166
TC0700013390.hg.1	TRIM73	5.6	6.9	2.576495	0.00052746	0.00731752

TC0600012432.hg.1	PGM3	9.4	10.9	2.910527	0.00052893	0.00733317
TC0100018248.hg.1	RNPC3	7.6	9.1	2.82977	0.00052968	0.00733891
TC0300013864.hg.1	PDIA5; MIR7110	7.7	9.1	2.626797	0.0005311	0.00735208
TC1600011551.hg.1	TK2	8.0	9.5	2.703719	0.0005316	0.00735208
TC0400008803.hg.1	RAB33B	7.8	9.2	2.682903	0.00053166	0.00735208
TC0X00008832.hg.1	GDI1	11.3	12.7	2.596485	0.00053435	0.00737972
TC1700007451.hg.1	CPD	8.7	10.1	2.573906	0.00053506	0.00738006
TC1000008984.hg.1	FAM160B1	7.5	8.9	2.579399	0.00053575	0.0073848
TC0700010189.hg.1	CYTH3	9.8	11.2	2.662229	0.00054066	0.0074286
TC1800007101.hg.1	KIAA1328	6.0	7.5	2.75385	0.00054154	0.00743121
TC1200011099.hg.1	GNS	13.9	15.3	2.607858	0.00054307	0.00743315
TC2000007817.hg.1	FAM210B	6.9	8.4	2.807196	0.00054583	0.0074662
TC0100015258.hg.1	DRAM2	9.8	11.2	2.628282	0.00054726	0.00748009
TC1600011442.hg.1	MAP1LC3B	11.0	12.6	3.003533	0.00054778	0.00748009
TC1500006994.hg.1	CHP1	13.9	15.3	2.526587	0.00054806	0.00748009
TC0200016421.hg.1	BRE	12.7	14.0	2.581035	0.00054859	0.00748009
TC2200009314.hg.1	RTN4R	6.9	9.0	4.444481	0.0005499	0.00748842
TC0300008561.hg.1	PARP14	8.5	10.3	3.489293	0.00055197	0.00750877
TC1500008988.hg.1	GOLGA8A; GOLGA	9.3	10.9	3.038807	0.00055552	0.00755062
TC0300013146.hg.1	TNFSF10	4.9	6.8	3.731654	0.00055997	0.00759611
TC0300011450.hg.1	TMF1	8.5	9.9	2.64528	0.00056106	0.00760172
TC0700011944.hg.1	ZNF394	9.0	10.4	2.805076	0.0005642	0.00762436
TC1000011938.hg.1	SHTN1	9.8	11.2	2.64005	0.0005645	0.00762436
TC0X00007535.hg.1	ARR3	3.8	5.2	2.600448	0.00056531	0.00763042
TC1100013061.hg.1	NADSYN1	9.0	10.5	2.662433	0.00057348	0.0077067
TC2000008493.hg.1	BFSP1	7.0	8.4	2.559469	0.0005739	0.00770754
TC0200015976.hg.1	SP110	6.7	8.2	2.706259	0.00057598	0.00772102
TC0500010282.hg.1	CDH12	4.8	6.2	2.765959	0.00057936	0.00774939
TC1900007957.hg.1	ZNF568	6.0	7.8	3.54662	0.00058004	0.00775124
TC0600007257.hg.1	TRIM38	7.6	9.1	2.906796	0.0005877	0.00783403
TC0700008264.hg.1	DBF4	5.5	6.9	2.523656	0.00058914	0.00784842
TC0X00008509.hg.1	ZNF449	7.0	8.5	2.777661	0.00059003	0.00785151
TC0600010779.hg.1	LINC00518	4.5	6.2	3.177672	0.00059183	0.00785389
TC1100013191.hg.1	POLD4	6.5	8.0	2.762261	0.00059462	0.00787692
TC0100014344.hg.1	MYSM1	13.4	14.8	2.610824	0.00059722	0.00789231
TC1000008400.hg.1	IFIT1	8.9	10.4	2.914605	0.00059954	0.00791798
TC1800006710.hg.1	CHMP1B	9.5	10.9	2.693076	0.0005999	0.00791798
TC0900011575.hg.1	RPL12	15.6	17.0	2.609792	0.00060241	0.0079413
TC1700008372.hg.1	SCPEP1	10.2	11.5	2.511249	0.00060402	0.00794936
TC0600011822.hg.1	MRPS10	11.3	12.9	3.057399	0.00060497	0.00794936
TC1500010251.hg.1	HOMER2	9.0	10.3	2.553126	0.000605	0.00794936
TC0400012944.hg.1	HSD17B13	4.1	5.7	3.139892	0.0006055	0.00794936
TC0200015870.hg.1	WDFY1	7.0	8.8	3.491652	0.00060562	0.00794936
TC0X00009650.hg.1	WDR45; PRAF2	7.0	9.0	4.052621	0.00060691	0.00795661
TC1900008699.hg.1	ZNF528	6.8	8.2	2.630186	0.00060823	0.00796904
TC0700011571.hg.1	YWHAG	10.5	12.0	2.745026	0.00061558	0.00802614
TC0800009486.hg.1	DEFB107A; DEFB1	4.5	5.8	2.512668	0.00061889	0.00806029
TC0800009094.hg.1	DENND3	6.0	7.5	2.721506	0.00061895	0.00806029
TC0500011490.hg.1	TTC37	9.7	11.1	2.522554	0.00061991	0.00806786
TC0100014653.hg.1	USP33	10.0	11.4	2.759219	0.00062093	0.00807619

TC1900011029.hg.1	MEIS3	5.7	7.2	2.88934	0.00062204	0.00808342
TC0100017118.hg.1	YOD1	6.9	8.8	3.743455	0.00062336	0.00809317
TC1700006721.hg.1	SLC16A13	8.8	10.3	2.781331	0.00062858	0.00815102
TC1600011315.hg.1	FAM234A; ARHGD	11.1	12.6	2.722251	0.00063021	0.00816724
TC1100013145.hg.1	COPB1	10.9	12.2	2.606909	0.00063366	0.00820198
TC0300013337.hg.1	MCF2L2	6.2	7.8	3.104315	0.00063589	0.0082209
TC1000008447.hg.1	TNKS2	11.1	12.8	3.205567	0.0006397	0.00826027
TC0600011483.hg.1	PBX2	9.7	11.1	2.490042	0.00064304	0.00829345
TC0200009270.hg.1	RAB6C	6.5	7.9	2.600403	0.00064421	0.00830308
TC0100014175.hg.1	EPS15	12.0	13.4	2.759697	0.00064456	0.00830308
TC0300006827.hg.1	UBE2E2	5.5	6.9	2.511292	0.00065158	0.00836826
TC1800006506.hg.1	MYL12A	10.9	12.3	2.561805	0.00065382	0.00838058
TC0900010366.hg.1	KLF9	6.7	9.4	6.249085	0.00065402	0.00838058
TC1100006516.hg.1	BRSK2	5.0	6.4	2.692423	0.0006541	0.00838058
TC1100008985.hg.1	ATM	13.7	15.1	2.51598	0.00065518	0.00838944
TC0500013336.hg.1	SSBP2	6.6	8.0	2.549308	0.00065562	0.00839002
TC0100015716.hg.1	MTMR11	6.6	8.0	2.67515	0.00065684	0.00840067
TC0100015803.hg.1	TDRKH	7.6	9.0	2.591819	0.00066009	0.00843211
TC0400011130.hg.1	PAQR3	9.1	10.4	2.448817	0.00066556	0.00847946
TC1200008942.hg.1	PLBD2	11.6	13.0	2.625841	0.00066623	0.00847946
TC1600011354.hg.1	NPIPA2	10.0	11.6	2.970046	0.00066635	0.00847946
TC0900008467.hg.1	KIAA1958	8.0	9.4	2.547248	0.00066843	0.00848813
TC0100018515.hg.1	CFAP45	3.3	4.6	2.454493	0.00067003	0.00849838
TC2100007321.hg.1	PDXK	12.0	13.4	2.671741	0.00067102	0.00850093
TC1100006580.hg.1	KCNQ1	5.5	6.9	2.539607	0.00067336	0.00851357
TC0100017471.hg.1	WDR26; MIR4742	11.1	12.5	2.591029	0.00067361	0.00851357
TC0700009333.hg.1	UBN2	10.1	11.6	2.736099	0.00067488	0.00851849
TC0100008126.hg.1	AKR1A1	6.9	8.3	2.710493	0.0006788	0.00855559
TC0100013692.hg.1	KIAA0319L	12.3	13.6	2.528112	0.00067893	0.00855559
TC0100017018.hg.1	ETNK2	4.0	5.9	3.774851	0.00067967	0.0085599
TC0600007306.hg.1	BTN3A3	7.2	8.8	3.067419	0.00068032	0.00856189
TC1900008543.hg.1	FCGRT	5.0	6.6	2.877019	0.00068091	0.00856189
TC0300009310.hg.1	MLF1	6.7	8.2	2.810721	0.00068142	0.00856189
TC1600010341.hg.1	AKTIP	8.1	9.5	2.588391	0.00068464	0.0085714
TC1600010028.hg.1	C16orf58	12.1	13.3	2.456415	0.00068527	0.0085714
TC0200006757.hg.1	TRIB2	9.4	11.0	2.917949	0.00068532	0.0085714
TC0200015080.hg.1	PRKRA	9.3	10.8	2.80425	0.00068555	0.0085714
TC0200015364.hg.1	SATB2	9.4	10.9	2.678536	0.00068652	0.0085714
TC1700009081.hg.1	ENGASE	8.1	9.6	2.702698	0.00068671	0.0085714
TC0200009049.hg.1	INSIG2	7.0	8.6	2.95047	0.00068737	0.0085714
TC2100008030.hg.1	RCAN1	13.8	15.1	2.432635	0.00069125	0.00860109
TC1200008667.hg.1	HSP90B1	7.9	9.5	3.02607	0.00069243	0.0086023
TC0100014766.hg.1	LPAR3	3.8	5.2	2.526649	0.00069306	0.0086023
TC0200007458.hg.1	EPAS1	3.7	5.1	2.575415	0.00069532	0.00862537
TC0100018171.hg.1	PRAMEF25; PRAM	3.5	4.8	2.4819	0.00069975	0.00866522
TC1100007257.hg.1	CAT	6.5	7.8	2.461487	0.00070052	0.00866977
TC0800008786.hg.1	NSMCE2	10.2	11.5	2.515405	0.00070564	0.00871562
TC0200016654.hg.1	COX7A2L	11.9	13.2	2.449004	0.00070572	0.00871562
TC1000007103.hg.1	RAB18	10.2	11.5	2.43326	0.00070592	0.00871562
TC2100008165.hg.1	BRWD1	11.3	12.6	2.469638	0.00070691	0.00871562

TC1500008341.hg.1	MAN2A2	8.5	10.1	2.912383	0.00070707	0.00871562
TC1200012593.hg.1	CLEC4A	4.1	5.5	2.569531	0.00071193	0.00874996
TC1700010686.hg.1	JUP	7.3	8.6	2.448673	0.00071207	0.00874996
TC0200012488.hg.1	LOC100506142; RI	3.6	5.1	2.684196	0.00071377	0.00876304
TC0600012413.hg.1	IBTK	11.7	13.0	2.497354	0.00071856	0.0088017
TC0300010931.hg.1	LZTFL1	5.6	7.4	3.555235	0.00072829	0.00890509
TC0400006990.hg.1	MED28	9.9	11.3	2.751837	0.00072898	0.00890509
TC1600011550.hg.1	BEAN1-AS1	4.5	5.9	2.563811	0.00073008	0.00891226
TC0800008184.hg.1	DECR1	7.6	8.9	2.447221	0.00073242	0.00893571
TC1900011766.hg.1	MARK4	9.4	10.8	2.615206	0.00073544	0.00896747
TC1500010698.hg.1	SNRPN; SNURF; S	9.5	11.2	3.155481	0.00073753	0.00898268
TC1600011355.hg.1	NPIPA1	10.5	12.1	3.055777	0.00073862	0.00899085
TC1900010708.hg.1	BLVRB	9.0	10.6	2.840668	0.00074209	0.00900427
TC2100007803.hg.1	APP	16.7	18.1	2.614211	0.00074291	0.00900427
TC0500011881.hg.1	CEP120	8.7	10.0	2.410496	0.00074346	0.00900427
TC0700013223.hg.1	LMBR1	5.4	7.1	3.415745	0.00074352	0.00900427
TC0600008348.hg.1	ZNF451	12.3	13.7	2.539149	0.00074392	0.00900427
TC0100010284.hg.1	PEA15	12.5	13.8	2.57256	0.00074837	0.00904835
TC1100007884.hg.1	SLC3A2	13.2	14.5	2.41651	0.00074847	0.00904835
TC1200012835.hg.1	NT5DC3	10.4	11.8	2.603864	0.00074883	0.00904835
TC0600009622.hg.1	CCDC28A	7.7	9.2	2.817674	0.00075015	0.00905922
TC1100008127.hg.1	SSH3	8.3	9.7	2.519408	0.00075132	0.00906826
TC0200008803.hg.1	GCC2	8.6	10.4	3.523637	0.00075437	0.0090949
TC1900010856.hg.1	PLAUR	9.0	10.3	2.503263	0.00075821	0.00913093
TC2000007509.hg.1	CTSA	13.6	14.9	2.418588	0.0007591	0.00913643
TC2000009898.hg.1	RP1-122P22.2; RIN	5.8	7.2	2.627417	0.00076389	0.00917356
TC0300006719.hg.1	BTD	8.6	10.0	2.623504	0.00076946	0.0092265
TC1400009912.hg.1	GALC	7.8	9.1	2.405972	0.00077262	0.00925097
TC0100016406.hg.1	VAMP4	8.2	9.5	2.409869	0.00077947	0.00929855
TC1900011944.hg.1	FBXO17	8.8	10.2	2.544479	0.0007795	0.00929855
TC0600012971.hg.1	ZUFSP	8.0	9.4	2.634412	0.00078886	0.00938408
TC1400009629.hg.1	ZFYVE1	5.6	7.4	3.447654	0.00079895	0.00948258
TC0100014191.hg.1	RAB3B	5.2	6.8	2.87692	0.00079935	0.00948258
TC0900006655.hg.1	LURAP1L	10.3	12.0	3.159398	0.00080178	0.00950088
TC1400007710.hg.1	JDP2	4.3	5.9	3.0322	0.00080317	0.00951213
TC0500012238.hg.1	HBEGF	5.5	6.8	2.40523	0.00080375	0.00951368
TC1700008452.hg.1	GDPD1	7.3	9.3	3.930859	0.00080531	0.00952309
TC0100011400.hg.1	PFKFB2	6.7	8.1	2.574594	0.00080543	0.00952309
TC1900006819.hg.1	TRIP10	12.9	14.2	2.420004	0.00080982	0.00955913
TC0100007638.hg.1	SERINC2	7.2	8.4	2.380311	0.00081551	0.00959987
TC1400007354.hg.1	PPM1A	8.7	10.3	2.86113	0.00081631	0.00960402
TC1800008824.hg.1	LMAN1	15.7	17.0	2.48135	0.00081897	0.00963007
TC1000008234.hg.1	FAM213A	8.3	9.9	3.137988	0.0008277	0.0097031
TC0Y00006882.hg.1	SLC25A6	15.5	16.8	2.411006	0.000829	0.00971071
TC1900009963.hg.1	ANO8	8.4	10.0	3.069758	0.00083525	0.00977859
TC1200011699.hg.1	ARL1	9.1	10.4	2.483052	0.00083644	0.00978214
TC0300013334.hg.1	MCCC1	10.4	11.7	2.579854	0.00085304	0.00993807
TC1600008199.hg.1	ZFP90	10.1	11.3	2.417465	0.00085402	0.00994415
TC0800008352.hg.1	VPS13B	12.7	14.0	2.451867	0.0008567	0.00996641
TC1200011719.hg.1	CCDC53	7.8	9.3	2.823159	0.00085687	0.00996641

TC1900010531.hg.1	ZNF461	8.6	10.0	2.661186	0.0008575	0.0099684
TC1000011445.hg.1	MYOF	14.8	16.2	2.547047	0.00085908	0.00997048
TC1400009780.hg.1	NOXRED1	4.3	5.5	2.428887	0.00086252	0.01000499
TC1200008002.hg.1	XPOT	13.5	14.8	2.362959	0.00086561	0.01001609
TC1500010630.hg.1	ADAMTS17	3.5	5.1	3.04239	0.00086617	0.01001609
TC1900009397.hg.1	LONP1	6.7	8.2	2.930191	0.0008671	0.01001609
TC0700006906.hg.1	KLHL7	8.6	9.9	2.406914	0.00087051	0.01003151
TC0500010549.hg.1	RICTOR	8.7	10.2	2.887569	0.00087088	0.01003151
TC0100010111.hg.1	EFNA1	5.0	7.3	4.792388	0.00087182	0.01003151
TC1900007399.hg.1	TMEM59L	5.4	6.8	2.799861	0.0008825	0.01013264
TC0400008429.hg.1	ALPK1	5.9	7.6	3.353936	0.00088489	0.01014743
TC0200010416.hg.1	SPATS2L	9.2	10.6	2.789816	0.00088554	0.01014743
TC1500007802.hg.1	GOLGA6B	5.5	6.8	2.436939	0.00088568	0.01014743
TC1400010605.hg.1	CTAGE5	7.6	8.9	2.445738	0.00088745	0.01016227
TC0200009078.hg.1	EPB41L5	10.0	11.4	2.601367	0.00088903	0.01016961
TC1400006715.hg.1	PCK2	6.5	8.0	2.868407	0.0008912	0.01018892
TC1300009956.hg.1	LACC1	4.0	5.3	2.514175	0.00089326	0.01020162
TC0400012366.hg.1	DDX60	5.1	7.1	4.119731	0.00089411	0.01020316
TC1200006445.hg.1	B4GALNT3	4.9	6.7	3.334949	0.00089503	0.01020316
TC0500010662.hg.1	HCN1	4.4	5.7	2.435039	0.0008956	0.01020316
TC1900011777.hg.1	CYTH2	10.3	11.6	2.349184	0.00089576	0.01020316
TC1900010511.hg.1	POLR2I	7.7	8.9	2.399361	0.00089651	0.01020617
TC0X00010097.hg.1	RLIM	11.7	13.0	2.425421	0.00090503	0.01028097
TC0200010837.hg.1	ANKZF1	8.6	9.9	2.381177	0.00090743	0.01028133
TC0200013019.hg.1	MCEE	4.4	6.0	3.031581	0.00090849	0.01028252
TC1900010781.hg.1	RABAC1	9.5	11.0	2.877447	0.00091063	0.01029584
TC1600011517.hg.1	DOC2A	6.1	7.3	2.399153	0.00091177	0.01030331
TC1500007804.hg.1	BBS4	6.3	8.2	3.630769	0.00091254	0.0103065
TC1000010498.hg.1	8-Mar	4.8	6.1	2.565296	0.00091322	0.01030882
TC0800012351.hg.1	WDYHV1	8.1	9.3	2.412827	0.00091465	0.01031876
TC0800007027.hg.1	SLC25A37	9.8	11.2	2.778151	0.00091553	0.01031876
TC0500013354.hg.1	P4HA2	9.2	10.5	2.490828	0.0009193	0.01035022
TC0100011552.hg.1	VASH2	3.8	5.4	2.973857	0.00092562	0.01040495
TC1800008231.hg.1	ESCO1	8.7	10.3	2.989678	0.00092733	0.01040845
TC0600014257.hg.1	HLA-C	6.5	9.4	7.257413	0.00092878	0.01040845
TC0300008536.hg.1	EAF2	5.5	7.4	3.561192	0.00092914	0.01040845
TC0900009470.hg.1	ERMP1	14.5	15.7	2.339329	0.00092933	0.01040845
TC0100013182.hg.1	CAMK2N1	7.0	8.5	2.863768	0.00093369	0.01043591
TC0300008142.hg.1	TBC1D23	10.8	12.1	2.447833	0.00093506	0.01044338
TC1800009284.hg.1	C18orf32	8.3	9.8	2.941893	0.00093566	0.01044338
TC0900012154.hg.1	TMEFF1	7.0	8.7	3.2666	0.00094034	0.0104826
TC0700007065.hg.1	PLEKHA8	9.5	10.8	2.364925	0.00094142	0.01048912
TC0X00011413.hg.1	L1CAM	5.3	7.0	3.277714	0.00094736	0.01053888
TC0700009083.hg.1	AHCYL2	8.2	9.5	2.472499	0.00095036	0.01056127
TC1200007764.hg.1	ZNF385A	6.1	7.4	2.573114	0.00095159	0.01056954
TC1000009140.hg.1	TACC2	7.3	9.1	3.699037	0.00095381	0.01058868
TC1200009747.hg.1	ING4	5.7	8.0	4.76916	0.0009565	0.01061303
TC0100015990.hg.1	PAQR6	6.1	7.6	2.729487	0.00096013	0.0106478
TC0100018499.hg.1	MUC1	6.8	8.4	3.046051	0.00096231	0.01066494
TC1000012270.hg.1	EBF3	4.5	5.7	2.344776	0.00096317	0.01066494

TC0100011554.hg.1	RPS6KC1	7.6	9.0	2.591396	0.00096558	0.01068064
TC2200007614.hg.1	FAM118A	8.5	9.8	2.475509	0.00096714	0.01068691
TC0400010772.hg.1	SRP72	4.4	6.6	4.647741	0.00097257	0.01073027
TC0100008912.hg.1	CYR61	9.1	10.4	2.356491	0.00097874	0.01078726
TC0700006529.hg.1	ELFN1	6.7	7.9	2.333855	0.00098042	0.01079467
TC1000010844.hg.1	RUFY2	11.7	13.0	2.473784	0.0009822	0.01080873
TC1500009669.hg.1	VPS13C	8.1	9.4	2.464355	0.0009831	0.01081228
TC1700008603.hg.1	CEP95	8.9	10.1	2.424109	0.0009843	0.01081523
TC1200008803.hg.1	MVK	8.2	9.4	2.331712	0.00098822	0.01083715
TC0100017365.hg.1	EPRS	11.9	13.1	2.3478	0.00098915	0.01083715
TC0600007887.hg.1	ZFAND3	11.4	12.6	2.430983	0.00098933	0.01083715
TC1100008523.hg.1	CAPN5	4.8	6.2	2.563811	0.00099461	0.01088941
TC1600011364.hg.1	NPIPB5	14.4	15.7	2.459373	0.00099893	0.0109312
TC0900009018.hg.1	NTNG2	5.4	6.7	2.392071	0.00099963	0.01093319
TC0500008784.hg.1	REEP2	8.7	10.5	3.583961	0.00100369	0.01097204
TC1100013130.hg.1	IGF2; INS-IGF2	4.0	5.5	2.719639	0.00100744	0.01099626
TC0600013300.hg.1	IFNGR1	7.7	8.9	2.392626	0.0010086	0.01100324
TC1500010736.hg.1	BLOC1S6	10.3	11.5	2.378167	0.00101087	0.01102248
TC1400010012.hg.1	LGMN	12.4	13.7	2.43867	0.00102038	0.01111483
TC1400010748.hg.1	LINC01588	5.0	6.3	2.494836	0.00102096	0.01111554
TC1100010551.hg.1	TRAF6	7.1	8.3	2.333993	0.00102549	0.01115917
TC1600007887.hg.1	RBL2	11.0	12.3	2.517724	0.00103092	0.01118353
TC1900011452.hg.1	PTPRH	6.9	8.2	2.353071	0.00103118	0.01118353
TC0100006483.hg.1	ISG15	4.4	5.8	2.599322	0.00103138	0.01118353
TC1700009256.hg.1	NARF	9.4	10.7	2.328143	0.00103303	0.01119572
TC1200011423.hg.1	TSPAN19	3.1	4.4	2.318907	0.00104451	0.01128886
TC0700013363.hg.1	GBAS	11.1	12.4	2.393455	0.00104478	0.01128886
TC0100014848.hg.1	CCBL2; RBMXL1	8.2	9.4	2.35556	0.00105257	0.01134448
TC0200009806.hg.1	TANK	8.3	9.6	2.521347	0.00105449	0.01135943
TC1300008229.hg.1	ZMYM5	8.9	10.2	2.45315	0.00105571	0.01136693
TC1600011047.hg.1	HSDL1	9.4	10.7	2.382902	0.00106269	0.01142487
TC0100010926.hg.1	OCLM	9.6	11.3	3.16738	0.00106353	0.01142818
TC0200016475.hg.1	INO80B	6.7	8.1	2.611937	0.00107583	0.01153141
TC0700012998.hg.1	ZNF767P	9.3	10.7	2.639656	0.00107714	0.01153966
TC0X00007655.hg.1	SLC16A2	9.6	11.0	2.564478	0.00107966	0.01155545
TC0600007748.hg.1	HMGA1	14.6	15.8	2.329298	0.00108073	0.01156083
TC1300009898.hg.1	GRTP1	5.4	6.9	2.759028	0.00108468	0.01158949
TC2200008055.hg.1	PI4KA	9.2	10.5	2.442637	0.00109067	0.0116439
TC0200013265.hg.1	CAPG	8.9	10.3	2.70356	0.0010925	0.01164755
TC0100014173.hg.1	TTC39A	4.3	5.8	2.710108	0.00109644	0.01167644
TC0100010192.hg.1	RRNAD1	7.6	8.8	2.423169	0.00110303	0.01174079
TC0100007562.hg.1	TRNAU1AP	6.4	8.2	3.36347	0.00110389	0.01174421
TC1700011128.hg.1	SPAG9	13.8	15.1	2.467636	0.0011068	0.01176927
TC0300009843.hg.1	TP63	12.3	13.5	2.287719	0.0011101	0.01179853
TC0300014050.hg.1	NPHP3	8.7	10.1	2.678554	0.00111265	0.01180809
TC1700009677.hg.1	BORCS6	4.0	5.2	2.291091	0.00111409	0.0118117
TC0500011448.hg.1	ARRDC3	7.9	9.5	3.028272	0.00111776	0.01183892
TC1500010788.hg.1	CHD2; MIR3175	9.1	10.3	2.277451	0.00112126	0.01185835
TC0300013854.hg.1	NXPE3	3.4	4.6	2.28703	0.00112245	0.01186509
TC1700007858.hg.1	FKBP10	14.0	15.2	2.364672	0.00112415	0.01187725

TC1400007899.hg.1	SPATA7	6.7	7.9	2.294135	0.00112818	0.01191391
TC1900008989.hg.1	ZNF460	11.8	13.2	2.673723	0.00114213	0.01201987
TC1900011233.hg.1	KLK10	4.2	5.4	2.378785	0.00114763	0.01206531
TC0600009393.hg.1	RNF146	10.9	12.2	2.463219	0.00114826	0.01206531
TC0900006539.hg.1	JAK2	7.4	9.2	3.415602	0.00115647	0.01210248
TC1300006481.hg.1	ZMYM2	7.2	8.4	2.331737	0.00116595	0.012169
TC0200008900.hg.1	TMEM87B	10.0	11.3	2.461376	0.00116876	0.01218645
TC2200009351.hg.1	CSNK1E	11.2	12.4	2.272003	0.00117394	0.01223455
TC1000012561.hg.1	JMJD1C	10.9	12.1	2.305844	0.00117813	0.01225113
TC0500008229.hg.1	PAM	7.2	8.5	2.500913	0.00117904	0.01225113
TC1200006888.hg.1	CDKN1B	9.1	10.6	2.719949	0.00120074	0.01241728
TC0300013703.hg.1	RNF168	10.1	11.3	2.290956	0.00120344	0.01243318
TC0200011980.hg.1	ITSN2	11.0	12.4	2.710163	0.00120511	0.01244452
TC1400007628.hg.1	PSEN1	11.2	12.4	2.293507	0.00120944	0.01248317
TC0200009402.hg.1	MGAT5	11.0	12.2	2.292918	0.00121118	0.01249511
TC0600007302.hg.1	BTN2A2	9.8	11.2	2.65186	0.00121332	0.01250474
TC0800012405.hg.1	FUT10	11.7	12.9	2.254605	0.00121384	0.01250474
TC1200012711.hg.1	LINC00173	5.0	6.8	3.396349	0.00121865	0.012531
TC0200015377.hg.1	TYW5	7.9	9.1	2.282081	0.00121875	0.012531
TC0500013089.hg.1	GFPT2	4.7	6.3	2.927472	0.0012223	0.01254344
TC1100008045.hg.1	TSGA10IP	5.0	6.4	2.674928	0.00122978	0.01260214
TC0600014366.hg.1	PDE10A	11.7	12.8	2.244805	0.00123875	0.01266988
TC1600008580.hg.1	GAN; MIR4720	10.0	11.7	3.357424	0.0012396	0.01267253
TC0700007472.hg.1	C7orf69	9.2	10.5	2.332925	0.00124277	0.01269887
TC1900008286.hg.1	BCAM	6.3	7.9	3.126406	0.00124813	0.01274754
TC0200007636.hg.1	CFAP36	7.1	8.7	2.898056	0.00125023	0.01276296
TC1300008819.hg.1	TPT1; SNORA31	12.5	13.8	2.528085	0.00125201	0.01277497
TC0200012022.hg.1	KIF3C	7.2	8.4	2.263712	0.00125751	0.01280074
TC0800010427.hg.1	RB1CC1	7.9	9.3	2.577514	0.00125992	0.01281656
TC0600007410.hg.1	ZSCAN9	8.1	9.6	2.845466	0.00126086	0.01281656
TC1900011794.hg.1	ZNF701; ZNF137P	7.1	8.5	2.726084	0.00126153	0.01281724
TC1600011353.hg.1	NPIPA3	10.1	11.6	2.872745	0.00126565	0.01285304
TC0800011206.hg.1	RPL30	14.6	15.9	2.423733	0.00126988	0.01287785
TC0100013925.hg.1	MED8	7.7	9.0	2.440844	0.0012743	0.0129042
TC0100013647.hg.1	TRIM62	4.4	5.6	2.287109	0.00127654	0.01290867
TC1600006822.hg.1	METTL22	7.9	9.3	2.736809	0.00129763	0.01307877
TC0X00011317.hg.1	TCEAL1	6.5	8.2	3.29779	0.00130358	0.01312024
TC0700010716.hg.1	DPY19L1	7.8	9.1	2.614119	0.00130837	0.0131622
TC1900011004.hg.1	SLC1A5	12.2	13.4	2.378513	0.00131094	0.01318196
TC1300009348.hg.1	SPRY2	9.9	11.1	2.323267	0.00131221	0.0131885
TC0200010255.hg.1	PMS1	9.3	10.5	2.241361	0.00131496	0.01320376
TC0300012484.hg.1	SLCO2A1	3.3	4.7	2.471248	0.00131621	0.01320393
TC1300008114.hg.1	CUL4A	8.3	9.5	2.246338	0.00132059	0.01324171
TC1900011868.hg.1	EPOR	4.7	6.1	2.702389	0.00132339	0.01325124
TC0300013103.hg.1	RPL22L1	10.0	11.2	2.39607	0.00132443	0.01325241
TC1200012610.hg.1	SSPN	9.3	10.6	2.46697	0.00133124	0.01329967
TC1000008431.hg.1	PCGF5	4.4	5.7	2.34619	0.00133185	0.01329967
TC0100011421.hg.1	CD46	6.8	7.9	2.227545	0.00133195	0.01329967
TC1600011487.hg.1	NPIPA8	10.2	11.7	2.912635	0.0013393	0.01335441
TC2200009350.hg.1	CSNK1E	12.5	13.6	2.228712	0.00135584	0.0134788

TC1800008781.hg.1	ATP8B1	7.0	8.7	3.333217	0.00135749	0.0134788
TC0100017058.hg.1	TMEM81	4.6	5.7	2.217555	0.00136261	0.01350517
TC1000009149.hg.1	PLEKHA1	7.3	8.6	2.49264	0.00136634	0.01353592
TC0500013369.hg.1	SIL1	10.7	12.1	2.589351	0.00136875	0.01355353
TC1900006524.hg.1	CIRBP	12.5	13.6	2.231169	0.00137098	0.01356933
TC2200007904.hg.1	ATP6V1E1	10.3	11.6	2.387482	0.00137514	0.01358782
TC1500010906.hg.1	RP11-351M8.2; ME	4.1	5.5	2.558628	0.00137566	0.01358782
TC0300009728.hg.1	VPS8	3.2	4.8	3.037596	0.00138086	0.01362942
TC1100006484.hg.1	EPS8L2	8.3	9.5	2.270681	0.00139131	0.01371356
TC1900007917.hg.1	ZNF146	12.5	13.7	2.215527	0.00139298	0.01372379
TC0X00008908.hg.1	SLC25A6	15.6	16.8	2.298998	0.00139868	0.0137673
TC0100015771.hg.1	SEMA6C	5.0	6.4	2.720506	0.0014012	0.01378577
TC0400012452.hg.1	FBXO8	6.9	8.2	2.482786	0.00140738	0.01382385
TC1500007038.hg.1	STARD9	7.7	9.2	2.763382	0.0014158	0.01389747
TC1900011750.hg.1	ZNF155	5.6	6.7	2.208207	0.00141947	0.01392719
TC1300007845.hg.1	CLYBL	4.9	6.5	2.960046	0.00142281	0.01395354
TC1100011058.hg.1	FTH1	17.2	18.3	2.208935	0.00142535	0.01395884
TC1500010613.hg.1	TTC23	8.5	9.7	2.267991	0.00143197	0.01400747
TC1700007504.hg.1	RHOT1	9.7	10.8	2.236038	0.00143223	0.01400747
TC0500007552.hg.1	LOC100421561	7.1	8.4	2.37312	0.00144104	0.01408722
TC2000008218.hg.1	C20orf194	10.4	11.5	2.202574	0.00144289	0.01409892
TC0700011497.hg.1	MLXIPL	7.4	9.1	3.30422	0.00145825	0.01421013
TC2200009266.hg.1	GTPBP1	8.1	9.2	2.289124	0.00146159	0.01423625
TC0300010561.hg.1	SLC4A7	14.0	15.1	2.189831	0.00147792	0.01436263
TC0900009333.hg.1	FAM157B	9.2	10.4	2.210626	0.0014814	0.01437042
TC0200008536.hg.1	ANKRD36	10.7	11.9	2.273736	0.00148224	0.01437209
TC0700013382.hg.1	KCTD7	8.1	9.4	2.311092	0.00148326	0.01437552
TC0900007050.hg.1	DNAI1	6.6	7.9	2.390429	0.00149022	0.01441687
TC1600011378.hg.1	MVP; PAGR1	8.5	9.8	2.479828	0.0015002	0.01450032
TC0X00010643.hg.1	6-Sep	9.3	10.6	2.348623	0.00150348	0.0145255
TC0800010506.hg.1	PLAG1	3.3	4.9	2.985391	0.00150492	0.01453291
TC0X00008241.hg.1	PGRMC1	7.9	9.2	2.445586	0.00150872	0.01455642
TC1500009336.hg.1	MYEF2	5.2	6.5	2.570012	0.00151057	0.01456779
TC1500008023.hg.1	ZFAND6	8.2	9.4	2.312495	0.00151259	0.01457345
TC1700012242.hg.1	LOC101060389; TE	8.0	9.1	2.196421	0.00151456	0.01457345
TC0400010519.hg.1	APBB2	6.2	7.6	2.59528	0.00151944	0.01461387
TC1900008507.hg.1	FTL	14.4	15.7	2.480817	0.00152063	0.01461873
TC1000008088.hg.1	SAMD8	9.7	11.0	2.477629	0.00152437	0.01464342
TC1600007007.hg.1	PDXDC1	8.1	9.3	2.297565	0.00152654	0.01465586
TC0700013343.hg.1	ZNRF2	7.9	9.2	2.458238	0.00152933	0.01466948
TC0700006727.hg.1	TMEM106B	8.4	9.6	2.289901	0.0015445	0.01476221
TC0100013611.hg.1	BSDC1	8.3	9.5	2.303775	0.00156169	0.0148981
TC0600007585.hg.1	MICB	7.7	9.0	2.483922	0.00156179	0.0148981
TC0400008450.hg.1	ANK2	5.9	7.1	2.34359	0.00156357	0.01490461
TC1600011386.hg.1	ORAI3	7.1	8.3	2.271374	0.00156502	0.01490519
TC2000009819.hg.1	HELZ2	3.9	5.1	2.340911	0.00156613	0.01490919
TC1200006881.hg.1	CREBL2	7.8	9.0	2.343971	0.00157161	0.01494139
TC0600007303.hg.1	BTN3A1	7.5	8.9	2.640854	0.00157391	0.01495666
TC1500009429.hg.1	DMXL2	8.5	9.7	2.241004	0.00158729	0.01507021
TC1700010088.hg.1	NATD1	3.8	5.1	2.35967	0.00158807	0.01507021

TC0300013932.hg.1	SRGAP3	5.4	6.5	2.175663	0.00158867	0.01507021
TC1700011629.hg.1	SLC39A11	8.0	9.2	2.234226	0.00159514	0.01511155
TC0200011421.hg.1	ING5	7.6	8.8	2.227608	0.00160418	0.01517791
TC0300013970.hg.1	PFKFB4; MIR6823	5.0	6.8	3.370973	0.00160533	0.01517791
TC1200011574.hg.1	FGD6	8.6	9.8	2.292355	0.00160568	0.01517791
TC0500008086.hg.1	NR2F1	11.1	12.4	2.438679	0.00161017	0.01520694
TC1900010037.hg.1	SUGP2	10.5	11.6	2.220346	0.00161632	0.0152516
TC0700012296.hg.1	C7orf60	5.2	6.4	2.252278	0.00162436	0.0152982
TC1900007384.hg.1	GDF15	3.8	5.2	2.498297	0.00162546	0.0152982
TC0100015023.hg.1	DPYD	15.0	16.1	2.184889	0.00162593	0.0152982
TC0500013064.hg.1	MGAT4B	9.1	10.3	2.263695	0.00163091	0.01530846
TC0400011057.hg.1	SCARB2	7.9	9.5	3.018057	0.00163431	0.01532082
TC0500008849.hg.1	CYSTM1	8.7	10.2	2.819746	0.00163498	0.01532082
TC0500010930.hg.1	TRIM23	8.9	10.0	2.251513	0.0016358	0.01532082
TC1100012375.hg.1	C11orf71	6.5	7.7	2.257092	0.00163704	0.01532576
TC0X00009606.hg.1	SSX3	2.7	4.2	2.731086	0.0016386	0.01533304
TC0400010768.hg.1	AASDH	7.1	8.3	2.167159	0.00163925	0.01533304
TC0300007482.hg.1	NISCH	9.1	10.3	2.282398	0.0016421	0.01535297
TC0600007711.hg.1	ITPR3	10.7	11.8	2.187336	0.00164514	0.01537398
TC1700010515.hg.1	TBC1D3	6.5	7.6	2.159624	0.00164578	0.01537398
TC1400010019.hg.1	MOAP1	5.3	6.6	2.378381	0.001648	0.01538137
TC0100013000.hg.1	CASP9	5.2	6.7	2.824548	0.00165249	0.01540149
TC0100018222.hg.1	LOC100129924; C1	5.9	7.4	2.864076	0.00165299	0.01540149
TC0800009997.hg.1	KIF13B	8.5	9.7	2.287553	0.00166499	0.01547413
TC1300006930.hg.1	COG6	8.4	9.8	2.680783	0.00166612	0.01547633
TC1000010026.hg.1	PIP4K2A	4.8	6.1	2.326401	0.00167134	0.01550162
TC0100018339.hg.1	MDM4	11.7	12.9	2.343485	0.00167173	0.01550162
TC0300008912.hg.1	PCCB	8.5	9.6	2.174344	0.00168912	0.01564261
TC2100007597.hg.1	HSPA13	11.2	12.4	2.180336	0.00169149	0.01565777
TC0400010529.hg.1	APBB2	5.7	6.8	2.269068	0.00169464	0.01567696
TC2100007056.hg.1	SIM2	8.2	9.4	2.223951	0.00169502	0.01567696
TC1900012000.hg.1	TEAD2	9.9	11.0	2.177172	0.00170612	0.01575921
TC1100011257.hg.1	EFEMP2	5.0	6.3	2.460984	0.00170828	0.01576007
TC0100010389.hg.1	HSD17B7	11.9	13.2	2.41816	0.00171116	0.01576007
TC1100010236.hg.1	KCNJ11	5.5	6.7	2.349103	0.00172023	0.01582143
TC1500009082.hg.1	BMF	5.0	6.3	2.49596	0.00173146	0.01589737
TC0100015561.hg.1	NBPF15	11.9	13.0	2.17143	0.0017325	0.01589737
TC0800011285.hg.1	ZNF706	9.1	10.6	2.849689	0.00173294	0.01589737
TC0500008157.hg.1	LNPEP	12.1	13.3	2.17958	0.00173805	0.01593744
TC0400011220.hg.1	WDFY3	6.7	7.9	2.291091	0.0017393	0.0159421
TC1000010870.hg.1	SRGN	13.5	14.6	2.239334	0.00174118	0.01594792
TC1700011476.hg.1	GNA13	10.9	12.0	2.144238	0.00174245	0.01594792
TC0100010863.hg.1	LAMC1	11.9	13.0	2.214207	0.00174484	0.01595875
TC1000009065.hg.1	FAM45A	5.3	7.1	3.501602	0.00174956	0.01598146
TC1500008521.hg.1	MEF2A	10.0	11.3	2.359359	0.00176506	0.01609568
TC1300009492.hg.1	TGDS	9.1	10.3	2.399552	0.00176638	0.01610089
TC0900008865.hg.1	CERCAM	8.4	9.6	2.279804	0.00176713	0.01610089
TC0300012665.hg.1	XRN1	9.9	11.0	2.237674	0.00176954	0.01610911
TC0300008018.hg.1	C3orf38	7.8	9.2	2.769192	0.00177072	0.01611305
TC0900010769.hg.1	NFIL3	7.2	8.3	2.155205	0.00177419	0.0161309

TC0500010085.hg.1	CMBL	10.4	11.5	2.205323	0.00177666	0.0161425
TC0100009331.hg.1	STXBP3	10.3	11.5	2.355209	0.00178151	0.01617002
TC1200008677.hg.1	RPL18A	12.9	14.0	2.256247	0.00178283	0.0161752
TC0100010623.hg.1	SUCO	5.8	7.0	2.30005	0.00178582	0.01619546
TC0200007473.hg.1	CRIPT	9.9	11.0	2.254324	0.00178914	0.0162076
TC1900009854.hg.1	NOTCH3	4.5	5.9	2.59412	0.00179406	0.01622984
TC1600009165.hg.1	MMP25-AS1	3.5	4.6	2.17405	0.00179415	0.01622984
TC1200007535.hg.1	CACNB3	6.2	7.4	2.392676	0.00179563	0.0162364
TC1900011106.hg.1	NUCB1	5.8	7.0	2.181432	0.00179641	0.01623661
TC1400006890.hg.1	SRP54	8.6	10.3	3.299987	0.00179873	0.01625069
TC1200008678.hg.1	EID3	9.7	10.8	2.158068	0.00180213	0.01625433
TC1400009410.hg.1	ZBTB25	8.4	9.6	2.240801	0.00180359	0.01625433
TC0900008109.hg.1	HABP4	5.5	7.1	2.934643	0.00180368	0.01625433
TC0500008494.hg.1	CSNK1G3	8.8	10.0	2.259259	0.00181352	0.01629787
TC0500009707.hg.1	SQSTM1	4.8	6.7	3.708113	0.00181369	0.01629787
TC0500011875.hg.1	PPIC	9.0	10.1	2.171271	0.00181426	0.01629787
TC1200007052.hg.1	GOLT1B	11.9	13.2	2.352778	0.00181443	0.01629787
TC1600008656.hg.1	KLHL36	7.7	9.1	2.610209	0.00181592	0.01629801
TC1500007885.hg.1	NEIL1	5.6	7.0	2.635415	0.00181612	0.01629801
TC2200006768.hg.1	VPREB1	4.2	5.6	2.628026	0.00181923	0.01631218
TC0100009199.hg.1	MFSD14A	11.3	12.4	2.146268	0.00182045	0.01631364
TC1700012440.hg.1	RNFT1	7.0	8.5	2.711977	0.00182091	0.01631364
TC0600013523.hg.1	PPIL4	7.6	9.1	2.841465	0.00182741	0.01635821
TC2000006823.hg.1	ZNF133	5.5	6.7	2.321762	0.00183062	0.01638011
TC1700011808.hg.1	ST6GALNAC2	4.4	5.8	2.664002	0.0018356	0.01641781
TC0100010227.hg.1	CD1D	5.4	6.9	2.69274	0.00184051	0.01644799
TC1900011913.hg.1	LINC00663	3.3	4.5	2.380591	0.00184426	0.01646779
TC0900008483.hg.1	SLC31A1	14.1	15.2	2.159504	0.00186056	0.01659954
TC1900009325.hg.1	MAP2K2	13.9	15.0	2.204972	0.00190001	0.01687426
TC1300010005.hg.1	MCF2L	3.1	4.5	2.688022	0.00190265	0.01687912
TC0500009609.hg.1	FGFR4	8.6	10.8	4.393468	0.00191471	0.01694174
TC1800009277.hg.1	PIAS2	11.7	12.7	2.11771	0.00191851	0.01696829
TC0200015402.hg.1	FAM126B	11.6	12.8	2.252707	0.00191996	0.01697415
TC0600007344.hg.1	PRSS16	4.5	5.6	2.257327	0.0019209	0.01697546
TC2200006614.hg.1	GP1BB; SEPT5	6.9	8.2	2.472842	0.00192261	0.01698358
TC0700008597.hg.1	CUX1	10.8	11.9	2.150318	0.00192744	0.01701224
TC2000008072.hg.1	PPDPF	6.9	8.3	2.483991	0.00193555	0.01705473
TC0200006524.hg.1	TRAPPC12	8.1	9.2	2.16888	0.0019408	0.0170865
TC1200007105.hg.1	LYRM5	7.6	9.0	2.505615	0.00194223	0.0170865
TC1200012808.hg.1	R3HDM2	8.3	9.4	2.152853	0.0019435	0.01709072
TC1400008945.hg.1	RALGAPA1	9.4	10.6	2.199682	0.00195141	0.01712786
TC0100006862.hg.1	FBXO6	7.2	8.6	2.768271	0.0019561	0.01715225
TC1600008128.hg.1	CMTM3	9.3	10.6	2.35405	0.00198109	0.01730064
TC0600011474.hg.1	TNXA	4.7	5.8	2.14126	0.00198669	0.01733546
TC1700011922.hg.1	LGALS3BP	5.8	7.4	2.977332	0.00198868	0.01734574
TC0600008050.hg.1	UBR2	12.3	13.4	2.113451	0.00199051	0.01735462
TC0900006597.hg.1	KDM4C	8.4	9.8	2.74129	0.00199278	0.01736743
TC0100009908.hg.1	ECM1	4.3	5.4	2.149141	0.00199787	0.01739765
TC0200012754.hg.1	FAM161A	6.0	7.6	3.031034	0.00200433	0.01744679
TC1600010409.hg.1	BBS2	7.2	8.4	2.18026	0.00201033	0.01748477

TC0700010581.hg.1	HIBADH	10.7	11.8	2.18993	0.00201884	0.01754459
TC0900008851.hg.1	DNM1	6.7	8.3	3.138391	0.00202744	0.01760831
TC0100009101.hg.1	ABCD3	6.5	7.7	2.368995	0.00202996	0.01761267
TC2100008490.hg.1	BACH1	10.3	11.6	2.472671	0.00203787	0.01764559
TC1100012510.hg.1	HYOU1	14.0	15.1	2.173078	0.00204937	0.0177237
TC0100018282.hg.1	POLR3GL	5.7	7.1	2.637992	0.00207047	0.0178589
TC0400009217.hg.1	KLHL2	5.6	6.7	2.139776	0.00207049	0.0178589
TC0X00008017.hg.1	PLP1	3.9	5.2	2.366763	0.00208078	0.0179231
TC1900007802.hg.1	KIAA0355	6.8	8.7	3.600008	0.00209617	0.01803396
TC0100010609.hg.1	DNM3	3.2	4.6	2.612625	0.00209972	0.01804997
TC1100010976.hg.1	OSBP	9.0	10.2	2.190318	0.00210299	0.01805637
TC1900008404.hg.1	NPAS1	5.4	6.6	2.295367	0.00211115	0.0181047
TC0700013337.hg.1	UMAD1	7.8	8.8	2.121846	0.00212088	0.01814777
TC2200007087.hg.1	RNF185	4.2	5.3	2.22308	0.00212462	0.0181694
TC1600007030.hg.1	NPIPA7; NPIPA8; F	10.9	12.5	3.122389	0.00212566	0.01817101
TC1900011666.hg.1	ZNF627	6.8	8.0	2.422229	0.00213263	0.01822333
TC0100007784.hg.1	AGO4	7.3	8.5	2.309299	0.00213761	0.01825861
TC0700013599.hg.1	ACHE	4.8	6.0	2.373005	0.0021636	0.01841775
TC0100009035.hg.1	KIAA1107	5.8	7.6	3.497721	0.00216594	0.01842201
TC1900011721.hg.1	HKR1	7.0	8.4	2.69316	0.00216796	0.01842976
TC2000007224.hg.1	SPAG4	5.3	6.5	2.226789	0.00217288	0.01844914
TC1400010765.hg.1	RDH11	10.8	12.0	2.225879	0.00217618	0.01844914
TC0200007181.hg.1	YIPF4	10.2	11.3	2.195067	0.00218456	0.01849764
TC0700010321.hg.1	ETV1	7.8	8.9	2.098502	0.00219744	0.01858961
TC1000011454.hg.1	FRA10AC1	10.1	11.3	2.224214	0.00219803	0.01858961
TC1200007701.hg.1	ZNF740	8.7	9.8	2.116675	0.00220873	0.01865807
TC1700006768.hg.1	EFNB3	4.3	5.7	2.561538	0.00221382	0.01869244
TC1700012240.hg.1	TBC1D3H; TBC1D3	7.1	8.5	2.598951	0.00223903	0.01883971
TC1200012802.hg.1	PAN2; CNPY2	5.8	7.2	2.570929	0.00224073	0.01883971
TC1500008995.hg.1	GOLGA8B	9.3	10.8	2.829662	0.0022484	0.01888164
TC1400010592.hg.1	NUBPL	6.5	7.5	2.096467	0.00225342	0.01890159
TC1600009943.hg.1	SEZ6L2	5.7	7.3	3.208625	0.00225873	0.01891348
TC0500006436.hg.1	PLEKHG4B	5.1	6.2	2.195813	0.00225925	0.01891348
TC0300012881.hg.1	SLC33A1	8.8	10.2	2.655097	0.00226229	0.018919
TC1100012186.hg.1	MSANTD4	8.9	10.0	2.086204	0.00226256	0.018919
TC1900009625.hg.1	ICAM3	3.4	4.6	2.283766	0.00226544	0.018919
TC1100012976.hg.1	NUCB2	14.2	15.3	2.117197	0.00226879	0.01893427
TC0900011631.hg.1	GOLGA2	8.7	9.8	2.146974	0.00227577	0.01897771
TC1600007275.hg.1	LCMT1	8.4	9.7	2.538155	0.00228532	0.01904257
TC0300013950.hg.1	EFHB	3.2	4.3	2.080074	0.00228843	0.01906109
TC1900006522.hg.1	MIDN	9.2	10.3	2.139695	0.0022919	0.01908254
TC2200008078.hg.1	GGT2	10.7	11.9	2.354025	0.00230069	0.0191409
TC1600011501.hg.1	NPIPB3	14.5	15.6	2.105868	0.00231602	0.01925065
TC1300007104.hg.1	LRCH1	7.6	8.7	2.151585	0.00231658	0.01925065
TC0400010470.hg.1	SMIM14	7.9	9.4	2.709581	0.00232314	0.01929769
TC2200008091.hg.1	PI4KAP2	10.1	11.3	2.208383	0.00232446	0.01930116
TC0300009035.hg.1	TRPC1	8.0	9.1	2.101675	0.0023295	0.01932808
TC0700013219.hg.1	LMBR1	14.1	15.2	2.089967	0.00233789	0.01937711
TC1200007866.hg.1	SHMT2	14.0	15.1	2.236643	0.00233866	0.01937711
TC0500013429.hg.1	RNF130	9.4	10.6	2.420634	0.00234243	0.01939781

TC0300012596.hg.1	COPB2	11.6	12.7	2.133128	0.00234384	0.01940202
TC0700011318.hg.1	ERV3-1; ZNF117	8.5	9.5	2.062201	0.00235326	0.01945731
TC0200010795.hg.1	SLC11A1	3.8	5.1	2.373778	0.00235415	0.01945731
TC0100006550.hg.1	PRKCZ	9.7	11.0	2.444662	0.00235514	0.01945806
TC1600006580.hg.1	SYNGR3	5.2	6.3	2.148031	0.00235648	0.01946157
TC0200012489.hg.1	PIGF	7.3	8.4	2.077919	0.00236257	0.01948936
TC0100016597.hg.1	STX6	9.9	11.0	2.153727	0.00236655	0.01951471
TC1300008688.hg.1	FOXO1	3.1	4.7	3.070099	0.00237107	0.01953699
TC0100013629.hg.1	YARS	11.3	12.5	2.332803	0.00237454	0.01955804
TC1200012417.hg.1	SLC15A4	9.3	10.4	2.145271	0.00237753	0.01956763
TC0900009307.hg.1	ARRDC1	8.4	9.5	2.131523	0.0023806	0.01958536
TC0300009866.hg.1	CCDC50	9.9	11.0	2.151273	0.00238406	0.01959933
TC0400010886.hg.1	UBA6	8.8	9.9	2.167407	0.00239193	0.01965596
TC1000010327.hg.1	FZD8; MIR4683	9.2	10.6	2.492355	0.00240867	0.01975747
TC0200015226.hg.1	HIBCH	6.6	7.9	2.389063	0.00241078	0.01976546
TC1900008986.hg.1	ZNF264	9.5	10.8	2.507987	0.002413	0.01977604
TC1500008485.hg.1	IGF1R	11.2	12.3	2.125084	0.00241715	0.01980256
TC0100011197.hg.1	SHISA4	4.8	6.2	2.695532	0.00243158	0.01990698
TC0100018484.hg.1	MCL1	13.1	14.3	2.269147	0.00243177	0.01990698
TC1400009123.hg.1	SOS2	7.8	9.1	2.563829	0.00243514	0.01991946
TC0800006866.hg.1	VPS37A	8.1	9.2	2.091829	0.0024423	0.01996281
TC1200007061.hg.1	CMAS	8.4	9.5	2.160987	0.00244951	0.02000646
TC0500007738.hg.1	MAP1B	4.1	5.6	2.934012	0.00245105	0.02000755
TC0X00007382.hg.1	RRAGB	7.7	8.8	2.058316	0.00245244	0.02000755
TC0500007868.hg.1	SCAMP1	10.2	11.3	2.162515	0.00245438	0.0200158
TC2100007508.hg.1	KCNE1	3.9	5.3	2.60466	0.00245867	0.02003557
TC1100011744.hg.1	GAB2	2.7	3.9	2.252536	0.00246105	0.02004731
TC0300011423.hg.1	LRIG1	3.6	4.9	2.43737	0.00246501	0.02006447
TC1900011057.hg.1	PLA2G4C	3.2	4.6	2.471805	0.00246503	0.02006447
TC0900011501.hg.1	NR6A1	5.6	6.9	2.399959	0.00247104	0.02010575
TC0400009526.hg.1	C4orf47	4.2	5.2	2.09731	0.00247402	0.0201148
TC1800006889.hg.1	RIOK3	8.5	9.9	2.627098	0.00247506	0.02011559
TC1000007986.hg.1	ANAPC16	9.3	10.3	2.112095	0.00249554	0.0202744
TC0600007771.hg.1	UHRF1BP1	8.9	10.2	2.363779	0.00253151	0.02051217
TC0400009467.hg.1	TRAPPC11	8.7	9.9	2.239025	0.00254041	0.02056875
TC0300007077.hg.1	ACVR2B	4.4	5.6	2.221524	0.00255458	0.02066794
TC1900008498.hg.1	NUCB1	11.9	13.0	2.148373	0.00256659	0.02072977
TC1900010023.hg.1	FKBP8	9.3	10.5	2.251006	0.00257198	0.02076167
TC2000007239.hg.1	EPB41L1	5.4	6.5	2.096982	0.00258568	0.02083242
TSUnmapped0000024	HYOU1	13.0	14.0	2.061144	0.00258657	0.02083242
TC1000012398.hg.1	FUOM	6.0	7.0	2.072174	0.00259343	0.02086826
TC0500011435.hg.1	LYSMD3	8.6	10.0	2.633616	0.00259394	0.02086826
TC0100018510.hg.1	GLMP	7.4	8.5	2.127427	0.00262052	0.02105839
TC0700010619.hg.1	SCRN1	11.9	13.0	2.140332	0.00262398	0.02107831
TC1100011602.hg.1	UCP2	6.5	7.7	2.246066	0.00264933	0.02125806
TC0200007717.hg.1	AHSA2	7.9	9.0	2.137686	0.00265126	0.02126558
TC2000008110.hg.1	OPRL1	3.1	4.7	2.957258	0.00265463	0.02127673
TC0300013972.hg.1	SLC26A6; MIR682	7.2	8.2	2.047658	0.00266043	0.02130143
TC0700010914.hg.1	CAMK2B	3.1	4.2	2.184707	0.00266648	0.02132387
TC0X00010406.hg.1	TCEAL8	5.9	7.2	2.534402	0.00266865	0.02133329

TC0200007702.hg.1	PAPOLG	9.4	10.5	2.121037	0.00267497	0.02136858
TC1700007057.hg.1	NT5M	7.3	8.5	2.300209	0.00267506	0.02136858
TC1600009050.hg.1	IFT140	5.7	7.2	2.93792	0.00267719	0.02137768
TC0700009020.hg.1	SND1; SND1-IT1	12.5	13.6	2.076797	0.0026852	0.02142562
TC0600012655.hg.1	PNISR	10.4	11.5	2.191502	0.0026892	0.02144959
TC0200012429.hg.1	PPM1B	7.9	9.2	2.624959	0.002697	0.02149161
TC1200012647.hg.1	MYL6B	6.9	8.0	2.229261	0.00269747	0.02149161
TC0100009054.hg.1	MTF2	10.3	11.4	2.051771	0.00270595	0.0215431
TC0800010081.hg.1	RNF122	5.6	6.7	2.119553	0.00271287	0.02158543
TC1100010041.hg.1	ST5	3.3	4.4	2.079418	0.00271328	0.02158543
TC2200006521.hg.1	BCL2L13	11.9	13.1	2.330897	0.00271894	0.02162249
TC0500012372.hg.1	LARS	13.0	14.0	2.097935	0.00272332	0.02164126
TC0900006538.hg.1	JAK2	5.9	7.1	2.273823	0.00272638	0.02164935
TC0700011905.hg.1	BAIAP2L1	9.2	10.3	2.151705	0.00274376	0.02176215
TC1600008735.hg.1	FOXF1	7.9	9.0	2.154353	0.00274564	0.02176215
TC2200009128.hg.1	ALG12	7.4	8.5	2.065513	0.00275052	0.02178477
TC0400008984.hg.1	RPS3A; SNORD73	15.2	16.3	2.122412	0.00275509	0.02180328
TC1600006537.hg.1	BAIAP3	4.1	5.5	2.728721	0.00275591	0.02180328
TC0400012989.hg.1	TMEM192	8.6	9.7	2.096758	0.00277218	0.02189167
TC1400008714.hg.1	SLC22A17	8.5	9.6	2.118452	0.002793	0.02200744
TC0900011655.hg.1	ZER1	9.2	10.4	2.404623	0.0028063	0.02206413
TC0100011533.hg.1	ATF3	6.6	8.3	3.163156	0.00280685	0.02206413
TC2100008408.hg.1	POFUT2	7.8	8.8	2.062816	0.00280842	0.02206413
TC0100009352.hg.1	SYPL2	6.5	7.6	2.218631	0.00281789	0.02213042
TC1200012571.hg.1	ITFG2	10.2	11.4	2.183678	0.00282332	0.02215679
TC1600009958.hg.1	NPIPB4	14.5	15.6	2.055829	0.00282745	0.02218111
TC1900011724.hg.1	ZNF540; ZNF571-A	5.0	6.1	2.209033	0.00282867	0.02218222
TC0800010490.hg.1	TMEM68	10.6	12.1	2.913504	0.00284672	0.02229149
TC0100006619.hg.1	TPRG1L	8.3	9.4	2.197754	0.00284858	0.02229792
TC1400009697.hg.1	NPC2; MIR4709	9.4	10.5	2.067948	0.00286098	0.02238685
TC2100007967.hg.1	PAXBP1	10.5	11.5	2.081466	0.00286334	0.02238893
TC1200012334.hg.1	UBC	15.1	16.2	2.183694	0.00287102	0.02241794
TC0700013291.hg.1	ESYT2	12.7	13.8	2.164989	0.00287227	0.02241794
TC1400010585.hg.1	NOP9	7.1	8.7	3.176009	0.00287996	0.02245343
TC0200016717.hg.1	TBC1D8	5.6	6.7	2.112161	0.00288631	0.02249364
TC0100011812.hg.1	ADCK3	4.8	5.9	2.181446	0.00288943	0.02249781
TC1600006733.hg.1	GLIS2	6.7	7.7	2.078302	0.00289978	0.02256696
TC0300009282.hg.1	TIPARP	9.0	10.3	2.429299	0.00290258	0.02258056
TC2200008706.hg.1	FAM227A	8.8	9.9	2.237837	0.00290509	0.02258366
TC1400009139.hg.1	SAV1	4.3	5.7	2.508404	0.00290765	0.02259542
TC1600011493.hg.1	RPS15A	14.5	15.6	2.079152	0.00291633	0.02263005
TC0500013195.hg.1	ATP6AP1L	5.8	7.0	2.33409	0.00291943	0.02264587
TC2100008506.hg.1	MORC3	9.9	11.0	2.103468	0.00294899	0.02285038
TC1700011574.hg.1	ABCA8	11.8	12.8	2.038694	0.00295386	0.02287985
TC0700010453.hg.1	TOMM7	11.7	12.8	2.128799	0.00295606	0.02288864
TC1600010825.hg.1	ZFHX3	4.4	5.5	2.125769	0.0029599	0.0229101
TC0100016122.hg.1	IGSF8	4.5	5.5	2.071585	0.00296488	0.02294042
TC0X00010675.hg.1	CUL4B	10.7	12.0	2.481659	0.00297144	0.02298284
TC1900006601.hg.1	ZNF554	6.3	7.4	2.069705	0.00297794	0.02302339
TC1100008505.hg.1	EMSY	11.4	12.4	2.03697	0.00297883	0.02302339

TC0200008632.hg.1	RPL31	15.0	16.0	2.055586	0.00298007	0.02302453
TC0300011502.hg.1	FOXP1; RP11-298(	4.6	5.7	2.170053	0.00298112	0.02302453
TC1900006470.hg.1	BSG	15.3	16.3	2.001553	0.00298525	0.02303982
TC0200007821.hg.1	SLC1A4	4.6	5.7	2.121648	0.00299859	0.02310952
TC0800011249.hg.1	RNF19A	5.8	6.9	2.16619	0.00300334	0.02313783
TC0200008259.hg.1	MAT2A	16.9	17.9	2.014191	0.00301338	0.02319402
TC1100011811.hg.1	CCDC90B	9.7	10.8	2.12089	0.00301388	0.02319402
TC0500007901.hg.1	THBS4	4.8	6.0	2.407524	0.00301955	0.02322931
TC2100008558.hg.1	TCP10L	4.3	5.9	3.144552	0.00303386	0.02331427
TC1600007037.hg.1	NPIPA7	11.0	12.4	2.500982	0.00303539	0.02331768
TC1200011081.hg.1	C12orf66	6.6	7.7	2.133659	0.0030436	0.023364
TC1600011351.hg.1	CARHSP1	7.8	9.0	2.240833	0.00304924	0.02339893
TC0600012656.hg.1	USP45	8.6	9.8	2.26521	0.00305225	0.02340532
TC0100008927.hg.1	SH3GLB1	8.5	9.6	2.235217	0.00308133	0.02360298
TC0200012854.hg.1	RAB1A	13.9	15.0	2.080196	0.00312585	0.0238843
TC1500010862.hg.1	ELL3	4.9	6.3	2.771871	0.00312826	0.02388568
TC0500009097.hg.1	SYNPO	4.1	5.2	2.123796	0.00314034	0.02394381
TC0100009386.hg.1	PROK1	5.5	6.5	2.040305	0.00314898	0.02400121
TC1900008287.hg.1	PVRL2	10.5	11.6	2.102958	0.00315044	0.02400381
TC0200015460.hg.1	ICA1L	7.6	8.8	2.248012	0.00316372	0.02407075
TC0800011595.hg.1	EXT1; hunera	6.2	7.3	2.146275	0.00316953	0.02409785
TC0100006874.hg.1	PLOD1	13.2	14.2	2.018223	0.003171	0.02410011
TC0X00007668.hg.1	UPRT	6.7	8.1	2.555774	0.00317207	0.02410011
TC1600010452.hg.1	DOK4	6.0	7.1	2.115986	0.0031745	0.02410857
TC0600012839.hg.1	CDK19	9.7	10.7	2.070493	0.00318889	0.02415087
TC1900011866.hg.1	RAB3D	9.2	10.5	2.39062	0.00319045	0.02415415
TC0X00007529.hg.1	IGBP1	8.9	10.0	2.142374	0.00319264	0.02416223
TC0800007210.hg.1	WRN	5.6	6.9	2.555765	0.00319595	0.0241688
TC1000008276.hg.1	CCSER2	12.4	13.5	2.032034	0.00319612	0.0241688
TC1900007356.hg.1	KCNN1	5.3	6.9	3.069609	0.00321631	0.02428055
TC0600013273.hg.1	BCLAF1	8.4	9.6	2.185654	0.00321729	0.02428055
TC1000008437.hg.1	HECTD2	4.4	5.7	2.488308	0.00321964	0.02428945
TC1800006733.hg.1	PRELID3A	7.4	8.8	2.602377	0.00322557	0.0243156
TC1900006537.hg.1	REEP6	9.9	11.0	2.136901	0.00322952	0.02432975
TC1900006510.hg.1	GPX4	11.3	12.3	2.045779	0.00323189	0.02433907
TC0900008425.hg.1	ZNF483	8.1	9.2	2.163271	0.00323821	0.02436954
TC0700011710.hg.1	SLC25A40	9.9	11.1	2.275266	0.00324466	0.0244009
TC1700008171.hg.1	PRAC2	3.8	4.8	2.044574	0.00325835	0.02447813
TC0600011857.hg.1	CUL7	7.9	9.3	2.584814	0.00326877	0.02454782
TC1200010705.hg.1	GALNT6	3.7	4.9	2.211982	0.00328124	0.02462422
TC0100015397.hg.1	CD58	10.0	11.4	2.702406	0.0033021	0.02475012
TC0100007800.hg.1	THRAP3	8.8	10.3	2.866729	0.00330263	0.02475012
TC0100013349.hg.1	RSRP1	8.6	9.7	2.151324	0.00330714	0.02476656
TC1700009409.hg.1	RTN4RL1	6.0	7.2	2.252239	0.00331163	0.0247829
TC1100012545.hg.1	TRIM29	3.2	4.2	2.025791	0.0033155	0.02480323
TC0300008994.hg.1	PXYLP1	4.5	5.5	2.027891	0.00332177	0.02483281
TC1500007791.hg.1	ARIH1; MIR630	9.5	10.6	2.128069	0.00332657	0.02486003
TC0600006441.hg.1	DUSP22	7.5	8.9	2.767753	0.00333497	0.02490545
TC1500007700.hg.1	RPLP1	15.7	16.8	2.160589	0.00334065	0.02493046
TC0700008096.hg.1	UPK3B	4.1	5.1	2.035735	0.00335649	0.02502261

TC0800007738.hg.1	SDCBP	11.1	12.2	2.031922	0.0033674	0.02508652
TC0700012148.hg.1	SRPK2	7.9	9.2	2.450932	0.00337528	0.02511966
TC1100012681.hg.1	SIAE	13.3	14.3	2.015175	0.00340397	0.02526802
TC2000009956.hg.1	PCMTD2	8.2	9.2	2.074358	0.00340402	0.02526802
TC0400011815.hg.1	MFSD8	10.3	11.6	2.453932	0.00340938	0.0252854
TC0300012397.hg.1	PLXND1	8.1	9.6	2.748148	0.00341837	0.02533422
TC1700009293.hg.1	METRNL	3.6	4.7	2.113816	0.00342676	0.02538296
TC0500011483.hg.1	MCTP1	6.3	7.7	2.69134	0.00342998	0.0253862
TC0900012276.hg.1	SCAI; GOLGA1	8.4	9.5	2.106751	0.00343641	0.02541521
TC1500010764.hg.1	CLK3	8.2	9.2	2.020699	0.00343959	0.02542997
TC1800007829.hg.1	HSBP1L1	7.3	8.5	2.276118	0.00345969	0.02556098
TC2000009744.hg.1	LAMA5; MIR4758	5.9	7.2	2.371163	0.0034869	0.02572657
TC1000011579.hg.1	HPS1; MIR4685	4.6	5.7	2.092423	0.00350488	0.0258059
TC0500007549.hg.1	ZSWIM6	9.2	10.4	2.194131	0.0035061	0.02580608
TC1700012369.hg.1	FLCN	7.0	8.2	2.280484	0.00350872	0.02581646
TSUnmapped0000024(	TMEM42	5.1	6.2	2.133637	0.00351214	0.0258328
TC1900011883.hg.1	ZNF799	7.2	8.4	2.370514	0.00351998	0.02588155
TC0100014127.hg.1	SPATA6	3.2	4.3	2.078439	0.00352631	0.02591923
TC1700010734.hg.1	PLEKHH3	4.6	5.7	2.152615	0.0035348	0.02593718
TC0600011996.hg.1	MUT	7.3	8.3	2.042272	0.0035386	0.02594947
TC0100014250.hg.1	TMEM59	10.4	11.5	2.146246	0.00358608	0.02622679
TC0900008148.hg.1	TDRD7	5.9	7.3	2.488256	0.0035865	0.02622679
TC1700012228.hg.1	LRRC37B	8.8	9.8	2.029431	0.00359394	0.02626332
TC0300009302.hg.1	PQLC2L	4.5	6.6	4.189913	0.00359664	0.02627407
TC1300009012.hg.1	THSD1	9.5	10.5	2.002358	0.00360647	0.02631901
TC1700008032.hg.1	ACBD4	6.2	7.3	2.11449	0.00361781	0.02637332
TC0500013351.hg.1	FNIP1	12.1	13.3	2.169828	0.0036191	0.02637332
TC1700011779.hg.1	ACOX1	9.6	10.6	2.032344	0.00362006	0.02637332
TC1900010528.hg.1	ZNF260	10.8	11.8	2.012028	0.00362578	0.02640602
TC1200012240.hg.1	RSRC2	12.0	13.0	2.099142	0.00363416	0.0264491
TC1300006861.hg.1	NBEA	5.3	6.4	2.121346	0.00364467	0.0265166
TC1200011599.hg.1	LTA4H	11.6	12.7	2.183609	0.00366785	0.02664004
TC1000009111.hg.1	WDR11	9.7	10.7	2.023118	0.00367712	0.02668927
TC1600008330.hg.1	HPR	3.1	4.4	2.425841	0.00368098	0.02669921
TC1600009620.hg.1	SMG1	15.1	16.1	2.001297	0.00368789	0.02674029
TC0100012434.hg.1	HES4	6.9	8.0	2.133644	0.00369782	0.02678483
TC0200013902.hg.1	RGPD5; RGPD8	13.9	15.1	2.185177	0.00369902	0.02678483
TC1200012210.hg.1	MORN3	4.3	5.3	2.024282	0.00370221	0.02679884
TC0500007314.hg.1	MRPS30	13.8	14.9	2.117196	0.00371201	0.02685167
TC0500009620.hg.1	RGS14	6.6	7.6	2.01253	0.00372542	0.0269214
TC1200009927.hg.1	TAS2R20	7.0	8.4	2.716709	0.00374223	0.02702237
TC1900008965.hg.1	ZFP28	7.3	8.5	2.282516	0.00374877	0.02704458
TC0200009936.hg.1	GAD1	5.8	6.8	2.042357	0.00376765	0.02716254
TC1900007314.hg.1	OCEL1	4.2	5.4	2.312038	0.00377663	0.027209
TC2100008499.hg.1	SMIM11A	9.8	10.8	2.007396	0.00378717	0.0272666
TC1400006659.hg.1	MMP14	4.5	5.8	2.504469	0.00379471	0.0272934
TC1400006823.hg.1	SCFD1	9.3	10.5	2.233088	0.00380478	0.02734118
TC2200009352.hg.1	LOC400927; CSNK	8.4	9.6	2.258446	0.00380941	0.02735889
TC0500013292.hg.1	11-Mar	4.0	5.1	2.092975	0.00381019	0.02735889
TC0200015958.hg.1	DNER	11.3	12.3	2.085329	0.0038351	0.02751928

TC1400007500.hg.1	ARG2	6.3	7.9	3.065398	0.00383926	0.02753091
TC0800007209.hg.1	WRN	9.3	10.5	2.210044	0.00384478	0.02755538
TC1500010134.hg.1	CIB2	6.7	7.7	2.057218	0.00387049	0.02768988
TC0900007646.hg.1	VPS13A	6.4	7.4	2.026303	0.00389089	0.0278173
TC1700007481.hg.1	RAB11FIP4; MIR47	3.5	5.2	3.383943	0.00389803	0.02785902
TC1600011045.hg.1	MBTPS1	12.0	13.2	2.339062	0.00390396	0.02788283
TC0800009579.hg.1	XKR6	5.0	6.0	2.051728	0.00390891	0.0279089
TC1700012396.hg.1	TBC1D3C	6.2	7.3	2.201024	0.00391072	0.02791253
TC1900010851.hg.1	ETHE1	6.8	7.9	2.212259	0.00392742	0.02795726
TC0200016669.hg.1	ASB3	9.2	10.3	2.194664	0.00396515	0.02816046
TC0500013425.hg.1	C5orf45	7.5	8.7	2.335829	0.00397004	0.02818586
TC0100010297.hg.1	NCSTN	10.1	11.2	2.162102	0.00398688	0.02827729
TC0800009764.hg.1	PSD3	12.6	13.8	2.218239	0.00399787	0.02834589
TC0300009147.hg.1	RNF13	11.6	12.6	2.070781	0.00401282	0.0284237
TC1400006664.hg.1	REM2	6.0	7.3	2.369758	0.00401519	0.02843108
TC2200007932.hg.1	PI4KAP1	10.9	12.0	2.068923	0.00402817	0.02848418
TC1100013194.hg.1	CORO1B	8.3	9.4	2.103096	0.00402933	0.02848418
TC1500009926.hg.1	LARP6	7.5	8.6	2.194314	0.00403749	0.02851617
TC0900012033.hg.1	DPP7	10.1	11.3	2.245233	0.00405113	0.02856298
TC1000007427.hg.1	ZNF485	6.4	7.6	2.207893	0.00405388	0.02856471
TC2200006701.hg.1	RIMBP3C; RIMBP3	3.9	5.1	2.203925	0.00406236	0.02859517
TC1200011675.hg.1	GOLGA2P5	5.5	6.8	2.340627	0.0040734	0.02862642
TC0400008879.hg.1	GAB1	5.2	6.4	2.155399	0.00407347	0.02862642
TC0600010235.hg.1	MLLT4	10.2	11.3	2.040404	0.00408441	0.02868447
TC1100009657.hg.1	IFITM3	14.0	15.0	2.056805	0.00410502	0.02879155
TC0200010473.hg.1	FZD7	6.6	7.7	2.038941	0.00412815	0.02894425
TC0100015271.hg.1	OVGP1	3.5	4.5	2.002323	0.00417873	0.02916542
TC0400008444.hg.1	LARP7	2.8	3.9	2.241788	0.00418781	0.02921147
TC1600011526.hg.1	PRSS53	4.3	5.5	2.389022	0.00422905	0.02943047
TC0X00009659.hg.1	PRICKLE3	6.1	7.1	2.082014	0.00424393	0.0294933
TC1900009459.hg.1	INSR	6.7	8.1	2.46856	0.00427481	0.02965268
TC1200011767.hg.1	SLC41A2	7.2	8.4	2.323678	0.00433792	0.02998376
TC0900007064.hg.1	DNAJB5	4.8	6.2	2.543376	0.00434348	0.03001251
TC1700012241.hg.1	TBC1D3L; TBC1D3	6.8	8.0	2.240483	0.00435048	0.03004792
TC0600007108.hg.1	RNF144B	3.3	4.3	2.029557	0.0043594	0.03007467
TC0900007977.hg.1	CARD19	7.7	9.0	2.411274	0.00435948	0.03007467
TC0400007789.hg.1	RUFY3	6.1	7.3	2.257147	0.00440257	0.03026486
TC1700006432.hg.1	SCGB1C2	6.7	7.7	2.1111	0.00440497	0.03027162
TC0X00009836.hg.1	SPIN3	8.1	9.1	2.062895	0.00442202	0.03034995
TC0800007316.hg.1	EIF4EBP1	9.2	10.3	2.135524	0.0044267	0.03036257
TC2200007783.hg.1	PIM3	6.4	7.4	2.003226	0.00447689	0.03061874
TC0300013989.hg.1	ABHD14B	8.2	9.4	2.317701	0.00452515	0.0308897
TC0600011805.hg.1	USP49	6.3	7.5	2.241726	0.00453296	0.03093314
TC0400011208.hg.1	FAM175A	8.3	9.4	2.16011	0.00455008	0.03096909
TC1700008499.hg.1	BCAS3	7.1	8.3	2.342388	0.00455407	0.03096909
TC0400008977.hg.1	MAB21L2	4.1	5.2	2.212044	0.00456698	0.03101731
TC0100015180.hg.1	WDR47	9.2	10.2	2.013185	0.00457798	0.0310625
TC0200016719.hg.1	SNORD89; RNF14	6.4	7.6	2.288647	0.00459217	0.03113907
TC1900011235.hg.1	KLK12	4.1	5.3	2.271625	0.00460394	0.03117943
TC0700012797.hg.1	PARP12	7.9	9.5	3.091774	0.0046249	0.0312819

TC0100017720.hg.1	LOC149373; RP5-1	2.9	3.9	2.016167	0.00462969	0.03130439
TC1500008269.hg.1	KIF7	4.4	5.8	2.704103	0.00463197	0.03130994
TC1400006658.hg.1	MRPL52	8.2	9.3	2.082396	0.00464176	0.03135635
TC1900010370.hg.1	SLC7A9	3.5	4.5	2.106116	0.00465006	0.03139269
TC0600011808.hg.1	MED20	7.7	8.9	2.295526	0.00465877	0.03142182
TC1700008690.hg.1	PITPNC1	6.5	7.5	2.090865	0.00466152	0.03142972
TSUnmapped0000040!	DGKD	5.7	7.1	2.56057	0.0046722	0.03146293
TC0300012155.hg.1	HGD	3.6	4.8	2.305645	0.00467563	0.03146624
TC1400007639.hg.1	C14orf169	8.0	9.0	2.090264	0.00469665	0.03157799
TC2100006991.hg.1	KCNE2	2.8	3.8	2.014309	0.0047157	0.03168616
TC0200008468.hg.1	FAHD2A	7.9	9.0	2.043122	0.00471794	0.03169133
TC0X00008945.hg.1	PRKX	8.6	9.7	2.093468	0.00473194	0.03173568
TC0100012473.hg.1	CCNL2	12.1	13.4	2.454279	0.00475239	0.03182305
TC1900010005.hg.1	RAB3A	4.5	6.0	3.02084	0.00479185	0.03203725
TC1900011861.hg.1	S1PR2	4.9	6.0	2.075185	0.00479727	0.03205353
TC0700007993.hg.1	CLDN4	5.1	6.5	2.586937	0.00480316	0.0320629
TC0200016607.hg.1	UBE2F	7.2	8.3	2.022795	0.00480912	0.03207691
TC0300008705.hg.1	ABTB1	4.9	6.0	2.122243	0.00480974	0.03207691
TC1600008021.hg.1	MMP15	5.9	7.0	2.050201	0.00481665	0.03210302
TC2200008856.hg.1	NAGA	7.9	9.0	2.107752	0.00483499	0.0321871
TC0100010332.hg.1	NIT1	7.3	8.4	2.139613	0.00483527	0.0321871
TC0600009365.hg.1	NCOA7	5.6	6.6	2.095617	0.00486558	0.03233872
TC2000008299.hg.1	GPCPD1	7.5	8.9	2.67618	0.0048753	0.03237317
TC1700009393.hg.1	RILP	5.5	6.7	2.245318	0.0048894	0.03243667
TC1200007844.hg.1	RBMS2	8.6	9.8	2.356164	0.0049187	0.03258067
TC0800009289.hg.1	ZNF517	4.6	5.8	2.294866	0.00495573	0.03271485
TC0100009482.hg.1	HIPK1	5.8	7.0	2.38831	0.00495824	0.03272134
TC0100011833.hg.1	ZNF678	3.8	5.0	2.368798	0.00497217	0.0327931
TC0100009696.hg.1	LOC388692; FAM2	3.5	4.6	2.068435	0.00499395	0.03290636
TC0100017597.hg.1	C1orf145	3.4	4.5	2.139487	0.00503618	0.033111
TC1500009224.hg.1	STRC	3.6	4.8	2.41175	0.0050958	0.03341318
TC1600011358.hg.1	C16orf45	9.4	10.5	2.109075	0.00511706	0.0334997
TC2000007670.hg.1	SNAI1	5.7	6.9	2.277553	0.00511994	0.0334997
TC1900006582.hg.1	OAZ1	9.5	10.7	2.369397	0.00512148	0.0334997
TC1000010411.hg.1	ZNF33B	8.4	9.5	2.172047	0.0051977	0.03388456
TC1700010198.hg.1	UNC119	9.6	10.6	2.002725	0.00524687	0.03412218
TC0900009970.hg.1	OR13C7	3.0	4.1	2.144661	0.00526871	0.0342123
TC0200012971.hg.1	ANXA4	3.5	4.9	2.588623	0.00528217	0.03425655
TC2100007263.hg.1	PDE9A	5.4	6.6	2.275029	0.00529218	0.03428349
TC0300013255.hg.1	ZMAT3	7.3	8.6	2.410588	0.00535908	0.03456887
TC1900008326.hg.1	FOSB	4.0	5.3	2.372898	0.00536375	0.03458861
TC0400009958.hg.1	ABLIM2	5.6	6.7	2.153234	0.00537256	0.03461631
TC0800008916.hg.1	EFR3A	9.2	10.3	2.015224	0.00539875	0.03471842
TCUn_GL000219v100(	LOC283788; AL592	5.0	6.2	2.381078	0.00540007	0.03471842
TC2000009922.hg.1	SGK2	2.9	4.0	2.120368	0.00543134	0.03483594
TC2100006980.hg.1	ITSN1	4.9	5.9	2.032873	0.00543921	0.03485511
TC1900011280.hg.1	ZNF350	6.7	7.7	2.104176	0.00545601	0.03494193
TC1600011316.hg.1	ARHGDIG	3.3	4.4	2.139302	0.005462	0.03496986
TC0500012312.hg.1	SPRY4	12.7	13.7	2.058431	0.0054777	0.03503901
TC0800012362.hg.1	KHDRBS3	2.9	4.0	2.063366	0.00550768	0.0351678

TC0700008690.hg.1	KMT2E	12.2	13.2	2.014337	0.00551955	0.03521217
TC1300006690.hg.1	POLR1D	8.6	9.7	2.068019	0.00554965	0.0353306
TC1000011017.hg.1	CFAP70	3.4	4.5	2.082772	0.00556351	0.03539785
TC0800009529.hg.1	PPP1R3B	4.0	5.3	2.487937	0.00558386	0.03551679
TC1900009240.hg.1	GNG7	7.6	8.8	2.220124	0.00558848	0.03553564
TC1000011892.hg.1	CCDC186; MIR211	8.9	10.0	2.14577	0.00559299	0.03554321
TC1100009689.hg.1	DEAF1	8.9	10.0	2.094651	0.00560468	0.03558888
TC1300010029.hg.1	N4BP2L1	4.0	5.9	3.69627	0.00560839	0.03558888
TC1500007180.hg.1	GALK2	9.6	10.7	2.075178	0.00560864	0.03558888
TC1900011725.hg.1	SPINT2	6.5	7.6	2.115128	0.00561882	0.03562969
TC0100013192.hg.1	SH2D5	5.9	7.1	2.302522	0.00563497	0.03568314
TC2100008520.hg.1	B3GALT5	3.0	4.2	2.312839	0.00564486	0.03571415
TC0500011523.hg.1	LNPEP	6.2	7.3	2.081834	0.005653	0.03574458
TC0100015747.hg.1	GOLPH3L	9.0	10.1	2.084166	0.00573592	0.03609539
TC0300013855.hg.1	NFKBIZ	9.9	11.1	2.249415	0.00575985	0.03621733
TC1200012080.hg.1	TESC	4.6	5.7	2.048078	0.00578049	0.03632581
TC1900011965.hg.1	PSG2	4.1	5.3	2.239645	0.00581122	0.03648685
TC1100013196.hg.1	TMEM134	8.0	9.1	2.145933	0.00581746	0.03650464
TC0300014065.hg.1	SERP1	7.3	8.3	2.017376	0.00582105	0.03651651
TC0700010182.hg.1	PMS2	12.0	13.2	2.183708	0.00583416	0.03653459
TC0X00008833.hg.1	FAM50A	9.1	10.3	2.32512	0.00585976	0.03662005
TC1700006735.hg.1	SLC2A4	4.6	5.7	2.111232	0.00591024	0.03687111
TC0500008539.hg.1	GRAMD3	11.0	12.1	2.038828	0.0059713	0.03714399
TC0700013182.hg.1	CNPY1	3.8	5.0	2.349396	0.00599506	0.03727014
TC1200012763.hg.1	TAS2R30; TAS2R4	8.4	9.5	2.119031	0.00600248	0.0373055
TC0500010781.hg.1	SLC38A9	5.7	7.0	2.431067	0.0060527	0.03749808
TC0400009904.hg.1	PPP2R2C	3.4	4.6	2.282041	0.00608567	0.03765878
TC0600014196.hg.1	SNX9	11.2	12.2	2.013269	0.00614411	0.03787837
TC0100009064.hg.1	FNBP1L	10.4	11.4	2.082787	0.0061483	0.03789332
TC0900009225.hg.1	EGFL7	7.7	8.9	2.234164	0.00616755	0.03795633
TC1600007368.hg.1	ATP2A1	6.2	7.2	2.010989	0.00619285	0.03808402
TC0800012435.hg.1	ZFAND1	8.6	9.7	2.079556	0.00624364	0.03830477
TC0200011890.hg.1	LAPTM4A	3.7	5.2	2.764378	0.00630683	0.03857113
TC0500008342.hg.1	DCP2	10.5	11.6	2.114476	0.00631607	0.03859958
TC0100006604.hg.1	PRDM16	5.5	6.5	2.07272	0.00632666	0.03861941
TC1200012663.hg.1	RAB3IP	5.3	6.6	2.575915	0.00632733	0.03861941
TC2200009284.hg.1	ARHGAP8	2.9	4.0	2.201116	0.00635035	0.03868281
TC0600014321.hg.1	SNX14	9.4	10.4	2.031386	0.00638231	0.03886649
TC0600007203.hg.1	ACOT13	7.4	8.5	2.1105	0.00651561	0.03934313
TC0200010518.hg.1	ABI2	10.3	11.4	2.072755	0.00652498	0.0393886
TC2000009196.hg.1	SERINC3	13.0	14.0	2.024906	0.00656422	0.03955871
TC0600014300.hg.1	GCLC	6.9	8.1	2.215312	0.00659657	0.03969401
TC0300013813.hg.1	ACKR2	4.8	6.0	2.217002	0.00663064	0.03984699
TC2200007505.hg.1	3-Sep	6.6	7.7	2.105744	0.00668003	0.04004512
TC1100008831.hg.1	ENDOD1	4.3	5.3	2.074703	0.00668943	0.04008799
TC0700011147.hg.1	VOPP1	7.5	8.7	2.251458	0.00672681	0.04028609
TC0700013423.hg.1	ZNF655	8.7	9.7	2.015203	0.00672812	0.04028609
TC1000012429.hg.1	PRPF18	7.6	8.7	2.22144	0.00676783	0.04046736
TC2000009809.hg.1	KCNQ2	7.8	8.9	2.162201	0.00680649	0.04064188
TC1700008878.hg.1	CDR2L	7.0	8.0	2.00588	0.00681791	0.04068796

TC1900006588.hg.1	GADD45B	9.4	10.5	2.166483	0.00690293	0.04107501
TC1200007594.hg.1	ASIC1	6.0	7.4	2.745749	0.00691222	0.04110153
TC2000007443.hg.1	TOX2	2.8	3.8	2.067977	0.0069574	0.04125405
TC0100018303.hg.1	EFNA3	4.0	5.1	2.108833	0.00696866	0.04129496
TC0700013349.hg.1	GHRHR	3.8	5.0	2.34372	0.00701894	0.04148311
TC0X00008612.hg.1	MAGEC1	2.7	3.8	2.043554	0.00702108	0.04148431
TC1200012592.hg.1	C1S	5.3	6.6	2.566665	0.00706165	0.04166667
TC2200008229.hg.1	LRRC75B	4.8	6.1	2.394069	0.00707369	0.04171475
TC1700012032.hg.1	LINC00482	5.4	6.5	2.207036	0.00718562	0.04220076
TC0600013144.hg.1	C6orf191andARHG	10.3	11.3	2.017676	0.00725844	0.04253525
TC0700011119.hg.1	SEC61G	11.6	12.7	2.158292	0.00729881	0.04273677
TC1900011597.hg.1	ZNF606	6.4	7.8	2.700506	0.00735995	0.04297744
TC1500010740.hg.1	DTWD1	7.5	8.5	2.055536	0.00739905	0.0431353
TC1900011132.hg.1	SLC6A16	5.9	6.9	2.029445	0.00747081	0.04344736
TC0300011899.hg.1	CBLB	3.9	4.9	2.006171	0.00747979	0.04348781
TC0900011072.hg.1	ABCA1	4.9	5.9	2.010779	0.00751268	0.04360809
TC0800012323.hg.1	CA13	3.3	4.3	2.01797	0.00752043	0.04364128
TC1200010806.hg.1	MAP3K12	5.2	6.3	2.085799	0.00759287	0.04393439
TC1900011172.hg.1	VRK3	10.8	11.8	2.008822	0.0076269	0.04409902
TC0400007572.hg.1	EXOC1	7.9	9.0	2.11812	0.00763024	0.04409955
TC0600011583.hg.1	C6orf1	7.0	8.3	2.427321	0.00774218	0.04457835
TC1100006549.hg.1	LINC01219	3.2	4.3	2.118136	0.00775521	0.04462941
TC0400012621.hg.1	ACSL1	8.4	9.6	2.24605	0.00778749	0.0447911
TC0900011209.hg.1	C9orf84	3.3	4.3	2.081957	0.00794506	0.04545361
TC1900011753.hg.1	ZNF284	6.3	7.7	2.578318	0.00801595	0.04571285
TC0400012807.hg.1	PRR27	2.9	4.1	2.351604	0.00803275	0.04578608
TC0100013303.hg.1	HMGCL	5.9	7.0	2.207855	0.00804582	0.04583016
TC0200012085.hg.1	FNDC4	5.7	7.1	2.718282	0.0080472	0.04583016
TC0300010123.hg.1	CRBN	7.5	8.8	2.418194	0.00806822	0.04592549
TC1100011980.hg.1	TAF1D; SNORA8; S	10.7	11.7	2.006025	0.00808749	0.04600075
TC1700008852.hg.1	CD300A	4.6	5.6	2.098015	0.0081892	0.04636802
TC0200015427.hg.1	TRAK2	6.6	7.7	2.13528	0.00821237	0.04647362
TC1900011606.hg.1	ZNF329	5.8	7.3	2.674594	0.00826126	0.04664054
TC0900012025.hg.1	ABCA2	7.5	8.8	2.327433	0.00830178	0.04679519
TC0600013743.hg.1	TAGAP	4.5	5.7	2.341252	0.00831445	0.04680533
TC1700012450.hg.1	GH1	4.1	5.2	2.16556	0.0083528	0.04698424
TC1900008011.hg.1	KCNK6	9.2	10.5	2.338291	0.00835991	0.04698651
TSUnmapped0000057	PRAMEF25	3.5	4.6	2.283442	0.00840153	0.04712393
TC0600007617.hg.1	C6orf48; SNORD52	9.3	10.3	2.012398	0.00841347	0.04717704
TC0900012155.hg.1	MSANTD3-TMEFF1	5.8	7.3	2.826634	0.0084374	0.04727415
TC0200007411.hg.1	PPM1B	9.2	10.3	2.073014	0.00844594	0.04729726
TC1900008113.hg.1	LTBP4	4.9	5.9	2.044014	0.00845786	0.04735167
TC0700007137.hg.1	BBS9	6.4	7.5	2.138049	0.00846289	0.04736746
TC1200007816.hg.1	DGKA	3.9	4.9	2.089206	0.00849574	0.04751408
TC0700013224.hg.1	LMBR1	7.0	8.4	2.758406	0.00852262	0.04762666
TC1000009447.hg.1	KNDC1	5.1	6.3	2.416384	0.00856181	0.0477591
TSUnmapped0000066	TRAPPC4	6.3	7.7	2.630095	0.00862422	0.04804094
TC0100018221.hg.1	C1orf50	7.1	8.5	2.729658	0.00867749	0.04825377
TC1400009239.hg.1	GCH1	3.6	4.8	2.284732	0.00870351	0.0483257
TC0800011597.hg.1	EXT1; spawla	5.7	6.9	2.340935	0.00871119	0.04833553

TC0200009982.hg.1	RAPGEF4	3.5	4.7	2.253965	0.00874087	0.04840004
TC1700007918.hg.1	AOC2	4.8	5.9	2.078957	0.00878981	0.04854822
TC0100017984.hg.1	AKT3	5.0	6.3	2.516955	0.0088005	0.04856365
TC2000009461.hg.1	ATP9A	5.1	6.6	2.758005	0.00880566	0.04856365
TC1900010855.hg.1	CADM4	7.0	8.1	2.044226	0.00883084	0.04865776
TC1600009100.hg.1	ECI1	7.2	8.5	2.421381	0.00891486	0.04893932
TC0100015586.hg.1	PDZK1	5.5	6.6	2.21387	0.00894652	0.04900024
TC1900008328.hg.1	PPM1N	4.8	5.9	2.074603	0.00898013	0.04914669
TC1000012497.hg.1	BORCS7-ASMT	7.5	8.5	2.026191	0.00908585	0.04961134
