# Supplementary material for: Nutrient restriction-activated Fra-2 promotes tumor progression via IGF1R in miR-15a downmodulated pancreatic ductal adenocarcinoma
Source: Signal Transduct Target Ther. 2024 Feb 12;9:31. doi: 10.1038/s41392-024-01740-4 (PMC10859382; doi:10.1038/s41392-024-01740-4)
Supplement: Supplementary file 4 — Supplementary Table 4 [file 41392_2024_1740_MOESM4_ESM.pdf]

ID	miR-15a	IGF1R	Fra-2	Age at diagnosis	Gender	Surgical resection	Staging	Grading	time to recurrence (months)	overall survival (months)	Status (0=alive; 1=dead)
**2**	2.47	30	30	72	M	SP	1B	2	11.25	14.38	1
**3**	0.79	20	15	72	M	DSP	2A	3	32.24	34.93	1
**4**	0.25	0	0	53	M	PD	2B	3	3.91	4.80	1
**5**	0.61	40	10	56	M	PPPD	2A	not reported	35.03	178.72	0
**6**	0.40	30	0	71	F	PPPD	2B	not reported	14.34	14.34	1
**7**	0.42	50	0	73	F	DSP	2A	2	no event	69.84	0
**8**	0.42	70	60	63	F	PPPD	2A	2	no event	12.83	0
**9**	0.12	60	60	69	M	PPPD	2A	2	9.44	25.13	1
**11**	0.18	70	40	56	M	PPPD	1B	3	no event	18.22	0
**12**	0.23	0	0	83	M	PPPD	2A	1	2.43	7.63	1
**13**	0.06	80	80	65	M	PPPD	2A	3	1.09	1.09	1
**14**	0.04	75	70	70	M	PPPD	2B	2	2.86	25.59	1
**15**	0.15	55	70	74	F	SP	2B	2	no event	4.74	0
**16**	0.43	25	20	56	M	PPPD	2B	3	12.11	61.51	0
**17**	0.10	80	80	78	F	PPPD	2B	2	8.13	14.21	1
**18**	0.19	80	70	75	F	SP	2B	3	30.92	30.92	1
**19**	0.19	60	50	82	F	DSP	2B	3	6.22	10.46	1
**20**	0.18	50	30	60	M	PPPD	2B	2	14.11	24.93	1
**21**	0.29	10	20	67	F	PPPD	2B	2	no event	4.11	0
**23**	0.20	0	20	76	F	SP	2B	3	no event	8.06	1
**24**	0.09	70	80	42	M	PPPD	2B	2	6.64	14.11	1
**26**	0.17	0	40	79	M	SP	2A	3	no event	5.10	0
**27**	0.18	60	60	56	M	PPPD	2B	3	9.01	9.01	1
**28**	0.13	80	75	78	M	SP	2B	3	no event	0.66	0
**29**	0.03	90	70	73	F	SP	2B	3	no event	1.88	0
**31**	0.08	80	80	71	M	PPPD	2A	2	no event	2.80	0
**32**	0.22	20	40	66	M	PPPD	2B	2	20.92	20.92	1
**33**	0.13	50	90	78	F	SP	2B	2	no event	14.97	0
**34**	0.15	0	20	73	F	SP	2B	2	4.44	11.71	1
**35**	0.10	90	80	51	F	PPPD	2B	3	no event	0.33	0
**36**	0.03	90	90	71	F	DSP	2A	2	no event	0.92	0
**37**	0.18	70	80	82	M	DSP	2A	3	no event	1.12	0
**38**	0.15	55	80	59	F	PPPD	2B	1	no event	7.50	0
**39**	0.27	70	40	75	M	PD	2B	2	0.95	0.95	1
**40**	0.13	80	80	71	M	DSP	2A	3	8.16	8.16	1
**41**	0.24	30	60	60	F	PD	2B	3		not available	
**43**	0.13	30	30	62	M	DSP	2B	3	11.35	21.78	1
**44**	0.15	40	20	74	F	SP	2B	not reported	no event	29.93	0
