# Supplementary material for: Nutrient restriction-activated Fra-2 promotes tumor progression via IGF1R in miR-15a downmodulated pancreatic ductal adenocarcinoma
Source: Signal Transduct Target Ther. 2024 Feb 12;9:31. doi: 10.1038/s41392-024-01740-4 (PMC10859382; doi:10.1038/s41392-024-01740-4)
Supplement: Supplementary file 5 — Supplementary Table 5 [file 41392_2024_1740_MOESM5_ESM.pdf]

ID	Gene Symbol	GL_C-diet Avg (log2)	KPP_C-diet Avg (log2)	Fold Change	P-val
TC0500000834.mm.2	Alb	16.33724	6.245717	1091.067	0.02064916
TC0300002684.mm.2	Chil3	17.49918	9.208976	313.0391	0.001159738
TC0300002685.mm.2	Chil4	13.74232	7.459085	77.88303	0.002636788
TC1400002002.mm.2	Rnase2a	9.72858	4.948689	27.47203	1.34E-06
TC1700001757.mm.2	Fkbp5	15.37361	10.65844	26.26689	0.000613271
TC1400000459.mm.2	Ear12; Ear2; E	13.33388	10.16851	8.971628	0.00315512
TC1100000900.mm.2	Per1	9.650115	6.677153	7.851463	0.005210708
TC0400002275.mm.2	Atp6v0d2	14.82153	12.32702	5.635367	0.02882726
TC1700001671.mm.2	Msln	11.30907	8.834246	5.558993	0.001220171
TC0200003574.mm.2	Cytip	9.905741	7.449884	5.48639	0.000628174
TC0800003059.mm.2	Slc7a5	10.66006	8.212318	5.455626	0.02942317
TC0100003599.mm.2	Ifi202b; Ifi205	8.163991	5.719613	5.442911	0.001491657
TC1000000215.mm.2	Ctgf	16.65755	14.22789	5.387699	0.007320641
TC1100001285.mm.2	Car4	9.526302	7.128925	5.268444	0.000830623
TC0600003237.mm.2	Olr1	11.88191	9.492996	5.237636	0.006524535
TC0X00003075.mm.2	Tsc22d3	11.30163	8.991127	4.960576	0.009074682
TC1500002075.mm.2	Abcd2	10.46706	8.184612	4.865015	0.000765451
TC0900000053.mm.2	Mmp7	14.10656	11.90916	4.586518	0.006611529
TC1700001938.mm.2	D17H6S56E-5	18.7206	16.52988	4.565327	0.000287266
TC0500002687.mm.2	Tmprss11e	9.319248	7.149263	4.500187	0.004361507
TC0800000115.mm.2	F10	9.775474	7.6477	4.370425	0.02396625
TC1700001522.mm.2	Fpr1	9.503291	7.421818	4.232394	0.02425543
TC0600000724.mm.2	Mmrn1	7.891918	5.813671	4.222936	0.01806395
TC1100003093.mm.2	Alox15	8.225152	6.14998	4.213947	0.000240051
TC0100003593.mm.2	AI607873	13.88598	11.81272	4.208342	0.03734305
TC0200004647.mm.2	Siglec1	6.813441	4.765677	4.134646	0.006630103
TC1000000171.mm.2	Sgk1	13.26196	11.26163	4.000892	0.004181499
TC0800000482.mm.2	Slc7a2	12.77782	10.78613	3.977042	0.001698891
TC1700000528.mm.2	Abcg1	10.44003	8.478856	3.893793	0.002159876
TC0400001790.mm.2	Hspb7	8.124698	6.21445	3.758736	0.001309828
TC0500001674.mm.2	C130050O18R	7.264188	5.35483	3.756418	0.000468074
TC1400001868.mm.2	Ear1	6.796003	4.89595	3.732268	0.04482986
TC1400001872.mm.2	Ear10	8.918098	7.024847	3.714716	0.001518489
TC0100003056.mm.2	Rassf5	7.731527	5.891224	3.580853	0.000366261
TC0700002644.mm.2	Axl	14.12643	12.29254	3.564961	0.008106419
TC1100003399.mm.2	Ccl6	17.24437	15.42568	3.527589	0.03091471
TC0200004537.mm.2	Atp8b4	8.93498	7.198631	3.331911	0.003406442
TC1300002143.mm.2	Ctla2a	13.78538	12.06992	3.284015	0.006333313
TC0800001244.mm.2	Pla2g15	12.41929	10.73081	3.223166	0.02425956
TC0700003826.mm.2	Relt	9.211276	7.562772	3.135083	0.01619869
TC0400001039.mm.2	Lepr	10.48546	8.866224	3.072113	0.03464897
TC1600001337.mm.2	Klhl6	11.35321	9.760429	3.016311	0.004937092
TC0300002053.mm.2	P2ry13	8.823354	7.232824	3.011597	0.003387784
TC0700004011.mm.2	Gm1966	9.854846	8.275301	2.988757	0.02614985
TC0300002051.mm.2	P2ry14; F6301	9.239581	7.667581	2.973166	0.00114729
TC0300000656.mm.2	Tmem154	9.874966	8.352552	2.872712	0.005096207

TC0400001557.mm.2	Laptm5	15.52153	14.00068	2.869594	0.03296331
TC0600003264.mm.2	Klra2	9.520571	8.005501	2.858126	0.007526171
TC0700002950.mm.2	Cd33	10.99417	9.48386	2.848708	0.003800752
TC1100001564.mm.2	Ppp1r1b	6.010007	4.508308	2.83176	0.008083662
TC1000001897.mm.2	Arg1	12.5893	11.11823	2.772279	0.01772594
TC1300000735.mm.2	Dapk1	12.79408	11.34296	2.7342	0.01949534
TC0200002602.mm.2	Ptpn1	14.6563	13.20698	2.730805	0.001903313
TC1100004060.mm.2	Cd300lf	12.74678	11.29954	2.726865	0.000274214
TC1400001528.mm.2	Kcnk5	7.096646	5.653276	2.719553	0.01048173
TC0700000028.mm.2	Lilra5	6.787347	5.367136	2.676248	0.01001635
TC0300002357.mm.2	Il6ra	9.791928	8.371807	2.67608	0.000443891
TC0700003804.mm.2	Slco2b1	7.334639	5.945642	2.618965	0.02578664
TC0800000020.mm.2	Mcemp1	8.483843	7.108293	2.594669	0.02222211
TC1100000802.mm.2	Trpv2	7.732379	6.357662	2.593172	0.005195417
TC0300003225.mm.2	Sirpb1a	12.97938	11.60478	2.592953	0.009002181
TC0800002847.mm.2	Dpep2	8.874156	7.500773	2.590774	0.008533218
TC1200001460.mm.2	Lpin1	9.572991	8.205857	2.579576	0.002520303
TC0200000432.mm.2	Bmyc	8.685225	7.31891	2.578112	0.03853454
TC0800002441.mm.2	Fcho1	5.230795	3.877311	2.555284	0.000199428
TC0300001110.mm.2	AI504432	7.899535	6.558345	2.533601	0.007312634
TC0400004097.mm.2	Kcnab2	7.577939	6.254871	2.501976	0.002943654
TC0400003888.mm.2	Plekhm2	10.81457	9.505902	2.477135	0.04362212
TC0100001574.mm.2	Fcgr4	9.321333	8.013082	2.476411	0.005367724
TC1100003090.mm.2	Alox12	6.294869	4.990426	2.469884	0.004809293
TC1700002814.mm.2	C4b	14.54004	13.23689	2.467656	0.02708985
TC0700004104.mm.2	Lyve1	8.983562	7.680816	2.466981	0.03688438
TC0300001674.mm.2	Sirpb1a; Sirpb1	8.196495	6.896347	2.462541	0.002152756
TC1200001885.mm.2	Pygl	12.00677	10.71008	2.456646	0.01187955
TC0600001532.mm.2	Clec12a	12.01678	10.72151	2.454221	0.03852912
TC1400002346.mm.2	Dmtn	5.529379	4.237816	2.447931	3.48E-05
TC0400002116.mm.2	Vmn1r3	5.334334	4.047278	2.440296	0.007272631
TC1500001656.mm.2	Sla	11.61812	10.3535	2.402642	0.000450693
TC0900000216.mm.2	Zfp809	15.23423	13.97246	2.397905	0.007930629
TC0700000693.mm.2	1600014C10Ri	8.495101	7.233772	2.397164	0.006589063
TC0800002102.mm.2	Msr1	11.61295	10.35237	2.395921	0.01339706
TC0X00001658.mm.2	Ap1s2	11.47482	10.22237	2.382467	0.01706102
TC0600001083.mm.2	Fbln2	12.56249	11.31301	2.37755	0.0409409
TC0600001890.mm.2	Tfec	11.31922	10.07338	2.371565	0.01669895
TC1900000373.mm.2	Klf9	11.34527	10.1205	2.337193	0.004083266
TC0700000756.mm.2	Siglecf	9.62972	8.406481	2.334702	0.01053974
TC0200000651.mm.2	Ptgs1	10.49188	9.283736	2.310394	0.01700707
TC1500001887.mm.2	Npcd; Cbx6; N	10.63236	9.42575	2.307953	0.01214661
TC1500000787.mm.2	Parvg	7.838411	6.654267	2.272286	0.007307113
TC0500003452.mm.2	Agfg2	8.5171	7.335253	2.268671	0.01661564
TC0500003463.mm.2	Pilra	11.58845	10.41578	2.254282	0.03555674
TC0700000024.mm.2	Leng8	12.61872	11.45434	2.241367	0.04306053
TC0900001511.mm.2	Bcl2a1c	8.253597	7.096037	2.230799	0.01453144
TC0200000740.mm.2	Kynu	7.58778	6.433701	2.225423	0.02884751
TC1400002031.mm.2	Rab2b	7.681511	6.529924	2.221581	0.003559063

TC1300000054.mm.2	Dip2c	8.930294	7.782674	2.21548	0.006397373
TC0500003205.mm.2	Tctn1	9.510888	8.364086	2.214226	0.009584217
TC0500003518.mm.2	Card11	5.63959	4.497224	2.207427	0.02492639
TC0600001203.mm.2	Bhlhe40	15.412	14.2812	2.189809	0.01736773
TC0400000726.mm.2	6330416G13Ri	8.836527	7.712403	2.179692	0.01269329
TC0700003594.mm.2	Furin	12.46992	11.35375	2.167712	0.02386142
TC0900001987.mm.2	Fli1	10.92266	9.808608	2.164526	0.02161377
TC1300000522.mm.2	Mylip	10.83592	9.722563	2.163483	0.000876482
TC1700001827.mm.2	Sik1	9.382764	8.274295	2.156167	0.03337354
TC0700000810.mm.2	Napsa	9.607574	8.501823	2.152108	0.0247763
TC0800001256.mm.2	Tango6	8.392529	7.288565	2.149446	0.002708372
TC0500000466.mm.2	Bst1	10.38492	9.283374	2.145851	0.009653044
TC0400001990.mm.2	Errfi1	14.41005	13.32163	2.12641	0.009012803
TC1700001699.mm.2	Dusp1	13.04168	11.96	2.116504	0.005457788
TC0800000114.mm.2	F7	10.91544	9.845357	2.099552	0.005174844
TC0200001959.mm.2	Sirpa	15.22815	14.15818	2.099388	0.006042662
TC0300003226.mm.2	Gm5150	8.117517	7.052055	2.092839	0.0145374
TC1300000365.mm.2	Serpinb9b	6.124835	5.06284	2.087818	0.002476342
TC0100002702.mm.2	Slc16a14	6.182132	5.127221	2.07759	0.0248602
TC1100004035.mm.2	Fam104a	7.639903	6.586854	2.074911	0.02533445
TC0300002482.mm.2	Ecm1; Mir7014	16.45604	15.4094	2.065717	0.03867629
TC1900001253.mm.2	Gda	16.38393	15.34095	2.060488	0.00050681
TC1100004173.mm.2	Cbx4	8.844441	7.810769	2.047229	0.005069064
TC0500000378.mm.2	Sh3bp2	10.43785	9.404959	2.046125	0.03433699
TC0700002500.mm.2	Bcam	10.91226	9.883463	2.040321	0.002883543
TC1400002273.mm.2	Gm4583	7.482574	6.456134	2.03699	0.003011674
TC0500001179.mm.2	Tpst2	9.069002	8.046469	2.031483	0.007282797
TC1000000759.mm.2	Itgb2	12.28982	11.2683	2.030058	0.03084445
TC0X00001069.mm.2	Tlr13	11.2533	10.2378	2.021609	0.01509292
TC0800000271.mm.2	Thsd1	7.254892	6.241119	2.019185	0.02331305
TC0100001479.mm.2	Vamp4	10.80353	9.790413	2.018271	0.02839006
TC0300000767.mm.2	Kcnn3	6.619415	5.608858	2.014689	0.04137158
TC1900000531.mm.2	Hhex	7.440954	6.431612	2.012994	0.001970085
TC1400002836.mm.2	Dok2	11.91048	10.90476	2.007943	0.01926934
TC1500000785.mm.2	Parvb	6.424083	5.419438	2.006448	0.02040687
TC1000002408.mm.2	Pcbp3	7.834416	6.833653	2.001058	0.01697198
TC0300002460.mm.2	Zfp687	9.503313	8.505235	1.997337	0.0157535
TC1800000689.mm.2	Pmaip1	10.90201	9.905529	1.995122	0.04846003
TC0600003428.mm.2	Bhlhe41	10.33591	9.341343	1.992477	0.02999634
TC1900000646.mm.2	Scd2; Mir5114	16.37721	15.38571	1.988258	0.002478061
TC1000002484.mm.2	Med16	8.360078	7.372602	1.982713	0.007995022
TC0700000403.mm.2	Arhgef1	10.57681	9.590992	1.98044	0.03050926
TC0100001568.mm.2	Sh2d1b1	10.20496	9.219707	1.979658	0.03561093
TC0200002538.mm.2	Pcif1	9.28342	8.3034	1.972491	0.01351809
TC0700004487.mm.2	Adam8	9.357466	8.387583	1.958682	0.000128449
TC0100000292.mm.2	Il1r2	7.607447	6.64764	1.94505	0.007056827
TC0200003332.mm.2	Mvb12b	9.218525	8.260267	1.942962	0.0386054
TC1100002249.mm.2	Plek	12.76583	11.81004	1.939644	0.01932385
TC1900000888.mm.2	Tcirg1	10.2946	9.340202	1.937775	0.02112045

TC0200004913.mm.2	Bcl2l1	11.91153	10.97043	1.919995	0.02755121
TC1100001276.mm.2	Acaca	9.732684	8.794061	1.916698	0.003641177
TC1900000600.mm.2	Pgam1	10.63836	9.705197	1.909458	0.003954177
TC1400002714.mm.2	Abcc4	12.64556	11.71243	1.909408	0.01909002
TC1400000986.mm.2	Tnfrsf10b	10.968	10.03729	1.90621	0.01860442
TC1900001030.mm.2	Fermt3	8.421018	7.49256	1.90324	0.03505828
TC1900000441.mm.2	Cd274	8.622024	7.695985	1.900052	0.01236672
TC1300000464.mm.2	Sycp2l; LOC10	5.008516	4.084455	1.897449	0.01283624
TC0400002198.mm.2	Plekhf2	10.39916	9.476307	1.895866	0.000717465
TC0700004539.mm.2	Pidd1	6.246559	5.327228	1.891238	0.02663386
TC1400000742.mm.2	Abhd4	12.73362	11.81613	1.888826	0.000972005
TC1000001466.mm.2	Grip1	5.849504	4.933905	1.886353	0.03461423
TC1100002966.mm.2	Hs3st3b1	7.360095	6.451539	1.877166	0.003634537
TC0300000709.mm.2	Isg20l2	7.558307	6.656367	1.868576	0.01606058
TC1900001027.mm.2	Vegfb	5.598519	4.69715	1.867838	0.01659113
TC0500000347.mm.2	Depdc5	9.924784	9.028803	1.860875	0.03027815
TC0X00001024.mm.2	Chic1	6.032443	5.138864	1.857779	0.01658009
TC0X00001569.mm.2	Samt4	4.673404	3.782371	1.854504	0.000158469
TC1500000782.mm.2	Pnpla3	4.493755	3.607096	1.84889	0.04019393
TC0400002092.mm.2	Ccnl2	12.10632	11.22091	1.8473	0.01615518
TC0900000340.mm.2	Pate2	5.065884	4.18412	1.842626	0.000497992
TC0100002887.mm.2	Rnf152	8.855002	7.978045	1.836499	0.01394489
TC1300001593.mm.2	Vmn1r205; Vm	4.018424	3.142032	1.835779	0.000217366
TC0300001109.mm.2	Kcna3	6.969051	6.09803	1.828957	0.009207372
TC1000002510.mm.2	Mob3a	9.324166	8.455109	1.826469	0.04070602
TC0100001994.mm.2	Sbspon	5.654502	4.787379	1.824021	0.01574786
TC0700002611.mm.2	Cd177	6.603062	5.738508	1.820777	0.007744729
TC1100003086.mm.2	Slc16a13	6.618989	5.75701	1.817529	0.01066003
TC1900000443.mm.2	Ric1	10.77218	9.913193	1.81376	0.002245226
TC1100001887.mm.2	Tmem104	8.606038	7.750788	1.809072	0.03085643
TC1300000164.mm.2	Zscan12	6.852773	5.998854	1.807404	0.02740401
TC1900001217.mm.2	Gcnt1	10.41346	9.562538	1.80365	0.00574353
TC0800000983.mm.2	Man2b1	12.02931	11.18696	1.792958	0.003217345
TC0X00001120.mm.2	Tex16	4.645226	3.803949	1.791635	0.004193653
TC0900001902.mm.2	1810064F22Ri	7.411649	6.57111	1.79072	0.01779763
TC0200000485.mm.2	Gm10134	8.519485	7.679548	1.789971	0.0330218
TC0200000352.mm.2	Apbb1ip	10.24309	9.405096	1.787567	0.02590153
TC0700004591.mm.2	Tnfrsf26	9.280508	8.442812	1.787195	0.01847462
TC1300002609.mm.2	Rnf180	7.738034	6.901617	1.785609	0.02124094
TC0700001631.mm.2	Ampd3	11.69186	10.85613	1.784755	0.04273871
TC1700000917.mm.2	Ccnd3	11.93895	11.10612	1.781167	0.001302257
TC0700000946.mm.2	Atp10a	9.315825	8.484529	1.779282	0.01041407
TC1600000199.mm.2	Top3b	11.20502	10.37508	1.777604	0.01225516
TC1900000664.mm.2	Sfxn3	10.07831	9.252889	1.772047	0.008760688
TC1500001238.mm.2	Cdh6	13.24864	12.42467	1.770271	0.02530802
TC1400000281.mm.2	Erc2	7.553102	6.731266	1.767653	0.005664425
TC1100004203.mm.2	Faap100	8.205469	7.383778	1.767476	0.03740218
TC0300002594.mm.2	Cd101	6.186746	5.365819	1.766541	0.001082887
TC1800000490.mm.2	Arl14epl	4.701753	3.886309	1.759839	0.001274406

TC0600001092.mm.2	Slc6a6	13.6864	12.87161	1.759053	0.02370743
TC0900001408.mm.2	Lamb2	8.892312	8.077743	1.758773	0.02696179
TC0X00001566.mm.2	Spin2c	6.787881	5.974389	1.75746	0.03069424
TC0200001735.mm.2	Eif2ak4	9.494929	8.684337	1.753932	0.04593167
TC0300002450.mm.2	Tuft1	9.297611	8.4878	1.752982	0.02260903
TC1500002295.mm.2	Npff; Atf7	11.8107	11.00184	1.751818	0.01027293
TC0200004548.mm.2	Itpripl1	10.90106	10.09238	1.751614	0.002704265
TC0600001401.mm.2	Clec4a2	9.103983	8.296084	1.75066	0.02344876
TC0400004062.mm.2	Car6	6.466085	5.660928	1.747336	0.006124646
TC0300003029.mm.2	Gm4862	5.232381	4.427718	1.746738	0.03248051
TC0900001142.mm.2	Bcl2a1a	12.39874	11.59435	1.746405	0.0493545
TC0100003272.mm.2	Lamc2	13.17984	12.3756	1.746224	0.01855982
TC0900002516.mm.2	Zfp609	11.46281	10.65915	1.745528	0.04183337
TC1600001320.mm.2	Cdc45	6.044287	5.240837	1.74527	0.03779988
TC1100000873.mm.2	Pik3r6	7.122828	6.321236	1.743024	0.04144064
TC0800001608.mm.2	Insr	13.45301	12.65382	1.740133	0.04614504
TC0900000533.mm.2	Amica1	9.242658	8.444328	1.739086	0.03263031
TC0800002307.mm.2	Tll1	6.615808	5.820957	1.734898	0.04673092
TC0300000631.mm.2	Pdgfc	11.67564	10.8829	1.732361	0.03764098
TC1400002223.mm.2	Mtmr9	9.670552	8.879745	1.730042	0.004405723
TC1800001437.mm.2	Adrb2	8.197405	7.406742	1.729869	0.02455963
TC1100002084.mm.2	Limk2	7.564663	6.779839	1.722883	0.03906358
TC1000000820.mm.2	Ptbp1	13.55403	12.77257	1.718869	0.009923655
TC0700003593.mm.2	Fes	5.9453	5.164064	1.718602	0.02912526
TC0900002320.mm.2	Dmxl2	10.38905	9.608718	1.717524	0.009566547
TC0100001451.mm.2	4930523C07Ri	7.638491	6.860067	1.715256	0.01346179
TC0700001624.mm.2	Wee1	9.488561	8.714481	1.710099	0.005098955
TC0800000707.mm.2	Lpl	11.5173	10.74719	1.705409	0.03282718
TC1000003032.mm.2	Msrb3	10.74587	9.978798	1.701816	0.04857179
TC0100002580.mm.2	Cxcr1	3.955227	3.188624	1.701258	0.04037884
TC1400000195.mm.2	Kat6b	10.92473	10.15946	1.699685	0.008816498
TC1300000687.mm.2	Slc25a48	5.382151	4.616964	1.699591	0.01981233
TC1000002420.mm.2	Adarb1	10.12993	9.366068	1.698025	0.001978414
TC1000000385.mm.2	Ostm1	10.16613	9.402886	1.697303	0.02067677
TC1200000788.mm.2	Rbm25	11.24475	10.48182	1.696938	0.001459599
TC1500000433.mm.2	Nsmce2	10.7858	10.02287	1.696936	0.01658192
TC0500003050.mm.2	Selplg	12.49441	11.7336	1.694439	0.01694652
TC0200002894.mm.2	Celf2	12.06487	11.30455	1.693856	0.01472678
TC1200001527.mm.2	9030624G23Ri	9.002363	8.243531	1.69212	0.008872245
TC0700002277.mm.2	Vmn2r39	6.011096	5.25589	1.687872	0.03450757
TC1600001343.mm.2	Abcc5	11.52522	10.77804	1.678502	0.02373903
TC1200000151.mm.2	B430203G13R	6.488658	5.742801	1.67697	0.02834912
TC1100002773.mm.2	Ccdc69	4.742895	3.997345	1.676612	0.001772213
TC0200000240.mm.2	Vim	15.7371	14.99231	1.675725	0.0310684
TC1500000865.mm.2	Ppp6r2	7.298608	6.554636	1.67478	0.02767731
TC0800000604.mm.2	Gm5927	4.273714	3.529896	1.674601	0.02471977
TC0500000384.mm.2	Grk4	6.603263	5.862264	1.671332	0.007237018
TC1900001493.mm.2	Tctn3	6.775688	6.03516	1.670787	0.003663088
TC1900000981.mm.2	Sssca1; Fam89	7.827328	7.087759	1.669676	0.03009457

TC0700003810.mm.2	Rnf169	12.56344	11.82993	1.662675	0.03641494
TC0200002363.mm.2	Map1lc3a	7.864007	7.131937	1.661022	0.02669977
TC0100001932.mm.2	Sntg1	6.213842	5.482705	1.659947	0.02782925
TC1100003839.mm.2	Itga2b	6.953137	6.222731	1.659107	0.04538804
TC1100004115.mm.2	Foxj1; Rnf157	5.243716	4.516972	1.654899	0.01021142
TC1700000415.mm.2	Ergic1	12.82559	12.10067	1.652809	0.02577416
TC0500002045.mm.2	5031425E22Ri	7.234487	6.509711	1.652644	0.03746625
TC0600002427.mm.2	Tacstd2	7.004669	6.282559	1.649592	0.01925781
TC0500001632.mm.2	Sap25; Lrch4; (	13.34715	12.62522	1.649382	0.01892998
TC1000003163.mm.2	Tmem198b	8.277016	7.556076	1.648255	0.03858379
TC0400000811.mm.2	Lurap1l	11.41498	10.69783	1.643931	0.002721465
TC1100002975.mm.2	Arhgap44	6.615246	5.901693	1.639838	0.01369841
TC1900000043.mm.2	Rbm4b	10.70088	9.98815	1.638903	0.03208056
TC1100001935.mm.2	Sphk1	7.30204	6.591616	1.636285	0.01533303
TC0400003829.mm.2	Pla2g2f	4.904096	4.194736	1.635078	0.03583524
TC0300000211.mm.2	Cldn11	4.624561	3.917371	1.632621	0.00322193
TC1100001442.mm.2	Lrrc59	14.14494	13.43884	1.631384	0.03669848
TC0300002734.mm.2	Cyb561d1	7.420011	6.715323	1.629791	0.004693012
TC0200002830.mm.2	Prpf6	11.2639	10.56103	1.627738	0.04790073
TC1100002334.mm.2	Ehbp1	10.10054	9.398381	1.626936	0.009443012
TC0700002057.mm.2	Ap2a2	11.72415	11.02329	1.625473	0.004610327
TC1800000378.mm.2	Pcdhb21; Pcdh	5.644163	4.949763	1.618211	0.002032482
TC1900001729.mm.2	Shtn1	11.5869	10.89396	1.616568	0.03113756
TC1100002114.mm.2	Mtmr3	10.23355	9.541554	1.615522	0.0298526
TC0800000196.mm.2	4930467E23Ri	5.037999	4.346923	1.614486	0.02524456
TC1700000332.mm.2	Prss27	4.948803	4.257935	1.614254	0.01638761
TC0500001880.mm.2	V1rg10; Vmn1r	5.843971	5.153271	1.614066	0.04504406
TC1100002426.mm.2	Eml6	4.849992	4.163163	1.609741	0.04822959
TC1100002176.mm.2	Myo1g	9.253807	8.567186	1.609508	0.03976398
TC0600003020.mm.2	Erc1	10.47948	9.793081	1.609256	0.00283078
TC1100004236.mm.2	Csnk1d	15.31421	14.62953	1.607349	0.02946283
TC0600001347.mm.2	Il17ra	9.600607	8.917078	1.606064	0.03538197
TC1800001666.mm.2	Atp9b	11.1446	10.46392	1.602891	0.0262301
TC0400002321.mm.2	Fut9	7.109411	6.429449	1.602098	0.00794562
TC0900001903.mm.2	Zfp599	8.294622	7.614814	1.601927	0.000199094
TC0X00002771.mm.2	Nap1l2	3.577901	2.898317	1.601678	0.006784049
TC0300003173.mm.2	Acadm	8.845016	8.169528	1.597136	0.01033034
TC0700001684.mm.2	Nucb2	11.38716	10.71317	1.595484	0.01875984
TC0400001356.mm.2	Cited4	4.941072	4.270143	1.592097	0.04047254
TC0100003826.mm.2	Rcor3	10.07832	9.409321	1.589971	0.006146106
TC1200001523.mm.2	Gm10330; LOC	8.230091	7.561607	1.589403	0.02379937
TC1100001361.mm.2	Vezf1	11.48276	10.81445	1.5892	0.02297434
TC0600001449.mm.2	Iffo1	6.01475	5.347776	1.587739	0.02596843
TC1500002313.mm.2	Nfe2	4.72282	4.057007	1.586461	0.04684863
TC0X00003327.mm.2	Car5b	10.75575	10.09014	1.586239	0.04312196
TC0500002945.mm.2	Mfsd7a	6.175138	5.512977	1.582451	0.02288194
TC1400002186.mm.2	Ebpl	7.799775	7.138077	1.581943	0.01536527
TC1800000994.mm.2	Npc1	10.44218	9.781761	1.580545	0.02903662
TC1900001592.mm.2	9130011E15Ri	7.330345	6.671346	1.578987	0.04984093

TC0200002426.mm.2	Lbp	14.13595	13.47937	1.576342	0.03354414
TC0900000402.mm.2	Olfr905	4.367802	3.711246	1.576315	0.04716307
TC1600001974.mm.2	Adamts1	9.149403	8.493649	1.575439	0.02980294
TC0700000131.mm.2	Vmn1r79	4.508678	3.85338	1.574941	0.03900918
TC0600000578.mm.2	Malsu1	7.513352	6.858918	1.573998	0.006677411
TC0500003051.mm.2	Coro1c	13.55609	12.90509	1.570261	0.009942024
TC0X00000670.mm.2	Zfp185	5.097204	4.446403	1.57004	0.01948595
TC0200000552.mm.2	Fubp3	8.600662	7.950462	1.569385	0.0311707
TC0200001514.mm.2	Traf6	9.567619	8.920597	1.565932	0.0315232
TC1700001621.mm.2	1600002H07Ri	7.608952	6.962478	1.565337	0.01158626
TC1400000477.mm.2	Ptger2	5.771997	5.125551	1.565308	0.04642651
TC0X00003098.mm.2	Acsl4	12.19708	11.55203	1.563789	0.0230261
TC1200001933.mm.2	Ppp2r5e	9.248205	8.603704	1.563198	0.004947968
TC0600000236.mm.2	Irf5	6.70254	6.059079	1.562073	0.04661952
TC0500001006.mm.2	Ptpn13	11.30492	10.66219	1.561285	0.009763992
TC0500001222.mm.2	Myo1h	5.084107	4.444537	1.557865	0.01210945
TC0100001164.mm.2	Fcamr	4.837734	4.199263	1.556679	0.02023171
TC1100003807.mm.2	Vat1	11.7389	11.10259	1.554356	0.002598055
TC0600000053.mm.2	Mios	5.11813	4.483088	1.552983	0.02589101
TC0400002945.mm.2	Ttc39b	11.5028	10.86849	1.552195	0.03210963
TC0900002887.mm.2	Zbtb38; E0300	14.95095	14.31783	1.550912	0.02341864
TC0500003054.mm.2	Ssh1	7.526179	6.894067	1.549833	0.006163236
TC0100001943.mm.2	Mybl1	6.135518	5.503655	1.549564	0.01641691
TC0400003530.mm.2	Thrap3	13.57279	12.94118	1.54929	0.003009561
TC1700000492.mm.2	Kctd20	9.607187	8.976318	1.548497	0.01767163
TC0400000015.mm.2	Lyn	13.45602	12.82544	1.548183	0.01848476
TC1700000351.mm.2	Msrb1	10.29476	9.664202	1.548163	0.01148677
TC1100004217.mm.2	Arhgdia	15.87835	15.25097	1.544758	0.01035185
TC1500000659.mm.2	Nol12	7.676515	7.050147	1.543674	0.003143581
TC1700000255.mm.2	Ppp2r1a	14.24513	13.61942	1.542962	0.009164552
TC1500002037.mm.2	Hdac10	4.327038	3.701732	1.542538	0.01979815
TC1900000777.mm.2	Vti1a	9.962148	9.337389	1.541953	0.04307438
TC0200002315.mm.2	Asxl1	10.23085	9.606133	1.541905	0.02391963
TC1800000249.mm.2	Lims2	4.665359	4.041026	1.541497	0.005665138
TC1600001191.mm.2	Emp2	9.778012	9.154614	1.5405	0.03891686
TC1600001464.mm.2	Tmem44	4.815571	4.192579	1.540066	0.02017592
TC0300003214.mm.2	Scamp3	9.419367	8.796542	1.539887	0.01099448
TC1800001577.mm.2	Smad4	12.70321	12.08064	1.539609	0.03504848
TC0700003690.mm.2	Olfr308	3.309618	2.688054	1.538543	0.003990223
TC0100003865.mm.2	Armc9	6.962808	6.343197	1.536461	0.01509195
TC1000001286.mm.2	Osbpl8	8.926553	8.307011	1.536388	0.006439165
TC0200003360.mm.2	Rab14	13.63504	13.01567	1.536199	0.008692329
TC0700000473.mm.2	Zfp626	8.554362	7.936445	1.534658	0.04571453
TC1200002083.mm.2	Arel1	15.51544	14.89815	1.533991	0.02204504
TC0700004612.mm.2	Tpcn2	8.56696	7.950122	1.533511	0.0333522
TC1200002115.mm.2	Pomt2	7.892033	7.275414	1.533278	0.01478955
TC1400000507.mm.2	Samd4	9.511415	8.895645	1.532375	0.006389158
TC0400002849.mm.2	Megf9	7.717763	7.10204	1.532327	0.001159472
TC1200000279.mm.2	Nampt	11.18964	10.57403	1.5322	0.007300625

TC1800000834.mm.2	Pstpip2	7.224673	6.609093	1.532174	0.0162041
TC0200004415.mm.2	Bmf	8.07194	7.457038	1.531455	0.03614572
TC0400003361.mm.2	Plk3	7.349925	6.73777	1.52854	0.01880024
TC0800000207.mm.2	Gm20946	3.57216	2.960251	1.52828	0.03291375
TC0100001631.mm.2	Aim2	9.493689	8.882614	1.527397	0.02982991
TC1900001617.mm.2	Nt5c2	12.0134	11.40645	1.523039	0.01319365
TC0800001694.mm.2	Irs2	9.163069	8.556411	1.522728	0.0204561
TC0700000596.mm.2	Gm9140	4.900375	4.293717	1.522728	0.03413735
TC1500000566.mm.2	Tigd5	4.472208	3.866331	1.521903	0.02264938
TC1100001773.mm.2	Map3k3	8.737489	8.131684	1.521826	0.02214286
TC0600002975.mm.2	Alox5	8.207174	7.601597	1.521587	0.04907082
TC0400002444.mm.2	Aptx	7.772779	7.168388	1.520336	0.02336763
TC0800000449.mm.2	6430573F11Ri	4.056816	3.453283	1.519433	0.041453
TC0400003821.mm.2	Pink1; Mir7019	4.716717	4.113529	1.51907	0.03132895
TC1700001838.mm.2	Akap8	10.08741	9.485054	1.518192	0.01831114
TC1200000175.mm.2	LOC10524476	8.685402	8.084869	1.516276	0.04561123
TC0600001344.mm.2	Kdm5a	9.741814	9.141528	1.516017	0.003829826
TC0200000293.mm.2	Mllt10	11.39385	10.79482	1.514698	0.04951685
TC0X00003400.mm.2	Gm6377	6.961593	6.363965	1.513226	0.02862499
TC0100003700.mm.2	Enah	9.787554	9.191095	1.512	0.04779286
TC1600001400.mm.2	Bcl6	8.048377	7.452006	1.511909	0.02160098
TC1400000438.mm.2	Gm7233	3.832501	3.23894	1.508966	0.03038838
TC0800001855.mm.2	Defb9	4.278571	3.685026	1.50895	0.02244538
TC0500003368.mm.2	Ncf1	13.14995	12.55718	1.508146	0.01811676
TC1100002374.mm.2	Rel	10.7059	10.11377	1.50747	0.001461357
TC0600001970.mm.2	Rbm28	6.774928	6.185367	1.504789	0.0175257
TC1400001223.mm.2	Gm6234	3.922548	3.333718	1.504027	0.0217041
TC1400001224.mm.2	Gm4666	3.922548	3.333718	1.504027	0.0217041
TC0X00002524.mm.2	Gm8787	6.479967	5.893523	1.501541	0.01597871
TC0600001769.mm.2	2810474O19Ri	14.09771	13.51137	1.501433	0.002629245
TC0600003163.mm.2	Kcna1	3.590481	4.177062	-1.501683	0.02229056
TC0700001955.mm.2	Nps	2.942469	3.530248	-1.502932	0.001922821
TC1700000398.mm.2	D630044L22Ri	3.212648	3.800535	-1.503044	0.01071951
TC0X00002999.mm.2	Armcx6	4.18888	4.776956	-1.50324	0.04398828
TC0200003685.mm.2	Gm26727	3.677615	4.266529	-1.504114	0.02081843
TC0900001349.mm.2	Pcbp4	7.416625	8.005643	-1.504223	0.02298819
TC1700002337.mm.2	Rfx2	4.805951	5.396864	-1.5062	0.01584357
TC1100002769.mm.2	Lyrm7	6.088838	6.681304	-1.507822	0.0378125
TC0Y00000266.mm.2	Gm20822; Gm	5.258172	5.850946	-1.508145	0.02493507
TC0Y00000274.mm.2	Gm20822	5.258172	5.850946	-1.508145	0.02493507
TC0X00002920.mm.2	Gm17521	4.341415	4.934868	-1.508854	0.01999445
TC1200001898.mm.2	Gpr135	2.991717	3.585489	-1.509188	0.04256641
TC0700001397.mm.2	Olfr521	3.36034	3.956423	-1.511607	0.005070369
TC0X00003404.mm.2	Gm15080; Ott;	4.67442	5.270745	-1.51186	0.02684862
TC0700003029.mm.2	Abcc6	4.608287	5.208785	-1.51624	0.008570202
TC1100000690.mm.2	1810065E05Ri	4.770434	5.371322	-1.51665	0.02291463
TC0900002542.mm.2	Tln2	6.378799	6.982519	-1.51963	0.03801351
TC0500003531.mm.2	Papolb	4.499567	5.103459	-1.519811	0.003714168
TC1400000425.mm.2	Gm7995	5.376247	5.980968	-1.520685	0.01397699

TC1400002310.mm.2	Slc25a37	7.665995	8.272023	-1.522063	0.03473871
TC0500002167.mm.2	Ost4	14.3938	15.00216	-1.524524	0.03459486
TC0100000268.mm.2	Mrpl30	7.63036	8.238758	-1.524566	0.01178227
TC1000002727.mm.2	Mrpl42	11.09258	11.70276	-1.526448	0.02824941
TC0900002972.mm.2	Tmem108	4.277802	4.890056	-1.528646	0.01018307
TC0800000294.mm.2	Nkx6-3	4.578384	5.192412	-1.530526	0.02566139
TC0200003389.mm.2	Olfr358	2.818931	3.433201	-1.530783	0.01723674
TC0200000543.mm.2	Prrx2	5.412742	6.031785	-1.535855	0.03980257
TC1300000589.mm.2	Gm904	5.698482	6.317536	-1.535868	0.01477367
TC1600001281.mm.2	Ccdc116	5.121562	5.743536	-1.538979	0.03621313
TC0500002738.mm.2	Btc	10.38112	11.00336	-1.539268	0.03307256
TC1800000564.mm.2	Gramd3	9.292442	9.91689	-1.541621	0.0445918
TC0600001391.mm.2	Nanog	5.165218	5.792066	-1.544188	0.01058405
TC0Y00000395.mm.2	Gm21943	2.800157	3.427243	-1.544443	0.009005794
TC0800003186.mm.2	Pcnxl2	3.806512	4.433779	-1.544636	0.02237069
TC0200005475.mm.2	Dusp19	7.393044	8.021889	-1.546327	0.02569976
TC1400000838.mm.2	4930563I02Rik	3.084133	3.713013	-1.546364	0.0107314
TC1000001584.mm.2	Atp5b	14.76085	15.39105	-1.547788	0.04013185
TC0100001730.mm.2	Cnih4	12.05417	12.68652	-1.550088	0.008484279
TC1300001777.mm.2	Serpinb6a	12.78064	13.41974	-1.557364	0.0287322
TC0300002553.mm.2	Hsd3b4; Gm10	3.472338	4.112196	-1.558175	0.03208245
TC0900002382.mm.2	Arid3b	5.076498	5.719462	-1.561534	0.004682996
TC1100002022.mm.2	Bahcc1	3.312482	3.955717	-1.561827	0.002261018
TC1500002040.mm.2	Plxnb2	11.63312	12.27774	-1.563336	0.03131647
TC0800002376.mm.2	Ndufa13	10.54099	11.18581	-1.563535	0.00875907
TC0200003873.mm.2	Tmx2	9.837976	10.48581	-1.56681	0.04679794
TC0700000766.mm.2	Zfp819	4.874263	5.52221	-1.566936	0.001797663
TC1100002595.mm.2	Gm12166	9.008178	9.65727	-1.568181	0.02501851
TC1100003795.mm.2	Plekhh3	5.86956	6.518677	-1.568208	0.001712781
TC0500002932.mm.2	Gfi1	5.713576	6.362694	-1.568208	0.03689993
TC1800000827.mm.2	St8sia5	3.251389	3.900849	-1.568581	0.003752884
TSUnmapped00000054.	Mrgpra8	3.397437	4.048665	-1.570504	0.003678372
TC1500001004.mm.2	Prph	3.48018	4.134196	-1.573543	0.03148806
TC0500001230.mm.2	Tchp	12.47801	13.13216	-1.573682	0.04027488
TC1800000589.mm.2	Slc27a6	4.367361	5.023469	-1.575827	0.009283608
TC1700001010.mm.2	Safb	8.83615	9.492443	-1.576028	0.02547842
TC0500001170.mm.2	Gm26897	6.716477	7.373522	-1.576849	0.008715406
TC1600001080.mm.2	Sh3bgr	5.327612	5.985387	-1.577648	0.007818989
TC0800001079.mm.2	Capns2	3.809416	4.4672	-1.577657	0.03506361
TC0700002511.mm.2	Gm16451; Gm	3.362514	4.020488	-1.577866	0.04496669
TC1700002534.mm.2	Dpy30	8.853498	9.512659	-1.579164	0.03881957
TC0400003218.mm.2	Lrrc42	7.2542	7.915613	-1.581631	0.02697015
TC1000000536.mm.2	Gm10322	7.729686	8.391614	-1.582195	0.04970998
TC1200000809.mm.2	Coq6	5.128103	5.792052	-1.584414	0.03635538
TC1600001561.mm.2	Csta1	3.540382	4.204699	-1.584818	0.01071832
TC1000000795.mm.2	Olfr1355	3.74307	4.413095	-1.591101	0.02160496
TC1900000191.mm.2	Tmem258	11.272	11.94308	-1.592262	0.01214939
TC0X00000741.mm.2	Fundc2	8.93854	9.610365	-1.593086	0.03955567
TC1400000581.mm.2	Rnase2b	3.367324	4.04079	-1.5949	0.01091642

TC1300000366.mm.2	Serpinb1b	7.947472	8.621151	-1.595136	0.01055916
TC0900000562.mm.2	Zpr1	9.15605	9.83055	-1.596045	0.0378159
TC1400000744.mm.2	Mrpl52	7.113743	7.788677	-1.596524	0.01449593
TC1700000382.mm.2	Gng13	4.478094	5.153247	-1.596766	0.009862409
TC0700001347.mm.2	Kctd21	7.673991	8.353969	-1.602115	0.0168354
TC0100003804.mm.2	Atf3	8.705334	9.385896	-1.602764	0.03133608
TC0300001475.mm.2	Odf2l	9.409985	10.09557	-1.60835	0.006199214
TC0300000116.mm.2	Cypt12	2.944083	3.632638	-1.611669	0.02644425
TC0700004652.mm.2	Snrpn; Snurf	6.64092	7.333334	-1.615986	0.04779709
TC0700002296.mm.2	Vmn2r46	5.650084	6.344429	-1.618149	0.006525262
TC0600003286.mm.2	Tas2r122	3.170376	3.868238	-1.622099	0.01325356
TC0900001290.mm.2	Anapc13	10.23594	10.94194	-1.631274	0.005201765
TC1500002316.mm.2	Gpr84	4.870175	5.576978	-1.632183	0.01152946
TC1900001764.mm.2	Gm21060	4.33469	5.043459	-1.634409	0.02511439
TC1900000102.mm.2	Batf2	3.772985	4.481778	-1.634435	0.003223086
TC1400001980.mm.2	Olfr733	3.313356	4.023631	-1.636116	0.007022938
TC1500000520.mm.2	Dennd3	4.615838	5.326734	-1.63682	0.02613042
TC1200002129.mm.2	LOC10086220(	3.424799	4.136456	-1.637685	0.0427137
TC0400000880.mm.2	Focad	5.700451	6.41227	-1.637868	0.02210614
TC0600001751.mm.2	Far2	5.110254	5.824773	-1.640936	0.02572951
TC1400001851.mm.2	Gm8122	4.199137	4.915001	-1.642467	0.01133015
TC0400000169.mm.2	Ggh	11.5059	12.22332	-1.644234	0.01751754
TC0300002488.mm.2	Mrps21	7.804734	8.523013	-1.645218	0.0121321
TC1700000207.mm.2	Vmn2r90	3.707294	4.42706	-1.646915	0.01106006
TC0500002963.mm.2	Vmn2r12	3.86112	4.581054	-1.647107	0.006112394
TC1700002092.mm.2	Esp36	3.905534	4.626777	-1.648602	0.01213767
TC1700001909.mm.2	Btnl4	4.935976	5.657889	-1.649368	0.02696791
TC0100002840.mm.2	Pdcd1	4.369117	5.097306	-1.656558	0.0239797
TC0200002181.mm.2	6430503K07Ri	8.292167	9.021025	-1.657327	0.001348329
TC0500003166.mm.2	Sdsl	6.196063	6.926103	-1.658685	0.002373912
TC1100001480.mm.2	Gip	5.956703	6.687723	-1.659812	0.008618434
TC0500000844.mm.2	Cxcl3	7.115694	7.847577	-1.660805	0.04327978
TC0200002849.mm.2	Meig1	4.412054	5.146583	-1.663854	0.004412187
TC0800001518.mm.2	Spire2	6.160009	6.895627	-1.66511	0.04885392
TC1300001666.mm.2	Gmnn	5.94473	6.681362	-1.666281	0.03119187
TC1400002863.mm.2	Ipo4	6.852138	7.588971	-1.666513	0.006737867
TC0700000988.mm.2	Pcsk6	7.910994	8.648164	-1.666903	0.04124083
TC1300001378.mm.2	Akr1c12	8.175999	8.914702	-1.668676	0.01533721
TC0X00000075.mm.2	Slc35a2	7.644003	8.384637	-1.67091	0.01291427
TC0700002293.mm.2	Vmn2r47	4.34937	5.092684	-1.674016	0.03931469
TC0700002297.mm.2	Vmn2r47	4.34937	5.092684	-1.674016	0.03931469
TC1900001769.mm.2	Tmem179b	9.519997	10.26605	-1.677203	0.002296928
TC0800000179.mm.2	Defb14	3.142469	3.890455	-1.679447	0.001572452
TC0200002760.mm.2	Cdh26	3.23909	3.988864	-1.681529	0.02045294
TC1600000122.mm.2	Gm11172	7.927423	8.678731	-1.683318	0.000713214
TC0100003879.mm.2	Als2	7.618269	8.371955	-1.686095	0.003864039
TC0200001472.mm.2	Lrrc4c	3.827672	4.583066	-1.688092	0.003266057
TC0700004288.mm.2	Nupr1	11.84866	12.60815	-1.692894	0.01085264
TC1100003193.mm.2	Rap1gap2	7.219156	7.98032	-1.694858	0.02616905

TC0700003834.mm.2	Atg16l2	8.642738	9.405862	-1.697161	0.03392541
TSUnmapped00000015.	Nudt7	4.687139	5.450977	-1.698002	0.01241958
TC1000000192.mm.2	Vnn3	4.094034	4.857895	-1.698029	0.0380982
TC0500000907.mm.2	Gm6523	3.969737	4.733625	-1.698061	0.0183723
TC0500000910.mm.2	Gm7942	3.969737	4.733625	-1.698061	0.0183723
TC0X00002604.mm.2	4930415L06Ri	3.391098	4.159279	-1.703121	0.002960473
TC0200004042.mm.2	Olfr1221	3.750072	4.519925	-1.705095	0.0114764
TC1400000829.mm.2	Fgf9	6.165718	6.938023	-1.707996	0.03360906
TC0700002021.mm.2	Olfr541	3.748592	4.523637	-1.711243	0.02041805
TC0200001798.mm.2	Stard9	10.12957	10.90768	-1.714883	0.02685731
TC0300001252.mm.2	1810037I17Rik	10.48432	11.26453	-1.717378	0.04704878
TC0500001401.mm.2	P2rx4	11.41851	12.19942	-1.718213	0.02216824
TC0400001259.mm.2	Prdx1	17.15806	17.93979	-1.719191	0.04560451
TC0X00000625.mm.2	1700020N15Ri	5.595931	6.3778	-1.719357	0.01462647
TC0200003994.mm.2	Olfr1138	3.112798	3.898167	-1.723533	0.01093496
TC1700002766.mm.2	Tcp10b; Tcp10	4.833163	5.621099	-1.726603	0.01605807
TC1100003709.mm.2	Krt23	5.712132	6.502304	-1.729281	0.01380601
TC1900001172.mm.2	Olfr1446	3.587607	4.379583	-1.731444	0.04821515
TC0800001630.mm.2	Prr36	4.060801	4.853608	-1.732442	0.009042174
TC0900000387.mm.2	Olfr890	2.965546	3.76537	-1.740889	0.002045816
TC0400004119.mm.2	Prdm16	5.849651	6.649916	-1.741421	0.02222768
TC1700002308.mm.2	Stap2	9.103539	9.912039	-1.751389	0.01854561
TC1100000856.mm.2	Myh8	3.728001	4.541596	-1.757586	0.02397884
TC0Y00000487.mm.2	Gm20917	3.41577	4.229506	-1.757757	0.02067937
TC0200005235.mm.2	Gm11011	7.194122	8.007889	-1.757794	0.02751119
TC1200000331.mm.2	Meox2	3.715726	4.529688	-1.758032	0.009924942
TC1700000751.mm.2	Znrd1as	5.068305	5.883189	-1.759157	0.009658735
TC1500001183.mm.2	Slc1a3	4.000526	4.817769	-1.762035	0.000793626
TC0M00000011.mm.2	ND3	17.58015	18.4054	-1.771847	0.04845303
TC1400002795.mm.2	Gm8281	4.851136	5.684971	-1.782416	0.01407583
TC0700001466.mm.2	Olfr558	4.817441	5.66529	-1.799817	0.03680507
TC0600001659.mm.2	Gm11077	3.318896	4.168706	-1.802263	0.02264628
TC0100003532.mm.2	Tomm40l	4.583044	5.434072	-1.803786	0.01130808
TC0200002989.mm.2	St8sia6	7.753718	8.607088	-1.806717	0.01898495
TC0600001632.mm.2	Pde6h	5.464869	6.320349	-1.80936	0.000974485
TC1400000580.mm.2	Rnase6	9.491761	10.35264	-1.81614	0.01041094
TC0300002944.mm.2	Ostc	11.45784	12.31993	-1.817678	0.03956061
TC0400001631.mm.2	Trim63	3.857601	4.726561	-1.826345	0.003861827
TC1400002314.mm.2	Gm16867; Gm	5.259256	6.130537	-1.829286	0.000144699
TC0200000801.mm.2	Kif5c	6.621877	7.493205	-1.829346	0.01137915
TC0600002253.mm.2	Cycs	12.83368	13.70569	-1.83021	0.03774769
TC1900001123.mm.2	AW112010	7.453381	8.328736	-1.834461	0.0239761
TC0100002338.mm.2	Hecw2	5.395963	6.287428	-1.855059	0.005842188
TC0100002261.mm.2	Fhl2	9.241073	10.13868	-1.862968	0.03576917
TC0400000280.mm.2	Gabrr1	3.856723	4.761569	-1.872344	0.0160171
TC0200003118.mm.2	Cacna1b	5.729432	6.639755	-1.879466	0.04671606
TC0300002212.mm.2	Glrb	4.171959	5.094844	-1.895903	0.004277484
TC1800001375.mm.2	C330018D20R	6.842349	7.77196	-1.904762	0.02868274
TC0X00001146.mm.2	Ube2dnl1	3.594211	4.524644	-1.905848	0.03584361

TC0200002151.mm.2	Gm561	4.176263	5.110854	-1.911348	0.04021055
TC1500000037.mm.2	Fyb	7.644592	8.584583	-1.918516	0.01548528
TC0600000655.mm.2	Plekha8	6.978465	7.920335	-1.921016	0.001753091
TC0700002079.mm.2	Tnnt3	4.168401	5.114724	-1.926956	0.03937327
TC0500000093.mm.2	Sema3e	7.92218	8.868751	-1.927285	0.004185167
TC1700000305.mm.2	Vmn2r113	3.643611	4.604399	-1.946373	0.002734799
TC1500000827.mm.2	Trmu	4.47787	5.444455	-1.95421	0.0188109
TC1200001643.mm.2	Tspan13	11.92531	12.89639	-1.960302	0.008070444
TC1000002380.mm.2	Susd2	5.278618	6.263398	-1.97901	0.04505946
TC0200000966.mm.2	Xirp2	3.768721	4.778108	-2.013056	0.001363287
TC1100000887.mm.2	Slc25a35	7.801395	8.811587	-2.01418	0.03972023
TC0200004925.mm.2	Tspyl3	3.718997	4.729942	-2.015232	0.02451855
TC0500001139.mm.2	Pole	4.990436	6.003372	-2.018014	0.0322676
TC1200001686.mm.2	Stxbp6	7.941687	8.955125	-2.018716	0.04282502
TC0200001866.mm.2	Sema6d	7.905208	8.919647	-2.020117	0.04580865
TC1400001960.mm.2	1700011H14Ri	6.562911	7.582656	-2.027561	0.03427219
TC1900000317.mm.2	Rfk	12.66241	13.68589	-2.032816	0.006125835
TC0200002961.mm.2	Pfkfb3	9.513014	10.53668	-2.03308	0.004227845
TC1900000843.mm.2	Pnliprp2	6.584584	7.623534	-2.054731	0.02243764
TC0500000857.mm.2	Areg	9.68759	10.74301	-2.078319	0.01049453
TC0700002636.mm.2	Ceacam2	3.958545	5.046892	-2.126302	0.003484757
TC0100001656.mm.2	Exo1	4.288678	5.379914	-2.130564	0.04177837
TC0600002539.mm.2	Reg3g	13.40871	14.53932	-2.189523	0.03456607
TC0200001849.mm.2	Slc28a2	6.235012	7.367574	-2.192478	0.02881618
TC1000002645.mm.2	Chpt1	8.610624	9.744387	-2.194302	0.007396985
TC1600000560.mm.2	B4galt4	5.126369	6.283308	-2.229836	0.03028783
TC1700000402.mm.2	Tmem8	9.32916	10.49712	-2.246931	0.03496084
TC1400002150.mm.2	Ska3	4.460665	5.646802	-2.275426	0.02668089
TC0400000234.mm.2	Epha7	7.920888	9.111121	-2.281895	0.001249286
TC0400001878.mm.2	Zfp600	6.172093	7.363945	-2.284456	0.001318212
TC1800001274.mm.2	Spink1	7.51552	8.714923	-2.296445	0.00862072
TC0500003591.mm.2	Atp5j2	12.64429	13.86621	-2.33257	0.04234637
TC1400000575.mm.2	Pnp2	9.332105	10.57127	-2.360612	0.000540415
TC1100004124.mm.2	St6galnac2	8.091998	9.386328	-2.452631	0.009959325
TC0100003258.mm.2	1700025G04Ri	10.54189	11.83888	-2.457156	0.01713566
TC1000001085.mm.2	Slc5a8	5.624395	6.954847	-2.514816	0.005894863
TC0800002918.mm.2	Chst4	6.949739	8.303853	-2.556401	0.02371452
TC1400000569.mm.2	Parp2	6.300354	7.685436	-2.611868	0.000159942
TC0600002155.mm.2	Prss3	4.025801	5.430472	-2.647575	0.02916121
TC0200003130.mm.2	Noxa1	4.768693	6.193223	-2.68427	0.02571314
TC1000000674.mm.2	Slc16a9	5.914991	7.349231	-2.702397	0.02631794
TC1400000574.mm.2	Pnp	9.264094	10.69992	-2.705376	0.002356728
TC0200001746.mm.2	Phgr1	5.383633	6.858616	-2.779805	0.02763005
TC0700001280.mm.2	Sytl2	8.120367	9.64791	-2.882944	0.02745889
TC1300000635.mm.2	Cdhr2	5.476838	7.013467	-2.901159	0.02442911
TC0800001191.mm.2	Ces2a	6.373956	7.912542	-2.905096	0.03806939
TC1100000953.mm.2	Tm4sf5	4.197459	5.737724	-2.908479	0.009431495
TC1100000119.mm.2	Upp1	6.786834	8.340881	-2.936396	0.03584873
TC0500002698.mm.2	Sult1b1	6.296031	7.861728	-2.960204	0.02281964

TC1700002437.mm.2	Themis3	8.107457	9.68164	-2.977666	0.0359563
TC0700002635.mm.2	Ceacam1	13.49744	15.0719	-2.978249	0.009138842
TC0500003660.mm.2	Hsph1	9.21141	10.7958	-2.998806	0.0336887
TC1800000791.mm.2	Myo5b	7.152617	8.749289	-3.024449	0.002182789
TC1200001030.mm.2	Ifi27	7.299194	8.939256	-3.11679	0.0248397
TC1000001543.mm.2	Avil	8.438018	10.07817	-3.116978	0.04472391
TC1700000325.mm.2	Prss32	6.305021	7.946295	-3.119413	0.01386479
TC1400000530.mm.2	Tmem260	7.896893	9.607331	-3.272603	1.77E-05
TC0600001903.mm.2	Asz1	8.410056	10.16375	-3.372216	0.000473658
TC1400000968.mm.2	Stc1	5.985919	7.752145	-3.40163	1.79E-05
TC0400002174.mm.2	Car8	7.849981	9.692492	-3.586338	0.004893971
TC0900001050.mm.2	Gm10639	7.791844	9.641101	-3.603144	0.03762664
TC0100000933.mm.2	Ano7	8.092452	9.971294	-3.677799	0.0475283
TC1800000980.mm.2	Abhd3	4.394443	6.303148	-3.754719	0.03114567
TC0700002062.mm.2	Muc5ac	5.157903	7.089862	-3.815731	0.006924571
TC0300001306.mm.2	Elovl6	8.716985	10.65575	-3.833782	0.0193929
TC0300001345.mm.2	Gimd1	5.196807	7.156578	-3.890002	0.02541174
TC1500000418.mm.2	Fer1l6	4.963673	7.078897	-4.332574	0.02765471
TC0800001193.mm.2	Ces2b	6.76272	8.908267	-4.424601	0.01447274
TC0500003435.mm.2	Muc3	6.858293	9.010748	-4.445836	0.02399386
TC1100002165.mm.2	Npc1l1	4.822577	7.114001	-4.895391	0.01190189
TC1000000898.mm.2	Smim24	8.263597	10.67693	-5.327035	0.02557445
TC0900001049.mm.2	Gm3776; Gsta	9.313335	11.73277	-5.349626	0.04015579
TC1700001911.mm.2	Btnl5-ps	5.748098	8.226836	-5.574097	0.04786933
TC1400000800.mm.2	Mcpt2	9.635539	12.22005	-5.998142	0.02219602
TC0400000351.mm.2	Spink4	9.549457	12.16249	-6.11787	0.02029917
TC0200004602.mm.2	Il1b	8.969912	11.91346	-7.693029	0.01379845
TC1100004241.mm.2	Sectm1b	4.907196	8.225436	-9.974472	0.002946909
TC0500003434.mm.2	Muc3	4.635571	7.977441	-10.13919	0.03685243
TC0600001007.mm.2	Gkn2	14.54484	18.40173	-14.48909	0.01632712
TC1400000866.mm.2	Trim13	2.958452	6.904336	-15.41095	1.21E-06
TC1900001131.mm.2	Ms4a7	8.16516	12.12375	-15.54725	0.02239786
TC0600002653.mm.2	Gkn1	13.21059	17.48947	-19.41203	0.02330218
TC1500001849.mm.2	Lgals2	8.975722	13.37199	-21.05756	0.004774563
TC1400002302.mm.2	Adamdec1	3.920724	8.406153	-22.40004	9.23E-05
TC0300003084.mm.2	Clca1	11.25409	15.95408	-25.99191	0.000569725
