# Supplementary material for: Nutrient restriction-activated Fra-2 promotes tumor progression via IGF1R in miR-15a downmodulated pancreatic ductal adenocarcinoma
Source: Signal Transduct Target Ther. 2024 Feb 12;9:31. doi: 10.1038/s41392-024-01740-4 (PMC10859382; doi:10.1038/s41392-024-01740-4)
Supplement: Supplementary file 6 — original and uncropped films of Western blots [file 41392_2024_1740_MOESM6_ESM.pptx]

## Slide 1
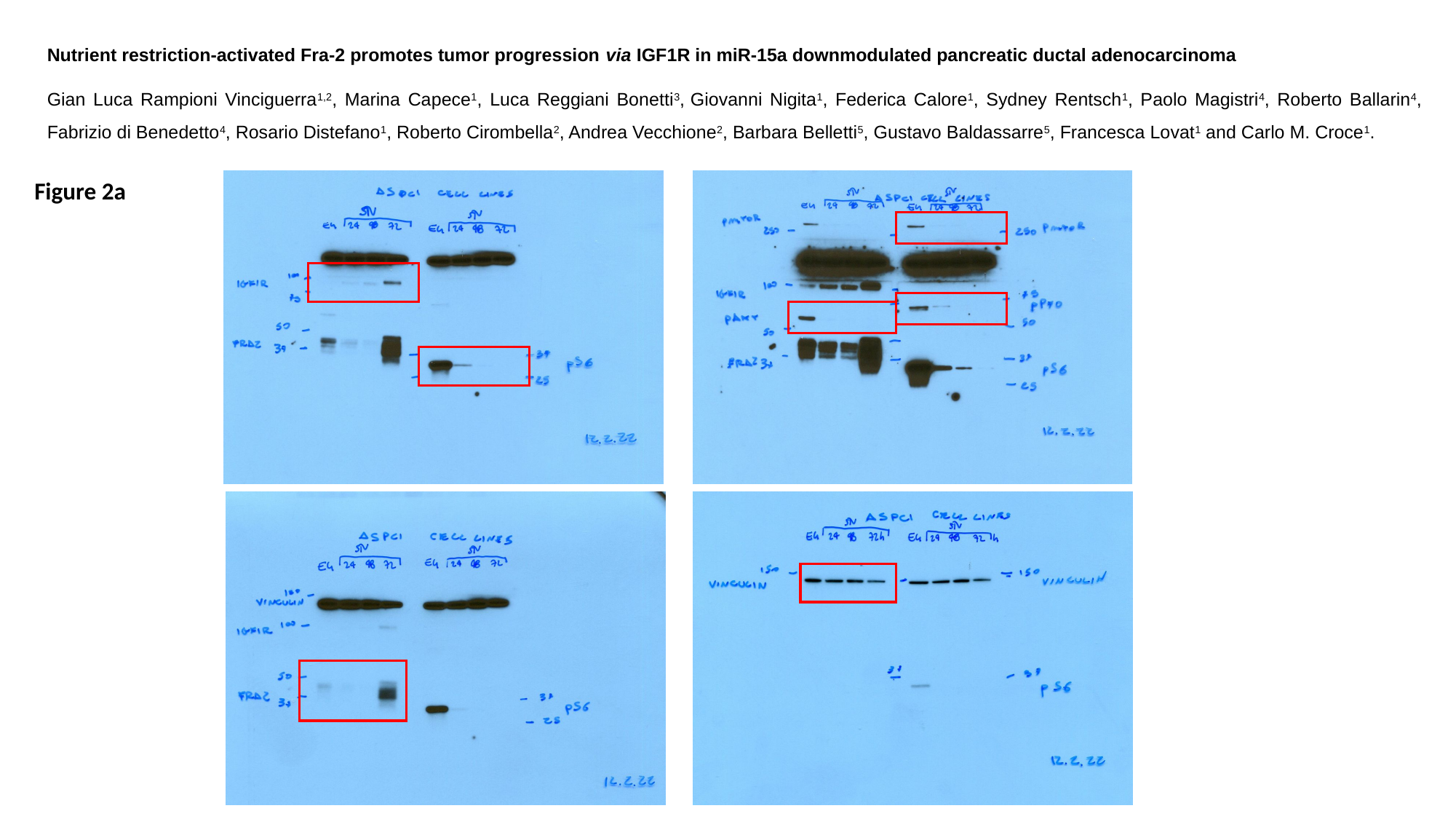

Nutrient restriction-activated Fra-2 promotes tumor progression via IGF1R in miR-15a downmodulated pancreatic ductal adenocarcinoma
Gian Luca Rampioni Vinciguerra1,2, Marina Capece1, Luca Reggiani Bonetti3, Giovanni Nigita1, Federica Calore1, Sydney Rentsch1, Paolo Magistri4, Roberto Ballarin4, Fabrizio di Benedetto4, Rosario Distefano1, Roberto Cirombella2, Andrea Vecchione2, Barbara Belletti5, Gustavo Baldassarre5, Francesca Lovat1 and Carlo M. Croce1.
Figure 2a

## Slide 2
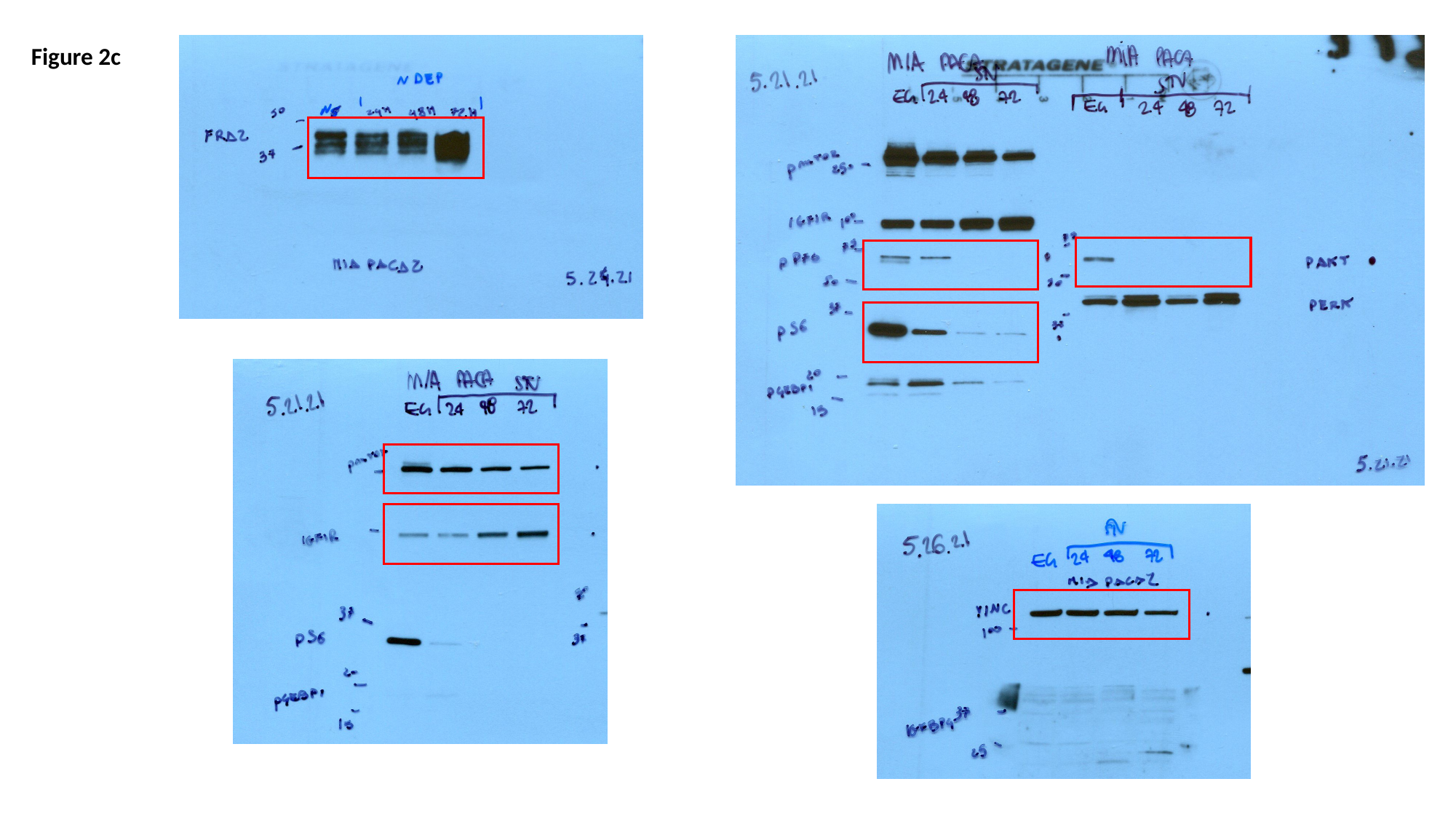

Figure 2c

## Slide 3
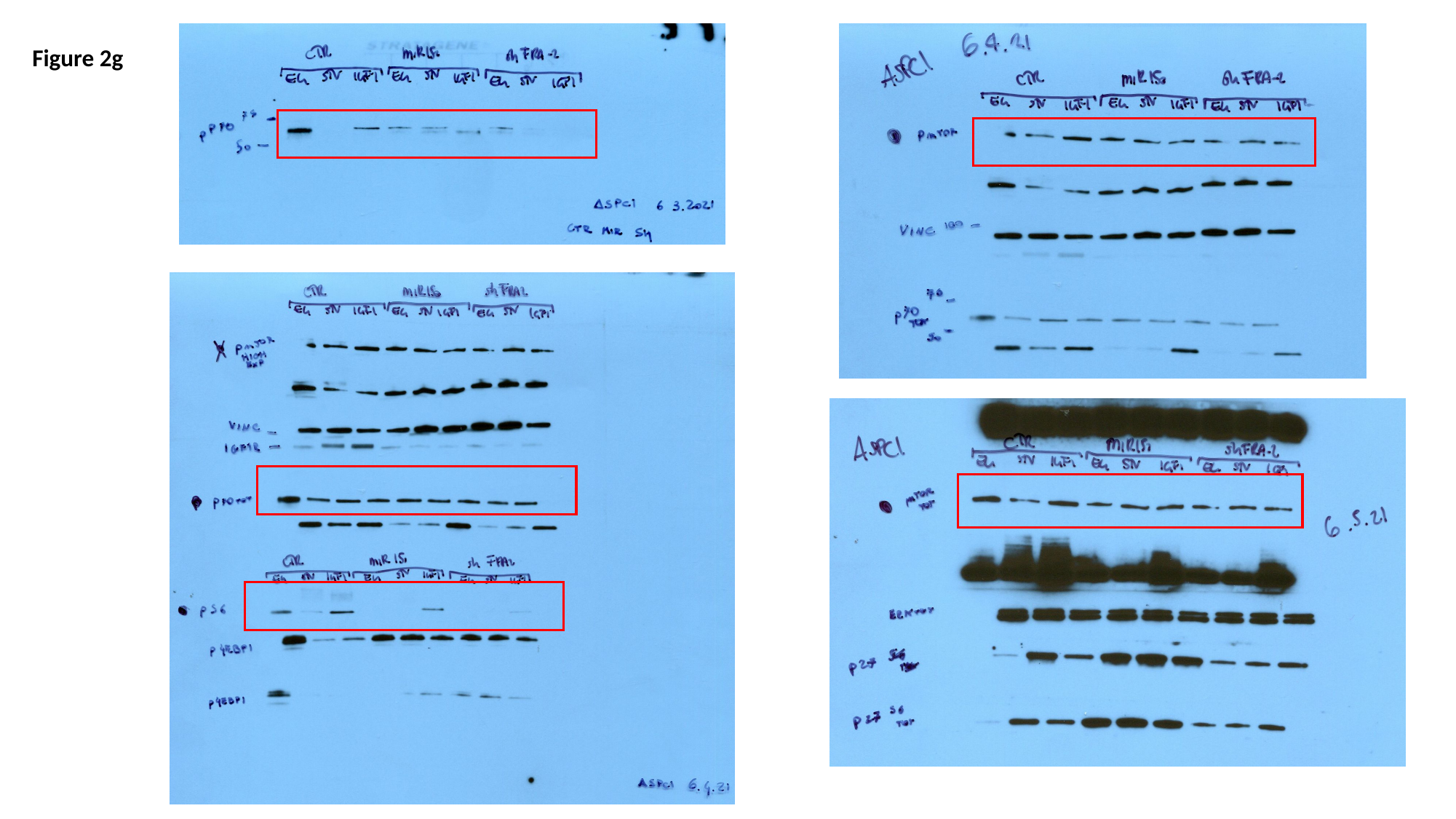

Figure 2g

## Slide 4
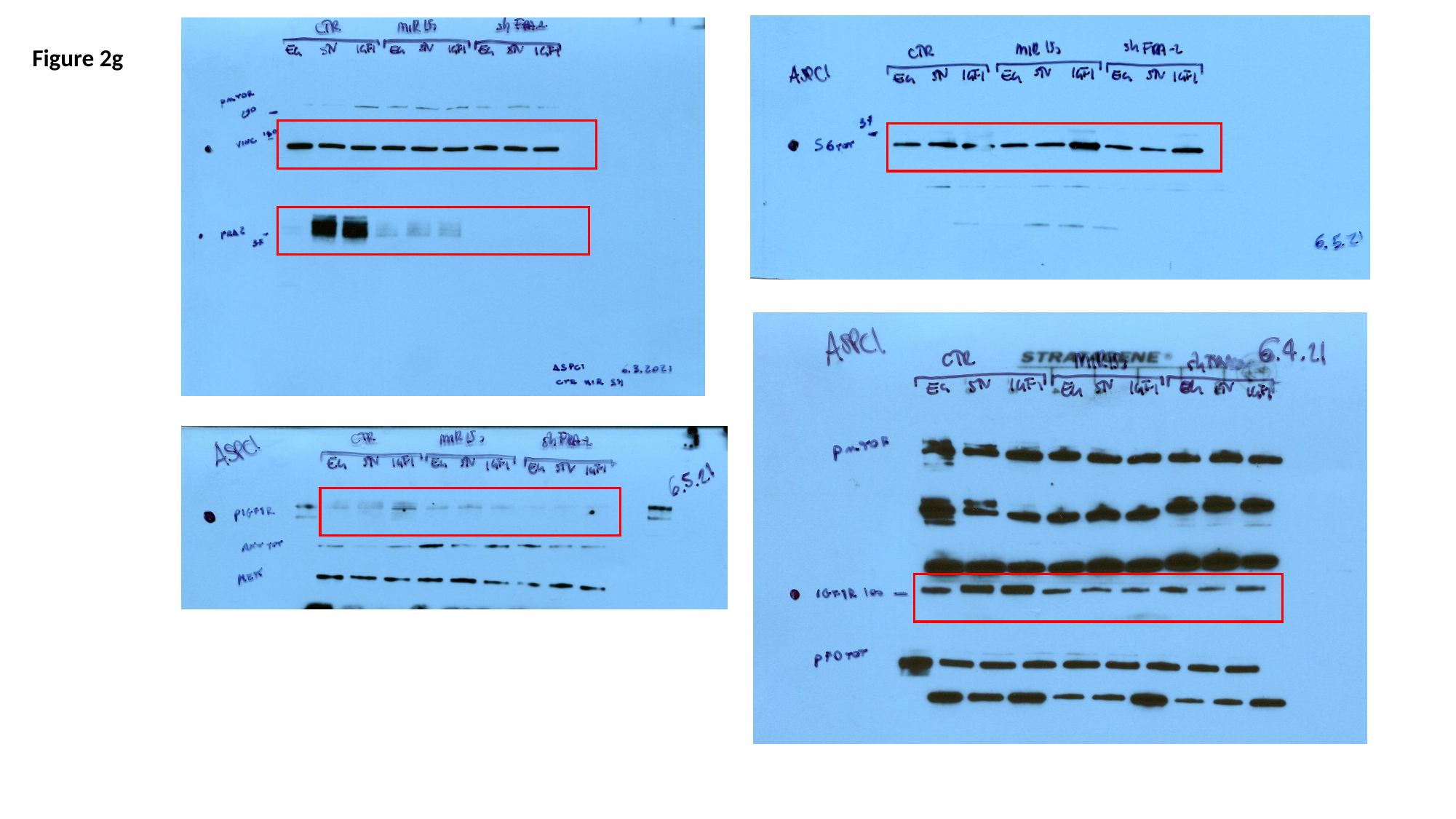

Figure 2g

## Slide 5
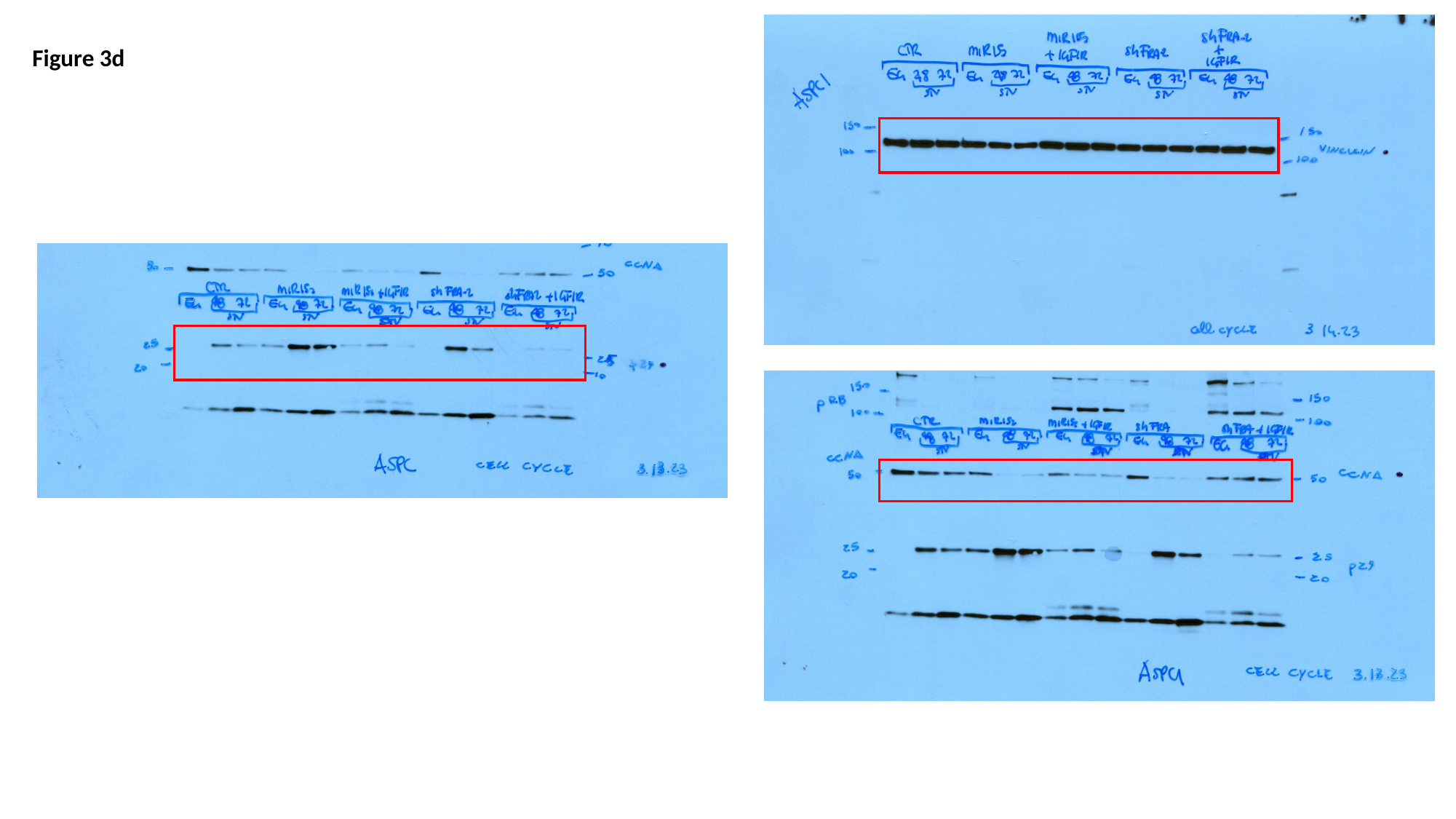

Figure 3d

## Slide 6
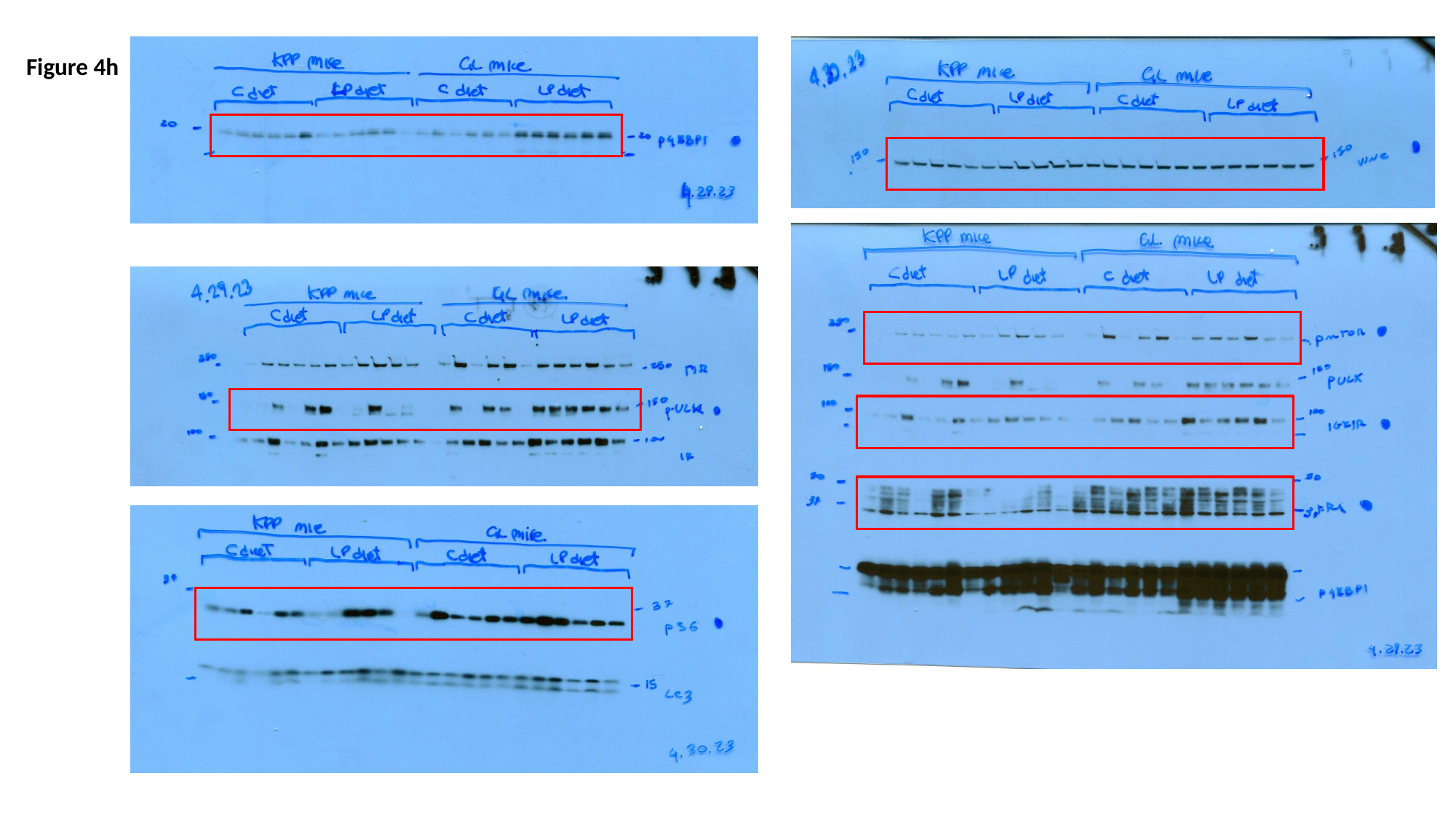

Figure 4h

## Slide 7
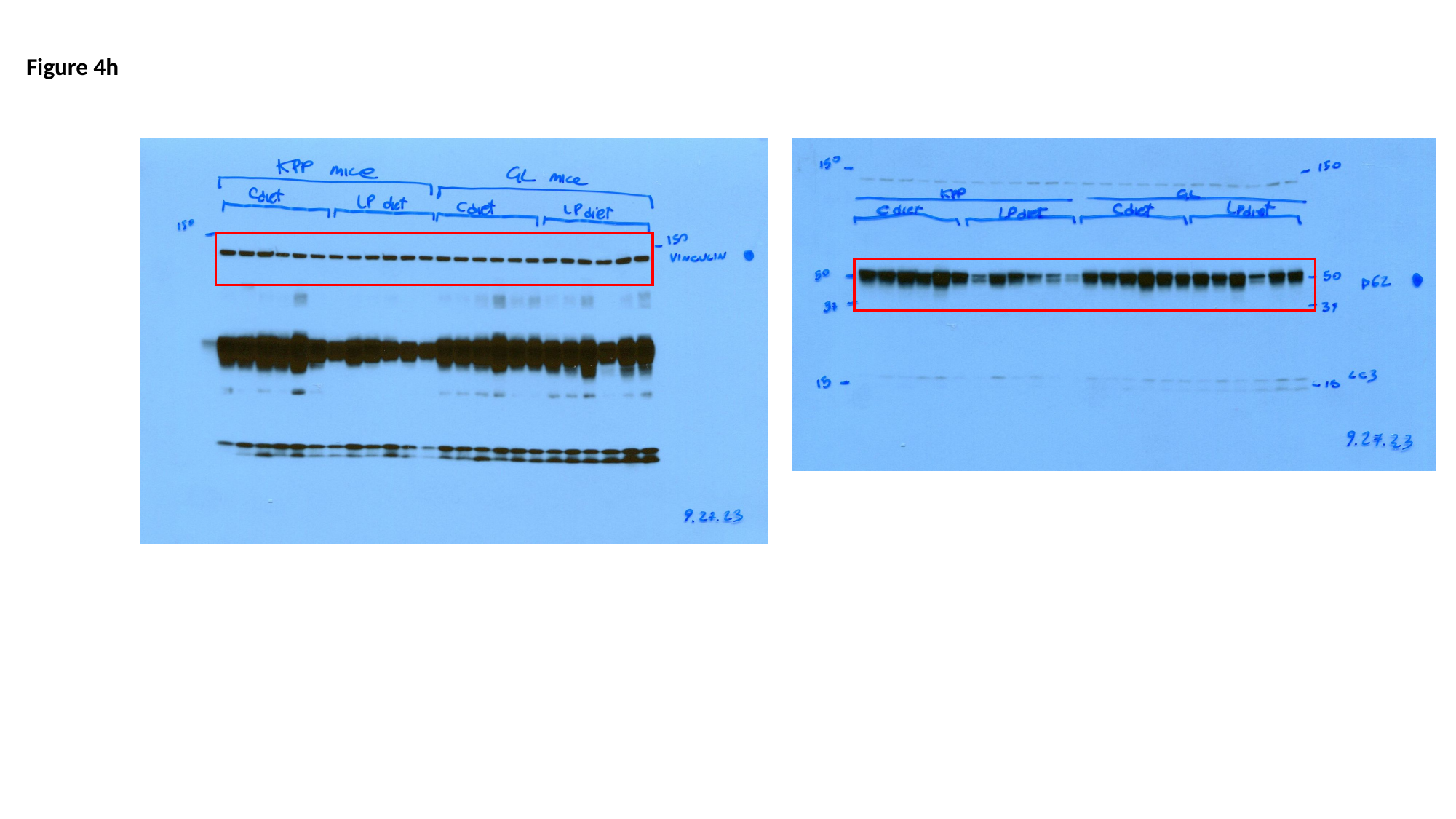

Figure 4h

## Slide 8
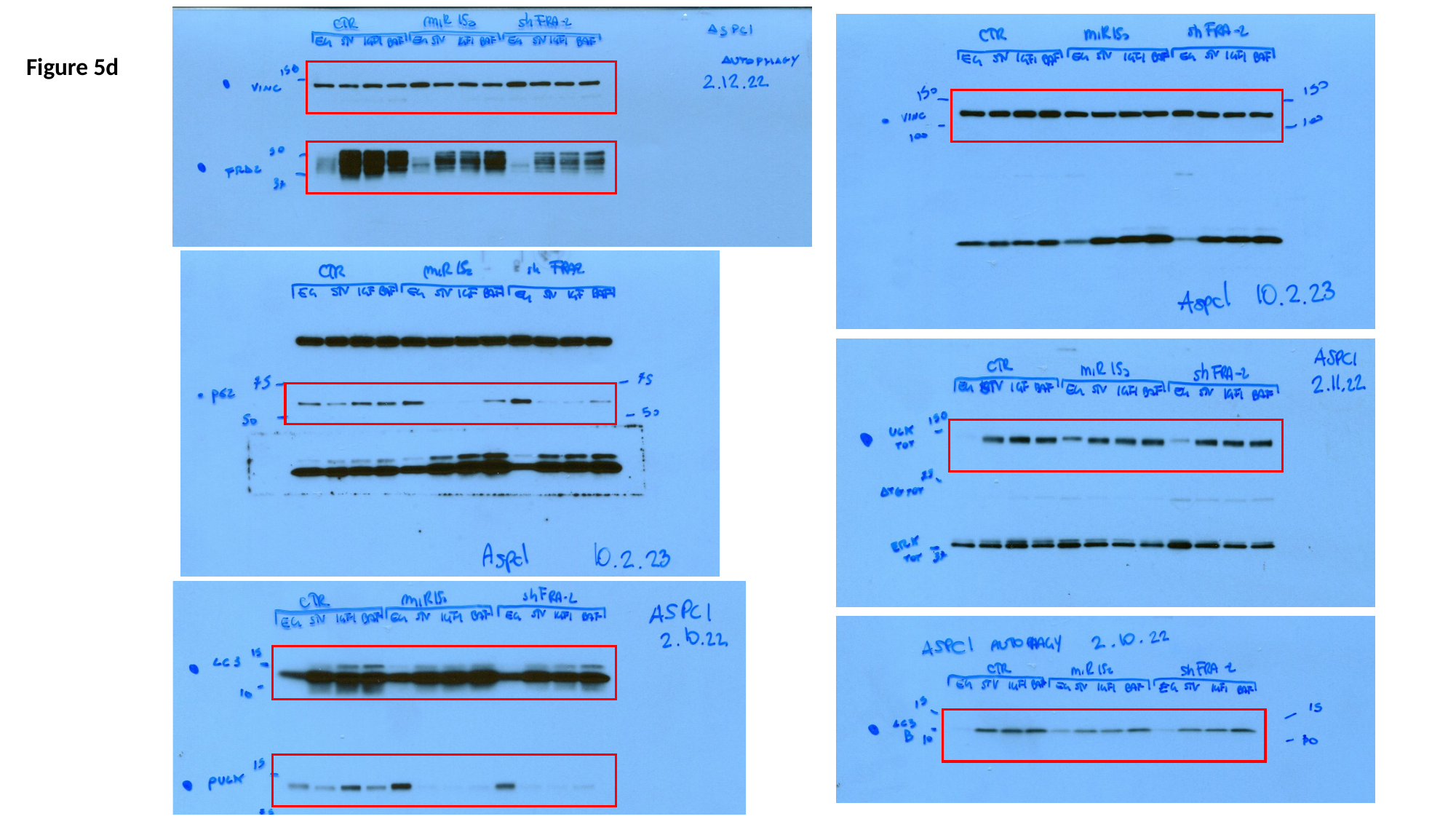

Figure 5d

## Slide 9
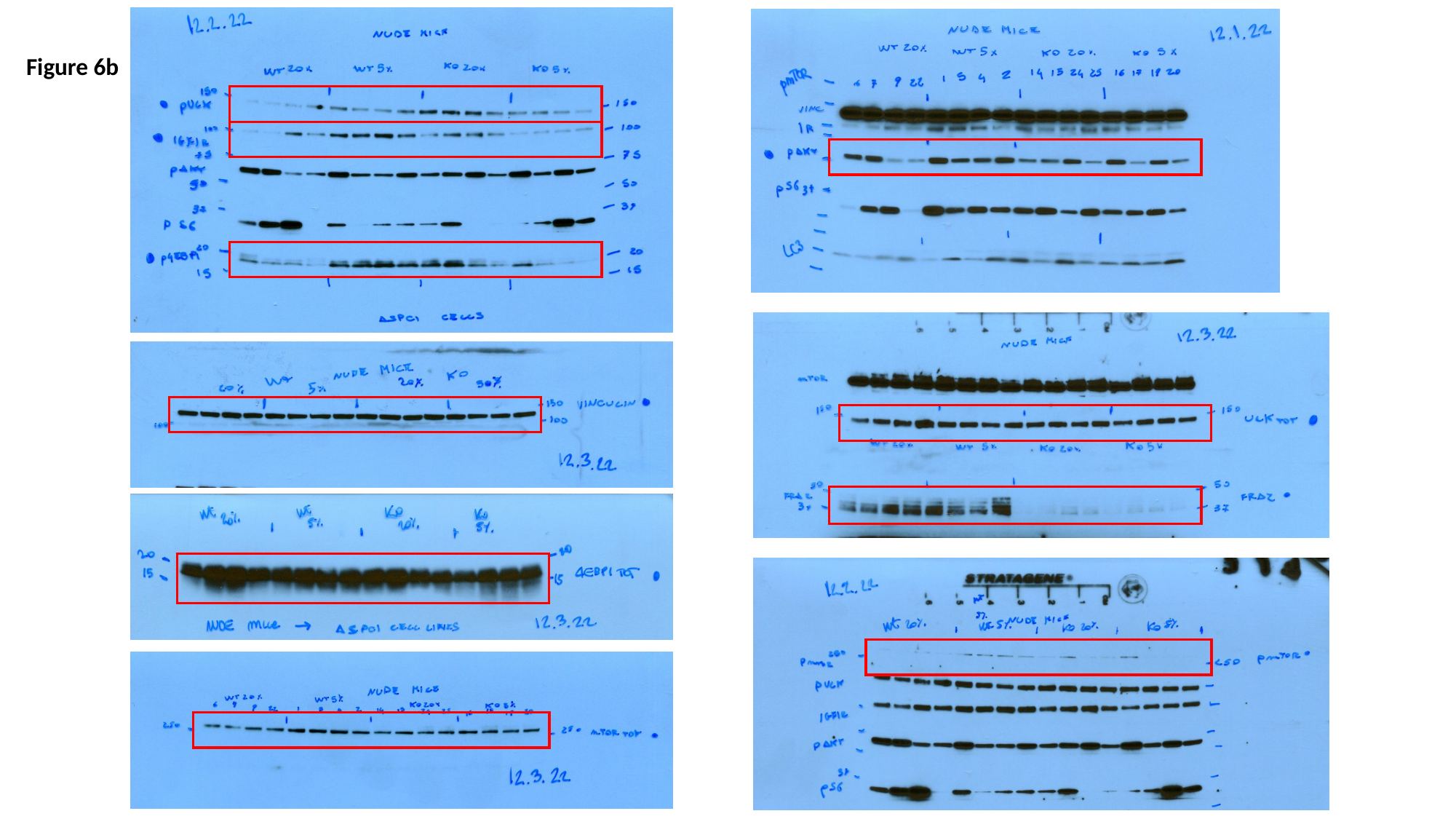

Figure 6b

## Slide 10
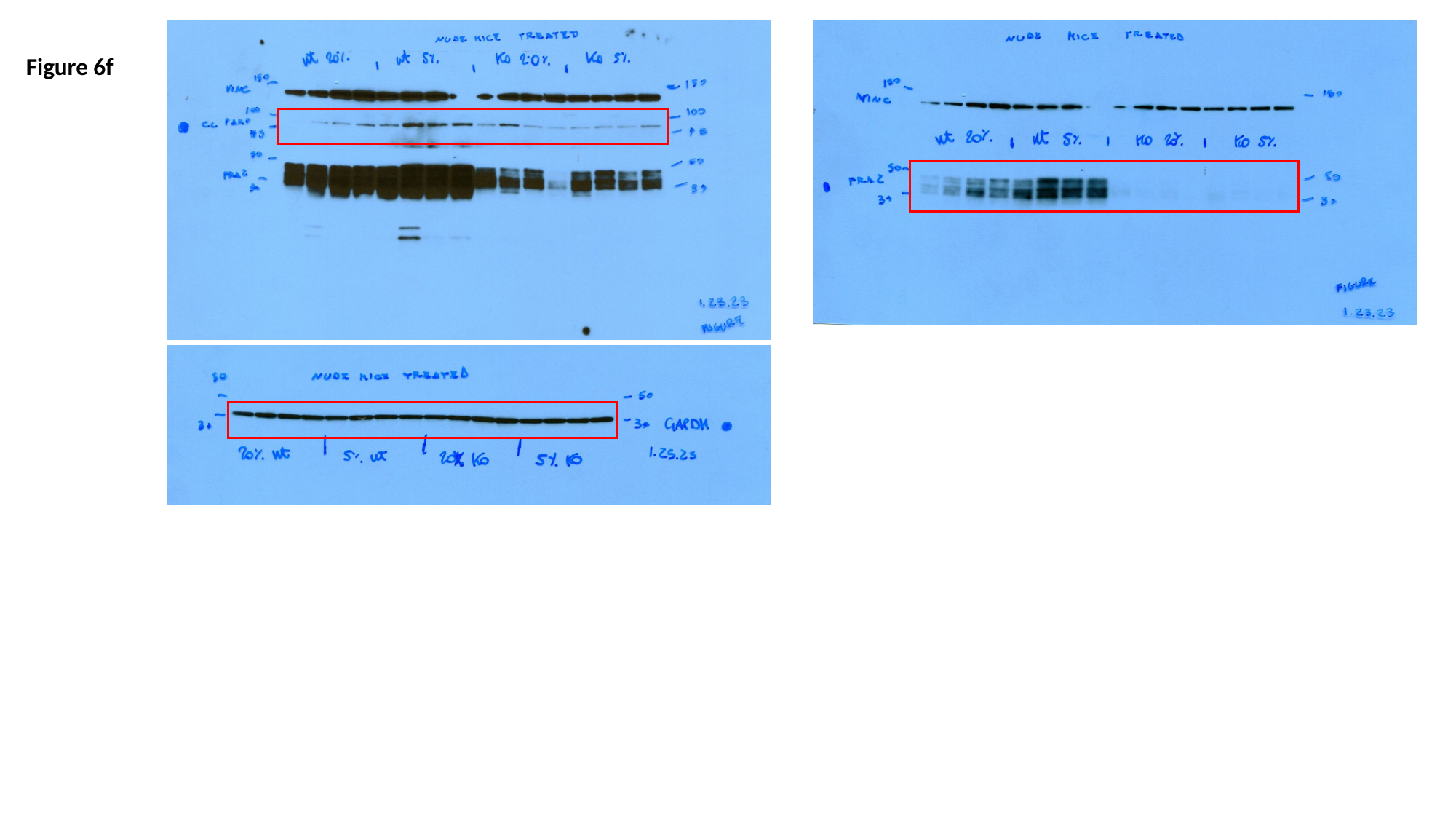

Figure 6f

## Slide 11
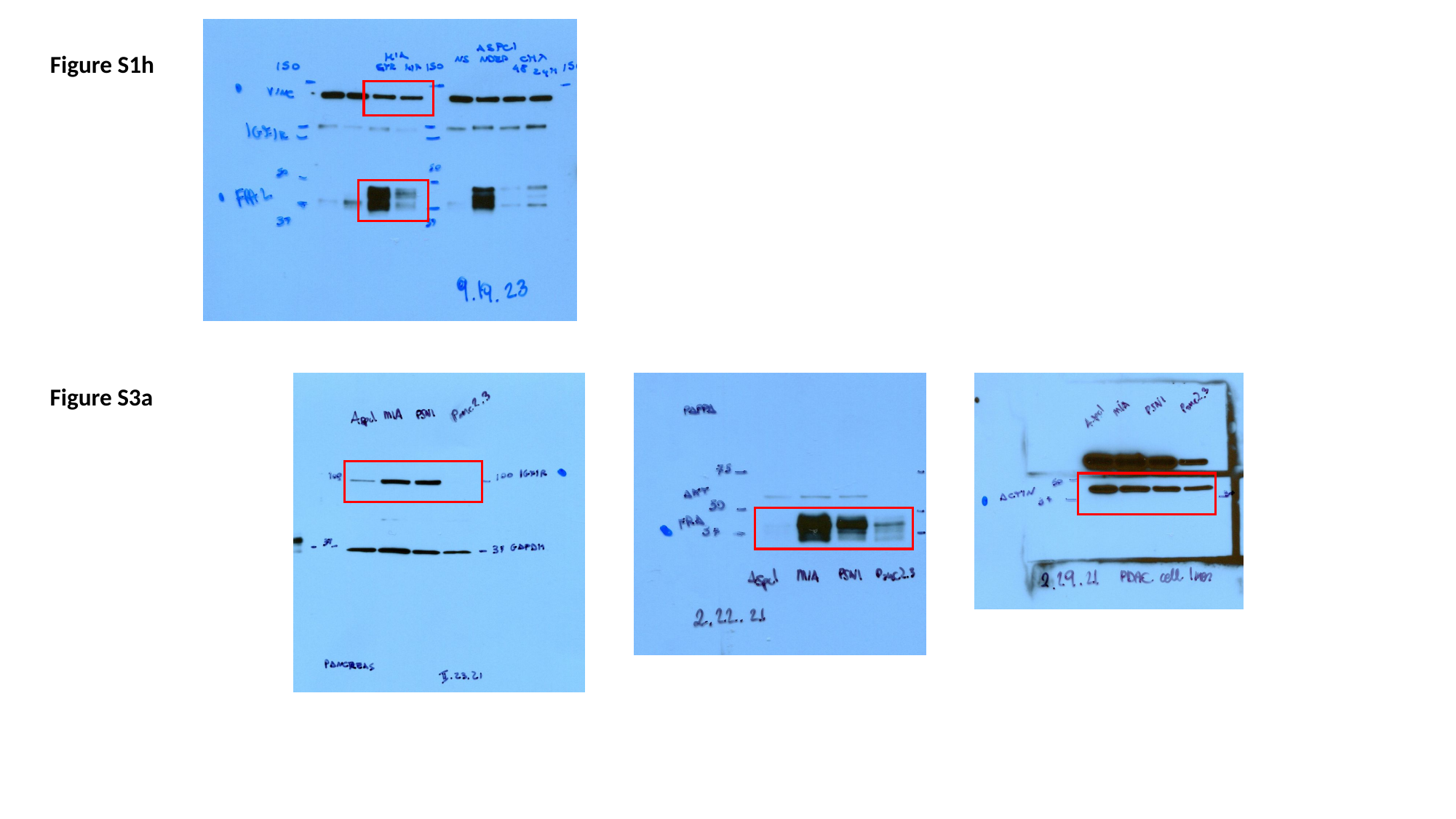

Figure S1h
Figure S3a

## Slide 12
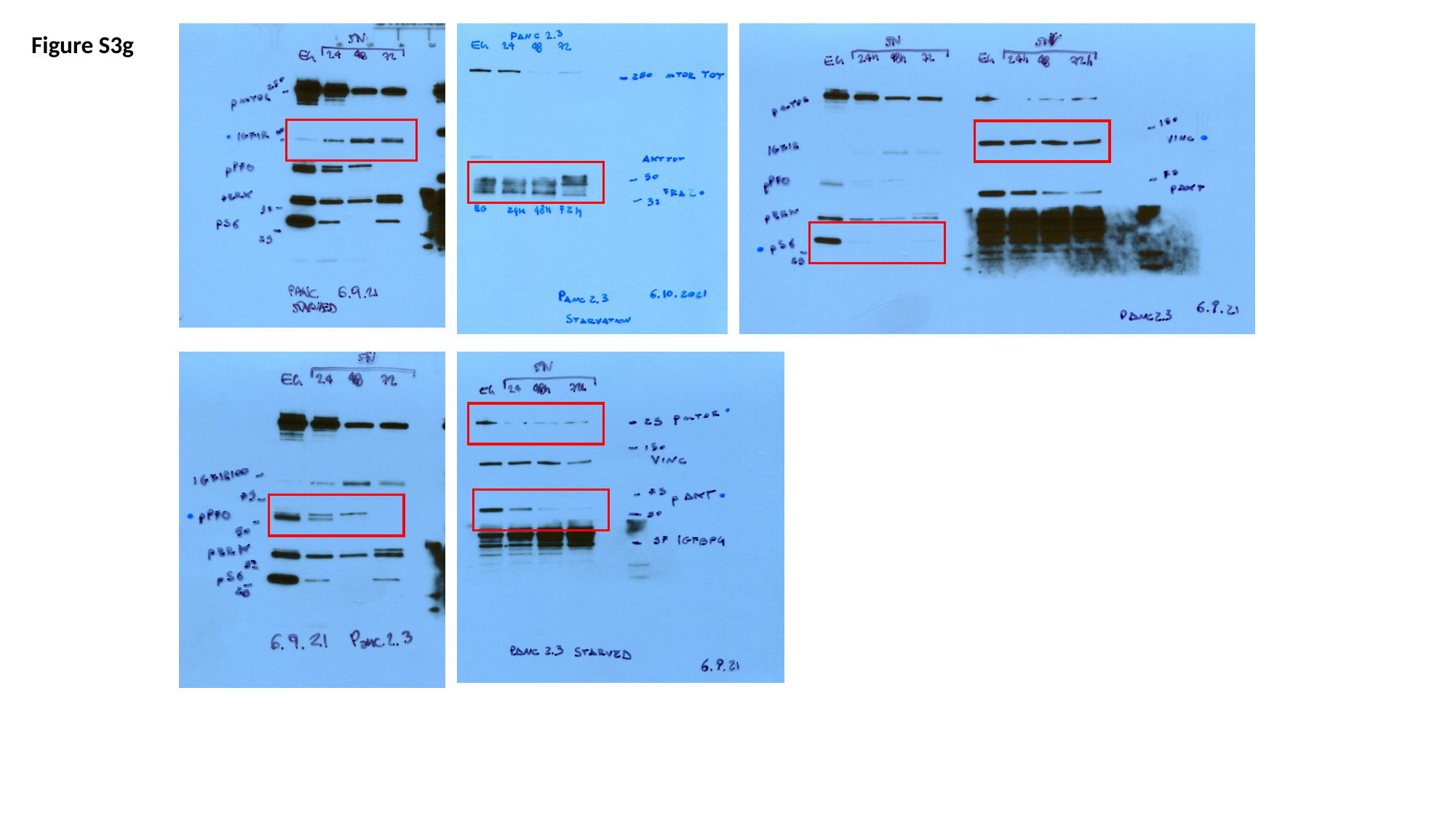

Figure S3g

## Slide 13
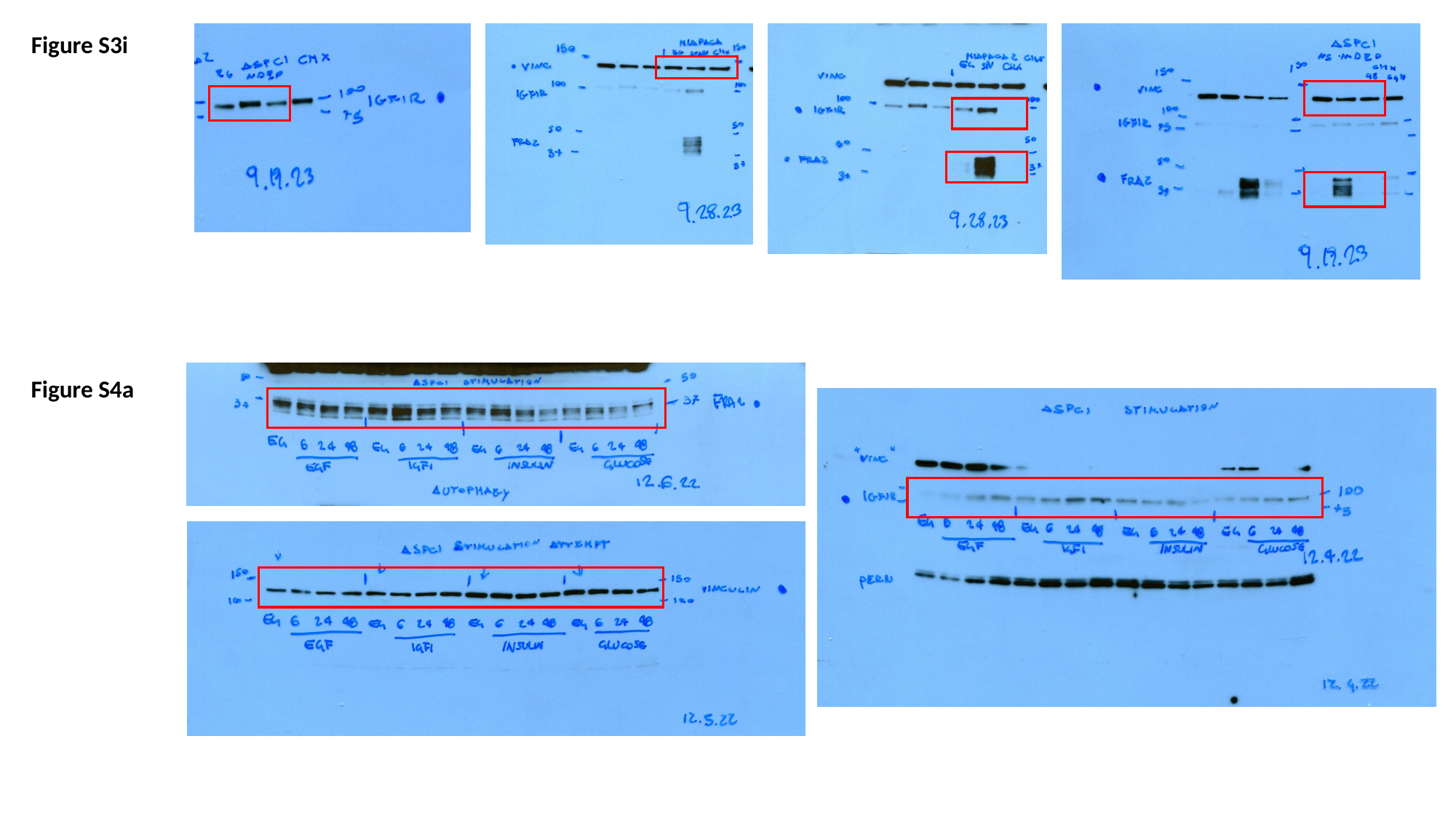

Figure S3i
Figure S4a

## Slide 14
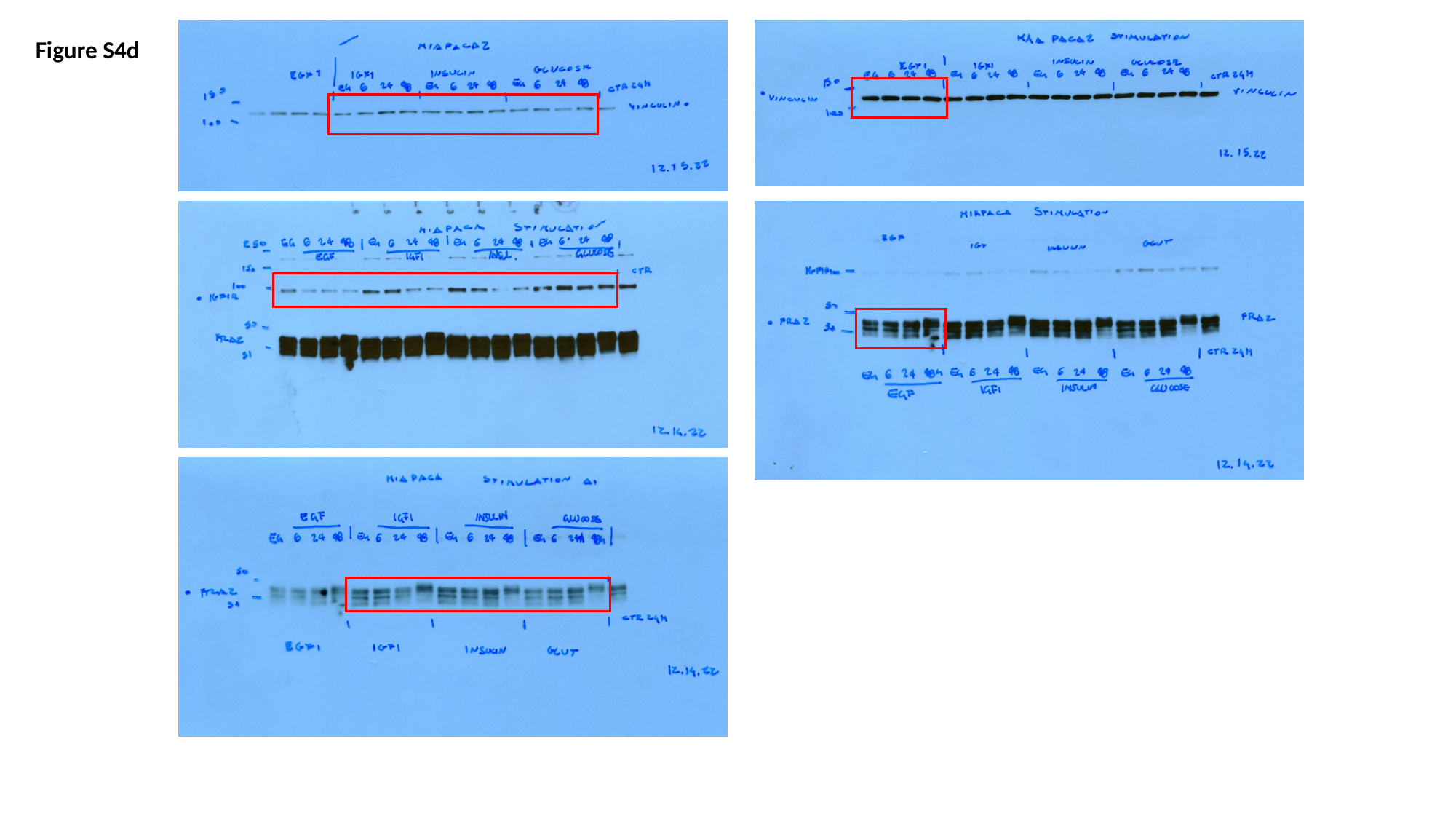

Figure S4d

## Slide 15
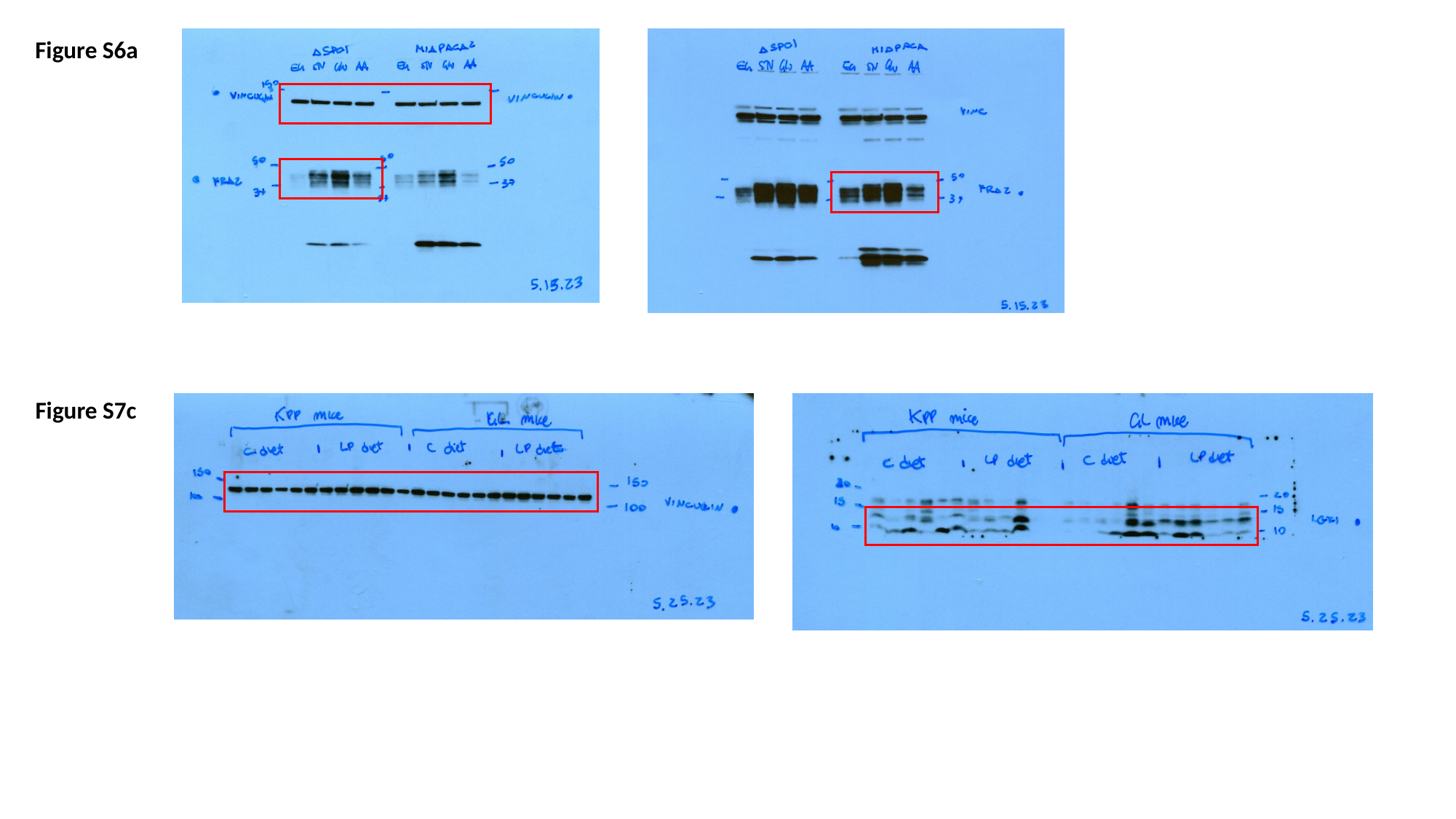

Figure S6a
Figure S7c

## Slide 16
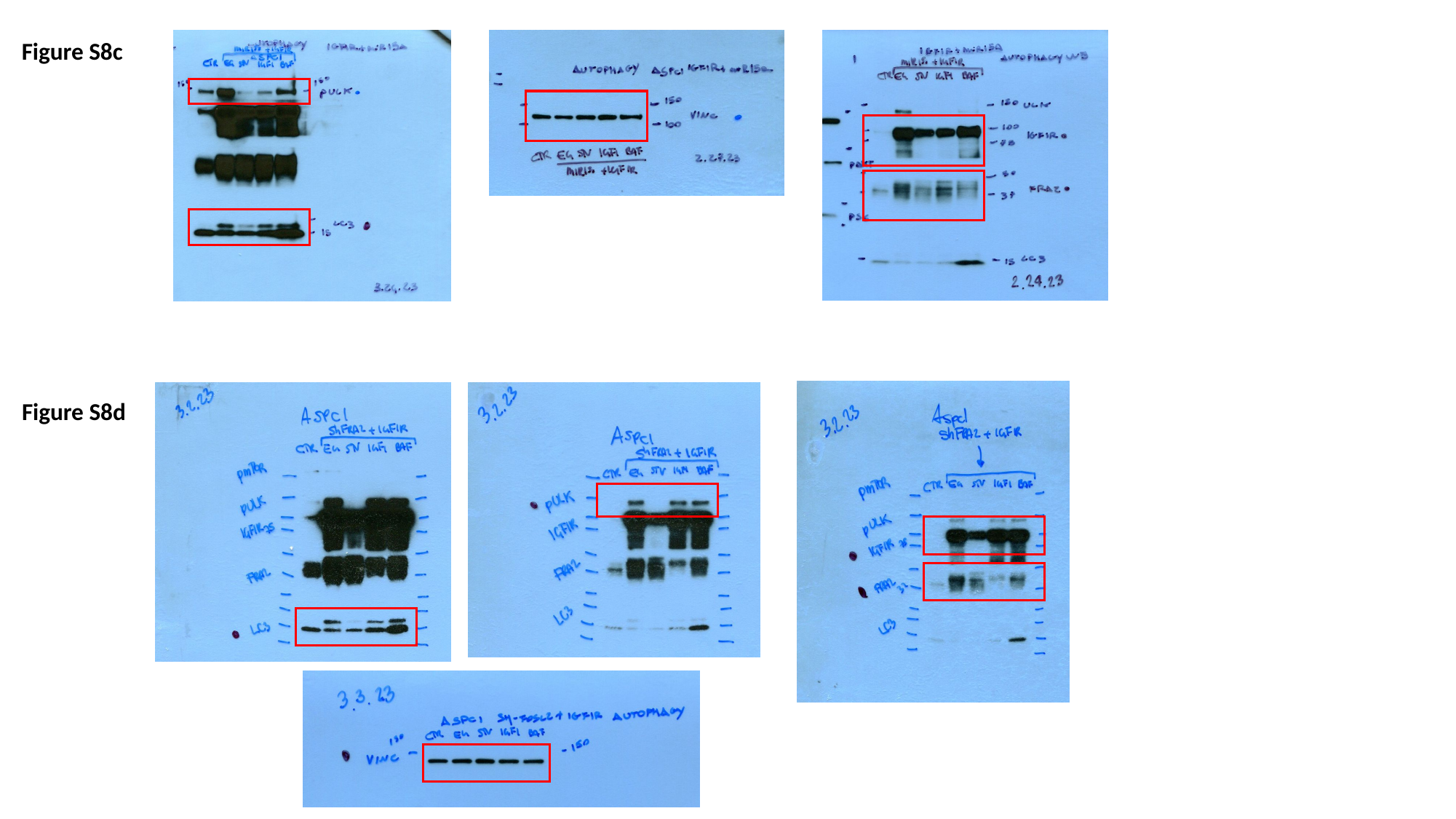

Figure S8c
Figure S8d

## Slide 17
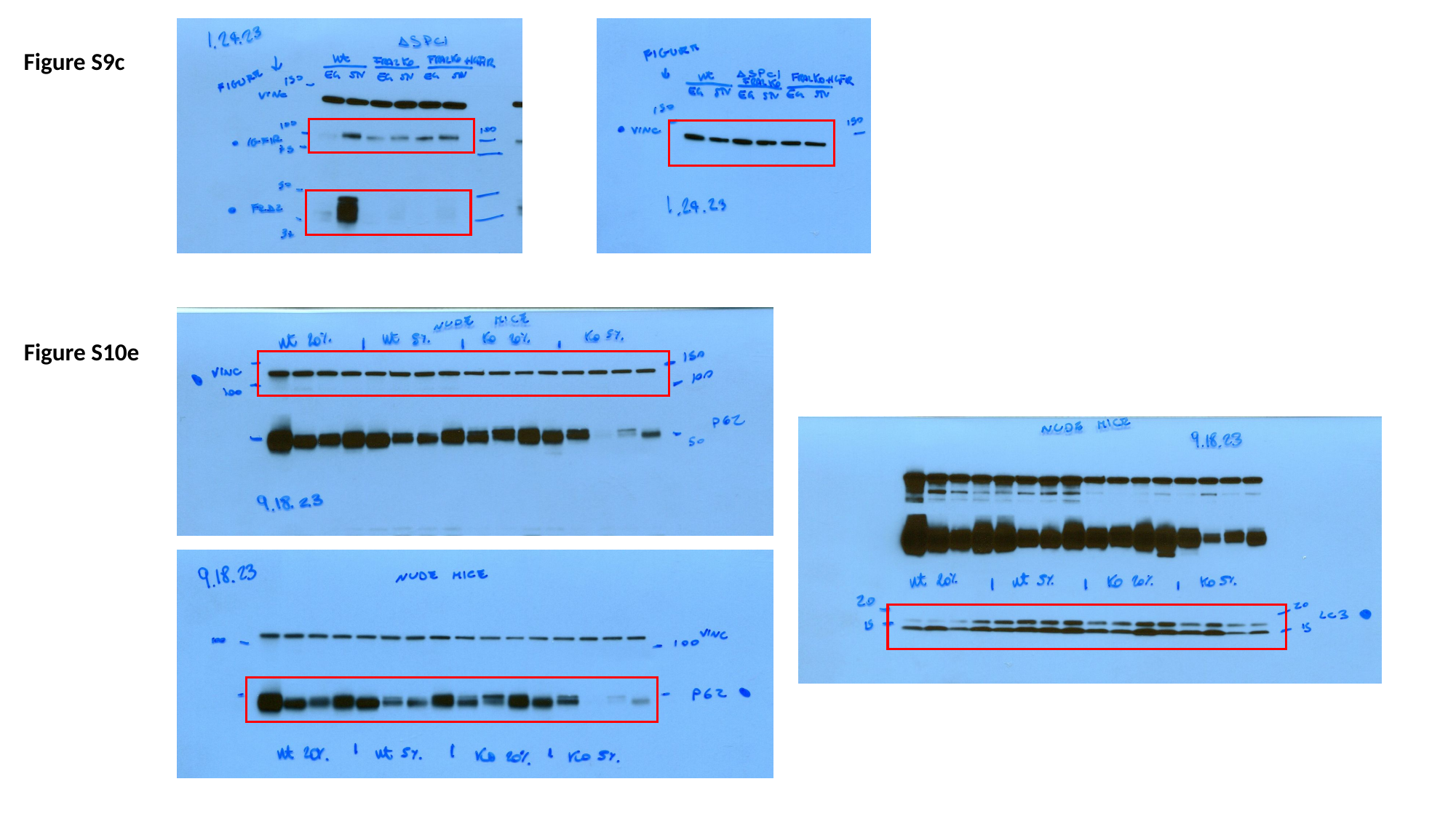

Figure S9c
Figure S10e

## Slide 18
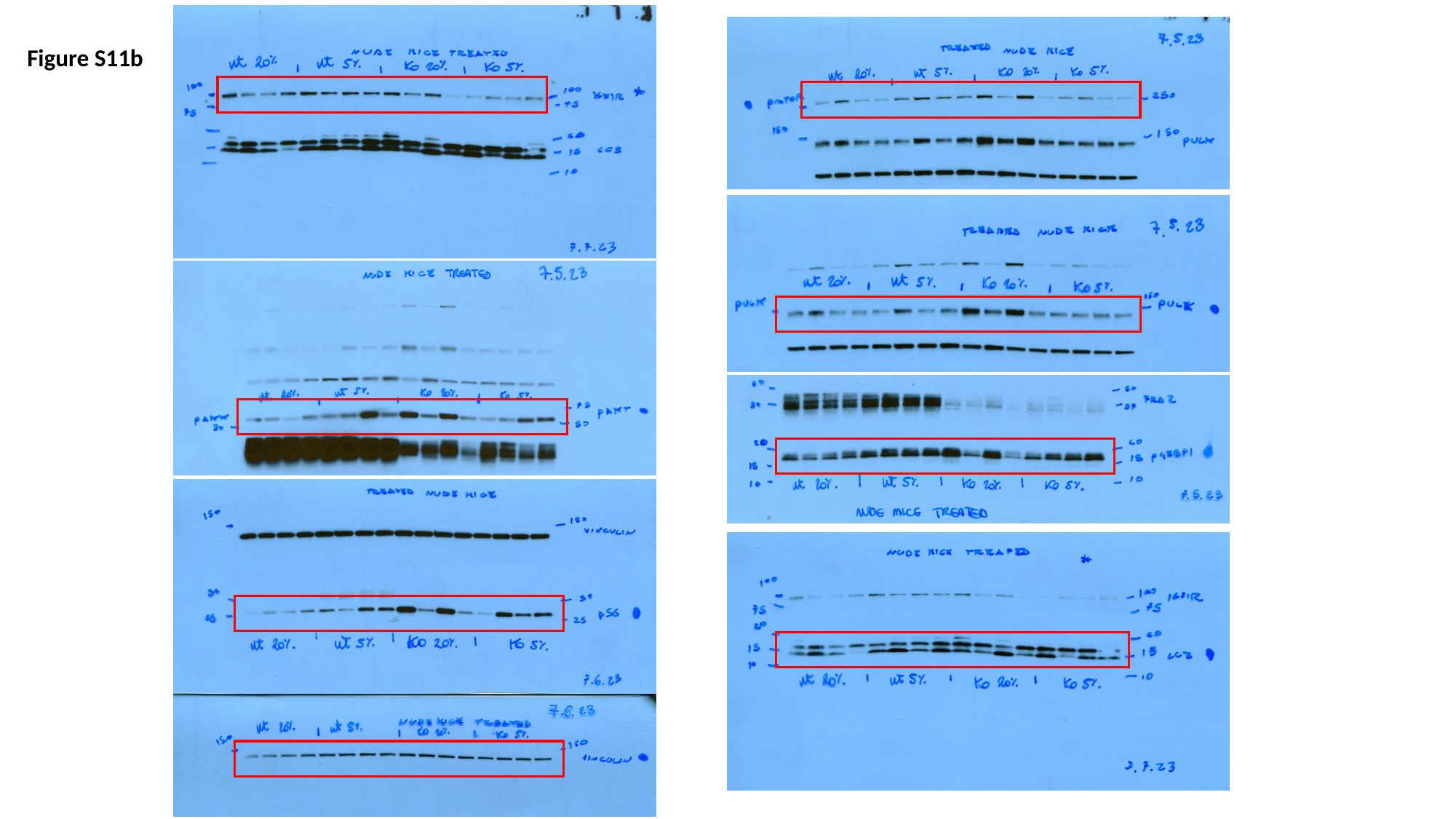

Figure S11b
